# Elephants as an animal model for self-domestication

**DOI:** 10.1073/pnas.2208607120

**Published:** 2023-04-03

**Authors:** Limor Raviv, Sarah L. Jacobson, Joshua M. Plotnik, Jacob Bowman, Vincent Lynch, Antonio Benítez-Burraco

**Affiliations:** ^a^The Language Evolution and Adaptation in Diverse Situations (LEADS) Group, Language & Genetics Department, Max Planck Institute for Psycholinguistics, Nijmegen 6525 XD, The Netherlands; ^b^Centre for Social, Cognitive and Affective Neuroscience, School of Psychology and Neuroscience, University of Glasgow, Glasgow G12 8QB, UK; ^c^Department of Computer Science, Artificial Intelligence Lab, Vrije Universiteit 1050 Brussels, Belgium; ^d^Department of Psychology, The Graduate Center, City University of New York, New York, NY 10016; ^e^Department of Psychology, Hunter College, City University of New York, New York, NY 10065; ^f^Department of Biological Sciences, University at Buffalo The State University of New York (SUNY), Buffalo, NY 14260-4610; ^g^Department of Spanish, Linguistics, and Theory of Literature, University of Seville 41004 Seville, Spain

**Keywords:** elephants, self-domestication, evolution, prosociality, cross-species comparisons

## Abstract

Humans are unique in their sophisticated culture and societal structures, their complex languages, and their extensive tool use. According to the human self-domestication hypothesis, this unique set of traits may be the result of an evolutionary process of self-induced domestication, in which humans evolved to be less aggressive and more cooperative. However, the only other species that has been argued to be self-domesticated besides humans so far is bonobos, resulting in a narrow scope for investigating this theory limited to the primate order. Here, we propose an animal model for studying self-domestication: the elephant. First, we support our hypothesis with an extensive cross-species comparison, which suggests that elephants indeed exhibit many of the features associated with self-domestication (e.g., reduced aggression, increased prosociality, extended juvenile period, increased playfulness, socially regulated cortisol levels, and complex vocal behavior). Next, we present genetic evidence to reinforce our proposal, showing that genes positively selected in elephants are enriched in pathways associated with domestication traits and include several candidate genes previously associated with domestication. We also discuss several explanations for what may have triggered a self-domestication process in the elephant lineage. Our findings support the idea that elephants, like humans and bonobos, may be self-domesticated. Since the most recent common ancestor of humans and elephants is likely the most recent common ancestor of all placental mammals, our findings have important implications for convergent evolution beyond the primate taxa, and constitute an important advance toward understanding how and why self-domestication shaped humans’ unique cultural niche.

## Significance

Why did humans, and no other animal, develop the complement of complex cultures, languages, and tools? Answering this question is one of the most important endeavors of modern science, which can shed light not only on our distinctive cognitive and behavioral phenotype, but also on the evolutionary pressures that gave rise to it. A promising theory, the human self-domestication hypothesis, suggests that humans’ uniqueness is the outcome of an evolutionary process of selection against aggression. While compelling, this theory is hard to test: Besides humans, only one other species (bonobos) has been argued to be self-domesticated. Our work suggests that elephants may also be self-domesticated, leading to exciting future research on the evolutionary similarities between humans and other species beyond the primate order.

## 1.1. The Human Self-Domestication Hypothesis (HSD)

What makes us humans unique? Humans possess many remarkable traits such as sophisticated culture and social dynamics, complex communication abilities, and extensive tool use. While none of these traits are uniquely human per se, their combination seems to be distinct to our species (1). Researchers have long attempted to explain why this is the case – why did humans, but not other animals, develop this combination of complex traits? Answering this question is an important endeavor of modern science, and can shed light not only on our distinctive cognitive and behavioral phenotype, but also on the evolutionary pressures that gave rise to these complex traits. A new theory, the HSD hypothesis, suggests that humans’ distinctiveness is, to a large extent, the outcome of an evolutionary process similar to that of nonhuman animal domestication (2–4).

The HSD hypothesis builds on the finding that humans display many of the biological and behavioral features that typically characterize the outcome of domestication in other mammals such as dogs, pigs, and sheep – aka the “domestication syndrome” (2, 5–7), including smaller skulls/brains, childish facial features, less hair, prolonged childhood, increased play behavior, and particularly, less aggressive behaviors (8–11). According to the HSD hypothesis, human evolution in the middle and late Paleolithic was characterized by selective pressures for having less aggressive partners, in sexual or other social relationships. This resulted in more prosocial individuals who were more prone to interact with others (not just with their kin, but also with strangers), giving rise to increased contacts and complex community structure as well as more sophisticated teaching, learning, and experimentation (mostly through playing). Ultimately, these properties may have enabled the cultural evolution of many distinctive human traits (2), and most notably, the emergence of our complex linguistic abilities (12–15).

However, while animal domestication is directly and intentionally guided by humans via artificial selection for tameness, humans’ self-domestication is suggested to have been an organic process, likely triggered by external changes in our environment as well as internal, nondirected pressures favoring within-species prosocial behavior over aggression. In other words, domestication-like traits in humans evolved as a by-product of natural selection that favored increased in-group prosociality over aggression (2). As such, self-domestication can be defined as the exhibition of the typical features commonly associated with domestication in other animals, but without the obvious presence of another species serving as a domesticator (16–19). Out of the many factors that were suggested to trigger this selection for less aggressive behaviors in humans, the two most prominent explanations for HSD are a) changes in our foraging ecology, where humans began relying on more diverse and nonlocal food sources that resulted in a need to move around and/or share resources with others (20), and b) climate deterioration and harsh environmental conditions during the last glaciation, which have increased the need for exchanging and sharing resources between groups (21). In both cases, selection for intergroup tolerance and less aggressive individuals would have benefitted the survival of the entire population, and as such may have triggered the process of self-domestication in humans. All in all, self-domestication can be seen as a sort of cultural niche construction, in which a species (in this case, humans) reduces or redirects the impact of selective pressures that individuals experience via gene-culture coevolution (22).

## 1.2. Self-Domestication in Other Species

While isolated features of domestication have been identified in some wild animals [e.g., pigmentation in Marmoset monkeys, *Callithrix jacchus*; (23)], the only other species so far that has been argued to be self-domesticated besides humans is the bonobo (*Pan paniscus*). Bonobos show lower levels of reactive aggression compared with chimpanzees (*Pan troglodytes*), and exhibit morphological, physiological, behavioral, and psychological features that are typically found in domesticated animals (2, 16, 17, 24, 25). In bonobos, the process of self-domestication may have been triggered by relaxed feeding competition (16), and/or as the outcome of changes in social dynamics associated with founder effects, i.e., when populations move to newly available environments and need to collaborate in order to adapt (26).

Notably, some authors suggest that dogs (*Canis lupus familiaris*) may also be a self-domesticated species through a commensalism pathway (2, 16), at least in the first stages of the dog-human relationship when selection against reactive aggression enabled some wolves to feed on prey remains at human camps without being rejected by people (27, 28). Nevertheless, it is assumed that even during this early period, humans have selected against specific traits in dogs, either consciously or unconsciously (e.g., too bold/aggressive animals are killed), and that this selection continued for thousands of years (18). Moreover, since modern dog breeds are clearly the outcome of extensive and systematic breeding by humans (29–32), dogs are most often treated as a domesticated (as opposed to a self-domesticated) species. In addition, self-domestication is most crucially characterized by an increase in intragroup prosociality (as opposed to increased prosociality toward another species). In the case of dogs, however, increased prosociality is mostly seen toward humans, but not within species – supporting the idea that they are not a suitable model for self-domestication. Consequently, studying the process of self-domestication is currently limited to two primate species: humans and bonobos.

Critically, the potential difference between animals that were involuntarily domesticated by humans (e.g., pigs, sheep), animals that were perhaps only partially domesticated by humans (e.g., dogs), and animals such as humans and bonobos that were domesticated without external guidance and without selection pressures enforced by another species (i.e., self-domestication) is currently unknown. Although the end result of self-domestication and domestication processes seems to be similar, little is known about the evolutionary trajectories and physiological mechanisms that might differentiate them. No work to date has examined the potential implications of being a self-domesticated species as opposed to a domesticated one, and there has been no cross-species comparison of behavioral and genetic traits in domesticated vs. self-domesticated species. As such, finding an animal model for self-domestication beyond the primate order can provide the much-needed insight into the causes and environmental pressures that might trigger a process of self-domestication.

## 2. Elephants as a Model of Self-Domestication

**Fig. 1.**
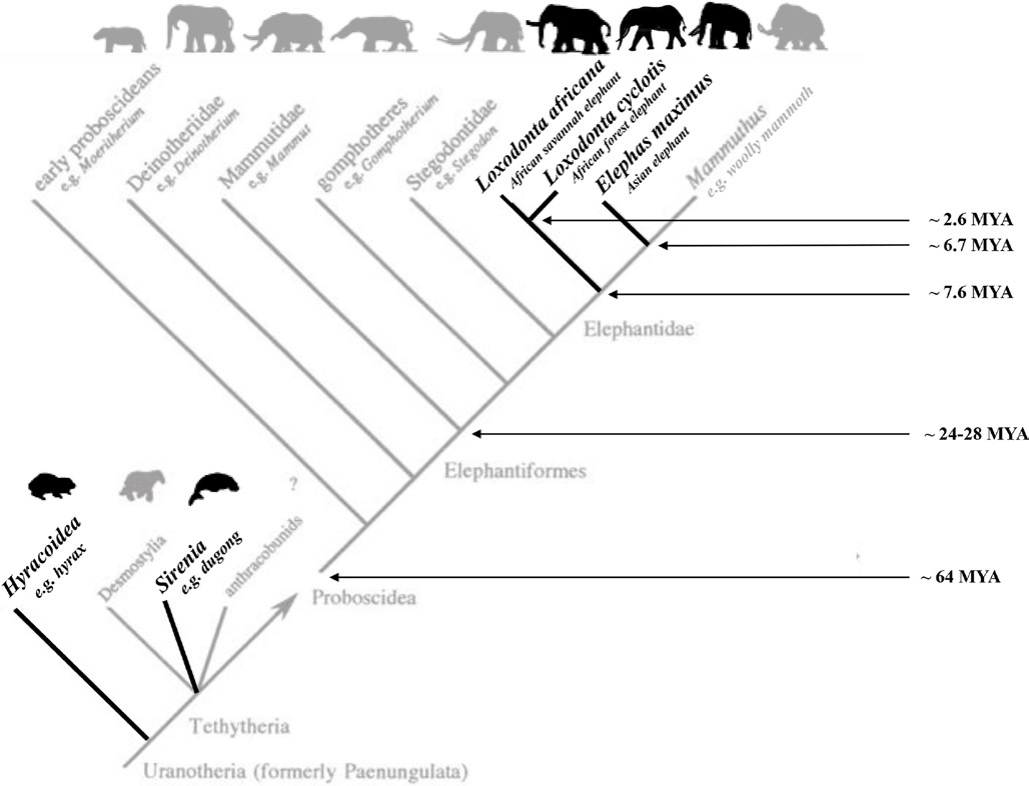
A simplified cladogram of the superorder Uranotheria demonstrating the extant (black) and extinct (gray) clades. The estimated divergence dates between the three extant species of elephants and other key evolutionary splits are also included (33, 34). Figure adapted and updated with permission from Shoshani, 1998 (35).

Elephants are the largest land animals, and consist of three different species: African savanna elephants (*Loxodonta africana)* and African forest elephants (*Loxodonta cyclotis)* in Africa, and Asian elephants (*Elephas maximus*) in Asia. They are the only extant members of their order, which also includes mastodons and mammoths (Fig. 1).

Although there are some physiological differences across species such as in their ear shape, tusk presence, and overall size (36, 37), the three species are often grouped together when it comes to their behavioral, social, and cognitive traits: All elephant species live in complex fission–fusion social groups where adult females cooperate in group defense, care of related offspring, and resource acquisition (38–41). The basic unit of social association in elephants consists of an adult female with dependent offspring, and these units associate with each other to form larger groups of both related and unrelated females in multilevel or multitiered societies (42). While African savanna family groups of two or more mother–offspring units associate over 70% of the time (43), Asian elephants appear to have more variable social groupings, yet still maintain long-term associates (44). Communication is well developed across sensory modalities and an important part of social life for all species (45). Elephants are generalist foragers, primarily relying on browse but also some grasses (46).

The core proposal of the current paper is that elephants are self-domesticated, and have thus undergone a similar evolutionary process to humans and bonobos. Since the most recent common ancestor of humans and elephants is likely the most recent common ancestor of all placental mammals (an as yet unidentified species) (47), comparing the process of self-domestication in these evolutionarily distant species can lead to important insights about convergent evolution and the process of self-domestication beyond primates (15).

In this proposal, we first discuss the cognitive, behavioral, and physiological similarities between elephants and the two other species that have been put forth as self-domesticated, namely, humans and bonobos (Table 1; section 2.1). By synthesizing existing data from various sources, Table 1 serves as an extensive cross-species comparison of elephants, humans, and bonobos with respect to classic features of self-domestication, which we review in more detail below. Crucially, this comparison provides ample supportive evidence for our hypothesis: African and Asian elephants display many of the hallmark outcomes of self-domestication, and show striking similarities with the other two self-domesticated species.

**Table 1.** Cognitive, behavioral, and physiological similarities between humans, bonobos, and elephants

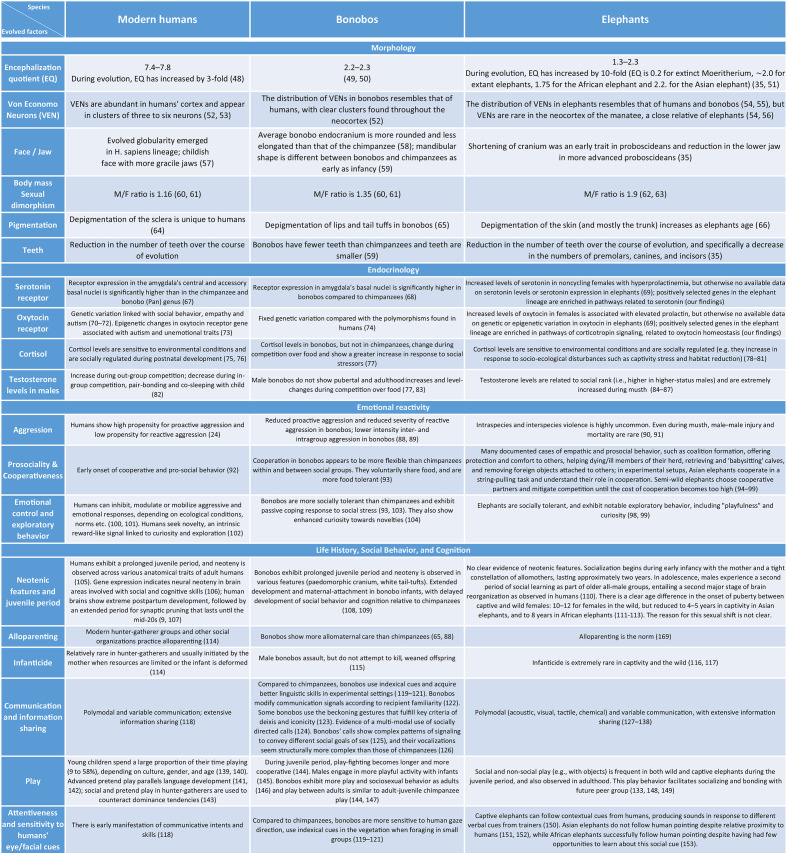

This table is adapted with permission from Shilton et al. (162), and expanded to include elephant data and additional features not considered in the original paper. It includes a cross-species comparison describing the presence of 19 relevant features in humans, bonobos, and elephants. Whenever possible, comparisons are made between a species and their distant relatives. Note that due to the lack of sufficient data on elephants, not all traits can be properly assessed beyond the anecdotal level. Thus, more research is required in order to strongly establish the presence of these traits and evaluate their complexity in elephants.

While elephants are an understudied taxa in general, existing evidence suggests similar behavioral and physiological traits across the three species, as reviewed in Table 1. As such, we hypothesize that all species of elephants have undergone self-domestication. While some Asian elephants were likely tamed as “beasts of burden” centuries ago (155), there is no evidence that these wild elephants were ever subjected to the pressures of artificial selection by humans, and no evidence that the use of captive Asian over African elephants is based on their temperament or prosociality. Asian elephants were likely captured from the wild and used in captivity due to a human need for large, strong animals to assist with a number of activities (particularly war, but later logging and tourism), and the close proximity of growing kingdoms in Asia to wild elephant populations and long-standing traditional knowledge about human-elephant relationships (156). Since elephants found in captivity and in the wild are thought to be genetically indistinguishable (155), and since domestication is a multigenerational process spanning many consecutive events of selective breeding toward reduced aggression, this never occurred systematically in any elephant species.

Given the fact that the three surviving species of elephants diverged from a common ancestor ~7 Mya (157), yet all show similar features associated with self-domestication; the implication is that these features were likely present in their common ancestor as well. The proposed process of self-domestication may in fact be a very old one in the elephant lineage, dating back before the divergence of the wooly mammoths. Interestingly, mammoths may also have shown hallmark features of self-domestication such as prosocial behavior (158). In contrast, bonobos and chimpanzees, which diverged approximately 1 to 2 Mya (159), do not share all of the characteristic features reviewed in Table 1. This suggests that similarities in behavior, social structure, and environmental pressures may matter more for characterizing self-domestication than just the length of time that has passed between an evolutionary split.

Given that our cross-species comparison of cognitive, behavioral, and physiological traits suggests that elephants may have indeed undergone a process of self-domestication, we developed a priori predictions about finding genetic and molecular markers of domestication in their genome as well. Specifically, since domestication syndrome is associated with selection and overrepresentation in known lists of genes, our next step was to test the hypothesis that elephants should display selection and enrichment in at least some of these genes. We ran genetic analyses to expose the positive selection of candidate genes for domestication in African elephants, for which a high-quality genome exists (*SI Appendix, Supplemental File 13*; section 2.2). Our analyses provided empirical evidence for our hypothesis: They showed that genes positively selected in African elephants are enriched in pathways that are likely involved in domestication, and that several candidate genes associated with domestication have been positively selected in African elephants. Together, these analyses provide convincing support for the idea that elephants are also self-domesticated.

Lastly, in light of the evidence presented in sections 2.1 and 2.2, we go on to discuss several likely reasons for why a self-domestication process may have been triggered in the elephant lineage (section 2.3). Specifically, we propose three nonmutually exclusive explanations for elephants’ reduced aggression and increased prosociality, namely, a secure environment that reduces survival pressures, a harsh environment promoting the need to cooperate, and founder effects associated with the colonization of new areas.

### 2.1. Cognitive, Behavioral, and Physiological Features of Self-Domestication in Elephants: Synthesis of Existing Evidence.

Evidence for the self-domestication of elephants is summarized in Table 1, which is a cross-species comparison adapted from Shilton et al. (154) and expanded to include elephant data and additional features not considered in the original paper. Specifically, the table includes a comparison of an extensive (yet not exhaustive) list of 19 relevant cognitive, behavioral, and physiological features. It describes the presence of these features in humans and bonobos, and, whenever possible, includes a comparative description of these traits in elephants as well. Notably, unlike bonobos who can sometimes be directly compared with their potentially nondomesticated close relatives (i.e., chimpanzees), we can rarely make comparisons in the case of the elephant species and humans, as they are the only surviving species in their respective genera (160, 161), and do not have a nondomesticated close relative. While much of the evidence we review in this table is anecdotal or observational, it paints a picture in which elephants indeed exhibit many of the important features associated with self-domestication, similar to those observed in humans and bonobos. Nonetheless, more dedicated research is clearly needed in order to strongly establish the presence and evaluate the complexity of these traits in elephants, and in order to compare them with humans and bonobos in a more systematic way. For example, while the table supports the presence of some of the most typical morphological characteristics associated with self-domestication in elephants (e.g., smaller jaws, reduction in the number of teeth, pigmentation), there is very little research into other potential byproducts such as cranial shape change in Elephantidae.

Below, we review some of the evidence presented in Table 1 in more detail, focusing and expanding on four core features that we regard as being crucially involved in the emergence of the domesticated phenotype: i) reduced aggression, which is hypothesized to be the triggering factor for domestication (6); ii) socially sensitive cortisol levels, which are regarded as a reliable biomarker of altered stress responses and changes in the management and control of aggression; iii) extended juvenile period and enhanced play behavior, as domestication usually results in neotenic features with child-like behavior favoring many of the processes associated with social learning; and iv) sophisticated communication systems, given that the evolution of more complex communication systems has been recently identified as one of the prominent outcomes of self-domestication (13).

#### 2.1.1. Reduced aggression and increased prosociality.

First, we focus on the most prominent feature of self-domestication – reduced aggression and increased prosociality (2, 154, 162). Similar to humans and bonobos, elephants also exhibit low levels of aggression, with intraspecific and interspecific violence being relatively uncommon (90). Even during musth, which is a periodic condition in which male elephants display elevated aggression and a spike in testosterone levels, male-on-male injury and mortality are relatively rare, and males in musth even emit warning vocalizations and chemical signals that communicate their state and warn others from engaging with them (91, 163, 164). Interestingly, in several documented cases of elephants killing rhinos during musth, this kind of unusually aggressive behavior has been attributed to the lack of “mentoring” by older males and to other trauma-inducing conditions such as poaching, habitat reduction, premature weaning, witness to family deaths, etc. (163, 175). Moreover, in wild elephants, no evidence of infanticide has been found (117, 166–168). Even in potentially competitive situations, elephants are often socially tolerant or work to mitigate conflict, although this varies with rank and affiliation (99).

There is also considerable evidence for prosocial behavior in elephants. They exhibit coalition formation and shared parenting (169), offer protection and comfort to distressed conspecifics (96, 98), help dying or ill members of their herd (170), and have been documented helping individuals who do not belong to their group (95). In addition, elephants can cooperate in string-pulling tasks, showing signs of understanding their role in cooperation (97) and freely choosing partners, with elephants often working hard to mitigate competition in order to maintain cooperation (99). In addition, Asian elephants (*E. maximus*) have a millennium-long history of working with humans in logging and war, and a more recent history of working in tourism (155).

The capacity for advanced social awareness and empathy in elephants is further supported by the presence of Von Economo neurons (VEN) in elephants’ neocortex, which are rare in the neocortex of the manatee, a close relative of elephants (54, 56). These neurons are associated with “the social brain” [specifically with human-like social cognitive abilities, empathy, and self-awareness; (171)], and are suggested to reflect a specialization for the transmission of social information (53, 54). These neurons may enhance the functioning of this circuit and explain the behavioral observations of elephants helping and reassuring conspecifics. Interestingly, the distribution of VENs in elephants’ brains show a similar clustering pattern to that found in humans and bonobos, the two species hypothesized to have gone through self-domestication, with VENs being primarily found in layer 5 of the cortical regions that contain them (54, 55).

#### 2.1.2. Socially sensitive cortisol levels.

Second, elephants’ cortisol levels (which are a biomarker of reactive aggression) are sensitive to changes in the social environment, another distinctive feature of domestication. As shown for humans and bonobos (but not chimpanzees), cortisol levels in elephants are socially regulated and generally increase in stressful situations such as socioecological disturbance (79). For example, elephants show elevated cortisol levels in response to some aspects of captivity (172) and habitat reduction, as well as contact with nonkin individuals (e.g., when an unfamiliar individual is introduced into a herd) (78, 80). Related to this point, elephants appear to be highly sensitive to stress, and in response to mass deaths or social breakdown (e.g., from poaching), wild elephants can display symptoms typically associated with human posttraumatic stress disorder, including abnormal startle response, depression, unpredictable asocial behavior, hyper aggression, and reduced reproduction (110).

#### 2.1.3. Extended juvenile period and enhanced play.

Third, an extended juvenile period and enhanced play behaviors have been hypothesized to be a crucial outcome of self-domestication, contributing in turn to the behavioral changes associated with self-domestication, particularly to cultural niche construction, in a sort of positive feedback loop (144). Accordingly, a prolonged developmental window and dependence on parental care impact learning by giving rise to more learning opportunities through culture, imitation, and exposure – as opposed to innate knowledge – which in turn facilitate the acquisition of richer behaviors. Indeed, research shows that much of the wild elephant’s behavioral repertoire is socially transmitted. This includes knowledge of what to eat, how to use one’s trunk, or even how to raise offspring – an ability that is typically considered to be innate in many species (173–175). Illustrating this latter point, in the absence of exposure to an older female (e.g., when the older matriarch has been killed due to poaching, or when the elephant was raised in captivity), female elephants demonstrate poorer maternal skills and can display infant neglect (163).

Finally, enhanced playfulness in adulthood can counteract tendencies toward dominance, promoting more egalitarian and cooperative behaviors and thus contributing to the sophistication of culture (143). Crucially, the socialization patterns of elephants (and the associated changes in the social brain) parallel what we find in humans, with elephants showing increased play behavior across development. Specifically, calves play for about 5 to 20% of their active time, engaging in both social and independent play (149). Hence, young calves seek same-sex play partners outside their family, and engage in mounting, tail grasping, chasing, and wrestling. This play behavior helps young elephants explore and assess the strength of their future rivals, and also facilitates socializing and bonding with future peers (169). Besides social play, elephant calves also show nonsocial play, such as exploring and moving objects with different body parts, or showing locomotor play like floppy running, rocking, or spinning (149). Interestingly, many of these play behaviors are displayed by adult elephants as well, and seem to persist throughout their lives (e.g., casual swimming, splashing, and playing with mud) (133).

#### 2.1.4. Sophisticated communication systems.

The HSD hypothesis has been recently invoked to specifically explain the cultural evolution of language in humans, seeing as many of the biological and cognitive changes, which are at the very core of our linguistic abilities may have been brought about by self-domestication (12–14, 176, 177). That is, increased communication complexity is seen as a prominent outcome of self-domestication. In line with this idea, bonobos’ vocal repertoire was shown to be more structurally complex than that of chimpanzees (126).

Elephants display an impressive capacity for short- and long-range communication, and rely on a rich multimodal sensory system that includes vocal, visual, tactile, and chemical signals. Their acoustic communication system includes an extensive vocal repertoire (e.g., trumpets, roars, low-frequency rumbles), which encompasses specific functions and intents shared with other members of the herd (45, 128, 129, 134, 135, 138, 178). For example, elephants in Kenya have different alarm calls for humans and for bees, which elicit different responses (134, 136). Research has shown that African elephants can recognize a large network of individuals by contact vocalizations alone (130). All three species also produce calls combining several types of vocalizations in different orders, indicating potential syntax in these combination calls (179). In addition to vocalizations, elephants also use an array of visual and tactile gestures and displays, as well as intricate chemical signals. This complex system of signals mediates the intricate teamwork displayed by members of an elephant family, including day-to-day decision making about when and where to go or how to respond to predators (38). Furthermore, it shows a high degree of intraspecific variation both within and between individuals, and across different groups of elephants (180), which is also seen as an important feature of human language (181, 182).

Another relevant aspect of sophisticated communication abilities is vocal learning. Vocal learning is the ability to change one’s vocalizations based on experience, and is typically contrasted with innate calls, which are insensitive to the environment and are not learned. While humans are not the only animal capable of vocal learning (183–185), vocal learning is seen as the basis for human speech and one of its most specialized components. As such, animals capable of vocal learning are often seen as highly relevant for studying the evolution of language (186–188). Notably, the process of domestication has been directly linked to vocal learning: Domesticated animals typically display more complex vocalizations than their wild nondomesticated relatives (19, 189–191). Relevant to our hypothesis, elephants have also been recently identified as vocal learners (192–194), capable of imitating, matching, and copying artificial sounds (e.g., truck sounds), human language (e.g., Korean speech), and acoustic signals of different species.

### 2.2. Molecular Evolutionary Features of Self-Domestication in African Elephants: Genetic Evidence.

Previous studies have proposed that domestication is associated with selection on regulatory or dosage-sensitive genes, particularly, genes related to the development of the neural crest (NC), an embryonic structure giving rise to many body organs (6, 195). The presence of many features associated with the potential for self-domestication in elephants reviewed above suggests that domestication-associated genes may have also been positively selected for in the elephant lineage. To test this hypothesis, we used a dataset of 11,742 protein-coding alignments from 261 mammals (*SI Appendix, Supplemental File 13*), including the reference African elephant genome (loxAfr3), which was generated from a wild caught individual, and an adaptive branch site likelihood adaptive branch-site random effects likelihood (ABSREL) method (196) to identify positively selected genes in the elephant lineage; the ABSREL method infers the optimal number of *d_N_/d_S_* rate categories for each gene, with positive selection inferred whenever a site class is identified with *d_N_/d_S_* > 1 at *P* ≤ 0.05 (likelihood ratio test). Note that this test can only identify positive selection acting on genes, thus episodes of positive selection acting on cis-regulatory elements will not be identified but may contribute to the evolution of self-domestication phenotypes in elephants.

Next, we tested whether the 674 genes with significant evidence for positive selection in African elephants were enriched in pathways or functions of interest for our hypothesis. For this, we conducted gene ontology (GO) analyses using Panther (197, 198) and WebGestalt (199, 200) testing for enrichment in all possible terms and pathways, controlling for multiple hypothesis testing with a false discovery rate (FDR), and requiring a minimum of five genes for a term to be enriched. Seventy nine of 674 genes were annotated to pathways related to domestication. We then tested whether these 674 positively selected genes were also enriched in an a priori set of 764 candidate genes for mammal domestication (see *SI Appendix*, *Supplemental file* 1; tab “Domestication”), which were derived from merging genes that have been previously found to be positively selected in several domesticated species, including the pig (*Sus scrofa domesticus)*, lab rat (*Rattus norvegicus)*, dog (*C. lupus familiaris)*, cat (*Felis silvestris catus)*, cattle (*Bos taurus)*, horse (*Equus ferus caballus)*, rabbit (*Oryctolagus cuniculus domesticus)*, and sheep (*Ovis orientalis aries)* (3, 6, 201–215).

Because of the suggested role of the NC in the emergence of domestication traits (6), we also included a set of 89 genes that are essential for NC development and function (*SI Appendix*, *Supplemental file* 1; tab “NC”). As noted above, NC cells are a specific class of stem cells that contribute to brain and skull formation among other body structures. According to the NC hypothesis (NCH) brought forth by Wilkins et al. (6), a reduced input to these NC cells during embryonic development might account for the constellation of distinctive traits (physical, cognitive, and behavioral) found in all domesticated mammals, i.e., the so-called “domestication syndrome.” Note, however, that while the involvement of the NC in the emergence of domestication features is a promising explanation for the cooccurrence, syndrome-like presentation of selected traits in most domesticated animals, this explanation is not without concerns. In a recent paper, Wilson et al. (2021) found no clear support for this view (216), as the greater variation in domestication features associated with tissues derived from the NC, like skull shape, cannot be linked to magnitude changes in the integration among either NC or mesoderm-derived elements. Nevertheless, in order to test the reliability of the NCH with regard to elephants’ self-domestication, we included in our analysis candidates for NC development and function, which were compiled using pathogenic and functional criteria: neurocristopathy-associated genes annotated in the Online Mendelian Inheritance in Man (OMIM) database, as well as NC markers, genes that are functionally involved in NC induction and specification, genes involved in NC signaling (within NC-derived structures), and genes involved in cranial NC differentiation.

To infer the functional significance of positively selected genes, we performed two enrichment tests: 1) A hypothesis-free test, based on determining if there are biological pathways in which positively selected genes are overrepresented (enriched); and 2) A candidate gene test, based on determining if positively selected genes are overrepresented (enriched) among genes previously implicated in (self-)domestication. For the first test, positively selected genes in the African elephant were enriched in Panther pathways (hypergeometric *P* ≤ 0.05) likely related to domestication. While pathway enrichments can be biased such that well annotated genes may map to multiple pathways while little studied genes may not be annotated in any pathway, we do not expect positively selected genes to be biased toward well-studied gene sets. Thus, these kinds of ascertainment bias in gene function annotation are unlikely to bias our pathway enrichment results. Similarly, there is reduced power to detect positive selection on genes with short branch lengths but the elephant/hyrax/manatee divergence occurred ~60 Mya, which mitigates these short branch length effects.

Among the enriched pathways were those involved in socialization and the management of aggression, according to the available literature (*SI Appendix*, Supplemental File 13), as well as 5HT (serotonin) signaling, which is involved in many defensive behaviors (217). Enhanced serotonin activity in the brain, for example, inhibits predatory aggression (218), whereas exogenous serotonin administration increases harm avoidance and cooperative behavior (219, 220). Similarly, domesticated animals have higher levels of serotonin in the brain that correlate with reduced emotional reactivity and aggression (221, 222). Interestingly, bonobos exhibit increased serotonin levels and serotonin innervation in the amygdala compared to chimpanzees (68), as well as differential selection of genes involved in serotonin pathways (25). These features correlate with facial feminization and reduced cranial capacity, which are typical traits of domestication (16).

Moreover, we found enrichment in corticotropin signaling. Corticotropin (also known as adrenocorticotropic hormone, ACTH) is a component of the hypothalamic–pituitary–adrenal system, and has an important role in stress responses (223, 224). Reduced levels of ACTH have been found in domesticated animals (202), seemingly accounting for their attested reduction in aggression and anxiety toward humans. In addition, reduced ACTH levels have been hypothesized to result from changes in oxytocin levels, as this hormone inhibits ACTH and stress responses (225, 226). Interestingly, genes related to serotonin function (*HTR1F*, encoding a serotonin receptor) and oxytocin activity (*OXTR*, encoding the oxytocin receptor) have been positively selected in bonobos and early humans (17), reinforcing the view that a common physiological mechanism might account for self-domestication in different species, eventually entailing a common genetic signature too.

For the second test, genes with evidence of positive selection in elephants were not significantly enriched in either the apriori gene set of candidates for domestication (hypergeometric *P *= 0.075, FDR = 0.15) or the NC-expressed gene set (hypergeometric *P *= 0.96, FDR = 0.96). We did find 37 genes from the apriori set and two NC genes that have been positively selected for in African elephants (*SI Appendix*). Some of them stand out as potential factors accounting for selected self-domestication features in elephants. These include *SETBP1*, *CDH1,* and *NEK4. SETBP1* has mutations that result in cognitive and behavioral disturbances, including impaired communication abilities, with a variable clinical presentation. Heterozygous missense variants result in Schinzel–Giedion midface retraction syndrome (OMIM #269150), a condition involving multisystem malformations, whereas heterozygous loss-of-function variants result in *SETBP1* haploinsufficiency disorder (OMIM #616078), which is much milder, but features language problems (227, 228). Common DNA polymorphisms in the gene also result in language deficits (229), including phonological working memory dysfunction (230). High-penetrance point mutations of *SETBP1* have been found to disrupt speech development (231) and to impact negatively on speech and language abilities (232). Alterations of the gene also result in social and behavioral disturbances in humans (233). As for the other two genes, *CDH1* encodes a cadherin involved in cortical neurogenesis (234) as well as neural connectivity (235, 236), and *NEK4* regulates replicative senescence and cell cycle arrest (237), and has been associated with neuropsychiatric conditions that consist of abnormal socialization patterns as well as an altered presentation of self-domestication features like autism, schizophrenia, and bipolar disorder (238–242).

Overall, we found evidence of several genes previously implicated in domestication and social behavior having been positively selected for in elephants.

### 2.3. Potential Triggers for Elephants’ Self-Domestication.

In the previous sections, we have provided foundational evidence for our hypothesis that elephants show many of the typical behavioral, physical, and genetic markers of domestication. Since elephants have never been domesticated by humans, this implies that they have potentially undergone an organic process of self-domestication, similar to humans and bonobos, who also display domestication features without the existence of a domesticator. However, what may have triggered this process in elephants?

In humans, selection against aggression is hypothesized to have been triggered by several (potentially interacting) factors, including the reliance on more variable and nonlocal food sources that require more cooperation to obtain (20), and the adaptation to harsh environmental conditions like those resulting from the Last Glaciation, which increase the need for resource sharing (21). In bonobos, an abundance of available food resources and the lack of competition in accessing them are hypothesized to have also relieved the pressure for aggressive behaviors and promoted more prosocial behavior (16). Taken together, it seems that self-domestication can arise under two very different types of environmental conditions: lush and secure conditions that ease survival pressures and thus reduce the need for aggressive behaviors, or the exact opposite, i.e., harsh and meager conditions that increase survival pressures and require more cooperation in order to survive.

Accordingly, in the case of elephants, we propose several (and not mutually exclusive) triggers that may have promoted a selection against aggressive behaviors, which we discuss below. While some of these explanations may be more prominent or more likely than others, all of them can be seen as potential and converging drivers toward increased prosociality in elephants, which is the main selective pressure characterizing self-domestication.

#### 2.3.1. Secure environment: Lack of predators and great food availability.

As noted, the process of (self)-domestication is often linked with a kind of buffered environment, in which animals have reduced exposure to predators and enjoy a reliable and rich food supply. Such a safe and abundant environment relaxes survival pressures and stress, and has been argued to be the trigger for higher levels of prosociality in bonobos (16), as well as promote greater communicative richness and flexibility in other domesticated species such as the Bengalese finch (13, 243).

Due to their massive size and relative strength compared with most animals, elephants have very few natural predators besides humans (244), and those predators, which occasionally may include cats from the *Panthera* genus, mostly threaten calves or sick elephants (245–247). As such, elephants may be generally less worried about evading or fighting other animals for their survival. This could free cognitive resources and open up opportunities for exploration, communication, and play, which are at the very core of enhanced prosociality and self-domestication.

Moreover, elephants are more generalized eaters, and enjoy a greater availability of food. Based on changes in tooth structure in the fossil record, it appears that early Proboscideans were mostly browsers, but that there was a later shift toward a more grassy diet, with the teeth of extant species having chewing surfaces that allow them to process highly abrasive grasses (35). The three living species of elephants are generalist foragers, consuming both grasses and browse with the predominant forage varying between individuals and location (46, 248). This tooth adaptation may have allowed elephants to spend less time searching for food and more time engaging in social interactions. Similar arguments have been made for humans, with more generalized eating behaviors associated with facilitation of our unique cultural niche (249).

#### 2.3.2. Harsh environment and the rise of alloparenting.

In humans, harsh environmental conditions promoted tolerance and friendly behavior toward conspecifics, as the need to share food and other resources became beneficial for survival (21). In addition, such conditions have been claimed to promote alloparenting, i.e., the need to cooperate in order to raise offspring (250), which further promotes prosocial behaviors as less aggressive individuals are more prone to involve themselves in the upbringing of children.

While there is, to our knowledge, no evidence from the fossil record about the environmental conditions contributing to changes in the social structure of elephant species over time, variability in resource abundance across seasons and landscapes likely contributed to the evolution of fission–fusion social structure and to behavioral flexibility in elephant social behavior (251). Periodic reunification of social groups is often facilitated by more tolerance and less aggression between individuals. Indeed, in the three extant elephant species, high levels of tolerance and cooperation have been observed within and between family groups (96). This is primarily evident in female elephants who remain in their family groups with related females throughout their lives and work together to defend and care for calves (41). Allomothering is common in elephants where females other than the mother comfort, protect, and assist calves (169, 252). This cooperative care is also observed throughout the long lives of adult female elephants past their last reproduction, as they continue to assist in the survival of their kin until their death (253). It is possible that these social behaviors evolved during a period of harsh environmental conditions in the distant past within Elephantidae, but further paleontological evidence would be needed to reach any conclusions about this.

#### 2.3.3. Founder effects.

Another potential triggering factor of humans’ increased prosocial behavior and self-domestication process might be the founder effect, which is associated with the colonization of new areas as people spread across the world. In bonobos, the founder sociality hypothesis suggests that the movement of individuals from a population of the bonobo–chimpanzee common ancestor across the Congo River in Africa led to the habitation of novel environments, new group formation, and strong selection pressures for long-term changes in social structure and behavior, including increased tolerance and strong female social bonds (26).

In the recent past, there is evidence for this type of behavior change within species as well, as male elephants have acted as founders in exploiting agricultural products in human areas. In India, males have begun to form long-term and stable associations together in groups in these human-dominated landscapes (254) rather than remaining solitary or forming short-term male associations. These novel male associations are likely associated with increased tolerance as they cooperatively navigate the risky human environment. Perhaps a similar change happened at a population-level millions of years ago, leading to the nonterritorial behavior (255, 256) and prosociality of both male and female elephants observed in the three extant species.

## 3. Discussion and Conclusions

In this paper, we have presented evidence that supports the hypothesis that elephants are a self-domesticated animal, similar to humans and bonobos. This suggests that the niche that enabled elephants to evolve more complex behaviors may result from the cognitive and behavioral changes linked to a reduction in reactive aggression. Examining the behavior and genetics of elephants under this prism of self-domestication can thus provide valuable insights into this process beyond the primate order.

Future work should investigate the relevant behavioral and cognitive features outlined in Table 1 more closely to provide data in areas where they are lacking, as well as contribute quantitative data that can serve to support or refute the current hypothesis. On the genetic side, future research should examine selection for domestication genes in the Asian elephant genome, which was not tested here due to poor quality of the genetic data available, and underscore the exact role of the genes selected in African and Asian elephants that are linked to self-domestication and to prosocial behavior. Given the similarities between these species with respect to the cognitive, behavioral, and physiological traits associated with domestication (Table 1), and given our hypothesis that self-domestication is likely an old process in the elephant's lineage, we expect to find similar genetic support for self-domestication in future analyses with Asian elephants. While we argue that self- domestication happened in the elephant taxon prior to the split between the three extant species, it may be the case that the self-domestication phenotype varies between the three species due to their varied histories and environments. Although Asian elephants have never been domesticated, their close contact with people and captive breeding may have led to some changes in their behavioral phenotype that would promote less aggressive interactions with humans. This would be an interesting point for future work. Further, analyses of ancient DNA could help uncover the timeline of self-domestication in the elephant lineage by determining the presence or absence of genetic signatures for self-domestication in extinct species such as the straight-tusked elephant, mastodons, wooly mammoths, and Columbian mammoths, from which ancient genomes are now available (257, 258).

Notably, although some features of domestication such as floppy ears and curly tails are absent in elephants, domesticated species do not usually show the full suite of features associated with domestication (7). This is not only because different domesticated animals have been selected for different reasons, but mostly because blocks of features can become segregated. The reason is that domestication, similar to any developmental process, is subject to the effects of modularity (i.e., the existence of separate developmental programs for a set of features) and plasticity (i.e., the capacity to generate different phenotypes in response to changes in the environment). For instance, elephants’ big ears have already evolved under pressures to function in cooling and regulating elephants’ body temperature as well as sound localization (259, 260). Furthermore, it is important to differentiate domesticated animals, which are the outcome of only several hundreds of years of artificial and guided selection, from potentially self-domesticated animals, which are the outcome of thousands of years of true unguided evolution, as we have suggested to be the case for elephants. For this reason, we have compared the elephant phenotype with other self-domesticated species instead of with domesticated animals. While the potential differences between domestication and self-domestication features are not fully understood, they likely result in slightly different sets of genetic, physiological and cognitive traits. These potential differences that may arise due to the unique circumstances of self-induced domestication may also explain why we have found only a marginal overrepresentation of candidates for classic mammal domestication in the pool of genes that were positively selected in elephants. Nevertheless, as noted, these genes are significantly enriched in highly relevant physiological aspects and biological functions for the (self-)domestication processes, supporting our general hypothesis.

Interestingly, we found no enrichment in genes related to the NC function in elephants, although we did find intriguing positive selection in selected genes involved in NC function such as *EDNRB*, which encodes a receptor for endothelins, and which has been associated to neurocristopathies like Hirschsprung disease (OMIM#600155) (261) and Waardenburg syndrome (262). At this early stage of research, our view is that self-domestication in elephants was not primarily achieved via changes in NC input, but mostly through changes in neurotransmitter pathways associated with aggression management, and ultimately, with social behavior. This evolutionary trajectory is, in fact, better fitted to the narrative of self-domestication as opposed to guided domestication, and should be explored in future research focusing specifically on humans, bonobos, and elephants. Clearly, more research is needed on this issue, and particularly on the parallels and differences between early embryonic development in elephants, other self-domesticated species, and domesticated mammals.

Notably, the fact that self-domestication features emerge almost automatically from a reduction in reactive aggression suggests that self-domestication might be more widespread than previously thought, and it is possible that other highly social animals (e.g., dolphins, whales, parrots, Zanzibar red colobuses, and field mice) may also display the critical features associated with self-domestication and cultural niche construction. In fact, recent work has cast some doubt on whether self-domestication is even needed for explaining general features of human social evolution, seeing as these features appear to be more similar to other social mammals compared with other domesticated mammals (154). In other words, at times, it is not clear whether some of the criteria that single out self-domestication (e.g., extended juvenile period, the rareness of infanticide) cannot also be associated with increased prosociality alone. However, the fact that only one of two closely related species (e.g., bonobos vs. chimpanzees) can show markers of self-domestication while the other does not suggest that several factors might be interacting in complex ways in terms of triggering, or failing to trigger, self-domestication. For instance, if two closely related species show different ecologies, one may end up on a path of self-domestication while the other would not [as suggested for bonobos (16)], despite both species showing high prosociality. Similarly, if some factors preserve or even promote high levels of aggression in one species, this species is less likely to go through a self-domestication process. Future research should thus test for self-domestication markers in other candidate species as well and try to distinguish between different evolutionary paths toward increased prosociality (162).

Overall, we could expect the presentation of self-domestication features in different species to be quite variable, implying that self-domestication itself may be seen as more of a continuum (i.e., there may be a number of degrees of self-domestication) rather than a dichotomous trait (i.e., either a species is self-domesticated or it is not). Eventually, as in the case of the elephants’ ears discussed above, it is possible that some other adaptations might mask the effects of self-domestication in some species. As such, discovering that other animals like the elephant have also been self-domesticated would provide us with more living models for testing the effects of self-domestication on the human phenotype, as well as for understanding how the distinctive cognitive and behavioral components of the human phenotype evolved, and which of them are shared with other species.

The self-domestication hypothesis has, in part, been investigated in bonobos using experimental paradigms testing for helping and prosocial behaviors (263, 264), but results have been mixed (265). Elephants can be trained to exchange items such as tokens (or sticks) with humans for valuable rewards (e.g., ref. 266), making a direct comparison between elephants, and across ‘self-domesticated’ species like bonobos possible. Future experimental research in elephants could employ prosocial choice tasks or other helping paradigms – for a review, see ref. 265 – to investigate the variability in prosocial and altruistic tendencies within and between elephant species, and thus provide further potential cognitive evidence for their self-domestication.

If elephants have undergone self-domestication, one can expect to see at least some of human’s unique social and cognitive abilities in elephants as well, especially those associated with cultural niche construction and cultural evolution. Indeed, there is preliminary evidence in support of elephants as a relevant animal model for cultural evolution. For instance, elephants exhibit behaviors that are indicative of self-awareness and potentially the existence of ‘‘theory-of-mind,’’ including the attribution of mental or biological states to others (174), displays of empathy (96, 98), mirror self-recognition (267, 268), and sensitivity to the remains of deceased conspecifics (95, 269). In addition, elephants display tool use, which is another significant marker of cultural evolution (270, 271). Future work should carefully consider the behavior of elephants under this lens.

Our hypothesis for self-domestication in elephants thus has important implications for studying the process and outcomes of cultural evolution, which is seen as one of the most prominent and powerful hallmarks of humanity. The exciting potential of future research on elephants can also inform our understanding of the evolution of prosocial behavior across evolutionarily distant species.

## 4. Methods

*SI Appendix* include the lists of the candidate genes tested and their annotated terms (*SI Appendix*, *Supplemental file* 1); a list of Human Genome Organisation (HUGO) gene IDs for genes tested for positive selection (*SI Appendix*, *Supplemental file* 2); genes with significant evidence of positive selection (*SI Appendix*, *Supplemental file* 3); the results of the enrichment analyses, i.e., enriched Panther pathway terms (*SI Appendix*, *Supplemental file* 4), redundancy in terms reduced with affinity propagation (*SI Appendix*, *Supplemental file* 5), and weighted set cross-over (*SI Appendix*, *Supplemental file* 6); GO slim summary of biological process (*SI Appendix*, *Supplemental file* 7), cellular component (*SI Appendix*, *Supplemental file* 8), and molecular function (*SI Appendix*, *Supplemental file* 9); category terms for the user uploaded gene IDs and positively selected gene annotations (*SI Appendix*, *Supplemental file* 10); and genes without unique IDs (*SI Appendix*, *Supplemental file* 11). Finally, we also include an interactive .html file showing all the data (*SI Appendix*, *Supplemental file* 12), consisting of the enriched Panther terms, the enrichment *P*-values (hypergeometric test), *Q*-values (FDR) for the test, the Panther term set size, and the enrichment ratio.

## Supplementary Material

Appendix 01 (PDF)

## Data Availability

.html data have been deposited in Open Science Framework (OSF) (https://osf.io/download/nfvmx/). All other data are included in the article and/or *SI Appendix*.
