# Supplementary material for: Elephants as an animal model for self-domestication
Source: Proc Natl Acad Sci U S A. 2023 Apr 3;120(15):e2208607120. doi: 10.1073/pnas.2208607120 (PMC10104499; doi:10.1073/pnas.2208607120)
Supplement: Supplementary file 1 — Appendix 01 (PDF) [file pnas.2208607120.sapp.pdf]

Supporting Information for

Elephants as an animal model for self-domestication.

Limor Raviv
Sarah L. Jacobson
Joshua M. Plotnik
Jacob Bowman
Vincent Lynch
Antonio Benítez-Burraco

Corresponding author: Limor Raviv
Email: limor.raviv@mail.huji.ac.il

This PDF file includes:

1. Supplemental File 1a - Raviv et al (2023)
2. Supplemental File 1b - Raviv et al (2023)
3. Supplemental File 2 - Raviv et al (2023)
4. Supplemental File 3 - Raviv et al (2023)
5. Supplemental File 4 - Raviv et al (2023)
6. Supplemental File 5 - Raviv et al (2023)
7. Supplemental File 6 - Raviv et al (2023)
8. Supplemental File 7 - Raviv et al (2023)
9. Supplemental File 8 - Raviv et al (2023)
10. Supplemental File 9 - Raviv et al (2023)
11. Supplemental File 10 - Raviv et al (2023)
12. Supplemental File 11 - Raviv et al (2023)
13. Supplemental File 12 - Raviv et al (2023)
14. Figure S1 (Supplemental File 13 - Raviv et al (2023))

Supplemental File 1a: List of candidate Domestication genes and their annotated terms (Benítez-Burraco et al., 2017 + Theofanopoulou et al., 2017)

Gene abbreviation	Gene name	NCBI link
ABAT	4-aminobutyrate aminotransferase	https://www.ncbi.nlm.nih.gov/gene/?term=ABAT
ABCA10	ATP binding cassette subfamily A member 10	https://www.ncbi.nlm.nih.gov/gene/?term=ABCA10
ABCA5	ATP binding cassette subfamily A member 5	https://www.ncbi.nlm.nih.gov/gene/?term=ABCA5
ABCB10	ATP binding cassette subfamily B member 10	https://www.ncbi.nlm.nih.gov/gene/?term=ABCB10
ABCG1	ATP binding cassette subfamily G member 1	https://www.ncbi.nlm.nih.gov/gene/?term=ABCG1
ABCG2	ATP binding cassette subfamily G member 2 (Junior blood group)	https://www.ncbi.nlm.nih.gov/gene/?term=ABCG2
ABHD1	abhydrolase domain containing 1	https://www.ncbi.nlm.nih.gov/gene/?term=ABHD1
ACA64	small nucleolar RNA, H/ACA box 78	https://www.ncbi.nlm.nih.gov/gene/?term=ACA64
ACAD8	acyl-CoA dehydrogenase family member 8	https://www.ncbi.nlm.nih.gov/gene/?term=ACAD8
ACMSD	aminocarboxymuconate semialdehyde decarboxylase	https://www.ncbi.nlm.nih.gov/gene/?term=ACMSD
ACOT11	acyl-CoA thioesterase 11	https://www.ncbi.nlm.nih.gov/gene/?term=ACOT11
ACOT13	acyl-CoA thioesterase 13	https://www.ncbi.nlm.nih.gov/gene/?term=ACOT13
ACOT8	acyl-CoA thioesterase 8	https://www.ncbi.nlm.nih.gov/gene/?term=ACOT8
ACOX2	acyl-CoA oxidase 2	https://www.ncbi.nlm.nih.gov/gene/?term=ACOX2
ACOX3	acyl-CoA oxidase 3, pristanoyl	https://www.ncbi.nlm.nih.gov/gene/?term=ACOX3
ACSF3	acyl-CoA synthetase family member 3	https://www.ncbi.nlm.nih.gov/gene/?term=ACSF3
ACSM5	acyl-CoA synthetase medium chain family member 5	https://www.ncbi.nlm.nih.gov/gene/?term=ACSM5
ACSS2	acyl-CoA synthetase short chain family member 2	https://www.ncbi.nlm.nih.gov/gene/?term=ACSS2
ACTA1	actin alpha 1, skeletal muscle	https://www.ncbi.nlm.nih.gov/gene/?term=ACTA1
ADAM2	ADAM metallopeptidase domain 2	https://www.ncbi.nlm.nih.gov/gene/?term=ADAM2
ADAMDEC1	ADAM like decysin 1	https://www.ncbi.nlm.nih.gov/gene/?term=ADAMDEC1
ADAMTS13	ADAM metallopeptidase with thrombospondin type 1 motif 13	https://www.ncbi.nlm.nih.gov/gene/?term=ADAMTS13
ADAMTSL3	ADAMTS like 3	https://www.ncbi.nlm.nih.gov/gene/?term=ADAMTSL3
ADRB2	adrenoceptor beta 2	https://www.ncbi.nlm.nih.gov/gene/?term=ADRB2

AHCY	adenosylhomocysteinase	https://www.ncbi.nlm.nih.gov/gene/?term=AHCY
AHCYL2	adenosylhomocysteinase like 2	https://www.ncbi.nlm.nih.gov/gene/?term=AHCYL2
AK1	adenylate kinase 1	https://www.ncbi.nlm.nih.gov/gene/?term=AK1
AKAP1	A-kinase anchoring protein 1	https://www.ncbi.nlm.nih.gov/gene/?term=AKAP1
ALDH16A1	aldehyde dehydrogenase 16 family member A1	https://www.ncbi.nlm.nih.gov/gene/?term=ALDH16A1
ALDH18A1	aldehyde dehydrogenase 18 family member A1	https://www.ncbi.nlm.nih.gov/gene/?term=ALDH18A1
ALDH1L2	aldehyde dehydrogenase 1 family member L2	https://www.ncbi.nlm.nih.gov/gene/?term=ALDH1L2
ALK	ALK receptor tyrosine kinase	https://www.ncbi.nlm.nih.gov/gene/?term=ALK
ALS2CR12	flagellum associated containing coiled-coil domains 1	https://www.ncbi.nlm.nih.gov/gene/?term=ALS2CR12
AMACR	alpha-methylacyl-CoA racemase	https://www.ncbi.nlm.nih.gov/gene/?term=AMACR
AMBRA1	autophagy and beclin 1 regulator 1	https://www.ncbi.nlm.nih.gov/gene/?term=AMBRA1
ANK1	ankyrin 1	https://www.ncbi.nlm.nih.gov/gene/?term=ANK1
ANKDD1A	ankyrin repeat and death domain containing 1A	https://www.ncbi.nlm.nih.gov/gene/?term=ANKDD1A
ANKRD2	ankyrin repeat domain 2	https://www.ncbi.nlm.nih.gov/gene/?term=ANKRD2
ANKRD49	ankyrin repeat domain 49	https://www.ncbi.nlm.nih.gov/gene/?term=ANKRD49
ANKRD50	ankyrin repeat domain 50	https://www.ncbi.nlm.nih.gov/gene/?term=ANKRD50
ANKS4B	ankyrin repeat and sterile alpha motif domain containing 4B	https://www.ncbi.nlm.nih.gov/gene/?term=ANKS4B
APEH	acylaminoacyl-peptide hydrolase	https://www.ncbi.nlm.nih.gov/gene/?term=APEH
APOBEC4	apolipoprotein B mRNA editing enzyme catalytic polypeptide like 4	https://www.ncbi.nlm.nih.gov/gene/?term=APOBEC4
APOPT1	cytochrome c oxidase assembly factor 8	https://www.ncbi.nlm.nih.gov/gene/?term=APOPT1
ARHGAP26	Rho GTPase activating protein 26	https://www.ncbi.nlm.nih.gov/gene/?term=ARHGAP26
ARID1B	AT-rich interaction domain 1B	https://www.ncbi.nlm.nih.gov/gene/?term=ARID1B
ARID3B	AT-rich interaction domain 3B	https://www.ncbi.nlm.nih.gov/gene/?term=ARID3B
ARL6IP1	ADP ribosylation factor like GTPase 6 interacting protein 1	https://www.ncbi.nlm.nih.gov/gene/?term=ARL6IP1
ARL9	ADP ribosylation factor like GTPase 9	https://www.ncbi.nlm.nih.gov/gene/?term=ARL9
ART3	ADP-ribosyltransferase 3	https://www.ncbi.nlm.nih.gov/gene/?term=ART3
ASAP1	ArfGAP with SH3 domain, ankyrin repeat and PH domain 1	https://www.ncbi.nlm.nih.gov/gene/?term=ASAP1

ASB11	ankyrin repeat and SOCS box containing 11	https://www.ncbi.nlm.nih.gov/gene/?term=ASB11
ASIP	agouti signaling protein	https://www.ncbi.nlm.nih.gov/gene/?term=ASIP
ASTN1	astrotactin 1	https://www.ncbi.nlm.nih.gov/gene/?term=ASTN1
ASTN2	astrotactin 2	https://www.ncbi.nlm.nih.gov/gene/?term=ASTN2
ATL1	atlastin GTPase 1	https://www.ncbi.nlm.nih.gov/gene/?term=ATL1
ATXN7L1	ataxin 7 like 1	https://www.ncbi.nlm.nih.gov/gene/?term=ATXN7L1
B3GALTL	beta 3-glucosyltransferase	https://www.ncbi.nlm.nih.gov/gene/?term=B3GALTL
B3GLCT	beta 3-glucosyltransferase	https://www.ncbi.nlm.nih.gov/gene/?term=B3GLCT
BAG5	BAG cochaperone 5	https://www.ncbi.nlm.nih.gov/gene/?term=BAG5
BARD1	BRCA1 associated RING domain 1	https://www.ncbi.nlm.nih.gov/gene/?term=BARD1
BAZ1B	bromodomain adjacent to zinc finger domain 1B	https://www.ncbi.nlm.nih.gov/gene/?term=BAZ1B
BCAP31	B cell receptor associated protein 31	https://www.ncbi.nlm.nih.gov/gene/?term=BCAP31
BMP15	bone morphogenetic protein 15	https://www.ncbi.nlm.nih.gov/gene/?term=BMP15
BMPR1B	bone morphogenetic protein receptor type 1B	https://www.ncbi.nlm.nih.gov/gene/?term=BMPR1B
BPI	bactericidal permeability increasing protein	https://www.ncbi.nlm.nih.gov/gene/?term=BPI
BRAF	B-Raf proto-oncogene, serine/threonine kinase	https://www.ncbi.nlm.nih.gov/gene/?term=BRAF
BRCA1	BRCA1 DNA repair associated	https://www.ncbi.nlm.nih.gov/gene/?term=BRCA1
BTAF1	B-TFIID TATA-box binding protein associated factor 1	https://www.ncbi.nlm.nih.gov/gene/?term=BTAF1
C11orf54	chromosome 11 open reading frame 54	https://www.ncbi.nlm.nih.gov/gene/?term=C11orf54
C11orf63	junctional cadherin complex regulator	https://www.ncbi.nlm.nih.gov/gene/?term=C11orf63
C15orf60	REC114 meiotic recombination protein	https://www.ncbi.nlm.nih.gov/gene/?term=C15orf60
C16orf71	chromosome 16 open reading frame 71	https://www.ncbi.nlm.nih.gov/gene/?term=C16orf71
C17orf67	chromosome 17 open reading frame 67	https://www.ncbi.nlm.nih.gov/gene/?term=C17orf67
C1orf109	chromosome 1 open reading frame 109	https://www.ncbi.nlm.nih.gov/gene/?term=C1orf109
C22orf31	chromosome 22 open reading frame 31	https://www.ncbi.nlm.nih.gov/gene/?term=C22orf31
C2orf40	ECRG4 augurin precursor	https://www.ncbi.nlm.nih.gov/gene/?term=C2orf40
C2orf62	ciliogenesis associated TTC17 interacting protein	https://www.ncbi.nlm.nih.gov/gene/?term=C2orf62

C3orf62	chromosome 3 open reading frame 62	https://www.ncbi.nlm.nih.gov/gene/?term=C3orf62
C4orf33	chromosome 4 open reading frame 33	https://www.ncbi.nlm.nih.gov/gene/?term=C4orf33
C5orf15	chromosome 5 open reading frame 15	https://www.ncbi.nlm.nih.gov/gene/?term=C5orf15
C7orf72	spermatogenesis associated 48	https://www.ncbi.nlm.nih.gov/gene/?term=C7orf72
C8B	complement C8 beta chain	https://www.ncbi.nlm.nih.gov/gene/?term=C8B
C9orf89	caspase recruitment domain family member 19	https://www.ncbi.nlm.nih.gov/gene/?term=C9orf89
C9orf96	serine/threonine kinase like domain containing 1	https://www.ncbi.nlm.nih.gov/gene/?term=C9orf96
CACNA1C	calcium voltage-gated channel subunit alpha1 C	https://www.ncbi.nlm.nih.gov/gene/?term=CACNA1C
CACNA1D	calcium voltage-gated channel subunit alpha1 D	https://www.ncbi.nlm.nih.gov/gene/?term=CACNA1D
CADM2	cell adhesion molecule 2	https://www.ncbi.nlm.nih.gov/gene/?term=CADM2
CAGE1	cancer antigen 1	https://www.ncbi.nlm.nih.gov/gene/?term=CAGE1
CALCB	calcitonin related polypeptide beta	https://www.ncbi.nlm.nih.gov/gene/?term=CALCB
CASP7	caspase 7	https://www.ncbi.nlm.nih.gov/gene/?term=CASP7
CAST	calpastatin	https://www.ncbi.nlm.nih.gov/gene/?term=CAST
CAV1	caveolin 1	https://www.ncbi.nlm.nih.gov/gene/?term=CAV1
CAV2	caveolin 2	https://www.ncbi.nlm.nih.gov/gene/?term=CAV2
CBD118	defensin beta 118	https://www.ncbi.nlm.nih.gov/gene/?term=CBD118
CBD121	defensin beta 121	https://www.ncbi.nlm.nih.gov/gene/?term=CBD121
CBD122	defensin beta 122	https://www.ncbi.nlm.nih.gov/gene/?term=CBD122
CBX2	chromobox 2	https://www.ncbi.nlm.nih.gov/gene/?term=CBX2
CCDC38	coiled-coil domain containing 38	https://www.ncbi.nlm.nih.gov/gene/?term=CCDC38
CCDC64B	BICD family like cargo adaptor 2	https://www.ncbi.nlm.nih.gov/gene/?term=CCDC64B
CCDC67	deuterosome assembly protein 1	https://www.ncbi.nlm.nih.gov/gene/?term=CCDC67
CCDC70	coiled-coil domain containing 70	https://www.ncbi.nlm.nih.gov/gene/?term=CCDC70
CCDC82	coiled-coil domain containing 82	https://www.ncbi.nlm.nih.gov/gene/?term=CCDC82
CCNJ	cyclin J	https://www.ncbi.nlm.nih.gov/gene/?term=CCNJ
CCNT2	cyclin T2	https://www.ncbi.nlm.nih.gov/gene/?term=CCNT2

CD27	CD27 molecule	https://www.ncbi.nlm.nih.gov/gene/?term=CD27
CD36	CD36 molecule	https://www.ncbi.nlm.nih.gov/gene/?term=CD36
CD48	CD48 molecule	https://www.ncbi.nlm.nih.gov/gene/?term=CD48
CD93	CD93 molecule	https://www.ncbi.nlm.nih.gov/gene/?term=CD93
CDH1	cadherin 1	https://www.ncbi.nlm.nih.gov/gene/?term=CDH1
CDH6	cadherin 6	https://www.ncbi.nlm.nih.gov/gene/?term=CDH6
CDK5RAP1	CDK5 regulatory subunit associated protein 1	https://www.ncbi.nlm.nih.gov/gene/?term=CDK5RAP1
CDKL3	cyclin dependent kinase like 3	https://www.ncbi.nlm.nih.gov/gene/?term=CDKL3
CDRT1	CMT1A duplicated region transcript 1	https://www.ncbi.nlm.nih.gov/gene/?term=CDRT1
CDRT4	CMT1A duplicated region transcript 4	https://www.ncbi.nlm.nih.gov/gene/?term=CDRT4
CELA1	chymotrypsin like elastase 1	https://www.ncbi.nlm.nih.gov/gene/?term=CELA1
CENPE	centromere protein E	https://www.ncbi.nlm.nih.gov/gene/?term=CENPE
CENPM	centromere protein M	https://www.ncbi.nlm.nih.gov/gene/?term=CENPM
CEP68	centrosomal protein 68	https://www.ncbi.nlm.nih.gov/gene/?term=CEP68
CEP97	centrosomal protein 97	https://www.ncbi.nlm.nih.gov/gene/?term=CEP97
CERS3	ceramide synthase 3	https://www.ncbi.nlm.nih.gov/gene/?term=CERS3
CETN3	centrin 3	https://www.ncbi.nlm.nih.gov/gene/?term=CETN3
CHD7	chromodomain helicase DNA binding protein 7	https://www.ncbi.nlm.nih.gov/gene/?term=CHD7
CHMP4B	charged multivesicular body protein 4B	https://www.ncbi.nlm.nih.gov/gene/?term=CHMP4B
CIB4	calcium and integrin binding family member 4	https://www.ncbi.nlm.nih.gov/gene/?term=CIB4
CKB	creatine kinase B	https://www.ncbi.nlm.nih.gov/gene/?term=CKB
CKM	creatine kinase, M-type	https://www.ncbi.nlm.nih.gov/gene/?term=CKM
CLCA3	chloride channel accessory 3, pseudogene	https://www.ncbi.nlm.nih.gov/gene/?term=CLCA3
CLDN17	claudin 17	https://www.ncbi.nlm.nih.gov/gene/?term=CLDN17
CLEC5A	C-type lectin domain containing 5A	https://www.ncbi.nlm.nih.gov/gene/?term=CLEC5A
CLK3	CDC like kinase 3	https://www.ncbi.nlm.nih.gov/gene/?term=CLK3
CNGA2	cyclic nucleotide gated channel subunit alpha 2	https://www.ncbi.nlm.nih.gov/gene/?term=CNGA2

CNTN6	contactin 6	https://www.ncbi.nlm.nih.gov/gene/?term=CNTN6
COA5	cytochrome c oxidase assembly factor 5	https://www.ncbi.nlm.nih.gov/gene/?term=COA5
COBL	cordon-bleu WH2 repeat protein	https://www.ncbi.nlm.nih.gov/gene/?term=COBL
COG6	component of oligomeric golgi complex 6	https://www.ncbi.nlm.nih.gov/gene/?term=COG6
COIL	coilin	https://www.ncbi.nlm.nih.gov/gene/?term=COIL
COL11A1	collagen type XI alpha 1 chain	https://www.ncbi.nlm.nih.gov/gene/?term=COL11A1
COL14A1	collagen type XIV alpha 1 chain	https://www.ncbi.nlm.nih.gov/gene/?term=COL14A1
COL22A1	collagen type XXII alpha 1 chain	https://www.ncbi.nlm.nih.gov/gene/?term=COL22A1
COL6A3	collagen type VI alpha 3 chain	https://www.ncbi.nlm.nih.gov/gene/?term=COL6A3
COL9A3	collagen type IX alpha 3 chain	https://www.ncbi.nlm.nih.gov/gene/?term=COL9A3
COMMD1	copper metabolism domain containing 1	https://www.ncbi.nlm.nih.gov/gene/?term=COMMD1
COQ10B	coenzyme Q10B	https://www.ncbi.nlm.nih.gov/gene/?term=COQ10B
COX4I1	cytochrome c oxidase subunit 4I1	https://www.ncbi.nlm.nih.gov/gene/?term=COX4I1
COX4I2	cytochrome c oxidase subunit 4I2	https://www.ncbi.nlm.nih.gov/gene/?term=COX4I2
CPEB3	cytoplasmic polyadenylation element binding protein 3	https://www.ncbi.nlm.nih.gov/gene/?term=CPEB3
CRH	corticotropin releasing hormone	https://www.ncbi.nlm.nih.gov/gene/?term=CRH
CROCC	ciliary rootlet coiled-coil, rootletin	https://www.ncbi.nlm.nih.gov/gene/?term=CROCC
CRTC3	CREB regulated transcription coactivator 3	https://www.ncbi.nlm.nih.gov/gene/?term=CRTC3
CRYM	crystallin mu	https://www.ncbi.nlm.nih.gov/gene/?term=CRYM
C-SKI	SKI proto-oncogene	https://www.ncbi.nlm.nih.gov/gene/?term=C-SKI
CSPP1	centrosome and spindle pole associated protein 1	https://www.ncbi.nlm.nih.gov/gene/?term=CSPP1
CTTN	cortactin	https://www.ncbi.nlm.nih.gov/gene/?term=CTTN
CUL1	cullin 1	https://www.ncbi.nlm.nih.gov/gene/?term=CUL1
CUX2	cut like homeobox 2	https://www.ncbi.nlm.nih.gov/gene/?term=CUX2
CXCL10	C-X-C motif chemokine ligand 10	https://www.ncbi.nlm.nih.gov/gene/?term=CXCL10
CYB5R1	cytochrome b5 reductase 1	https://www.ncbi.nlm.nih.gov/gene/?term=CYB5R1
CYFIP1	cytoplasmic FMR1 interacting protein 1	https://www.ncbi.nlm.nih.gov/gene/?term=CYFIP1

CYP1A1	cytochrome P450 family 1 subfamily A member 1	https://www.ncbi.nlm.nih.gov/gene/?term=CYP1A1
CYP1A2	cytochrome P450 family 1 subfamily A member 2	https://www.ncbi.nlm.nih.gov/gene/?term=CYP1A2
CYP26A1	cytochrome P450 family 26 subfamily A member 1	https://www.ncbi.nlm.nih.gov/gene/?term=CYP26A1
CYP26C1	cytochrome P450 family 26 subfamily C member 1	https://www.ncbi.nlm.nih.gov/gene/?term=CYP26C1
CYP27B1	cytochrome P450 family 27 subfamily B member 1	https://www.ncbi.nlm.nih.gov/gene/?term=CYP27B1
DACT1	dishevelled binding antagonist of beta catenin 1	https://www.ncbi.nlm.nih.gov/gene/?term=DACT1
DAPK1	death associated protein kinase 1	https://www.ncbi.nlm.nih.gov/gene/?term=DAPK1
DBI	diazepam binding inhibitor, acyl-CoA binding protein	https://www.ncbi.nlm.nih.gov/gene/?term=DBI
DCC	DCC netrin 1 receptor	https://www.ncbi.nlm.nih.gov/gene/?term=DCC
DCST1	DC-STAMP domain containing 1	https://www.ncbi.nlm.nih.gov/gene/?term=DCST1
DDC	dopa decarboxylase	https://www.ncbi.nlm.nih.gov/gene/?term=DDC
DEFB103B	defensin beta 103B	https://www.ncbi.nlm.nih.gov/gene/?term=DEFB103B
DEFB119	defensin beta 119	https://www.ncbi.nlm.nih.gov/gene/?term=DEFB119
DEFB122	defensin beta 122 (pseudogene)	https://www.ncbi.nlm.nih.gov/gene/?term=DEFB122
DGAT1	diacylglycerol O-acyltransferase 1	https://www.ncbi.nlm.nih.gov/gene/?term=DGAT1
DHDH	dihydrodiol dehydrogenase	https://www.ncbi.nlm.nih.gov/gene/?term=DHDH
DLGAP1	DLG associated protein 1	https://www.ncbi.nlm.nih.gov/gene/?term=DLGAP1
DLL3	delta like canonical Notch ligand 3	https://www.ncbi.nlm.nih.gov/gene/?term=DLL3
DMRT3	doublesex and mab-3 related transcription factor 3	https://www.ncbi.nlm.nih.gov/gene/?term=DMRT3
DNAH3	dynein axonemal heavy chain 3	https://www.ncbi.nlm.nih.gov/gene/?term=DNAH3
DNAH9	dynein axonemal heavy chain 9	https://www.ncbi.nlm.nih.gov/gene/?term=DNAH9
DNAJA1	DnaJ heat shock protein family (Hsp40) member A1	https://www.ncbi.nlm.nih.gov/gene/?term=DNAJA1
DNAJB9	DnaJ heat shock protein family (Hsp40) member B9	https://www.ncbi.nlm.nih.gov/gene/?term=DNAJB9
DNTTIP2	deoxynucleotidyltransferase terminal interacting protein 2	https://www.ncbi.nlm.nih.gov/gene/?term=DNTTIP2
DOCK2	dedicator of cytokinesis 2	https://www.ncbi.nlm.nih.gov/gene/?term=DOCK2
DPEP3	dipeptidase 3	https://www.ncbi.nlm.nih.gov/gene/?term=DPEP3
DSCAM	DS cell adhesion molecule	https://www.ncbi.nlm.nih.gov/gene/?term=DSCAM

DTD1	D-aminoacyl-tRNA deacylase 1	https://www.ncbi.nlm.nih.gov/gene/?term=DTD1
DUSP19	dual specificity phosphatase 19	https://www.ncbi.nlm.nih.gov/gene/?term=DUSP19
ECHDC1	ethylmalonyl-CoA decarboxylase 1	https://www.ncbi.nlm.nih.gov/gene/?term=ECHDC1
EDC3	enhancer of mRNA decapping 3	https://www.ncbi.nlm.nih.gov/gene/?term=EDC3
EDN3	endothelin 3	https://www.ncbi.nlm.nih.gov/gene/?term=EDN3
EDNRB	endothelin receptor type B	https://www.ncbi.nlm.nih.gov/gene/?term=EDNRB
EEA1	early endosome antigen 1	https://www.ncbi.nlm.nih.gov/gene/?term=EEA1
EHBP1L1	EH domain binding protein 1 like 1	https://www.ncbi.nlm.nih.gov/gene/?term=EHBP1L1
EIF2S2	eukaryotic translation initiation factor 2 subunit beta	https://www.ncbi.nlm.nih.gov/gene/?term=EIF2S2
ELF2	E74 like ETS transcription factor 2	https://www.ncbi.nlm.nih.gov/gene/?term=ELF2
EMC2	ER membrane protein complex subunit 2	https://www.ncbi.nlm.nih.gov/gene/?term=EMC2
ENKUR	enkurin, TRPC channel interacting protein	https://www.ncbi.nlm.nih.gov/gene/?term=ENKUR
ENTPD1	ectonucleoside triphosphate diphosphohydrolase 1	https://www.ncbi.nlm.nih.gov/gene/?term=ENTPD1
ENTPD7	ectonucleoside triphosphate diphosphohydrolase 7	https://www.ncbi.nlm.nih.gov/gene/?term=ENTPD7
EPHB4	EPH receptor B4	https://www.ncbi.nlm.nih.gov/gene/?term=EPHB4
EPS15	epidermal growth factor receptor pathway substrate 15	https://www.ncbi.nlm.nih.gov/gene/?term=EPS15
ERBB4	erb-b2 receptor tyrosine kinase 4	https://www.ncbi.nlm.nih.gov/gene/?term=ERBB4
ETNPPL	ethanolamine-phosphate phospho-lyase	https://www.ncbi.nlm.nih.gov/gene/?term=ETNPPL
ETV4	ETS variant transcription factor 4	https://www.ncbi.nlm.nih.gov/gene/?term=ETV4
EVC2	EvC ciliary complex subunit 2	https://www.ncbi.nlm.nih.gov/gene/?term=EVC2
EYA1	EYA transcriptional coactivator and phosphatase 1	https://www.ncbi.nlm.nih.gov/gene/?term=EYA1
F9	coagulation factor IX	https://www.ncbi.nlm.nih.gov/gene/?term=F9
FABP5	fatty acid binding protein 5	https://www.ncbi.nlm.nih.gov/gene/?term=FABP5
FAF1	Fas associated factor 1	https://www.ncbi.nlm.nih.gov/gene/?term=FAF1
FAIM3	Fc fragment of IgM receptor	https://www.ncbi.nlm.nih.gov/gene/?term=FAIM3
FAM107B	family with sequence similarity 107 member B	https://www.ncbi.nlm.nih.gov/gene/?term=FAM107B
FAM114A2	family with sequence similarity 114 member A2	https://www.ncbi.nlm.nih.gov/gene/?term=FAM114A2

FAM131B	family with sequence similarity 131 member B	https://www.ncbi.nlm.nih.gov/gene/?term=FAM131B
FAM172A	family with sequence similarity 172 member A	https://www.ncbi.nlm.nih.gov/gene/?term=FAM172A
FAM179A	TOG array regulator of axonemal microtubules 2	https://www.ncbi.nlm.nih.gov/gene/?term=FAM179A
FAM40B	striatin interacting protein 2	https://www.ncbi.nlm.nih.gov/gene/?term=FAM40B
FAM69A	divergent protein kinase domain 1A	https://www.ncbi.nlm.nih.gov/gene/?term=FAM69A
FANCA	FA complementation group A	https://www.ncbi.nlm.nih.gov/gene/?term=FANCA
FANCB	FA complementation group B	https://www.ncbi.nlm.nih.gov/gene/?term=FANCB
FAT4	FAT atypical cadherin 4	https://www.ncbi.nlm.nih.gov/gene/?term=FAT4
FBN3	fibrillin 3	https://www.ncbi.nlm.nih.gov/gene/?term=FBN3
FBXL22	F-box and leucine rich repeat protein 22	https://www.ncbi.nlm.nih.gov/gene/?term=FBXL22
FBXO10	F-box protein 10	https://www.ncbi.nlm.nih.gov/gene/?term=FBXO10
FBXO28	F-box protein 28	https://www.ncbi.nlm.nih.gov/gene/?term=FBXO28
FBXO31	F-box protein 31	https://www.ncbi.nlm.nih.gov/gene/?term=FBXO31
FBXW10	F-box and WD repeat domain containing 10	https://www.ncbi.nlm.nih.gov/gene/?term=FBXW10
FBXW11	F-box and WD repeat domain containing 11	https://www.ncbi.nlm.nih.gov/gene/?term=FBXW11
FCHSD2	FCH and double SH3 domains 2	https://www.ncbi.nlm.nih.gov/gene/?term=FCHSD2
FCRL4	Fc receptor like 4	https://www.ncbi.nlm.nih.gov/gene/?term=FCRL4
FER	FER tyrosine kinase	https://www.ncbi.nlm.nih.gov/gene/?term=FER
FGA	fibrinogen alpha chain	https://www.ncbi.nlm.nih.gov/gene/?term=FGA
FGD6	FYVE, RhoGEF and PH domain containing 6	https://www.ncbi.nlm.nih.gov/gene/?term=FGD6
FGF13	fibroblast growth factor 13	https://www.ncbi.nlm.nih.gov/gene/?term=FGF13
FGF18	fibroblast growth factor 18	https://www.ncbi.nlm.nih.gov/gene/?term=FGF18
FGF4	fibroblast growth factor 4	https://www.ncbi.nlm.nih.gov/gene/?term=FGF4
FGF5	fibroblast growth factor 5	https://www.ncbi.nlm.nih.gov/gene/?term=FGF5
FGF8	fibroblast growth factor 8	https://www.ncbi.nlm.nih.gov/gene/?term=FGF8
FGFBP3	fibroblast growth factor binding protein 3	https://www.ncbi.nlm.nih.gov/gene/?term=FGFBP3
FHL1	four and a half LIM domains 1	https://www.ncbi.nlm.nih.gov/gene/?term=FHL1

FMO3	flavin containing dimethylaniline monoxygenase 3	https://www.ncbi.nlm.nih.gov/gene/?term=FMO3
FN3K	fructosamine 3 kinase	https://www.ncbi.nlm.nih.gov/gene/?term=FN3K
FOXD3	forkhead box D3	https://www.ncbi.nlm.nih.gov/gene/?term=FOXD3
FOXI1	forkhead box I1	https://www.ncbi.nlm.nih.gov/gene/?term=FOXI1
FOXJ3	forkhead box J3	https://www.ncbi.nlm.nih.gov/gene/?term=FOXJ3
FRMD6	FERM domain containing 6	https://www.ncbi.nlm.nih.gov/gene/?term=FRMD6
FRMD7	FERM domain containing 7	https://www.ncbi.nlm.nih.gov/gene/?term=FRMD7
FRMPD1	FERM and PDZ domain containing 1	https://www.ncbi.nlm.nih.gov/gene/?term=FRMPD1
FSTL4	follistatin like 4	https://www.ncbi.nlm.nih.gov/gene/?term=FSTL4
GABRA5	gamma-aminobutyric acid type A receptor subunit alpha5	https://www.ncbi.nlm.nih.gov/gene/?term=GABRA5
GAK	cyclin G associated kinase	https://www.ncbi.nlm.nih.gov/gene/?term=GAK
GALR1	galanin receptor 1	https://www.ncbi.nlm.nih.gov/gene/?term=GALR1
GAPDHS	glyceraldehyde-3-phosphate dehydrogenase, spermatogenic	https://www.ncbi.nlm.nih.gov/gene/?term=GAPDHS
GCNT7	glucosaminyl (N-acetyl) transferase family member 7	https://www.ncbi.nlm.nih.gov/gene/?term=GCNT7
GDNF	glial cell derived neurotrophic factor	https://www.ncbi.nlm.nih.gov/gene/?term=GDNF
GEMIN7	gem nuclear organelle associated protein 7	https://www.ncbi.nlm.nih.gov/gene/?term=GEMIN7
GGT6	gamma-glutamyltransferase 6	https://www.ncbi.nlm.nih.gov/gene/?term=GGT6
GGT7	gamma-glutamyltransferase 7	https://www.ncbi.nlm.nih.gov/gene/?term=GGT7
GLRA1	glycine receptor alpha 1	https://www.ncbi.nlm.nih.gov/gene/?term=GLRA1
GNAT3	G protein subunit alpha transducin 3	https://www.ncbi.nlm.nih.gov/gene/?term=GNAT3
GNG10	G protein subunit gamma 10	https://www.ncbi.nlm.nih.gov/gene/?term=GNG10
GNG4	G protein subunit gamma 4	https://www.ncbi.nlm.nih.gov/gene/?term=GNG4
GNPTAB	N-acetylglucosamine-1-phosphate transferase subunits alpha and beta	https://www.ncbi.nlm.nih.gov/gene/?term=GNPTAB
GOLGA1	golgin A1	https://www.ncbi.nlm.nih.gov/gene/?term=GOLGA1
GP2	glycoprotein 2	https://www.ncbi.nlm.nih.gov/gene/?term=GP2
GPATCH8	G-patch domain containing 8	https://www.ncbi.nlm.nih.gov/gene/?term=GPATCH8
GPR133	adhesion G protein-coupled receptor D1	https://www.ncbi.nlm.nih.gov/gene/?term=GPR133

GPR139	G protein-coupled receptor 139	https://www.ncbi.nlm.nih.gov/gene/?term=GPR139
GPR15	G protein-coupled receptor 15	https://www.ncbi.nlm.nih.gov/gene/?term=GPR15
GPR174	G protein-coupled receptor 174	https://www.ncbi.nlm.nih.gov/gene/?term=GPR174
GPRASP2	G protein-coupled receptor associated sorting protein 2	https://www.ncbi.nlm.nih.gov/gene/?term=GPRASP2
GPRC5A	G protein-coupled receptor class C group 5 member A	https://www.ncbi.nlm.nih.gov/gene/?term=GPRC5A
GPRC5B	G protein-coupled receptor class C group 5 member B	https://www.ncbi.nlm.nih.gov/gene/?term=GPRC5B
GPRIN2	G protein regulated inducer of neurite outgrowth 2	https://www.ncbi.nlm.nih.gov/gene/?term=GPRIN2
GRHL3	grainyhead like transcription factor 3	https://www.ncbi.nlm.nih.gov/gene/?term=GRHL3
GRIA1	glutamate ionotropic receptor AMPA type subunit 1	https://www.ncbi.nlm.nih.gov/gene/?term=GRIA1
GRIA2	glutamate ionotropic receptor AMPA type subunit 2	https://www.ncbi.nlm.nih.gov/gene/?term=GRIA2
GRID1	glutamate ionotropic receptor delta type subunit 1	https://www.ncbi.nlm.nih.gov/gene/?term=GRID1
GRIK2	glutamate ionotropic receptor kainate type subunit 2	https://www.ncbi.nlm.nih.gov/gene/?term=GRIK2
GRIK3	glutamate ionotropic receptor kainate type subunit 3	https://www.ncbi.nlm.nih.gov/gene/?term=GRIK3
HADH	hydroxyacyl-CoA dehydrogenase	https://www.ncbi.nlm.nih.gov/gene/?term=HADH
HAS2	hyaluronan synthase 2	https://www.ncbi.nlm.nih.gov/gene/?term=HAS2
HEATR5B	HEAT repeat containing 5B	https://www.ncbi.nlm.nih.gov/gene/?term=HEATR5B
HECA	hdc homolog, cell cycle regulator	https://www.ncbi.nlm.nih.gov/gene/?term=HECA
HEPACAM2	HEPACAM family member 2	https://www.ncbi.nlm.nih.gov/gene/?term=HEPACAM2
HEPH	hephaestin	https://www.ncbi.nlm.nih.gov/gene/?term=HEPH
HERC2	HECT and RLD domain containing E3 ubiquitin protein ligase 2	https://www.ncbi.nlm.nih.gov/gene/?term=HERC2
HIPK2	homeodomain interacting protein kinase 2	https://www.ncbi.nlm.nih.gov/gene/?term=HIPK2
HMGA2	high mobility group AT-hook 2	https://www.ncbi.nlm.nih.gov/gene/?term=HMGA2
HMMR	hyaluronan mediated motility receptor	https://www.ncbi.nlm.nih.gov/gene/?term=HMMR
HOPX	HOP homeobox	https://www.ncbi.nlm.nih.gov/gene/?term=HOPX
HPS5	HPS5 biogenesis of lysosomal organelles complex 2 subunit 2	https://www.ncbi.nlm.nih.gov/gene/?term=HPS5
HS3ST4	heparan sulfate-glucosamine 3-sulfotransferase 4	https://www.ncbi.nlm.nih.gov/gene/?term=HS3ST4
HS6ST2	heparan sulfate 6-O-sulfotransferase 2	https://www.ncbi.nlm.nih.gov/gene/?term=HS6ST2

HSD3B7	hydroxy-delta-5-steroid dehydrogenase, 3 beta- and steroid delta-isomerase 7	https://www.ncbi.nlm.nih.gov/gene/?term=HSD3B7
HSPA13	heat shock protein family A (Hsp70) member 13	https://www.ncbi.nlm.nih.gov/gene/?term=HSPA13
HSPD1	heat shock protein family D (Hsp60) member 1	https://www.ncbi.nlm.nih.gov/gene/?term=HSPD1
HSPE1	heat shock protein family E (Hsp10) member 1	https://www.ncbi.nlm.nih.gov/gene/?term=HSPE1
HTR4	5-hydroxytryptamine receptor 4	https://www.ncbi.nlm.nih.gov/gene/?term=HTR4
IFT80	intraflagellar transport 80	https://www.ncbi.nlm.nih.gov/gene/?term=IFT80
IFT81	intraflagellar transport 81	https://www.ncbi.nlm.nih.gov/gene/?term=IFT81
IGF1	insulin like growth factor 1	https://www.ncbi.nlm.nih.gov/gene/?term=IGF1
IGF2	insulin like growth factor 2	https://www.ncbi.nlm.nih.gov/gene/?term=IGF2
IGHMBP2	immunoglobulin mu DNA binding protein 2	https://www.ncbi.nlm.nih.gov/gene/?term=IGHMBP2
IGSF1	immunoglobulin superfamily member 1	https://www.ncbi.nlm.nih.gov/gene/?term=IGSF1
IGSF3	immunoglobulin superfamily member 3	https://www.ncbi.nlm.nih.gov/gene/?term=IGSF3
IGSF9B	immunoglobulin superfamily member 9B	https://www.ncbi.nlm.nih.gov/gene/?term=IGSF9B
IKZF1	IKAROS family zinc finger 1	https://www.ncbi.nlm.nih.gov/gene/?term=IKZF1
IMMP2L	inner mitochondrial membrane peptidase subunit 2	https://www.ncbi.nlm.nih.gov/gene/?term=IMMP2L
INHBC	inhibin subunit beta C	https://www.ncbi.nlm.nih.gov/gene/?term=INHBC
INPP4B	inositol polyphosphate-4-phosphatase type II B	https://www.ncbi.nlm.nih.gov/gene/?term=INPP4B
INPP5J	inositol polyphosphate-5-phosphatase J	https://www.ncbi.nlm.nih.gov/gene/?term=INPP5J
IPO4	importin 4	https://www.ncbi.nlm.nih.gov/gene/?term=IPO4
IQCB1	IQ motif containing B1	https://www.ncbi.nlm.nih.gov/gene/?term=IQCB1
ISG15	ISG15 ubiquitin like modifier	https://www.ncbi.nlm.nih.gov/gene/?term=ISG15
ITGA2B	integrin subunit alpha 2b	https://www.ncbi.nlm.nih.gov/gene/?term=ITGA2B
ITGA9	integrin subunit alpha 9	https://www.ncbi.nlm.nih.gov/gene/?term=ITGA9
ITGBL1	integrin subunit beta like 1	https://www.ncbi.nlm.nih.gov/gene/?term=ITGBL1
ITPR3	inositol 1,4,5-trisphosphate receptor type 3	https://www.ncbi.nlm.nih.gov/gene/?term=ITPR3
JAM3	junctional adhesion molecule 3	https://www.ncbi.nlm.nih.gov/gene/?term=JAM3
JMJD1C	jumonji domain containing 1C	https://www.ncbi.nlm.nih.gov/gene/?term=JMJD1C

JPH3	junctophilin 3	https://www.ncbi.nlm.nih.gov/gene/?term=JPH3
JRKL	JRK like	https://www.ncbi.nlm.nih.gov/gene/?term=JRKL
KCNK10	potassium two pore domain channel subfamily K member 10	https://www.ncbi.nlm.nih.gov/gene/?term=KCNK10
KDM3A	lysine demethylase 3A	https://www.ncbi.nlm.nih.gov/gene/?term=KDM3A
KDM6B	lysine demethylase 6B	https://www.ncbi.nlm.nih.gov/gene/?term=KDM6B
KDR	kinase insert domain receptor	https://www.ncbi.nlm.nih.gov/gene/?term=KDR
KIAA0226	rubicon autophagy regulator	https://www.ncbi.nlm.nih.gov/gene/?term=KIAA0226
KIAA0556	KIAA0556	https://www.ncbi.nlm.nih.gov/gene/?term=KIAA0556
KIAA1549	KIAA1549	https://www.ncbi.nlm.nih.gov/gene/?term=KIAA1549
KIF1C	kinesin family member 1C	https://www.ncbi.nlm.nih.gov/gene/?term=KIF1C
KIF22	kinesin family member 22	https://www.ncbi.nlm.nih.gov/gene/?term=KIF22
KIF27	kinesin family member 27	https://www.ncbi.nlm.nih.gov/gene/?term=KIF27
KIRREL2	kirre like nephrin family adhesion molecule 2	https://www.ncbi.nlm.nih.gov/gene/?term=KIRREL2
KIT	KIT proto-oncogene, receptor tyrosine kinase	https://www.ncbi.nlm.nih.gov/gene/?term=KIT
KITLG	KIT ligand	https://www.ncbi.nlm.nih.gov/gene/?term=KITLG
KLF4	Kruppel like factor 4	https://www.ncbi.nlm.nih.gov/gene/?term=KLF4
KLHDC4	kelch domain containing 4	https://www.ncbi.nlm.nih.gov/gene/?term=KLHDC4
KRIT1	KRIT1 ankyrin repeat containing	https://www.ncbi.nlm.nih.gov/gene/?term=KRIT1
KRT71	keratin 71	https://www.ncbi.nlm.nih.gov/gene/?term=KRT71
KYNU	kynureninase	https://www.ncbi.nlm.nih.gov/gene/?term=KYNU
LAMC2	laminin subunit gamma 2	https://www.ncbi.nlm.nih.gov/gene/?term=LAMC2
LAMC3	laminin subunit gamma 3	https://www.ncbi.nlm.nih.gov/gene/?term=LAMC3
LAP3	leucine aminopeptidase 3	https://www.ncbi.nlm.nih.gov/gene/?term=LAP3
LATS2	large tumor suppressor kinase 2	https://www.ncbi.nlm.nih.gov/gene/?term=LATS2
LCAT	lecithin-cholesterol acyltransferase	https://www.ncbi.nlm.nih.gov/gene/?term=LCAT
LCLAT1	lysocardiolipin acyltransferase 1	https://www.ncbi.nlm.nih.gov/gene/?term=LCLAT1
LEPREL1	prolyl 3-hydroxylase 2	https://www.ncbi.nlm.nih.gov/gene/?term=LEPREL1

LHFPL3	LHFPL tetraspan subfamily member 3	https://www.ncbi.nlm.nih.gov/gene/?term=LHFPL3
LIAS	lipoic acid synthetase	https://www.ncbi.nlm.nih.gov/gene/?term=LIAS
LILRA6	leukocyte immunoglobulin like receptor A6	https://www.ncbi.nlm.nih.gov/gene/?term=LILRA6
LIMD1	LIM domains containing 1	https://www.ncbi.nlm.nih.gov/gene/?term=LIMD1
LIN28B	lin-28 homolog B	https://www.ncbi.nlm.nih.gov/gene/?term=LIN28B
LINC01927	long intergenic non-protein coding RNA 1927	https://www.ncbi.nlm.nih.gov/gene/?term=LINC01927
LINGO2	leucine rich repeat and Ig domain containing 2	https://www.ncbi.nlm.nih.gov/gene/?term=LINGO2
LMF1	lipase maturation factor 1	https://www.ncbi.nlm.nih.gov/gene/?term=LMF1
LRIG3	leucine rich repeats and immunoglobulin like domains 3	https://www.ncbi.nlm.nih.gov/gene/?term=LRIG3
LRP1B	LDL receptor related protein 1B	https://www.ncbi.nlm.nih.gov/gene/?term=LRP1B
LRRC32	leucine rich repeat containing 32	https://www.ncbi.nlm.nih.gov/gene/?term=LRRC32
LRRC36	leucine rich repeat containing 36	https://www.ncbi.nlm.nih.gov/gene/?term=LRRC36
LRRN3	leucine rich repeat neuronal 3	https://www.ncbi.nlm.nih.gov/gene/?term=LRRN3
LSM3	LSM3 homolog, U6 small nuclear RNA and mRNA degradation associated	https://www.ncbi.nlm.nih.gov/gene/?term=LSM3
LTF	lactotransferrin	https://www.ncbi.nlm.nih.gov/gene/?term=LTF
LYST	lysosomal trafficking regulator	https://www.ncbi.nlm.nih.gov/gene/?term=LYST
MAFK	MAF bZIP transcription factor K	https://www.ncbi.nlm.nih.gov/gene/?term=MAFK
MAGOH	mago homolog, exon junction complex subunit	https://www.ncbi.nlm.nih.gov/gene/?term=MAGOH
MAOA	monoamine oxidase A	https://www.ncbi.nlm.nih.gov/gene/?term=MAOA
MAOB	monoamine oxidase B	https://www.ncbi.nlm.nih.gov/gene/?term=MAOB
MAP3K1	mitogen-activated protein kinase kinase kinase 1	https://www.ncbi.nlm.nih.gov/gene/?term=MAP3K1
MAP3K4	mitogen-activated protein kinase kinase kinase 4	https://www.ncbi.nlm.nih.gov/gene/?term=MAP3K4
MAP7D2	MAP7 domain containing 2	https://www.ncbi.nlm.nih.gov/gene/?term=MAP7D2
MAP7D3	MAP7 domain containing 3	https://www.ncbi.nlm.nih.gov/gene/?term=MAP7D3
MAPK10	mitogen-activated protein kinase 10	https://www.ncbi.nlm.nih.gov/gene/?term=MAPK10
MARCH10	membrane associated ring-CH-type finger 10	https://www.ncbi.nlm.nih.gov/gene/?term=MARCH10
MARCH7	membrane associated ring-CH-type finger 7	https://www.ncbi.nlm.nih.gov/gene/?term=MARCH7

MARK2	microtubule affinity regulating kinase 2	https://www.ncbi.nlm.nih.gov/gene/?term=MARK2
MARK3	microtubule affinity regulating kinase 3	https://www.ncbi.nlm.nih.gov/gene/?term=MARK3
MARVELD3	MARVEL domain containing 3	https://www.ncbi.nlm.nih.gov/gene/?term=MARVELD3
MATN2	matrilin 2	https://www.ncbi.nlm.nih.gov/gene/?term=MATN2
MBD2	methyl-CpG binding domain protein 2	https://www.ncbi.nlm.nih.gov/gene/?term=MBD2
MBP	myelin basic protein	https://www.ncbi.nlm.nih.gov/gene/?term=MBP
MC1R	melanocortin 1 receptor	https://www.ncbi.nlm.nih.gov/gene/?term=MC1R
MC2R	melanocortin 2 receptor	https://www.ncbi.nlm.nih.gov/gene/?term=MC2R
MC4R	melanocortin 4 receptor	https://www.ncbi.nlm.nih.gov/gene/?term=MC4R
MCF2	MCF.2 cell line derived transforming sequence	https://www.ncbi.nlm.nih.gov/gene/?term=MCF2
MCHR2	melanin concentrating hormone receptor 2	https://www.ncbi.nlm.nih.gov/gene/?term=MCHR2
MED23	mediator complex subunit 23	https://www.ncbi.nlm.nih.gov/gene/?term=MED23
MERTK	MER proto-oncogene, tyrosine kinase	https://www.ncbi.nlm.nih.gov/gene/?term=MERTK
METAP2	methionyl aminopeptidase 2	https://www.ncbi.nlm.nih.gov/gene/?term=METAP2
METTL22	methyltransferase like 22	https://www.ncbi.nlm.nih.gov/gene/?term=METTL22
METTL8	methyltransferase like 8	https://www.ncbi.nlm.nih.gov/gene/?term=METTL8
MFAP3	microfibril associated protein 3	https://www.ncbi.nlm.nih.gov/gene/?term=MFAP3
MGAM	maltase-glucoamylase	https://www.ncbi.nlm.nih.gov/gene/?term=MGAM
MGC12345	MGC12345	https://www.ncbi.nlm.nih.gov/gene/?term=MGC12345
MIER3	MIER family member 3	https://www.ncbi.nlm.nih.gov/gene/?term=MIER3
MIF4GD	MIF4G domain containing	https://www.ncbi.nlm.nih.gov/gene/?term=MIF4GD
MIIP	migration and invasion inhibitory protein	https://www.ncbi.nlm.nih.gov/gene/?term=MIIP
MINOS1	mitochondrial contact site and cristae organizing system subunit 10	https://www.ncbi.nlm.nih.gov/gene/?term=MINOS1
MITF	melanocyte inducing transcription factor	https://www.ncbi.nlm.nih.gov/gene/?term=MITF
MKKS	McKusick-Kaufman syndrome	https://www.ncbi.nlm.nih.gov/gene/?term=MKKS
MMP16	matrix metallopeptidase 16	https://www.ncbi.nlm.nih.gov/gene/?term=MMP16
MOB4	MOB family member 4, phocein	https://www.ncbi.nlm.nih.gov/gene/?term=MOB4

MORC1	MORC family CW-type zinc finger 1	https://www.ncbi.nlm.nih.gov/gene/?term=MORC1
MPV17L	MPV17 mitochondrial inner membrane protein like	https://www.ncbi.nlm.nih.gov/gene/?term=MPV17L
MRPL11	mitochondrial ribosomal protein L11	https://www.ncbi.nlm.nih.gov/gene/?term=MRPL11
MRPL52	mitochondrial ribosomal protein L52	https://www.ncbi.nlm.nih.gov/gene/?term=MRPL52
MSI2	musashi RNA binding protein 2	https://www.ncbi.nlm.nih.gov/gene/?term=MSI2
MSRB3	methionine sulfoxide reductase B3	https://www.ncbi.nlm.nih.gov/gene/?term=MSRB3
MSTN	myostatin	https://www.ncbi.nlm.nih.gov/gene/?term=MSTN
MT1F	metallothionein 1F	https://www.ncbi.nlm.nih.gov/gene/?term=MT1F
MT1L	metallothionein 1L, pseudogene	https://www.ncbi.nlm.nih.gov/gene/?term=MT1L
MT2A	metallothionein 2A	https://www.ncbi.nlm.nih.gov/gene/?term=MT2A
MTIF2	mitochondrial translational initiation factor 2	https://www.ncbi.nlm.nih.gov/gene/?term=MTIF2
MTRF1	mitochondrial translation release factor 1	https://www.ncbi.nlm.nih.gov/gene/?term=MTRF1
MURC	caveolae associated protein 4	https://www.ncbi.nlm.nih.gov/gene/?term=MURC
MVK	mevalonate kinase	https://www.ncbi.nlm.nih.gov/gene/?term=MVK
MYBPC1	myosin binding protein C1	https://www.ncbi.nlm.nih.gov/gene/?term=MYBPC1
MYLK3	myosin light chain kinase 3	https://www.ncbi.nlm.nih.gov/gene/?term=MYLK3
MYO15A	myosin XVA	https://www.ncbi.nlm.nih.gov/gene/?term=MYO15A
MYO9A	myosin IXA	https://www.ncbi.nlm.nih.gov/gene/?term=MYO9A
MYOF	myoferlin	https://www.ncbi.nlm.nih.gov/gene/?term=MYOF
NAPRT1	nicotinate phosphoribosyltransferase	https://www.ncbi.nlm.nih.gov/gene/?term=NAPRT1
NCAPD3	non-SMC condensin II complex subunit D3	https://www.ncbi.nlm.nih.gov/gene/?term=NCAPD3
NCAPG	non-SMC condensin I complex subunit G	https://www.ncbi.nlm.nih.gov/gene/?term=NCAPG
NCOA6	nuclear receptor coactivator 6	https://www.ncbi.nlm.nih.gov/gene/?term=NCOA6
NCTIN1		
NDUFB1	NADH:ubiquinone oxidoreductase subunit B1	https://www.ncbi.nlm.nih.gov/gene/?term=NDUFB1
NEK1	NIMA related kinase 1	https://www.ncbi.nlm.nih.gov/gene/?term=NEK1
NEK4	NIMA related kinase 4	https://www.ncbi.nlm.nih.gov/gene/?term=NEK4

NFAM1	NFAT activating protein with ITAM motif 1	https://www.ncbi.nlm.nih.gov/gene/?term=NFAM1
NFKBIZ	NFKB inhibitor zeta	https://www.ncbi.nlm.nih.gov/gene/?term=NFKBIZ
NID2	nidogen 2	https://www.ncbi.nlm.nih.gov/gene/?term=NID2
NINJ1	ninjurin 1	https://www.ncbi.nlm.nih.gov/gene/?term=NINJ1
NIPA2	NIPA magnesium transporter 2	https://www.ncbi.nlm.nih.gov/gene/?term=NIPA2
NIPBL	NIPBL cohesin loading factor	https://www.ncbi.nlm.nih.gov/gene/?term=NIPBL
NKAIN2	sodium/potassium transporting ATPase interacting 2	https://www.ncbi.nlm.nih.gov/gene/?term=NKAIN2
NOCT	nocturnin	https://www.ncbi.nlm.nih.gov/gene/?term=NOCT
NOL4	nucleolar protein 4	https://www.ncbi.nlm.nih.gov/gene/?term=NOL4
NOLC1	nucleolar and coiled-body phosphoprotein 1	https://www.ncbi.nlm.nih.gov/gene/?term=NOLC1
NOSTRIN	nitric oxide synthase trafficking	https://www.ncbi.nlm.nih.gov/gene/?term=NOSTRIN
NOTCH2	notch receptor 2	https://www.ncbi.nlm.nih.gov/gene/?term=NOTCH2
NPAS3	neuronal PAS domain protein 3	https://www.ncbi.nlm.nih.gov/gene/?term=NPAS3
NPFFR2	neuropeptide FF receptor 2	https://www.ncbi.nlm.nih.gov/gene/?term=NPFFR2
NPTX1	neuronal pentraxin 1	https://www.ncbi.nlm.nih.gov/gene/?term=NPTX1
NR2F2	nuclear receptor subfamily 2 group F member 2	https://www.ncbi.nlm.nih.gov/gene/?term=NR2F2
NR3C1	nuclear receptor subfamily 3 group C member 1	https://www.ncbi.nlm.nih.gov/gene/?term=NR3C1
NR3C2	nuclear receptor subfamily 3 group C member 2	https://www.ncbi.nlm.nih.gov/gene/?term=NR3C2
NRF1	nuclear respiratory factor 1	https://www.ncbi.nlm.nih.gov/gene/?term=NRF1
NRG2	nuclear factor, erythroid 2 like 1	https://www.ncbi.nlm.nih.gov/gene/?term=NRG2
NRG4	neuregulin 2	https://www.ncbi.nlm.nih.gov/gene/?term=NRG4
NRSA2	neuregulin 4	https://www.ncbi.nlm.nih.gov/gene/?term=NRSA2
NRXN1	neurexin 1	https://www.ncbi.nlm.nih.gov/gene/?term=NRXN1
NT5DC2	5'-nucleotidase domain containing 2	https://www.ncbi.nlm.nih.gov/gene/?term=NT5DC2
NTAN1	N-terminal asparagine amidase	https://www.ncbi.nlm.nih.gov/gene/?term=NTAN1
NTM	neurotrimin	https://www.ncbi.nlm.nih.gov/gene/?term=NTM
NUDT15	nudix hydrolase 15	https://www.ncbi.nlm.nih.gov/gene/?term=NUDT15

NUMB	NUMB endocytic adaptor protein	https://www.ncbi.nlm.nih.gov/gene/?term=NUMB
NUP133	nucleoporin 133	https://www.ncbi.nlm.nih.gov/gene/?term=NUP133
NUP54	nucleoporin 54	https://www.ncbi.nlm.nih.gov/gene/?term=NUP54
NXPE3	neurexophilin and PC-esterase domain family member 3	https://www.ncbi.nlm.nih.gov/gene/?term=NXPE3
OLIG1	oligodendrocyte transcription factor 1	https://www.ncbi.nlm.nih.gov/gene/?term=OLIG1
OMA1	OMA1 zinc metallopeptidase	https://www.ncbi.nlm.nih.gov/gene/?term=OMA1
OPCML	opioid binding protein/cell adhesion molecule like	https://www.ncbi.nlm.nih.gov/gene/?term=OPCML
OPTC	opticin	https://www.ncbi.nlm.nih.gov/gene/?term=OPTC
OR10K1	olfactory receptor family 10 subfamily K member 1	https://www.ncbi.nlm.nih.gov/gene/?term=OR10K1
OR13C8	olfactory receptor family 13 subfamily C member 8	https://www.ncbi.nlm.nih.gov/gene/?term=OR13C8
OR2B11	olfactory receptor family 2 subfamily B member 11	https://www.ncbi.nlm.nih.gov/gene/?term=OR2B11
OR4D6	olfactory receptor family 4 subfamily D member 6	https://www.ncbi.nlm.nih.gov/gene/?term=OR4D6
OR51A7	olfactory receptor family 51 subfamily A member 7	https://www.ncbi.nlm.nih.gov/gene/?term=OR51A7
OR9A4	olfactory receptor family 9 subfamily A member 4	https://www.ncbi.nlm.nih.gov/gene/?term=OR9A4
OTOF	otoferlin	https://www.ncbi.nlm.nih.gov/gene/?term=OTOF
PAFAH2	platelet activating factor acetylhydrolase 2	https://www.ncbi.nlm.nih.gov/gene/?term=PAFAH2
PARP12	poly(ADP-ribose) polymerase family member 12	https://www.ncbi.nlm.nih.gov/gene/?term=PARP12
PARVG	parvin gamma	https://www.ncbi.nlm.nih.gov/gene/?term=PARVG
PAX2	paired box 2	https://www.ncbi.nlm.nih.gov/gene/?term=PAX2
PAX3	paired box 3	https://www.ncbi.nlm.nih.gov/gene/?term=PAX3
PCDH18	protocadherin 18	https://www.ncbi.nlm.nih.gov/gene/?term=PCDH18
PCDHA1	protocadherin alpha 1	https://www.ncbi.nlm.nih.gov/gene/?term=PCDHA1
PCDHB4	protocadherin beta 4	https://www.ncbi.nlm.nih.gov/gene/?term=PCDHB4
PCSK5	proprotein convertase subtilisin/kexin type 5	https://www.ncbi.nlm.nih.gov/gene/?term=PCSK5
PDE4D	phosphodiesterase 4D	https://www.ncbi.nlm.nih.gov/gene/?term=PDE4D
PDE4DIP	phosphodiesterase 4D interacting protein	https://www.ncbi.nlm.nih.gov/gene/?term=PDE4DIP
PDE5A	phosphodiesterase 5A	https://www.ncbi.nlm.nih.gov/gene/?term=PDE5A

PDE7B	phosphodiesterase 7B	https://www.ncbi.nlm.nih.gov/gene/?term=PDE7B
PDILT	protein disulfide isomerase like, testis expressed	https://www.ncbi.nlm.nih.gov/gene/?term=PDILT
PDRG1	p53 and DNA damage regulated 1	https://www.ncbi.nlm.nih.gov/gene/?term=PDRG1
PDXDC1	pyridoxal dependent decarboxylase domain containing 1	https://www.ncbi.nlm.nih.gov/gene/?term=PDXDC1
PEX7	peroxisomal biogenesis factor 7	https://www.ncbi.nlm.nih.gov/gene/?term=PEX7
PHF2	PHD finger protein 2	https://www.ncbi.nlm.nih.gov/gene/?term=PHF2
PHF20	PHD finger protein 20	https://www.ncbi.nlm.nih.gov/gene/?term=PHF20
PHLDB3	pleckstrin homology like domain family B member 3	https://www.ncbi.nlm.nih.gov/gene/?term=PHLDB3
PIK3C3	phosphatidylinositol 3-kinase catalytic subunit type 3	https://www.ncbi.nlm.nih.gov/gene/?term=PIK3C3
PITRM1	pitrilysin metallopeptidase 1	https://www.ncbi.nlm.nih.gov/gene/?term=PITRM1
PJA2	praja ring finger ubiquitin ligase 2	https://www.ncbi.nlm.nih.gov/gene/?term=PJA2
PKD1L1	polycystin 1 like 1, transient receptor potential channel interacting	https://www.ncbi.nlm.nih.gov/gene/?term=PKD1L1
PLA2G2E	phospholipase A2 group IIE	https://www.ncbi.nlm.nih.gov/gene/?term=PLA2G2E
PLA2G3	phospholipase A2 group III	https://www.ncbi.nlm.nih.gov/gene/?term=PLA2G3
PLAC1	placenta enriched 1	https://www.ncbi.nlm.nih.gov/gene/?term=PLAC1
PLAC8L1	PLAC8 like 1	https://www.ncbi.nlm.nih.gov/gene/?term=PLAC8L1
PLAG1	PLAG1 zinc finger	https://www.ncbi.nlm.nih.gov/gene/?term=PLAG1
PLCE1	phospholipase C epsilon 1	https://www.ncbi.nlm.nih.gov/gene/?term=PLCE1
PLEKHH1	pleckstrin homology, MyTH4 and FERM domain containing H1	https://www.ncbi.nlm.nih.gov/gene/?term=PLEKHH1
PLEKHM3	pleckstrin homology domain containing M3	https://www.ncbi.nlm.nih.gov/gene/?term=PLEKHM3
PLIN3	perilipin 3	https://www.ncbi.nlm.nih.gov/gene/?term=PLIN3
PLXNA4	plexin A4	https://www.ncbi.nlm.nih.gov/gene/?term=PLXNA4
PMEL	premelanosome protein	https://www.ncbi.nlm.nih.gov/gene/?term=PMEL
PML	promyelocytic leukemia	https://www.ncbi.nlm.nih.gov/gene/?term=PML
Pol		
POLI	DNA polymerase iota	https://www.ncbi.nlm.nih.gov/gene/?term=POLI
POLR1E	RNA polymerase I subunit E	https://www.ncbi.nlm.nih.gov/gene/?term=POLR1E

POMC	proopiomelanocortin	https://www.ncbi.nlm.nih.gov/gene/?term=POMC
POP1	POP1 homolog, ribonuclease P/MRP subunit	https://www.ncbi.nlm.nih.gov/gene/?term=POP1
PPAP2A	phospholipid phosphatase 1	https://www.ncbi.nlm.nih.gov/gene/?term=PPAP2A
PPAPDC1B	phospholipid phosphatase 5	https://www.ncbi.nlm.nih.gov/gene/?term=PPAPDC1B
PPARD	peroxisome proliferator activated receptor delta	https://www.ncbi.nlm.nih.gov/gene/?term=PPARD
PPFIBP1	PPFIA binding protein 1	https://www.ncbi.nlm.nih.gov/gene/?term=PPFIBP1
PPM1D	protein phosphatase, Mg2+/Mn2+ dependent 1D	https://www.ncbi.nlm.nih.gov/gene/?term=PPM1D
PPP1R13B	protein phosphatase 1 regulatory subunit 13B	https://www.ncbi.nlm.nih.gov/gene/?term=PPP1R13B
PPP2CA	protein phosphatase 2 catalytic subunit alpha	https://www.ncbi.nlm.nih.gov/gene/?term=PPP2CA
PRICKLE4	prickle planar cell polarity protein 4	https://www.ncbi.nlm.nih.gov/gene/?term=PRICKLE4
PRKAG1	protein kinase AMP-activated non-catalytic subunit gamma 1	https://www.ncbi.nlm.nih.gov/gene/?term=PRKAG1
PRKAG3	protein kinase AMP-activated non-catalytic subunit gamma 3	https://www.ncbi.nlm.nih.gov/gene/?term=PRKAG3
PRKCZ	protein kinase C zeta	https://www.ncbi.nlm.nih.gov/gene/?term=PRKCZ
PRKG2	protein kinase cGMP-dependent 2	https://www.ncbi.nlm.nih.gov/gene/?term=PRKG2
PRMT3	protein arginine methyltransferase 3	https://www.ncbi.nlm.nih.gov/gene/?term=PRMT3
PROM1	prominin 1	https://www.ncbi.nlm.nih.gov/gene/?term=PROM1
PRR11	proline rich 11	https://www.ncbi.nlm.nih.gov/gene/?term=PRR11
PRX	periaxin	https://www.ncbi.nlm.nih.gov/gene/?term=PRX
PSMB7	proteasome 20S subunit beta 7	https://www.ncbi.nlm.nih.gov/gene/?term=PSMB7
PSPH	phosphoserine phosphatase	https://www.ncbi.nlm.nih.gov/gene/?term=PSPH
PSTK	phosphoseryl-tRNA kinase	https://www.ncbi.nlm.nih.gov/gene/?term=PSTK
PTPN4	protein tyrosine phosphatase non-receptor type 4	https://www.ncbi.nlm.nih.gov/gene/?term=PTPN4
PTPRR	protein tyrosine phosphatase receptor type R	https://www.ncbi.nlm.nih.gov/gene/?term=PTPRR
PTPRS	protein tyrosine phosphatase receptor type S	https://www.ncbi.nlm.nih.gov/gene/?term=PTPRS
PUSL1	pseudouridine synthase like 1	https://www.ncbi.nlm.nih.gov/gene/?term=PUSL1
PVRL3	nectin cell adhesion molecule 3	https://www.ncbi.nlm.nih.gov/gene/?term=PVRL3
Q2ABD2	taste 2 receptor member 38	https://www.ncbi.nlm.nih.gov/gene/?term=Q2ABD2

RAB3GAP1	RAB3 GTPase activating protein catalytic subunit 1	https://www.ncbi.nlm.nih.gov/gene/?term=RAB3GAP1
RABGAP1L	RAB GTPase activating protein 1 like	https://www.ncbi.nlm.nih.gov/gene/?term=RABGAP1L
RABL3	RAB, member of RAS oncogene family like 3	https://www.ncbi.nlm.nih.gov/gene/?term=RABL3
RALGAPA2	Ral GTPase activating protein catalytic subunit alpha 2	https://www.ncbi.nlm.nih.gov/gene/?term=RALGAPA2
RALY	RALY heterogeneous nuclear ribonucleoprotein	https://www.ncbi.nlm.nih.gov/gene/?term=RALY
RANBP17	RAN binding protein 17	https://www.ncbi.nlm.nih.gov/gene/?term=RANBP17
RAPH1	Ras association (RalGDS/AF-6) and pleckstrin homology domains 1	https://www.ncbi.nlm.nih.gov/gene/?term=RAPH1
RASGEF1B	RasGEF domain family member 1B	https://www.ncbi.nlm.nih.gov/gene/?term=RASGEF1B
RBM11	RNA binding motif protein 11	https://www.ncbi.nlm.nih.gov/gene/?term=RBM11
RBP5	retinol binding protein 5	https://www.ncbi.nlm.nih.gov/gene/?term=RBP5
RCSD1	RCSD domain containing 1	https://www.ncbi.nlm.nih.gov/gene/?term=RCSD1
REEP1	receptor accessory protein 1	https://www.ncbi.nlm.nih.gov/gene/?term=REEP1
RELL1	RELT like 1	https://www.ncbi.nlm.nih.gov/gene/?term=RELL1
RELT	RELT TNF receptor	https://www.ncbi.nlm.nih.gov/gene/?term=RELT
RET	ret proto-oncogene	https://www.ncbi.nlm.nih.gov/gene/?term=RET
RFTN2	raftlin family member 2	https://www.ncbi.nlm.nih.gov/gene/?term=RFTN2
RG9MTD3	tRNA methyltransferase 10B	https://www.ncbi.nlm.nih.gov/gene/?term=RG9MTD3
RHBDD1	rhomboid domain containing 1	https://www.ncbi.nlm.nih.gov/gene/?term=RHBDD1
RHPN1	rhophilin Rho GTPase binding protein 1	https://www.ncbi.nlm.nih.gov/gene/?term=RHPN1
RIMKLA	ribosomal modification protein rimK like family member A	https://www.ncbi.nlm.nih.gov/gene/?term=RIMKLA
RNASE6	ribonuclease A family member k6	https://www.ncbi.nlm.nih.gov/gene/?term=RNASE6
RNF103	ring finger protein 103	https://www.ncbi.nlm.nih.gov/gene/?term=RNF103
RNF144B	ring finger protein 144B	https://www.ncbi.nlm.nih.gov/gene/?term=RNF144B
RNPC3	RNA binding region (RNP1, RRM) containing 3	https://www.ncbi.nlm.nih.gov/gene/?term=RNPC3
ROBO1	roundabout guidance receptor 1	https://www.ncbi.nlm.nih.gov/gene/?term=ROBO1
RPL3	ribosomal protein L3	https://www.ncbi.nlm.nih.gov/gene/?term=RPL3
RPL31	ribosomal protein L31	https://www.ncbi.nlm.nih.gov/gene/?term=RPL31

RRN3	RRN3 homolog, RNA polymerase I transcription factor	https://www.ncbi.nlm.nih.gov/gene/?term=RRN3
RRN3P1	RRN3 pseudogene 1	https://www.ncbi.nlm.nih.gov/gene/?term=RRN3P1
RRNRP2		
RSL1D1	ribosomal L1 domain containing 1	https://www.ncbi.nlm.nih.gov/gene/?term=RSL1D1
RSPO2	R-spondin 2	https://www.ncbi.nlm.nih.gov/gene/?term=RSPO2
RTP3	receptor transporter protein 3	https://www.ncbi.nlm.nih.gov/gene/?term=RTP3
RXFP2	relaxin family peptide receptor 2	https://www.ncbi.nlm.nih.gov/gene/?term=RXFP2
RYR1	ryanodine receptor 1	https://www.ncbi.nlm.nih.gov/gene/?term=RYR1
S100A12	S100 calcium binding protein A12	https://www.ncbi.nlm.nih.gov/gene/?term=S100A12
SAE1	SUMO1 activating enzyme subunit 1	https://www.ncbi.nlm.nih.gov/gene/?term=SAE1
SCARB2	scavenger receptor class B member 2	https://www.ncbi.nlm.nih.gov/gene/?term=SCARB2
SCN9A	sodium voltage-gated channel alpha subunit 9	https://www.ncbi.nlm.nih.gov/gene/?term=SCN9A
SCP2D1	SCP2 sterol binding domain containing 1	https://www.ncbi.nlm.nih.gov/gene/?term=SCP2D1
SCPEP1	serine carboxypeptidase 1	https://www.ncbi.nlm.nih.gov/gene/?term=SCPEP1
SCRIB	scribble planar cell polarity protein	https://www.ncbi.nlm.nih.gov/gene/?term=SCRIB
SDAD1	SDA1 domain containing 1	https://www.ncbi.nlm.nih.gov/gene/?term=SDAD1
SDHAF3	succinate dehydrogenase complex assembly factor 3	https://www.ncbi.nlm.nih.gov/gene/?term=SDHAF3
SDK2	sidekick cell adhesion molecule 2	https://www.ncbi.nlm.nih.gov/gene/?term=SDK2
SEC24A	SEC24 homolog A, COPII coat complex component	https://www.ncbi.nlm.nih.gov/gene/?term=SEC24A
SEC63	SEC63 homolog, protein translocation regulator	https://www.ncbi.nlm.nih.gov/gene/?term=SEC63
SEMA3D	semaphorin 3D	https://www.ncbi.nlm.nih.gov/gene/?term=SEMA3D
SEMA6A	semaphorin 6A	https://www.ncbi.nlm.nih.gov/gene/?term=SEMA6A
SENP5	SUMO specific peptidase 5	https://www.ncbi.nlm.nih.gov/gene/?term=SENP5
SENP7	SUMO specific peptidase 7	https://www.ncbi.nlm.nih.gov/gene/?term=SENP7
SEPT10	septin 10	https://www.ncbi.nlm.nih.gov/gene/?term=SEPT10
SERINC3	serine incorporator 3	https://www.ncbi.nlm.nih.gov/gene/?term=SERINC3
SETBP1	SET binding protein 1	https://www.ncbi.nlm.nih.gov/gene/?term=SETBP1

SETD9	SET domain containing 9	https://www.ncbi.nlm.nih.gov/gene/?term=SETD9
SETMAR	SET domain and mariner transposase fusion gene	https://www.ncbi.nlm.nih.gov/gene/?term=SETMAR
SF3B1	splicing factor 3b subunit 1	https://www.ncbi.nlm.nih.gov/gene/?term=SF3B1
SGCD	sarcoglycan delta	https://www.ncbi.nlm.nih.gov/gene/?term=SGCD
SH2D5	SH2 domain containing 5	https://www.ncbi.nlm.nih.gov/gene/?term=SH2D5
SH3GL2	SH3 domain containing GRB2 like 2, endophilin A1	https://www.ncbi.nlm.nih.gov/gene/?term=SH3GL2
SHC4	SHC adaptor protein 4	https://www.ncbi.nlm.nih.gov/gene/?term=SHC4
SIAE	sialic acid acetylesterase	https://www.ncbi.nlm.nih.gov/gene/?term=SIAE
SKA2	spindle and kinetochore associated complex subunit 2	https://www.ncbi.nlm.nih.gov/gene/?term=SKA2
SKI	SKI proto-oncogene	https://www.ncbi.nlm.nih.gov/gene/?term=SKI
SKP1	S-phase kinase associated protein 1	https://www.ncbi.nlm.nih.gov/gene/?term=SKP1
SLC22A13	solute carrier family 22 member 13	https://www.ncbi.nlm.nih.gov/gene/?term=SLC22A13
SLC22A15	solute carrier family 22 member 15	https://www.ncbi.nlm.nih.gov/gene/?term=SLC22A15
SLC22A18	solute carrier family 22 member 18	https://www.ncbi.nlm.nih.gov/gene/?term=SLC22A18
SLC25A38	solute carrier family 25 member 38	https://www.ncbi.nlm.nih.gov/gene/?term=SLC25A38
SLC35D1	solute carrier family 35 member D1	https://www.ncbi.nlm.nih.gov/gene/?term=SLC35D1
SLC35F5	solute carrier family 35 member F5	https://www.ncbi.nlm.nih.gov/gene/?term=SLC35F5
SLC39A7	solute carrier family 39 member 7	https://www.ncbi.nlm.nih.gov/gene/?term=SLC39A7
SLC39A8	solute carrier family 39 member 8	https://www.ncbi.nlm.nih.gov/gene/?term=SLC39A8
SLC41A2	solute carrier family 41 member 2	https://www.ncbi.nlm.nih.gov/gene/?term=SLC41A2
SLC43A1	solute carrier family 43 member 1	https://www.ncbi.nlm.nih.gov/gene/?term=SLC43A1
SLC46A1	solute carrier family 46 member 1	https://www.ncbi.nlm.nih.gov/gene/?term=SLC46A1
SLC5A1	solute carrier family 5 member 1	https://www.ncbi.nlm.nih.gov/gene/?term=SLC5A1
SLC5A4	solute carrier family 5 member 4	https://www.ncbi.nlm.nih.gov/gene/?term=SLC5A4
SLC6A1	solute carrier family 6 member 1	https://www.ncbi.nlm.nih.gov/gene/?term=SLC6A1
SLC6A17	solute carrier family 6 member 17	https://www.ncbi.nlm.nih.gov/gene/?term=SLC6A17
SLC9A6	solute carrier family 9 member A6	https://www.ncbi.nlm.nih.gov/gene/?term=SLC9A6

SLCO1A2	solute carrier organic anion transporter family member 1A2	https://www.ncbi.nlm.nih.gov/gene/?term=SLCO1A2
SMAD2	SMAD family member 2	https://www.ncbi.nlm.nih.gov/gene/?term=SMAD2
SMC4	structural maintenance of chromosomes 4	https://www.ncbi.nlm.nih.gov/gene/?term=SMC4
SMG1	SMG1 nonsense mediated mRNA decay associated PI3K related kinase	https://www.ncbi.nlm.nih.gov/gene/?term=SMG1
SMG6	SMG6 nonsense mediated mRNA decay factor	https://www.ncbi.nlm.nih.gov/gene/?term=SMG6
SMIM23	small integral membrane protein 23	https://www.ncbi.nlm.nih.gov/gene/?term=SMIM23
SMO	smoothened, frizzled class receptor	https://www.ncbi.nlm.nih.gov/gene/?term=SMO
SMYD2	SET and MYND domain containing 2	https://www.ncbi.nlm.nih.gov/gene/?term=SMYD2
SNAP29	synaptosome associated protein 29	https://www.ncbi.nlm.nih.gov/gene/?term=SNAP29
SNCG	synuclein gamma	https://www.ncbi.nlm.nih.gov/gene/?term=SNCG
SNRPD1	small nuclear ribonucleoprotein D1 polypeptide	https://www.ncbi.nlm.nih.gov/gene/?term=SNRPD1
SOCS4	suppressor of cytokine signaling 4	https://www.ncbi.nlm.nih.gov/gene/?term=SOCS4
SOX10	SRY-box transcription factor 10	https://www.ncbi.nlm.nih.gov/gene/?term=SOX10
SOX2	SRY-box transcription factor 2	https://www.ncbi.nlm.nih.gov/gene/?term=SOX2
SOX6	SRY-box transcription factor 6	https://www.ncbi.nlm.nih.gov/gene/?term=SOX6
SOX9	SRY-box transcription factor 9	https://www.ncbi.nlm.nih.gov/gene/?term=SOX9
SPATA19	spermatogenesis associated 19	https://www.ncbi.nlm.nih.gov/gene/?term=SPATA19
SPATA21	spermatogenesis associated 21	https://www.ncbi.nlm.nih.gov/gene/?term=SPATA21
SPATA7	spermatogenesis associated 7	https://www.ncbi.nlm.nih.gov/gene/?term=SPATA7
SPERT	chibby family member 2	https://www.ncbi.nlm.nih.gov/gene/?term=SPERT
SPHKAP	SPHK1 interactor, AKAP domain containing	https://www.ncbi.nlm.nih.gov/gene/?term=SPHKAP
SPINT1	serine peptidase inhibitor, Kunitz type 1	https://www.ncbi.nlm.nih.gov/gene/?term=SPINT1
SPTAN1	spectrin alpha, non-erythrocytic 1	https://www.ncbi.nlm.nih.gov/gene/?term=SPTAN1
SPTBN5	spectrin beta, non-erythrocytic 5	https://www.ncbi.nlm.nih.gov/gene/?term=SPTBN5
SREBF1	sterol regulatory element binding transcription factor 1	https://www.ncbi.nlm.nih.gov/gene/?term=SREBF1
SRP72	signal recognition particle 72	https://www.ncbi.nlm.nih.gov/gene/?term=SRP72
SRRM2	serine/arginine repetitive matrix 2	https://www.ncbi.nlm.nih.gov/gene/?term=SRRM2

STAB1	stabilin 1	https://www.ncbi.nlm.nih.gov/gene/?term=STAB1
STARD5	StAR related lipid transfer domain containing 5	https://www.ncbi.nlm.nih.gov/gene/?term=STARD5
STARD6	StAR related lipid transfer domain containing 6	https://www.ncbi.nlm.nih.gov/gene/?term=STARD6
STC2	stanniocalcin 2	https://www.ncbi.nlm.nih.gov/gene/?term=STC2
STK10	serine/threonine kinase 10	https://www.ncbi.nlm.nih.gov/gene/?term=STK10
STK11IP	serine/threonine kinase 11 interacting protein	https://www.ncbi.nlm.nih.gov/gene/?term=STK11IP
STS	steroid sulfatase	https://www.ncbi.nlm.nih.gov/gene/?term=STS
STX7	syntaxin 7	https://www.ncbi.nlm.nih.gov/gene/?term=STX7
STXBP6	syntaxin binding protein 6	https://www.ncbi.nlm.nih.gov/gene/?term=STXBP6
SUN3	Sad1 and UNC84 domain containing 3	https://www.ncbi.nlm.nih.gov/gene/?term=SUN3
SURF2	surfeit 2	https://www.ncbi.nlm.nih.gov/gene/?term=SURF2
SUSD3	sushi domain containing 3	https://www.ncbi.nlm.nih.gov/gene/?term=SUSD3
SYNJ2	synaptojanin 2	https://www.ncbi.nlm.nih.gov/gene/?term=SYNJ2
SYNM	synemin	https://www.ncbi.nlm.nih.gov/gene/?term=SYNM
SYTL1	synaptotagmin like 1	https://www.ncbi.nlm.nih.gov/gene/?term=SYTL1
TAOK1	TAO kinase 1	https://www.ncbi.nlm.nih.gov/gene/?term=TAOK1
TAS2R1	taste 2 receptor member 1	https://www.ncbi.nlm.nih.gov/gene/?term=TAS2R1
TAS2R16	taste 2 receptor member 16	https://www.ncbi.nlm.nih.gov/gene/?term=TAS2R16
TAS2R3	taste 2 receptor member 3	https://www.ncbi.nlm.nih.gov/gene/?term=TAS2R3
TAS2R38	taste 2 receptor member 38	https://www.ncbi.nlm.nih.gov/gene/?term=TAS2R38
TBC1D9	TBC1 domain family member 9	https://www.ncbi.nlm.nih.gov/gene/?term=TBC1D9
TBXAS1	thromboxane A synthase 1	https://www.ncbi.nlm.nih.gov/gene/?term=TBXAS1
TCOF1	treacle ribosome biogenesis factor 1	https://www.ncbi.nlm.nih.gov/gene/?term=TCOF1
TCTN1	tectonic family member 1	https://www.ncbi.nlm.nih.gov/gene/?term=TCTN1
TCTN3	tectonic family member 3	https://www.ncbi.nlm.nih.gov/gene/?term=TCTN3
TEKT3	tektin 3	https://www.ncbi.nlm.nih.gov/gene/?term=TEKT3
TEX14	testis expressed 14, intercellular bridge forming factor	https://www.ncbi.nlm.nih.gov/gene/?term=TEX14

TF	transferrin	https://www.ncbi.nlm.nih.gov/gene/?term=TF
TFCP2L1	transcription factor CP2 like 1	https://www.ncbi.nlm.nih.gov/gene/?term=TFCP2L1
TH	tyrosine hydroxylase	https://www.ncbi.nlm.nih.gov/gene/?term=TH
THBS2	thrombospondin 2	https://www.ncbi.nlm.nih.gov/gene/?term=THBS2
THEGL	theg spermatid protein like	https://www.ncbi.nlm.nih.gov/gene/?term=THEGL
THUMPD1	THUMP domain containing 1	https://www.ncbi.nlm.nih.gov/gene/?term=THUMPD1
THYN1	thymocyte nuclear protein 1	https://www.ncbi.nlm.nih.gov/gene/?term=THYN1
TLX3	T cell leukemia homeobox 3	https://www.ncbi.nlm.nih.gov/gene/?term=TLX3
TMEM114	transmembrane protein 114	https://www.ncbi.nlm.nih.gov/gene/?term=TMEM114
TMEM132D	transmembrane protein 132D	https://www.ncbi.nlm.nih.gov/gene/?term=TMEM132D
TMEM159	transmembrane protein 159	https://www.ncbi.nlm.nih.gov/gene/?term=TMEM159
TMEM182	transmembrane protein 182	https://www.ncbi.nlm.nih.gov/gene/?term=TMEM182
TMEM242	transmembrane protein 242	https://www.ncbi.nlm.nih.gov/gene/?term=TMEM242
TMEM59L	transmembrane protein 59 like	https://www.ncbi.nlm.nih.gov/gene/?term=TMEM59L
TMEM71	transmembrane protein 71	https://www.ncbi.nlm.nih.gov/gene/?term=TMEM71
TNFRSF9	TNF receptor superfamily member 9	https://www.ncbi.nlm.nih.gov/gene/?term=TNFRSF9
TNKS2	tankyrase 2	https://www.ncbi.nlm.nih.gov/gene/?term=TNKS2
TOE1	target of EGR1, exonuclease	https://www.ncbi.nlm.nih.gov/gene/?term=TOE1
TP53BP1	tumor protein p53 binding protein 1	https://www.ncbi.nlm.nih.gov/gene/?term=TP53BP1
TPH1	tryptophan hydroxylase 1	https://www.ncbi.nlm.nih.gov/gene/?term=TPH1
TRA2B	transformer 2 beta homolog	https://www.ncbi.nlm.nih.gov/gene/?term=TRA2B
TRAPPC8	trafficking protein particle complex 8	https://www.ncbi.nlm.nih.gov/gene/?term=TRAPPC8
TRBV25OR9-2	T cell receptor beta variable 25/OR9-2 (pseudogene)	https://www.ncbi.nlm.nih.gov/gene/?term=TRBV25OR9-2
TRDN	triadin	https://www.ncbi.nlm.nih.gov/gene/?term=TRDN
TRIM16	tripartite motif containing 16	https://www.ncbi.nlm.nih.gov/gene/?term=TRIM16
TRIM59	tripartite motif containing 59	https://www.ncbi.nlm.nih.gov/gene/?term=TRIM59
TRIO	trio Rho guanine nucleotide exchange factor	https://www.ncbi.nlm.nih.gov/gene/?term=TRIO

TRMT61A	tRNA methyltransferase 61A	https://www.ncbi.nlm.nih.gov/gene/?term=TRMT61A
TRPM1	transient receptor potential cation channel subfamily M member 1	https://www.ncbi.nlm.nih.gov/gene/?term=TRPM1
TRPV6	transient receptor potential cation channel subfamily V member 6	https://www.ncbi.nlm.nih.gov/gene/?term=TRPV6
TRY1	serine protease 1	https://www.ncbi.nlm.nih.gov/gene/?term=TRY1
TRY2	serine protease 2	https://www.ncbi.nlm.nih.gov/gene/?term=TRY2
TRY3	serine protease 3	https://www.ncbi.nlm.nih.gov/gene/?term=TRY3
TSTD2	thiosulfate sulfurtransferase like domain containing 2	https://www.ncbi.nlm.nih.gov/gene/?term=TSTD2
TTC21B	tetratricopeptide repeat domain 21B	https://www.ncbi.nlm.nih.gov/gene/?term=TTC21B
TTC39A	tetratricopeptide repeat domain 39A	https://www.ncbi.nlm.nih.gov/gene/?term=TTC39A
TUBGCP5	tubulin gamma complex associated protein 5	https://www.ncbi.nlm.nih.gov/gene/?term=TUBGCP5
TVP23B	trans-golgi network vesicle protein 23 homolog B	https://www.ncbi.nlm.nih.gov/gene/?term=TVP23B
TVP23C	trans-golgi network vesicle protein 23 homolog C	https://www.ncbi.nlm.nih.gov/gene/?term=TVP23C
TXN2	thioredoxin 2	https://www.ncbi.nlm.nih.gov/gene/?term=TXN2
TXNRD2	thioredoxin reductase 2	https://www.ncbi.nlm.nih.gov/gene/?term=TXNRD2
TYK2	tyrosine kinase 2	https://www.ncbi.nlm.nih.gov/gene/?term=TYK2
TYRP1	tyrosinase related protein 1	https://www.ncbi.nlm.nih.gov/gene/?term=TYRP1
U2	RNA, U2 small nuclear 1	https://www.ncbi.nlm.nih.gov/gene/?term=U2
UBE2B	ubiquitin conjugating enzyme E2 B	https://www.ncbi.nlm.nih.gov/gene/?term=UBE2B
UBXN10	UBX domain protein 10	https://www.ncbi.nlm.nih.gov/gene/?term=UBXN10
ULBP3	UL16 binding protein 3	https://www.ncbi.nlm.nih.gov/gene/?term=ULBP3
UMOD	uromodulin	https://www.ncbi.nlm.nih.gov/gene/?term=UMOD
UNC93A	unc-93 homolog A	https://www.ncbi.nlm.nih.gov/gene/?term=UNC93A
URB2	URB2 ribosome biogenesis homolog	https://www.ncbi.nlm.nih.gov/gene/?term=URB2
USP45	ubiquitin specific peptidase 45	https://www.ncbi.nlm.nih.gov/gene/?term=USP45
UVRAG	UV radiation resistance associated	https://www.ncbi.nlm.nih.gov/gene/?term=UVRAG
V1R	vomeronasal 1 receptor 51	https://www.ncbi.nlm.nih.gov/gene/?term=V1R
VDAC1	voltage dependent anion channel 1	https://www.ncbi.nlm.nih.gov/gene/?term=VDAC1

VEZT	vezatin, adherens junctions transmembrane protein	https://www.ncbi.nlm.nih.gov/gene/?term=VEZT
VPS26B	VPS26, retromer complex component B	https://www.ncbi.nlm.nih.gov/gene/?term=VPS26B
VRK1	VRK serine/threonine kinase 1	https://www.ncbi.nlm.nih.gov/gene/?term=VRK1
VWC2	von Willebrand factor C domain containing 2	https://www.ncbi.nlm.nih.gov/gene/?term=VWC2
VWDE	von Willebrand factor D and EGF domains	https://www.ncbi.nlm.nih.gov/gene/?term=VWDE
WASF3	WASP family member 3	https://www.ncbi.nlm.nih.gov/gene/?term=WASF3
WDR17	WD repeat domain 17	https://www.ncbi.nlm.nih.gov/gene/?term=WDR17
WDR62	WD repeat domain 62	https://www.ncbi.nlm.nih.gov/gene/?term=WDR62
WDR90	WD repeat domain 90	https://www.ncbi.nlm.nih.gov/gene/?term=WDR90
WFDC8	WAP four-disulfide core domain 8	https://www.ncbi.nlm.nih.gov/gene/?term=WFDC8
WIPF2	WAS/WASL interacting protein family member 2	https://www.ncbi.nlm.nih.gov/gene/?term=WIPF2
WIPF3	WAS/WASL interacting protein family member 3	https://www.ncbi.nlm.nih.gov/gene/?term=WIPF3
WNK2	WNK lysine deficient protein kinase 2	https://www.ncbi.nlm.nih.gov/gene/?term=WNK2
WWC1	WW and C2 domain containing 1	https://www.ncbi.nlm.nih.gov/gene/?term=WWC1
XCR1	X-C motif chemokine receptor 1	https://www.ncbi.nlm.nih.gov/gene/?term=XCR1
XPBP	X-C motif chemokine receptor 1	https://www.ncbi.nlm.nih.gov/gene/?term=XPBP
XPC	XPC complex subunit, DNA damage recognition and repair factor	https://www.ncbi.nlm.nih.gov/gene/?term=XPC
XPO6	exportin 6	https://www.ncbi.nlm.nih.gov/gene/?term=XPO6
YSK4	mitogen-activated protein kinase kinase kinase 19	https://www.ncbi.nlm.nih.gov/gene/?term=YSK4
YWHAH	tyrosine 3-monooxygenase/tryptophan 5-monooxygenase activation protein eta	https://www.ncbi.nlm.nih.gov/gene/?term=YWHAH
ZC3H3	zinc finger CCCH-type containing 3	https://www.ncbi.nlm.nih.gov/gene/?term=ZC3H3
ZFAT	zinc finger and AT-hook domain containing	https://www.ncbi.nlm.nih.gov/gene/?term=ZFAT
ZFYVE19	zinc finger FYVE-type containing 19	https://www.ncbi.nlm.nih.gov/gene/?term=ZFYVE19
ZMYND10	zinc finger MYND-type containing 10	https://www.ncbi.nlm.nih.gov/gene/?term=ZMYND10
ZNF236	zinc finger protein 236	https://www.ncbi.nlm.nih.gov/gene/?term=ZNF236
ZNF286A	zinc finger protein 286A	https://www.ncbi.nlm.nih.gov/gene/?term=ZNF286A
ZNF286B	zinc finger protein 286B (pseudogene)	https://www.ncbi.nlm.nih.gov/gene/?term=ZNF286B

ZNF436	zinc finger protein 436	https://www.ncbi.nlm.nih.gov/gene/?term=ZNF436
ZNF492	zinc finger protein 492	https://www.ncbi.nlm.nih.gov/gene/?term=ZNF492
ZNF516	zinc finger protein 516	https://www.ncbi.nlm.nih.gov/gene/?term=ZNF516
ZNF521	zinc finger protein 521	https://www.ncbi.nlm.nih.gov/gene/?term=ZNF521
ZNF555	zinc finger protein 555	https://www.ncbi.nlm.nih.gov/gene/?term=ZNF555
ZNF622	zinc finger protein 622	https://www.ncbi.nlm.nih.gov/gene/?term=ZNF622
ZNF679	zinc finger protein 679	https://www.ncbi.nlm.nih.gov/gene/?term=ZNF679
ZNF780B	zinc finger protein 780B	https://www.ncbi.nlm.nih.gov/gene/?term=ZNF780B
ZP2	zona pellucida glycoprotein 2	https://www.ncbi.nlm.nih.gov/gene/?term=ZP2
ZPBP	zona pellucida binding protein	https://www.ncbi.nlm.nih.gov/gene/?term=ZPBP
ZZEF1	zinc finger ZZ-type and EF-hand domain containing 1	https://www.ncbi.nlm.nih.gov/gene/?term=ZZEF1

Supplemental File 1b: List of candidate Neural Crest genes and their annotated terms (Benítez-Burraco et al., 2017)

Gene abbreviation	Gene name	NCBI link
ALX1	ALX homeobox 1	https://www.ncbi.nlm.nih.gov/gene/?term=ALX1
ALX3	ALX homeobox 3	https://www.ncbi.nlm.nih.gov/gene/?term=ALX3
ALX4	ALX homeobox 4	https://www.ncbi.nlm.nih.gov/gene/?term=ALX4
ASCL1	achaete-scute family bHLH transcription factor 1	https://www.ncbi.nlm.nih.gov/gene/?term=ASCL1
BDNF	brain derived neurotrophic factor	https://www.ncbi.nlm.nih.gov/gene/?term=BDNF
BMP2	bone morphogenetic protein 2	https://www.ncbi.nlm.nih.gov/gene/?term=BMP2
BMP4	bone morphogenetic protein 4	https://www.ncbi.nlm.nih.gov/gene/?term=BMP4
BMP7	bone morphogenetic protein 7	https://www.ncbi.nlm.nih.gov/gene/?term=BMP7
CAD7	cadherin 7	https://www.ncbi.nlm.nih.gov/gene/?term=CAD7
CDH2	cadherin 2	https://www.ncbi.nlm.nih.gov/gene/?term=CDH2
CDH6	cadherin 6	https://www.ncbi.nlm.nih.gov/gene/?term=CDH6
CMYC	MYC proto-oncogene, bHLH transcription factoR	https://www.ncbi.nlm.nih.gov/gene/?term=CMYC
COL1A2	collagen type I alpha 2 chain	https://www.ncbi.nlm.nih.gov/gene/?term=COL1A2
COL2A1	collagen type II alpha 1 chain	https://www.ncbi.nlm.nih.gov/gene/?term=COL2A1
CRKL	CRK like proto-oncogene, adaptor protein	https://www.ncbi.nlm.nih.gov/gene/?term=CRKL
DCT	dopachrome tautomerase	https://www.ncbi.nlm.nih.gov/gene/?term=DCT
DLX5	distal-less homeobox 5	https://www.ncbi.nlm.nih.gov/gene/?term=DLX5
DLX6	distal-less homeobox 6	https://www.ncbi.nlm.nih.gov/gene/?term=DLX6
EDN1	endothelin 1	https://www.ncbi.nlm.nih.gov/gene/?term=EDN1
EDN3	endothelin 3	https://www.ncbi.nlm.nih.gov/gene/?term=EDN3
EDNRA	endothelin receptor type A	https://www.ncbi.nlm.nih.gov/gene/?term=EDNRA
EDNRB	endothelin receptor type B	https://www.ncbi.nlm.nih.gov/gene/?term=EDNRB
EFNB1	ephrin B1	https://www.ncbi.nlm.nih.gov/gene/?term=EFNB1
EFNB2	ephrin B2	https://www.ncbi.nlm.nih.gov/gene/?term=EFNB2

ETS1	ETS proto-oncogene 1, transcription factor	https://www.ncbi.nlm.nih.gov/gene/?term=ETS1
FGF2	fibroblast growth factor 2	https://www.ncbi.nlm.nih.gov/gene/?term=FGF2
FGF8	fibroblast growth factor 8	https://www.ncbi.nlm.nih.gov/gene/?term=FGF8
FOXD3	forkhead box D3	https://www.ncbi.nlm.nih.gov/gene/?term=FOXD3
GBX2	gastrulation brain homeobox 2	https://www.ncbi.nlm.nih.gov/gene/?term=GBX2
GDNF	glial cell derived neurotrophic factor	https://www.ncbi.nlm.nih.gov/gene/?term=GDNF
GFAP	glial fibrillary acidic protein	https://www.ncbi.nlm.nih.gov/gene/?term=GFAP
GJB1	gap junction protein beta 1	https://www.ncbi.nlm.nih.gov/gene/?term=GJB1
GLI3	GLI family zinc finger 3	https://www.ncbi.nlm.nih.gov/gene/?term=GLI3
GSC	goosecoid homeobox	https://www.ncbi.nlm.nih.gov/gene/?term=GSC
HDAC	histone deacetylase 9	https://www.ncbi.nlm.nih.gov/gene/?term=HDAC
HES1	hes family bHLH transcription factor 1	https://www.ncbi.nlm.nih.gov/gene/?term=HES1
HES5	hes family bHLH transcription factor 5	https://www.ncbi.nlm.nih.gov/gene/?term=HES5
HOXA1	homeobox A1	https://www.ncbi.nlm.nih.gov/gene/?term=HOXA1
HOXA2	homeobox A2	https://www.ncbi.nlm.nih.gov/gene/?term=HOXA2
HOXA3	homeobox A3	https://www.ncbi.nlm.nih.gov/gene/?term=HOXA3
HOXB1	homeobox B1	https://www.ncbi.nlm.nih.gov/gene/?term=HOXB1
ID3	inhibitor of DNA binding 3, HLH protein	https://www.ncbi.nlm.nih.gov/gene/?term=ID3
ISL1	ISL LIM homeobox 1	https://www.ncbi.nlm.nih.gov/gene/?term=ISL1
ITGB1	integrin subunit beta 1	https://www.ncbi.nlm.nih.gov/gene/?term=ITGB1
KIF1B	kinesin family member 1B	https://www.ncbi.nlm.nih.gov/gene/?term=KIF1B
LHX1	LIM homeobox 1	https://www.ncbi.nlm.nih.gov/gene/?term=LHX1
LHX2	LIM homeobox 2	https://www.ncbi.nlm.nih.gov/gene/?term=LHX2
MASH1	achaete-scute family bHLH transcription factor 1	https://www.ncbi.nlm.nih.gov/gene/?term=MASH1
MAX	MYC associated factor X	https://www.ncbi.nlm.nih.gov/gene/?term=MAX
MITF	melanocyte inducing transcription factor	https://www.ncbi.nlm.nih.gov/gene/?term=MITF
MSX1	msh homeobox 1	https://www.ncbi.nlm.nih.gov/gene/?term=MSX1

MSX2	msh homeobox 2	https://www.ncbi.nlm.nih.gov/gene/?term=MSX2
NEUROD	neuronal differentiation 1	https://www.ncbi.nlm.nih.gov/gene/?term=NEUROD
NEUROG1	neurogenin 1	https://www.ncbi.nlm.nih.gov/gene/?term=NEUROG1
NF1	neurofibromin 1	https://www.ncbi.nlm.nih.gov/gene/?term=NF1
NFKB	nuclear factor kappa B subunit 1	https://www.ncbi.nlm.nih.gov/gene/?term=NFKB
NOTCH	notch receptor 1	https://www.ncbi.nlm.nih.gov/gene/?term=NOTCH
NRP1	neuropilin 1	https://www.ncbi.nlm.nih.gov/gene/?term=NRP1
NRP2	neuropilin 2	https://www.ncbi.nlm.nih.gov/gene/?term=NRP2
OLIG1	oligodendrocyte transcription factor 1	https://www.ncbi.nlm.nih.gov/gene/?term=OLIG1
OLIG2	oligodendrocyte transcription factor 2	https://www.ncbi.nlm.nih.gov/gene/?term=OLIG2
PAX3	paired box 3	https://www.ncbi.nlm.nih.gov/gene/?term=PAX3
PAX7	paired box 7	https://www.ncbi.nlm.nih.gov/gene/?term=PAX7
PHOX2B	paired like homeobox 2B	https://www.ncbi.nlm.nih.gov/gene/?term=PHOX2B
PMP22	peroxisomal membrane protein 2	https://www.ncbi.nlm.nih.gov/gene/?term=PMP22
POMT1	protein O-mannosyltransferase 1	https://www.ncbi.nlm.nih.gov/gene/?term=POMT1
RET	ret proto-oncogene	https://www.ncbi.nlm.nih.gov/gene/?term=RET
RHOB	ras homolog family member B	https://www.ncbi.nlm.nih.gov/gene/?term=RHOB
ROBO1	roundabout guidance receptor 1	https://www.ncbi.nlm.nih.gov/gene/?term=ROBO1
ROBO2	roundabout guidance receptor 2	https://www.ncbi.nlm.nih.gov/gene/?term=ROBO2
SDHB	succinate dehydrogenase complex iron sulfur subunit B	https://www.ncbi.nlm.nih.gov/gene/?term=SDHB
SDHD	succinate dehydrogenase complex subunit D	https://www.ncbi.nlm.nih.gov/gene/?term=SDHD
SNAIL1	snail family transcriptional repressor 1	https://www.ncbi.nlm.nih.gov/gene/?term=SNAIL1
SNAIL2	snail family transcriptional repressor 2	https://www.ncbi.nlm.nih.gov/gene/?term=SNAIL2
SOX10	SRY-box transcription factor 10	https://www.ncbi.nlm.nih.gov/gene/?term=SOX10
SOX5	SRY-box transcription factor 5	https://www.ncbi.nlm.nih.gov/gene/?term=SOX5
SOX9	SRY-box transcription factor 9	https://www.ncbi.nlm.nih.gov/gene/?term=SOX9
TBX1	T-box transcription factor 1	https://www.ncbi.nlm.nih.gov/gene/?term=TBX1

TFAP2A	transcription factor AP-2 alpha	https://www.ncbi.nlm.nih.gov/gene/?term=TFAP2A
TMEM127	transmembrane protein 127	https://www.ncbi.nlm.nih.gov/gene/?term=TMEM127
TWIST	twist family bHLH transcription factor 1	https://www.ncbi.nlm.nih.gov/gene/?term=TWIST
VHL	von Hippel-Lindau tumor suppressor	https://www.ncbi.nlm.nih.gov/gene/?term=VHL
WNT1	Wnt family member 1	https://www.ncbi.nlm.nih.gov/gene/?term=WNT1
WNT3a	Wnt family member 3A	https://www.ncbi.nlm.nih.gov/gene/?term=WNT3a
WNT6	Wnt family member 6	https://www.ncbi.nlm.nih.gov/gene/?term=WNT6
WNT7B	Wnt family member 7B	https://www.ncbi.nlm.nih.gov/gene/?term=WNT7B
WNT8	dickkopf WNT signaling pathway inhibitor 1	https://www.ncbi.nlm.nih.gov/gene/?term=WNT8
ZEB2	zinc finger E-box binding homeobox 2	https://www.ncbi.nlm.nih.gov/gene/?term=ZEB2
ZIC1	Zic family member 1	https://www.ncbi.nlm.nih.gov/gene/?term=ZIC1

Supplemental File 2: List of HUGO gene IDs for genes tested for positive selection

TNMD
C1orf112
NIPAL3
ENPP4
CFTR
CYP51A1
KRIT1
LAP3
HS3ST1
WNT16
HECW1
MAD1L1
LASP1
SNX11
RBM5
SARM1
KDM1A
RBM6
CAMKK1
RECQL
VPS50
HSPB6
ARHGAP33
NDUFAB1
PDK4
ZMYND10
ABCB5
CDC27
CALCR
FBXL3
ITGAL
PDK2
ITGA3
ZFX
LAMP2
ITGA2B
ASB4
GDE1
REX1BD
ABCC8
CACNG3
TMEM132A
TAC1
ZNF263
SPATA20
CACNA1G
TNFRSF12A
DLX6
MAP3K9
RALA
BAIAP2L1
KDM7A

ETV1
AGK
ALDH3B1
TTC22
PHTF2
USH1C
GGCT
DBF4
TBXA2R
IFRD1
COX10
GTF2IRD1
PAF1
VPS41
ARHGAP44
ELAC2
SCIN
CDKL3
UPP2
MARK4
CCDC124
PAFAH1B1
KIAA0100
SLC13A2
GAS7
TRAPPC6A
MATK
CD79B
ST7L
PAX6
RPUSD1
RHBDF1
CACNA2D2
BAIAP3
TSR3
PIGQ
CRAMP1
DNAJC11
FMO3
MYLIP
PSMB1
SYN1
JARID2
CDKL5
CAMK1G
NADK
TFAP2B
TFAP2D
ADAM22
SYPL1
CYB561
SPAG9
CELSR3
AASS
PLEKHG6
SS18L2

MGST1
CRY1
PGLYRP1
NFIX
ST3GAL1
MMP25
MAPK8IP2
MED24
RHOBTB2
HEATR5B
SEC62
RPS20
CSDE1
UBE3C
REV3L
MASP2
IYD
FAM76A
TRAF3IP3
POMT2
VTA1
MLXIPL
BAZ1B
RANBP9
SPRTN
EEF1AKNMT
ZNF207
UQCRC1
STARD3NL
CD9
HHATL
NCAPD2
IFFO1
GIPR
PHF7
SEMA3G
NISCH
STAB1
FUZ
LRRC23
BTK
HFE
SCMH1
FYN
HIVEP2
FMO1
ELOA
LYPLA2
CLCN6
MRC2
NME1-NME2
SLC6A7
TSPAN9
APBA3
MKS1
ABHD5

AKAP8L
MBTD1
UTP18
RNF216
PTBP1
DPF1
SYT7
LARS2
PIK3C2A
PLAUR
ANLN
WIZ
RABGAP1
DCN
QPCTL
PPP5C
MAP4K3
ZBTB32
TYROBP
ERCC1
SEMA3B
MBTPS2
PRICKLE3
LTF
EXTL3
NR1H4
ELOVL5
CALCOCO1
MAP4K5
SLC7A14
CLDN11
SLC25A39
MVP
NUB1
PGM3
RWDD2A
CLK1
POLR3B
ANGEL1
RNF14
DDX11
GPRC5A
MAMLD1
TACC3
UFL1
CAPN1
ACP3
SLC30A9
MTMR11
COX15
CCDC88C
YAF2
ZMYND11
WAS
DPEP1
NPC1L1

XYLT2
STMN4
ISL1
CHDH
IL20RA
CLCA1
GLT8D1
ATP2C1
IGF1
RALBP1
RUFY3
CNTN1
SLC11A1
WWTR1
AGPS
STEEP1
TTC27
VSIG2
PHLDB1
MARCO
CYP24A1
PRDM11
SYT13
SNAI2
HGF
ZRANB1
NCDN
ADGRA2
ZFP64
MNAT1
SAMD4A
RUNX3
MRE11
PLEKHB1
SERPINB1
SLC7A9
SPAST
OSBPL5
AQR
CPS1
C8B
FHL1
RTF2
SLC45A4
RNF10
ZDHHC6
GRAMD1B
RB1CC1
ERP44
ALAS1
AKAP11
GLRX2
SNAPC1
DERA
STRAP
ABCC2

DEF6
PLEKHO1
GCLM
UBR2
DEPDC1
CCDC28A
RRAGD
HSF2
PHF20
NR1H3
NCAPH2
TOMM34
SEC63
KPNA6
VIM
FAS
CD44
KCNG1
AGPAT4
SLAMF7
MIPEP
PRKCH
INSRR
B4GALT7
SH2D2A
VRK2
TNFRSF1B
VEZT
BRD9
SNX1
TBPL1
BCLAF1
SLC39A9
ANK1
IBSP
TFB1M
RABEP1
HMGB3
NUP160
BAK1
MUSK
IKZF2
GRN
FAM13B
ARHGAP31
CENPQ
SARS1
RANBP3
ARID4A
EIPR1
PNPLA6
IFT88
ALG1
ZCCHC8
CHPF2
LRRC7

FUT8
UBA6
GAB2
ATP6V0A1
PIAS1
SLC4A7
MAP2K3
EFCAB1
TMSB10
ASTE1
RNF19A
PEX3
GABARAPL2
MYOC
SH3YL1
FAM136A
VCL
DEPDC1B
NSMAF
ADSS2
STAP1
TIMP2
RFC1
TBC1D23
CUL3
OTC
CYP46A1
ZZZ3
SLC18A1
USP2
CASR
FLT4
FBXO42
MFAP3
MRI1
METTL1
HOXC8
AGA
PI4K2B
MAT2B
EDC4
CLEC16A
MSR1
CDH1
MTREX
DNAH5
RIPOR1
C6
RAI14
SOX30
PNKP
BEST2
PHLPP2
SPDL1
STAU2
SLC66A1

CTNS
PHF23
INPP4A
RAB27B
PSMA4
LSG1
PARP3
TNC
THAP3
RIPOR3
TDP1
AIFM2
MED17
RETSAT
CAPG
AP2S1
ZPBP
BARX2
DCUN1D1
JADE2
ZIC2
LCP2
TRIT1
GUCA2B
CUL7
CTNNA1
PHKA2
CNTLN
HSPA5
DSG2
GEMIN8
GPM6B
PREX2
WDR37
YTHDC2
ATP6V1H
POLR2B
FAM214A
ARAP2
TPR
CP
DTNBP1
XK
C12orf4
WWC3
ARHGAP6
FAM184B
GOPC
ROS1
USP28
TSPAN17
ZNF800
TNFRSF17
SNX29
MRPS10
RSF1

VPS13D
FAM120A
R3HDM1
COL9A2
KITLG
ERCC8
H6PD
VAMP3
EPN3
LTBP1
ELN
RFC2
NEDD4L
FOXP3
PPP1R3F
HEXB
PTCD2
NEXMIF
JKAMP
DKK3
NFE2L3
LIMA1
LETMD1
PTGER3
COL23A1
FAM160A2
HERPUD1
HOMER3
RAD51
PIK3CB
CYBA
THOC3
HEBP2
PLEKHA5
PRSS8
SIKE1
RRP12
MSMO1
TTC17
ALX4
FSTL4
FOXN3
METTL24
MRTO4
NNAT
USE1
AP5M1
ANAPC4
KCNQ1
TRAPPC3
THRAP3
PHPT1
ENTPD2
ARID4B
PTPRN
KIF1B

PLEKHH1
SPO11
CHRDL2
FAM168A
RELT
GALC
NOP58
SZRD1
KCNH2
CUL1
FAM114A2
TAB2
GINM1
EIF2AK2
MCOLN3
CCDC85A
PUM2
MRPL43
ITIH4
ITIH1
HPF1
ZFR
NPFFR2
PHF21B
TRAF1
RC3H2
IL17RB
TRAF3IP2
DCBLD2
SOAT1
PKP2
MSH4
F7
GDI2
PRDM1
ATG5
TMCC3
PITHD1
ATP11B
LAMC2
PPP1R12A
CROCC
ZC3H11A
RIOK2
YIPF1
NDC1
DGKG
FLYWCH1
TBXAS1
PARP12
ALDH18A1
GATB
MXD1
CDK17
DNAJC25
SLC2A3

PSD
YBX3
STYK1
WNK1
CCAR1
OGFR
GNA15
CREB3L3
PIGV
PTPRU
SNRNP40
RIMBP2
COL11A1
QSER1
MPC1
ACAA1
BCAT1
HDAC7
LZTS1
PRDM6
WNT8A
SPAG4
NCKAP1
MRPS35
GUCY1B1
SFSWAP
TNK2
MON2
CDH3
GPBP1
DGAT2
CS
MRPS24
ELMO2
WAPL
VMP1
APPBP2
POLD1
SEZ6
EIF4B
BICRA
SPHK2
CA11
ISOC2
U2AF2
EPN1
MED29
ZNF275
MTMR1
GPC1
ADCK1
HAGH
RNF4
CASP8
INTS13
DLX3

SPA17
CCN5
DMRT3
ST3GAL6
ATP2C2
NGFR
CDON
TAF2
TNPO3
RFXANK
TMEM161A
LPAR2
CTSA
SUGP2
SLC12A2
SNX24
CNN2
SNCAIP
DDX20
BCAS1
POU1F1
PMS1
HMG20B
TAF11
ANKS1A
AP3D1
ZNF76
SLC9A3R2
NTHL1
UHRF1BP1
OAT
WDR3
PKN2
WDR18
TRAM2
NTN1
GLP2R
MCM10
DGKA
ERBB3
KARS1
ADAT1
PDIA5
TBC1D22B
NDUFB4
SPEN
MYLK
ZC3H15
MAP2K4
PACC1
SNAP91
SLK
CYB5R4
GSTO2
TLE2
ASB1

FAM107B
TBC1D1
CDK13
MTHFD2
FOXJ2
PDE4A
PPP2R5A
ELAVL1
TIE1
SMARCD1
KDM4A
NFYC
ZMYND12
SLC9A3
NGEF
ELOVL1
SPI1
MPPED2
CLDN18
ZBTB11
ATXN3
GOLGA5
LRRC40
ISOC1
EML1
TRMT11
THUMPD1
MSANTD3
ATG2B
ZFAT
MTFR1
STAG3
FECH
MYO9A
IDI1
KLF6
PLPP1
NEO1
TRAM1
TNFRSF1A
CACNB1
STOML1
PKM
DHX29
DNTTIP2
METTL22
TP53BP1
RRP15
RHOA
DHX8
PITX1
ADGRF5
SDK2
ADAM7
NUP133
NUCKS1

VPS35
DNAJA2
BCL3
FUNDC1
RORA
TGFBR3
HES2
ATP1B3
NEDD4
PIGB
MAPK6
GNB5
HDHD5
UFD1
GUCY2C
ELP1
NUCB2
PFN2
PTPN3
SPTB
DAPP1
FGF10
SLC44A1
TMEM260
SMG6
EXOC5
FGF22
FSTL3
DGCR2
RNF126
MNT
JMJD6
POLB
WIPI1
FRMPD1
GBA2
NDST1
ASNS
AP3M2
CNGB1
ST6GALNAC2
CHAT
PABPC1
TESK2
CFAP20
CSNK2A2
PTPN21
EIF2B3
CDC42
OSBPL3
EPHA8
SLC12A3
RAD18
ATP2B1
NCK2
MAP4K4

MGAT4A
RPL31
WDR1
SNX13
ARHGAP10
ING3
VASH1
LMCD1
BUD23
SEL1L
TRIP13
ATP6AP1
TCF3
TRIB2
DAZAP1
CYP2W1
MCM2
PANX2
SELENOO
TP63
LLGL2
NLE1
SDHA
SMARCE1
FNDC8
KDM5A
ADAM11
PPP2R3A
FERMT2
ABCB11
DHRS9
PTGS2
IGF2BP2
MAP3K13
ST6GAL1
TBX21
PICALM
GLI2
CLASP1
NOTCH3
CLNS1A
TEAD2
CDHR2
SNCB
TSG101
NCBP3
ATP2A3
CA12
MGLL
NTN4
BCS1L
NUAK1
DPP8
ZNF532
LMAN1
HACD3

ZZEF1
NOX3
ENO1
SLC12A1
MYDGF
ANO8
TUBE1
ARHGEF10L
TXK
WSCD2
KCNQ2
TACR2
ACTR6
TIPIN
SRI
NUP37
SEMA3A
SEMA3C
TTC38
ACAT1
GRAMD4
WNT8B
ZNF638
SLC25A40
TIMM21
ADD2
RASAL2
VPS9D1
MARK3
SLC25A3
FNDC3B
FOSL2
CACNG4
FRYL
TMEM131
FSCN1
MOCOS
PLD1
RAB7A
SEC31B
SART3
ARHGAP15
MKRN2
MCM6
REXO2
RBM7
KLHL20
RGS11
SLC46A1
SPAG5
ANKRD13A
GPATCH1
NT5C2
MCAM
STXBP2
MAP2K7

CTTNBP2
UBE2T
PPP1R12B
DNAJC10
GTF3C1
SPAG6
EXOSC5
DYNC1I2
LRCH4
FAM76B
CAPZB
GPR137B
JADE1
SLC25A43
ITGA8
CST7
MAP2
PIAS2
ARAF
MCCC1
ACER3
UBE2K
PIK3C3
N4BP2
HOXA9
EDN1
MLLT10
ZCWPW1
ADCYAP1R1
FGF20
P2RY10
NRDC
VDAC3
PCM1
TNRC6C
CBFA2T2
BRINP1
ITCH
PKD2L2
TP53INP2
SDF4
MYH7B
BPIFB2
TP73
TOLLIP
RUNX1T1
CDH17
THOC1
FKBP7
OSBPL6
SLC1A3
XRCC5
LXN
MKNK1
TNS1
REXO1

SAR1A
CDC14A
RAPGEF3
SENP1
DUSP13
CIC
PAFAH1B3
OPHN1
AFM
KIF22
CARMIL1
PGM1
DDX1
EPB41L2
RIMS1
MOXD1
STX7
KEAP1
DCT
SLC35C2
SCTR
RFX3
RIF1
RAB21
RDH8
SESN1
DNAAF6
COL5A3
SRCAP
PUM3
CPB2
CHRNA3
KCNN2
CNOT4
CPOX
CLDND1
HSP90AA1
RBL1
DLGAP4
IGSF9B
NDC80
AP4E1
RSBN1
MAGI3
AFP
TCF7
OSTM1
IMPG2
PCNP
EXD2
ARG2
MEF2C
PKP1
UBA5
DELE1
SLC13A1

HSPB11
ATP8B1
IL12RB2
SMARCD3
WDR70
FYB1
MPP4
STRADB
BZW1
CCNT2
FAM135A
EPB41L3
TRAF5
MRPL22
GEMIN5
NFE2L1
SEMA5B
ITGB5
XPO1
RNF13
DOP1A
LYRM2
BCKDHB
KAT6A
TUT7
ULK2
GRHL2
TNPO1
PLOD1
ITGAE
DIS3
PIBF1
TDRD3
NUFIP1
EPYC
CYLD
SLC27A5
ZNF446
RPS5
FAT1
CHMP2B
SMAP2
PPIE
ZMPSTE24
STARD7
NOA1
REST
HAL
SSH1
APLP2
FAM234B
SLCO1A2
WBP11
EIF3I
NKAIN1
COL16A1

TXLNA
NCOA1
AGBL5
EFR3B
KIF3C
RAB10
MAPRE3
CAD
BCORL1
ATRX
MYNN
MECOM
SCAMP1
PREP
HACE1
SEH1L
WDR47
WDFY1
MAP3K4
IGSF9
AKR1B1
WNT11
MTIF2
DDHD2
TTC39A
EPS15
ORC1
MGST2
CHERP
POMGNT1
RAD54L
MAST2
DNAJA1
B4GALT1
CHMP5
NFX1
AQP6
DIMT1
IPO11
EIF2AK1
EPDR1
SNX10
SEPHS1
MRPL28
ITPKC
FAT2
RBM22
HUWE1
ZW10
ALG9
MYBPC2
NOX4
ACOX3
HSD17B14
TRIP6
ACHE

FTL
SRRT
BAX
NLK
PIGS
ATXN7L3
PGS1
PSMC5
UIMC1
MMP2
MT3
LPCAT2
OGFOD1
SH3BP2
NOP14
ADD1
L2HGDH
TXNDC16
RTRAF
NID2
GMCL1
SF3B2
KLHL42
GNAS
DNM1L
PTHLH
PHACTR3
ERGIC2
TFAP2C
AURKA
CASS4
PIR
AAMDC
RFX2
SULT2B1
ALG6
PTPN4
DDX18
KHSRP
EDEM2
DNMT3B
REM1
TPX2
PDRG1
EPB41L1
SLC15A1
DOCK9
ANKRD10
TGDS
DOCK3
C3orf18
COQ9
TMEM40
KIF9
ARHGAP28
PPP1R13B

ATRN
SMOX
FKBP1A
NSFL1C
SLC4A11
C20orf194
CPXM1
LZTS3
XRN2
DYNLL1
TESC
SNX5
RPL6
MAPKAPK5
P2RX7
ESF1
RBBP9
ANAPC5
SLC23A2
SLC8B1
TMEM230
DZANK1
KDM2B
CFAP61
LHX5
TASP1
OAS1
GCN1
RPLP0
PXN
SIRT4
RPH3A
KIF16B
TRMT6
CHGB
PEBP1
TBX5
BRAP
ERP29
NOS1
FUS
IGBP1
GRAMD1A
HEPH
KCNH4
GANAB
GMIP
RBM41
BIRC5
LAG3
MLF2
ZBTB25
NECAP1
ARHGAP4
ANKRD24
DHX32

RCOR1
GPATCH2L
LTBP4
BLVRB
SLC9A1
SPTLC1
PAPOLA
CCNK
PCBP4
RGS1
YPEL3
MRPS33
NDUFB2
NUDC
MAEA
STRN4
IRAK3
LYZ
SI
MUL1
TFAP4
PDCD7
SPG21
DNAJB11
P3H2
THPO
CHRD
GNPTG
GOLGA3
PABPC4
MCOLN1
USP48
EFNB1
PDPR
AARS1
GLG1
KIF4A
PLEKHG2
NAT14
PITPNM2
EXOC1
RBM27
POU4F3
OSBPL8
DTX2
PUS7
NRCAM
LAMB1
SLC26A4
DLD
WDR7
TXNL1
CMTM6
FH
SEL1L3
TF

CDV3
ORC6
ZFHX4
CPA1
ZC3HC1
CCDC80
CMA1
PPP2R3C
HAUS4
JPH4
CEBPE
SLC7A8
OSGEP
SCFD1
G2E3
HECTD1
HNRNPC
SUPT16H
TOX4
GEMIN2
DAZL
SEMA6A
TRPM7
TYRO3
WDR76
CAPN3
SNAP23
TBX15
PHGDH
COL9A3
MYL6
TEKT2
TGFB2
GPATCH2
NUP50
CDC45
COMT
ECHDC1
SEC22C
XYLB
HDAC6
GABRP
CDC6
UPRT
CBX5
FMO2
MSH2
MAP3K1
DHPS
TMEM38B
PSMD5
PTGS1
NUP188
CRAT
SH2D3C
NANS

TBC1D2
WHRN
PDE6C
CWF19L1
SEMA4G
BTAF1
IKZF5
BLNK
CYP26A1
TDRD1
SORBS1
CRTAC1
BAMBI
IL11
MYO3A
WAC
CREM
NUBP2
TREM2
KCNK16
FKBP5
BRPF3
PGC
TMEM14A
EFHC1
HSP90AB1
CDC5L
ITPR3
ZNF184
DSP
SIRT1
HNRNPH3
IFT74
JAK2
ABL1
ACOT7
SH3GLB1
CDC7
SYDE2
PCSK5
SCD
TMED1
ABLIM1
ERMP1
NRP1
PALMD
TSPAN15
MACROH2A2
OCEL1
KCNK6
PSMD8
FBXL19
HSD3B7
SETD1A
CIRBP
ATP5F1D

CBARP
IGFALS
HNRNPM
MARCHF2
NDUFB7
TECR
TIMM13
CDC34
MTAP
CEP170B
POLR2E
RASSF7
GADD45B
PALM
MKNK2
ARVCF
TRMT2A
RANBP1
ZDHHC8
KLHL22
MED15
SNAP29
CRKL
LZTR1
CECR2
P2RX6
BCL2L13
CABIN1
TBC1D10A
SUSD2
SF3A1
GGT5
RNF215
SPECC1L
PPIL2
UPB1
SNRPD3
PES1
PPM1F
SLC35E4
TOP3B
CRYBB3
ESS2
MFNG
CARD10
SLC25A1
PLA2G3
GGA1
HIRA
SH3BP1
LGALS1
HPS4
PIK3IP1
SRRD
PATZ1
TFIP11

GCAT
CRYBB1
ANKRD54
SNU13
MICALL1
POLR2F
SOX10
CCDC134
DEPDC5
PICK1
TTC28
CENPM
KDELR3
DMC1
HSCB
CBY1
TOMM22
RSPH14
XBP1
RTCB
JOSD1
FBXO7
GTPBP1
POLDIP3
RAB36
TIMP3
SBF1
SUN2
CYB5R3
DNAL4
C22orf31
MIOX
LMF2
RHBDD3
PACSIN2
TTLL1
RASL10A
HMGXB4
TOM1
NEFH
CHKB
HMOX1
MCAT
THOC5
MCM5
ARSA
TSPO
TTLL12
CBX7
PDGFB
CABP7
RPL3
ZMAT5
RBFOX2
SYNGR1
TAB1

ASCC2
MTMR3
MIEF1
SAMM50
TXN2
FOXRED2
GRAP2
EIF3D
TNRC6B
SGSM3
IFT27
KIAA0930
SLC25A17
UPK3A
FAM118A
KCTD17
ST13
IL2RB
RBX1
EP300
L3MBTL2
CHADL
RANGAP1
ZC3H7B
PHF5A
ACO2
POLR3H
TRMU
PMM1
DESI1
CERK
BRD1
ZBED4
MLC1
HDAC10
KCNK10
ABHD4
KHNYN
FKBP3
SDR39U1
PRMT5
COCH
POLE2
VCPKMT
CDKL1
NIN
TRIM9
PSMC6
GNPNAT1
DDHD1
CDKN3
CNIH1
CGRRF1
ATP6V1D
CCDC198
PLEK2

PIGH
PSMA3
VTI1B
TIMM9
GSTZ1
TMED8
AHSA1
DAAM1
LGMN
ALKBH1
SNW1
PPM1A
SIX4
GALNT16
CEP128
ERH
HIF1A
SUSD6
SRSF5
SLC10A1
EIF5
DICER1
ZFYVE21
MTHFD1
ZC3H14
TELO2
PCNX1
BDKRB1
GSKIP
VRK1
PSMC1
RPS6KA5
PPP4R3A
C14orf93
PSMB5
YY1
ACIN1
CCNB1IP1
TRIP11
APEX1
PABPN1
EFS
ARHGAP5
CINP
SRP54
CHD8
PSMA6
NFKBIA
BRMS1L
PNN
PLTP
PCIF1
GSS
MMP9
TRPC4AP
ABHD12

PROCR
GINS1
CD40
UQCC1
ZMYND8
IFT52
MYBL2
R3HDML
HNF4A
NDRG3
SLA2
RAB5IF
NFATC2
RIMS4
STK4
SALL4
ADNP
PFDN4
DOK5
CSTF1
RAE1
TPD52L2
DNAJC5
NELFCD
CTSZ
PRPF6
PRELID3B
HRH3
MTG2
PSMA7
SLCO4A1
NTSR1
MRGBP
TCFL5
GID8
SLC17A9
BIRC7
ARFGAP1
AVP
CHRNA4
GMEB2
C20orf27
SPEF1
CDC25B
ISM1
RNF24
ARFRP1
NDUFAF5
SEL1L2
TRIB3
RASSF2
CSNK2A1
SLC52A3
RSPO4
CDS2
HM13

SNPH
MYLK2
FERMT1
HAO1
PDYN
CCM2L
PLCB4
MYL9
TM9SF4
TLDC2
POFUT1
SAMHD1
PAK5
KIF3B
NOP56
MANBAL
IDH3B
MAPRE1
JAG1
CDK5RAP1
SNTA1
TTI1
E2F1
RPRD1B
PXMP4
CHMP4B
SLC32A1
CST3
ASIP
ACTR5
WFDC2
AHCY
PPP1R16B
FAM83D
DHX35
DNTTIP1
MAP1LC3A
SYNDIG1
PIGU
ACOT8
APMAP
CELF4
ADNP2
RBFA
USP14
VAPA
METTL4
LPIN2
SMCHD1
MYOM1
CEP76
ST8SIA5
RNMT
SMAD7
LIPG
RNF125

ANKRD12
NOL4
POLI
MIB1
RBBP8
RIOK3
PSMD10
ATG4A
PGRMC1
POLA1
NXT2
GUCY2F
ATP1B4
PRPS2
TLR8
MOSPD1
CHRDL1
WDR13
SUV39H1
SRPX
XIAP
STAG2
ATP11C
MCF2
F9
CCDC22
SYP
PLP2
BMX
RENBP
ELF4
ASB9
ZC3H12B
KCND1
FMR1
PIM2
SLC35A2
PQBP1
RS1
PCSK1N
TAZ
GATA1
SMS
PHEX
UBL4A
CD99L2
EEA1
RP2
JADE3
CDK16
USP11
BRS3
HTATSF1
VGLL1
CD40LG
TIMP1

FGD1
PIN4
PORCN
MAGED2
RBM3
SRPX2
SYTL4
ZDHHC15
DRP2
PBDC1
GLA
ARMCX3
NALCN
HTR2A
NDFIP2
TNFSF13B
FNDC3A
CDADC1
KLF5
ACP5
DNAJC3
UGGT2
SGCG
SUPT20H
KPNA3
FLT1
RGCC
VWA8
DGKH
INTS6
ACOD1
MEDAG
TSC22D1
CLN5
OLFM4
MGRN1
ZNF629
TRADD
HSF4
CORO1A
MAPK3
GDPD3
ELMO3
MT4
PHKB
LYRM1
NUTF2
NUP93
TSNAXIP1
NFAT5
LONP2
N4BP1
CBLN1
PLLP
ZNF423
DHODH

CCL17
CTCF
POLR2C
PARD6A
ZNF821
MMP15
USB1
CYB5B
CCDC113
PRSS54
NDRG4
PSMD7
SETD6
SLC38A7
VAC14
HAS3
TANGO6
COG4
SMPD3
SLC7A6OS
PLA2G15
ESRP2
FA2H
WDR59
MON1B
CMC2
AXIN1
HCFC1R1
NPRL3
MLYCD
MPG
NECAB2
HSDL1
TAF1C
SEC14L5
COTL1
USP10
CRISPLD2
TSC2
NME4
LMF1
FOXF1
CIAO3
MTHFSD
CLCN7
ANTKMT
SLC7A5
METRN
FBXO31
STUB1
RHBDL1
NUBP1
UBE2I
CRYM
CAPN15
ZNF174

CLUAP1
UBFD1
EARS2
ELOB
GGA2
AQP8
CPPED1
USP31
SALL1
TOX3
RBL2
QPRT
PYCARD
RPGRIP1L
MAZ
STX4
CDIPT
BCKDK
KAT8
SYT17
TMC5
CCP110
VPS35L
SLC6A2
RNF40
KNOP1
AQP9
AAGAB
IQCH
LACTB
CORO2B
CSK
TRIP4
RASL12
AP3B2
IGDCC4
RAB11A
CTSH
TTC23
FAH
CEMIP
RPAP1
HOMER2
TMEM87A
ZNF106
DTWD1
GABPB1
BMF
DMXL2
SCG3
DNAJC17
EIF3J
SPG11
RHOV
VPS18
OIP5

SLC30A4
BLOC1S6
MYEF2
SGK3
PDGFRL
ZDHHC2
BRF2
TRIM35
ZFAND1
RP1
CA2
FZD3
INTS9
RIPK2
NBN
CPQ
DECR1
CALB1
BPNT2
SFRP1
UBE2W
POP1
NIPAL2
IKBKB
PLAT
JPH1
GDAP1
RAB2A
EIF3E
EMC2
ESRP1
CCN4
NDRG1
ZC2HC1A
STMN2
ARMC1
TRPS1
CHRAC1
SNX16
UBR5
ANXA13
SQLE
SH2D4A
INTS10
ERI1
SLC39A14
LEPROTL1
DCTN6
R3HCC1
UBXN8
PPP2CB
NEFM
KLHDC4
MCM4
KCTD9
FGL1

ASAH1
BNIP3L
KCNN4
NUCB1
GYS1
SNRNP70
CLPTM1
RELB
CLASRP
LIN7B
PPP1R37
FCGRT
PIH1D1
ARHGEF18
PPP1R13L
PEX11G
ERCC2
DOT1L
RNASEH2A
CD37
SF3A2
AMH
DKKL1
LYL1
OAZ1
TRMT1
DMPK
RSPH6A
TBC1D17
IL4I1
CCDC130
PTOV1
SGTA
MED25
SNAPC2
C19orf53
TIMM44
CCDC61
TNNT1
VRK3
FAM32A
PPP6R1
MED26
OLFM2
RASAL3
EPHX3
ILVBL
SYDE1
SLC1A6
POP4
CCNE1
URI1
PDCD5
ANKRD27
RPS16
TIMM50

GPI
PLD3
PRX
PIAS4
NUMBL
EBI3
YJU2
SHD
TBCB
FSD1
POLR2I
OVOL3
CLIP3
TJP3
APLP1
CACTIN
CCDC9
HNRNPUL1
FZR1
BBC3
TGFB1
DENND3
DMAC2
PLIN3
MRPL4
CD79A
LIM2
ICAM4
RPS19
NOP53
NKG7
ICAM5
ETFB
CRX
BABAM1
TYK2
CDC37
NAPA
RABAC1
MEIS3
CNFN
MEGF8
GRWD1
GRIN2D
SYNGR4
CLEC11A
LIG1
HAS1
DBP
TMEM205
CAPS
PLPPR2
RASIP1
FGF21
MIER2
PLEKHA4

PPP2R1A
TNPO2
WDR83OS
GCDH
KLF1
DNASE2
MAST1
JAK3
RPL18A
SLC5A5
ARRDC2
ISYNA1
ELL
CRTC1
COMP
UPK1A
COPE
DDX49
ATP4A
ARMC6
TMEM147
GAPDHS
MAG
TMEM59L
USF2
LSR
KXD1
FKBP8
SUGP1
HPN
SCN1B
ERF
GSK3A
ATP13A1
ZNF574
SIPA1L3
ETHE1
CADM4
SMG9
AVL9
RUNDC3B
CFAP69
GTPBP10
RASA4
PMPCB
DNAJC2
TFPI2
NAMPT
POLR1F
PIK3CG
PON3
PON2
ITGB8
SP4
WDR91
CBLL1

DLX5
MTPN
PTN
MPP6
GSDME
ATP6V0A4
TTC26
NPVF
ADAP1
TFEC
CAV2
CAV1
MET
RNF32
HOXA1
DNAJB6
HOXA2
HOXA3
LFNG
HOXA5
HOXA6
BRAT1
VIPR2
TSPAN12
SSBP1
HOXA13
CPED1
EVX1
HIBADH
TAX1BP1
CPVL
GRB10
ABHD11
FKBP14
PLEKHA8
NOD1
GARS1
CRHR2
MINDY4
GHRHR
CASP2
CHCHD2
HSPB1
NPTX2
PDAP1
BUD31
ZKSCAN1
EIF3B
SNX8
NUDT1
PTPRZ1
TAF6
WASL
HYAL4
SPAM1
AIMP2

TFR2
FSCN3
MOSPD3
PCOLCE
FBXO24
PPP1R17
RBM28
USP42
IMPDH1
AGFG2
LSM5
SERPINE1
C1GALT1
PLOD3
RPA3
ZNHIT1
CLDN15
GLCCI1
PHF14
NRF1
TMEM106B
EZH2
CEP41
SFRP4
MEST
MEOX2
ANKMY2
POU6F2
TSPAN13
RARRES2
AGR2
AHR
GLI3
MRPL32
BLVRA
URGCP
TMEM248
RHEB
PRKAG2
AEBP1
POLD2
GCK
BCL7B
YKT6
TBL2
CLIP2
EIF4H
LIMK1
SPATA6L
SLC1A1
FKTN
FSD1L
SPIN1
NMRK1
TMEM245
MEGF9

TRIM14
CORO2A
TGFBR1
SEC61B
C5
OGN
ASPN
ECM2
LHX6
PTGR1
SUSD1
AMBP
TNFSF8
ENG
AK1
CDC37L1
PLGRKT
TBC1D13
RIC1
DOCK8
KANK1
NCS1
TESK1
CA9
FUBP3
TYRP1
CREB3
RGP1
MPDZ
DDX58
EDF1
GLIS3
RAPGEF1
NPDC1
APBA1
ABCA2
ABHD17B
EXOSC3
ZFAND5
PDLIM1
CCNJ
DNTT
GATA3
HPS1
PHYH
RASSF4
RAB11FIP2
CXCL12
EIF3A
PKD2L1
TRDMT1
DDX50
MAPK8
SEC23IP
ATE1
NSMCE4A

PLEKHA1
PALD1
UNC5B
CDH23
VSIR
SPOCK2
MICU1
PPP3CB
CCSER2
BMPR1A
MINPP1
LIPA
TLX1
TWNK
LZTS2
SFXN3
KAZALD1
FBXW4
FGF8
NPM3
TNKS2
PITX3
GBF1
ARHGAP21
CPEB3
FBXL15
CUEDC2
SUFU
ACBD5
LHPP
LARP4B
GTPBP4
EDRF1
BCCIP
MTPAP
NEURL1
SH3PXD2A
PITRM1
STN1
MAP3K8
DKK1
GLRX3
SORCS1
XPNPEP1
SMC3
SHOC2
TFAM
CCDC6
CUL2
CCNY
UBE2S
RPL28
ZMIZ1
DNAJC12
PPIF
PBLD

TSPAN14
LGI1
CRYBA1
NUFIP2
GIT1
RPL19
FBXL20
RUNDC3A
UBTF
CSF3
PSMD3
CASC3
RAPGEFL1
RGS9
RNF43
ASPA
RAD51C
MTMR4
TRIM37
P2RX1
DHX40
TUBD1
KPNB1
PNPO
RPS6KB1
CDK5RAP3
CBX1
RECQL5
PIGL
GALK1
INTS2
CAMTA2
MED13
HOXB6
ENO3
PFN1
RNF167
SLC25A11
CHRNE
RAI1
NUP88
C1QBP
SLC6A4
BLMH
CPD
CCDC47
MED31
DRG2
FTSJ3
AKAP10
ALDH3A1
SMARCD2
SYNGR2
B9D1
UTP6
DDX5

C17orf75
PSMD11
LGALS3BP
PEX12
DHX58
KAT2A
RAB5C
NAGLU
HSD17B1
MLX
CNTNAP1
ABI3
EZH1
DLX4
PPP1R9B
COL1A1
SGCA
MRPL27
VAT1
LRRC59
RND2
ALOX12
HDAC5
LUC7L3
MPP2
DUSP3
CACNG1
EFTUD2
HLF
SLC16A6
PRKAR1A
EFNB3
FAM20A
MMD
RANGRF
DPH1
DHRS7B
WSB1
RCVRN
SLC9A3R1
NAT9
TMEM104
VTN
TNFAIP1
IFT20
TMEM97
CDR2L
PMP22
FOXN1
UNC119
ALDOC
SUPT6H
RAB34
PHF12
PHOX2B
TMEM33

GABRA4
GNRHR
SLAIN2
OCIAD1
CWH43
DCUN1D4
ODAM
CHIC2
CRACD
LAMTOR3
NFKB1
AREG
MANBA
UBE2D3
ELF2
NDUFC1
TBC1D9
ZNF330
INPP4B
GAB1
IL2
CPE
RPL34
WFS1
ANXA10
GRPEL1
GAR1
CLCN3
AADAT
GALNT7
DHX15
SEPSECS
CPZ
TRIM2
FBXW7
NEIL3
TBC1D19
NSD2
SH3D19
NKX3-2
MFSD10
GLRB
BST1
RAPGEF2
HGFAC
SNX25
LRP2BP
UFSP2
KLF3
KLHL5
FAM149A
NCAPG
UGDH
PPARGC1A
DDX25
CRYAB

DBX1
HTATIP2
CTSC
ZBTB16
ELP4
ZPR1
TECTA
SC5D
CRTAM
JHY
B3GAT1
HSPA8
P2RX3
DNAJC4
SIAE
SNX15
LPXN
DTX4
ATG2A
OSBP
UNC93B1
PUS3
DCPS
KMT5B
FOXRED1
PPP6R3
NRXN2
ST3GAL4
CCND1
CCDC86
PRPF19
TMEM109
CCKBR
HPX
TRIM3
FOLR1
ANAPC15
PANX1
ARHGEF17
APOA5
APOA4
RNF141
IL10RA
GALNT18
UBE4A
DDX6
UPK2
CBL
NECTIN1
HIPK3
FBXO3
PDHX
SLC1A2
COMMD9
SLC15A3
ACCS

MDK
AMBRA1
MADD
NAA40
CD81
SLC35F2
ELMOD1
CALCA
SOX6
C11orf58
PITPNM1
RPS13
AIP
NUP98
NDUFS8
CHKA
POU2AF1
PTPN5
PSMD9
P3H3
PPFIBP1
PRPF40B
CD69
PRDM4
COQ5
SELPLG
CORO1C
ASIC1
DAO
TSPAN11
KCTD10
SLC11A2
MLEC
MVK
CSRNP2
CAMKK2
BIN2
IL23A
ATP5F1B
PTGES3
SYT10
RSRC2
CYP27B1
MYF6
MYF5
KRT18
ACSS3
TNS2
GLI1
PPM1H
METAP2
LTA4H
ELK3
TRPV4
ITFG2
FOXM1

PARP11
ARPC3
VPS29
FGF6
MYL2
RAD51AP1
AKAP3
KCNA1
CREBL2
ALDH2
CDKN1B
GPRC5D
NAA25
GSG1
SCNN1A
LTBR
OGFOD2
CDK2AP1
ART4
MGP
RASAL1
ARHGDIB
GTF2H3
EIF2B1
DDX55
SLC38A1
RERGL
ENDOU
SPRING1
FZD10
RFC5
STX2
ADGRD1
COPZ1
TBC1D30
CAND1
RAB5B
MDM1
NUP107
CNOT2
TIMELESS
CPSF6
KRR1
MRPL51
GAPDH
NOP2
CHD4
ACRBP
UHRF1BP1L
COPS7A
ING4
GNB3
CDCA3
CHPT1
USP5
TPI1

GNPTAB
SPSB2
ATN1
C12orf57
PTPN6
LPCAT3
NT5DC3
APOBEC1
SUDS3
GOLT1B
PRKAB1
CMAS
HCFC2
ST8SIA1
C2CD5
AICDA
RAB35
PHC1
COX6A1
RFX4
RIC8B
SRSF9
FGFR1OP2
COL12A1
TDP2
FRK
DSE
RWDD1
MAK
GCNT2
SMIM8
NEDD9
ADTRP
ASF1A
FAM184A
RNGTT
MAN1A1
GABRR2
SERINC1
HDDC2
TPD52L1
HINT3
NCOA7
RIPOR2
SASH1
UST
FBXO5
MTRF1L
PPARD
OPRM1
FANCE
TULP1
SLC26A8
RHAG
KCTD20
SRSF3

MRPL18
IL17A
IL17F
MCM3
RNF8
PHACTR1
MDGA1
CILK1
FBXO9
CD83
GLP1R
SAYSD1
BACH2
RBM24
CAP2
ZNF451
BAG2
RAB23
TSPO2
FHL5
GPR63
FBXL4
CCNC
PRDM13
E2F3
PTP4A1
SIM1
ASCC3
BVES
COL9A1
MED23
WASF1
GPLD1
ALDH5A1
VNN1
ACOT13
SMAP1
RPS12
C6orf62
B3GAT2
GMNN
EYA4
SOBP
NR2E1
SLC17A2
HBS1L
PEX7
ZBTB24
FIG4
PERP
ARFGEF3
SLC16A10
HECA
ADGRG6
PHACTR2
EPM2A

CCR6
UNC93A
SLC22A2
QKI
C6orf118
MDFI
TFEB
CCND3
BYSL
TBP
GUCA1B
PRPH2
BICRAL
PPP2R5D
MRPL2
PTK7
SRF
CUL9
DUSP22
EXOC2
COX7A2
TMEM30A
SENP6
PRPF4B
TTK
SLC29A1
CCN6
LAMA4
TENT5A
ENPP5
LY86
PRSS16
MEP1A
TBX18
ERBIN
HARS2
NUDT12
MAN2A1
C7
TENT4A
GHR
HMGCS1
DAP
NME5
BRD8
KIF20A
NNT
MRPS30
HSPA9
MRPS27
PFDN1
HBEGF
SLC4A9
LOX
GZMK
APBB3

TMCO6
SPARC
IK
HMGCR
CERT1
FAF2
HAND1
CLK4
GRM6
ITK
RNF130
THG1L
CLINT1
THBS4
CNOT6
IL12B
TTC1
MSH3
CCNG1
POLR3G
DROSHA
LMNB1
ARRDC3
GOLPH3
SUB1
NPR3
FAM172A
SLC27A6
TARS1
IRX4
LNPEP
RAD1
BRIX1
AGXT2
PRLR
IL5
ST8SIA4
GNPDA1
PCDH12
SKP1
NUP155
PPP2CA
FGF1
NR3C1
PPWD1
LIFR
TRIM23
TRAPPC13
C9
SEC24A
TXNDC15
TTC33
RARS1
WWC1
MACROH2A1
TCERG1

CSNK1A1
HMGXB3
ERGIC1
PDGFRB
CDX1
ATP6V0E1
BNIP1
STC2
CPEB4
HRH2
DBN1
ZNF346
UNC5A
EHHADH
SMC4
SELENOK
ACTR8
TBCCD1
TIMMDC1
CRBN
KNG1
HRG
BCL6
HGD
CLDN16
ARL6
NPHP3
AMOTL2
NIT2
FAM162A
OGG1
PCCB
UBE3A
ARMC8
CEP70
RBP2
RBP1
SLC25A36
RNF7
TFDP2
XRN1
BCHE
SERPINI2
PDCD10
COL7A1
HES1
USP4
ACAP2
ECT2
GNAT1
TFG
HYAL1
TUSC2
NPRL2
RPL24
CYB561D2

C3orf14
FXR1
CBLB
BBX
IFT57
IQCG
GBE1
UMPS
NCBP2
SNX4
FRMD4B
SLC41A3
ROPN1B
ATP6V1A
ABTB1
PODXL2
UPK1B
CSPG5
KLHL18
SCAP
NEK11
PLSCR4
HEMK1
CISH
MAPKAPK3
ACVR2B
WDR48
COMMD2
GORASP1
PEX5L
RRP9
ABCC5
ABHD14B
EIF1B
KLHL24
PLCH1
VIPR1
SSR3
ZBTB47
TNNC1
NKTR
CLCN2
FOXP1
EIF4G1
SPCS1
NEK4
SLC4A3
ADAM23
DGUOK
MOB1A
KANSL3
LMAN2L
RTKN
TTL
IL1A
PIKFYVE

KCNIP3
NCL
SLC35F5
ZAP70
ACTR3
STEAP3
EPB41L5
TFCP2L1
SF3B6
TP53I3
DNAJC27
POMC
STAM2
OTOF
GPD2
CENPA
CYTIP
ACVR1
TANC1
SLC30A3
MPV17
GTF3C2
EIF2B4
NRBP1
ITGB6
FNDC4
ITGA4
PSMD14
SNX17
PPM1G
PDE1A
REEP6
GCG
APC2
IFIH1
RPS15
GCA
MOGS
NDUFS7
PCGF1
GRB14
CLIP4
TLX2
SPTBN1
AUP1
RTN4
HTRA2
LOXL3
DOK1
GALNT3
POLE4
CCDC88A
ACADL
EVA1A
MRPL19
LANCL1

WDR75
EFEMP1
FANCL
FN1
STAT1
GLS
PAPOLG
PECR
UNC50
IGFBP2
ELMOD3
IGFBP5
USP34
KCNJ13
CCT4
GGCX
NEU2
EHBP1
OTX1
TXNDC9
COQ10B
SF3B1
ST3GAL5
CHST10
PDCL3
HSPE1
KDM3A
PLCD4
ZNF142
IL1R2
PRKAG3
SMYD1
IL1R1
WNT6
IL1RL2
IL1RL1
IL18R1
IL18RAP
SLC9A2
FHL2
CNPPD1
UXS1
ABCB6
STK16
SLC5A7
HDLBP
PPP1R7
STK25
PROC
ID2
TAF1B
ODC1
BIRC6
NOL10
PLEKHB2
GORASP2

STRN
CEBPZ
PRKD3
DCAF17
QPCT
RAB3GAP1
DLX2
LCT
DARS1
SRSF7
SDC1
SLC1A4
SOS1
KYNU
WIPF1
ORC2
COX7A2L
PNO1
ORC4
PLEK
RND3
ATF2
AAK1
TRAK2
C2orf42
TIA1
PCYOX1
KISS1R
EPAS1
ARID3A
SUMO1
CD207
GRIN3B
VAX2
ATP6V1B1
NFE2L2
MSH6
PLEKHA3
SPR
EPHA4
PARD3B
FARSB
BCL9
PRRX1
DHCR24
DNAJC16
MARK1
TNR
MORN1
GPX7
CACYBP
SCP2
PAPPA2
RALGPS2
ANGPTL1
CEP104

FAM20B
TCEANC2
TMEM59
LRRC42
WRAP73
NPHS2
MRPL37
ICMT
RPL22
QSOX1
STXBP3
PHF13
ERRFI1
PARK7
ELAPOR1
OPRD1
AMPD2
SRSF4
MECR
KCNC4
EDEM3
WDR77
ATP5PB
RAP1A
HDAC1
CAPZA1
S100PBP
RNF19B
TRIM62
ASH1L
DLGAP3
SFPQ
ARHGEF2
LAMTOR2
MEF2D
SRM
FBXO2
FBXO6
C1orf21
SWT1
MAD2L2
DNAJC6
LEPR
IVNS1ABP
KIAA2013
MFN2
PRG4
SMG7
NCF2
PDC
SLC35D1
PLA2G4A
GADD45A
WLS
PRDM2
RGS2

RPE65
RO60
AMPD1
BCAS2
SRSF11
CTH
AGMAT
OLFML3
PLEKHM2
CRYZ
PHTF1
ZBTB17
TFAP2E
NR5A2
TMEM9
ADPRS
MAP7D1
WARS2
HAO2
OSCP1
EXOC8
GNPAT
TSNAX
C1orf109
RRAGC
NID1
LGALS8
NT5C1A
MTR
MYCL
SIPA1L2
RLF
KMO
KCNQ4
RIMS3
ETV3
ACADM
ST6GALNAC5
SLAMF1
ADGRL2
PADI2
SDHB
MFAP2
RPF1
KDM5B
UAP1
CTBS
KLHL12
IGSF21
SSX2IP
ZNHIT6
PLA2G2D
RBBP5
KIF17
CDK18
RAB29

ECE1
HMGCL
GALE
PRPF3
APH1A
P3H1
SLC2A1
EBNA1BP2
CDC20
MPL
ARTN
IPO13
ATP6V0B
B4GALT2
ERI3
PTCH2
AKR1A1
PRDX1
TSPAN1
BLZF1
CCDC181
SLC19A2
FAAH
TMED5
DR1
CNN3
PRRC2C
F3
ABCD3
VAMP4
DPH5
FASLG
PTBP2
PRDX6
DARS2
HSD11B1
IRF6
UTP25
PLPPR5
PLPPR4
SERPINC1
RCAN3
SYF2
RSRP1
RCOR3
STMN1
MTFR1L
MAN1C1
NEK2
DHDDS
NENF
NSL1
PROX1
ARID1A
RPA2
PPP1R8

STX12
MTARC2
SLC5A9
OSBPL9
TXNDC12
ESYT2
MESD
RCN2
CHRNB4
COLEC11
A4GNT
STK11
KMT2A
IFT46
APOA1
KPTN
TNNT2
DDX59
CAMSAP2
ATF6
MREG
TNP1
FASTKD2
NRP2
CREB1
KLF7
TTR
B4GALT6
C1orf54
CA14
ATP10B
SPCS2
USP35
ELOVL4
FILIP1
HMGN3
UBE3D
CNR1
SPACA1
ANKRD13C
SGIP1
PHF3
ZC2HC1B
PLAGL1
FBXO30
TNFAIP3
AKAP7
RAB32
MYB
ALDH8A1
SGK1
RNF146
ARG1
CCN2
TCF21
PMFBP1

FBXL5
SLC16A7
RXYLT1
VAMP8
DCLRE1B
FOXO3
ARMC2
GHRH
RPN2
TGIF2
CASQ2
OLFM3
PKD2
ABCG2
SPP1
STBD1
CCNI
MFSD1
RAB3GAP2
EEF2KMT
PPL
UBN1
KLF12
UCHL3
PCDH17
HS1BP3
LDAH
WDR35
CCND2
FGF23
ELL2
CYP20A1
NDUFB3
GTF3C3
SATB2
UBE2B
TRPM6
GDA
KLF9
ECRG4
ITGB1BP1
CPSF3
PIGZ
SENP5
C1orf198
HEATR1
RAD23B
FKBP15
CTNNAL1
ABITRAM
DYNC2I2
SET
PTPA
GLE1
RAB14
CNTRL

TRIM32
FBXW2
PHF19
NEK6
BSPRY
PPP6C
NDUFA8
HDHD3
RBM18
SLC46A2
HSDL2
MAPKAP1
NR4A3
INVS
GALNT12
DENND1A
ALG2
CSF3R
KDSR
VPS4B
ONECUT2
C19orf25
ZBTB45
YLPM1
DCAF4
PROX2
VSX2
FCF1
PGF
BBOF1
NEK9
ACYP1
IFT43
NPC2
DNAL1
IRF2BPL
LTBP2
AREL1
MLH3
TTLL5
FLVCR2
ABCD4
DLST
TGFB3
ZC2HC1C
SLIRP
RBM25
ALDH6A1
GPR68
EIF2B2
NRDE2
COQ6
ZNF410
RHOQ
GPR75
SUPT7L

KLHL29
DNMT3A
TMEM214
ATAD2B
FKBP1B
ATL2
YPEL5
FAM98A
YIPF4
AFTPH
LGALSL
CNRIP1
BCL11A
CRIPT
EPCAM
SLC17A5
OGFRL1
SLF2
IDE
TECTB
ELOVL3
NKX2-3
IFIT2
GPAM
CUTC
PPP1R3C
PYROXD2
CNNM1
MXI1
SMNDC1
C10orf88
HELLS
PRLHR
TCTN3
DENND10
AVPI1
WDR11
ARMH3
KCNIP2
CFAP58
GOT1
SFRP5
GNA13
HOXB8
HOXB5
HOXB3
HOXB1
DUSP1
PANK3
MSX2
TEK
RCL1
CAAP1
EQTN
INSL6
MLANA

CD274
GRIA2
NUP43
CCDC170
PCMT1
PLEKHG1
MYCT1
TASL
CYSTM1
WDR55
ARAP3
TNN
MRPS14
CENPL
TNFSF18
GORAB
ACAT2
TCP1
SNX19
KCNJ5
MSANTD2
PDZD11
SLC10A7
NUDCD1
ENY2
MASTL
KIAA1217
PLXDC2
EPC1
CCDC77
TAF12
TNFSF11
MTRF1
DNAJC15
PROSER1
UFM1
WBP4
ELF1
HSPH1
KBTBD7
ALG5
EXOSC8
ETF1
TGFBI
FAM53C
SIL1
PAIP2
MYOT
KDM3B
EGR1
ZFP30
NR2C1
UTP20
TMPO
GLT8D2
MTERF2

SOCS2
NFYB
WASHC3
APAF1
DUSP4
CLU
SORBS3
PTK2B
CHRNA2
ADRA1A
PDLIM2
EPHX2
RNF170
UBIAD1
TARDBP
ZNF706
LYPLA1
CRISPLD1
COPS5
RDH10
AKAP1
COIL
TRIM25
SCPEP1
SPOP
TBX2
SLC35B1
TBX4
FAM117A
NCAPH
LRAT
TMEM131L
MND1
TRIM6
TENT4B
ADCY7
TSHZ3
ECHDC2
PLBD1
PYROXD1
KCNJ8
PSPC1
LHX4
RNF2
TRMT1L
SEC22A
CSTA
DPPA4
POPDC2
B4GALT4
NAA50
KIF18A
GJA8
DESI2
MAPK8IP1
CRY2

PEX16
DEPDC7
CAT
ZMYM2
GJB6
TBC1D15
ADGRB2
HCRTR1
ZCCHC17
FABP3
KHDRBS1
TMEM39B
POLR3GL
GHSR
TNFSF10
ZNF639
SLITRK3
PIK3CA
TMEM156
LIAS
CPXM2
TMEM54
ZSCAN20
CSMD2
LRIF1
CLCC1
GPSM2
GTDC1
CXCR4
ZRANB3
ACVR2A
POLK
FLT3
RPL21
MTIF3
RASL11A
UBL3
FYTTD1
MTERF4
XPNPEP2
SASH3
OCRL
MRPS2
FMOD
MYOG
LAX1
PLG
KIAA1191
COPA
RBBP6
ZC3H7A
SERAC1
ANXA11
LDB3
OPN4
PRXL2A

NAA60
RPL5
ODF2L
PTGFR
SPATA1
TRMT13
LRRC39
RWDD3
ZNF644
RPAP2
SLC66A2
BBS9
PMS2
ZMIZ2
SEPTIN7
EEPD1
KLHL7
HERPUD2
CBX3
HNRNPA2B1
NXPH1
NPY
FAM126A
HOXA7
INHBA
FKBP9
NT5C3A
ARL4A
POLM
MRM2
SMU1
GLIPR2
SLC25A51
CLTA
RECK
ACO1
PHF24
DNAI1
DCAF10
CNTFR
TRIM24
CYREN
CALD1
AKR1D1
NEUROG3
PLAU
SRGN
CHST3
BICC1
CISD1
EGR2
ECD
P4HA1
SLC25A16
ZWINT
VPS26A

CIT
IFT81
ACADS
HVCN1
DDX54
CDKN2C
RNF11
RASSF8
BHLHE41
SSPN
CCDC91
WWP1
ACOT9
PRDX4
DDX39A
PKN1
TRIR
WDR83
GIPC1
SPRYD7
EBPL
ZC3H13
NLN
CENPK
OPTN
ITIH5
ATF1
TSFM
NEUROD4
NCKAP1L
MMP19
PFDN5
SPATS2
ORMDL2
NR4A1
PDE1B
HOXC13
CDK2
LRP1
HOXC11
ATG101
NFE2
HOXC12
IKZF4
SMUG1
EEF1AKMT3
SARDH
DBH
ATPAF1
STIL
IL13RA2
COL10A1
NDUFAF4
PLP1
MORF4L2
RAB9B

NRK
FAM199X
RAB9A
METTL8
TTC21B
NMI
TNFAIP6
ACVR1C
BAZ2B
LPGAT1
BATF3
G0S2
KCNJ2
RAP2C
EXOSC9
B9D2
COQ8B
PFKFB2
C4BPB
RAB38
GPR83
MXD4
CKS2
DAW1
ACSL3
CHPF
DNPEP
INHA
MOGAT1
OBSL1
FAM124B
SLC12A4
ENKD1
MC3R
GCNT7
FAM210B
SNX21
TTPAL
PREX1
KCNS1
SDC4
NCOA3
MATN4
NCOA5
VAPB
PARD6B
ATP5F1E
CHD6
PLCG1
TOX2
SRSF6
GDAP1L1
GTSF1L
ZNFX1
EDN3
CSE1L

RAB22A
STAU1
SNAI1
MOCS3
STX16
PMEPA1
RNF114
ANKRD60
DDX27
RBPJL
C20orf85
TP53TG5
PCK1
NEURL2
MTRR
FASTKD3
CHST8
IQSEC2
VAMP7
IL9R
STAMBP
NAGK
MCEE
PAIP2B
SNRNP27
MPHOSPH10
IL17C
ATP8A1
USP22
POF1B
HIF3A
IRGC
LYPD3
NDP
USP9X
CRISP2
F13A1
TRERF1
PACSIN1
BTN2A2
SIRT5
WRNIP1
RRP36
SNRPC
XPO5
ABCC10
PEX6
NQO2
OARD1
UNC5CL
AARS2
MOCS1
MED20
TBCC
SPDEF
MAD2L1BP

APOBEC2
KLHDC3
GNMT
MEA1
KLHL31
CDKN1A
SOX4
GLO1
RREB1
SSR1
RIOK1
NRN1
SLC35B3
RPP40
ATXN1
NUP153
DEK
EEF1E1
RUNX2
OPN5
GCM2
RAB17
EREG
MYRF
EMC3
SSUH2
WNT1
CNOT1
LRRC29
BBS2
C16orf70
GOT2
DOK4
GPR18
TMTC4
SLC10A2
ABCC4
EFNB2
TM9SF2
HROB
IRF1
UPF3B
RNF113A
TMEM255A
NDUFA1
AMELX
BMP4
PTGER2
FAM193A
TEKT3
SLC25A35
MRPS7
GGA3
ARMC7
NUP85
SLC25A19

MIF4GD
NT5C
MSTO1
TTF1
GTF3C4
DDX31
BARHL1
PPP1R12C
FNDC11
PPDPF
IL1B
CHCHD5
PAX8
INSIG2
POLR1B
CCDC93
SLC25A23
PSPN
GTF2F1
ALKBH7
CLPP
THOC2
MED1
RPL23
ATG4C
SH2D3A
TRIP10
GPR108
TNFSF14
FOSB
OPA3
SNRPD2
RTN2
EML2
VASP
SYMPK
GPCPD1
SDCBP2
PANK2
FOXA2
GZF1
PAX1
CENPB
PSMF1
NKX2-2
RBCK1
TMX4
STK35
SNRPB
NRSN2
AP5S1
BMP2
FLRT3
OVOL2
PCSK2
GFRA4

MKKS
BFSP1
DSTN
LAMP5
SNRPB2
MGME1
LRRN4
TBC1D20
ITPA
TCF15
OTOR
MCM8
BANF2
TMEM74B
FAM110A
MRPS26
S1PR4
NCLN
MAX
GDF5
MMP24
ID1
RALY
DYNLRB1
EIF2S2
ROMO1
FAM83C
CEP250
PLAGL2
GRPR
AMOT
TMEM115
PSMB2
UROD
ST3GAL3
TMEM53
HECTD3
XRCC3
TUBGCP3
MCF2L
F10
PCID2
LRFN3
PDCD2L
RBM42
KIRREL2
FFAR2
FFAR1
COX6B1
KRT36
THRA
CCR7
NR1D1
FRMD8
PRDX5
BCL2L12

IRF3
PRMT1
RRAS
PRRG2
SCAF1
PRR12
TSKS
FLRT1
ASL
SBDS
WNK4
BECN1
PRKCG
TRAP1
NSRP1
AHDC1
ZNF384
EMG1
UXT
CFP
ELK1
PCNX4
ATG14
KTN1
SIX1
RHOJ
L3HYPDH
HSPA2
ZBTB1
TRMT5
PLEKHG3
RHOT1
EVI2A
OMG
AIF1L
NUP214
AVPR2
SLC10A3
MAP2K2
HNRNPH2
ARMCX1
TMEM35A
TIMM8A
ZC4H2
INTS11
RGS13
IPPK
OMD
FGD3
HIVEP3
PPCS
BCL11B
COX7C
TRAF2
ABHD8
MASP1

ATP13A4
PLAAT1
RAP1B
IL22
BEST3
RAB3IP
PTPRB
DYRK2
YEATS4
TAS2R3
TAS2R4
LRRC61
IDUA
FGFRL1
TMEM175
AUNIP
PIN1
FBXL12
EMC1
PLA2G5
UBR4
HP1BP3
SIN3B
SLC35E1
EPS15L1
GFER
SYNGR3
PKMYT1
WDR24
FBXL16
CHTF18
GNG13
KDM4B
TICAM1
METTL25
IL17B
EMC6
METTL16
VIL1
AAMP
PNKD
RNF6
ECHS1
GNG11
GNGT1
HIP1
PTPN12
FGL2
STYXL1
STEAP4
PEX1
MTERF1
SGCE
RBM48
CASD1
LRFN1

ZFP36
SRD5A3
SPINK2
RASL11B
PAICS
KDR
PPAT
ADM2
DGCR8
ASPHD2
VPREB3
SDF2L1
GAL3ST1
GNAZ
MGAT3
ADORA2A
ATF4
A4GALT
CDC42EP1
MCHR1
TPST2
BAIAP2L2
MPST
TST
LIF
C22orf23
RIBC2
KRT17
EMC4
RNF112
SPECC1
CPA4
POT1
ATP6V1F
LSM8
CDHR3
PRKRIP1
VGF
FOXP2
STRIP2
IFT22
MKLN1
DNAJB9
FLNC
LRRC4
CALU
CCDC136
SMO
IRF5
LRRC17
KLHDC10
NDUFA5
FEZF1
OPN1SW
MYO1B
HOXD1

HOXD3
MTX2
GAD1
OSGEPL1
ORMDL1
HAT1
HOXD9
HOXD10
HOXD11
HOXD13
SNRPN
PSMG2
TWSG1
ARHGAP22
EIF2AK4
MYO5C
CGNL1
TTBK2
ELL3
CCDC32
INO80
ICE2
DLL4
MINDY2
IVD
CHAC1
CLN6
ARPP19
VPS13C
CALML4
LOXL1
ACKR4
ANAPC13
MBD4
COPB1
PSMA1
SUMF2
SPCS3
BBOX1
MYOD1
SERGEF
KCNC1
TPH1
CSRP3
E2F8
DCTD
SOX15
PIMREG
RPAIN
SHBG
PLD2
AIPL1
CD68
TXNDC17
ATP1B2
FXR2

KIF1C
MPDU1
PHF20L1
LRRC6
CCNT1
PUS7L
KRI1
ILF3
SLC44A2
CDKN2D
MTUS1
KLK14
KLK10
KLK8
NGDN
RAB2B
BCL2L2
AJUBA
DTD2
PARP2
HEATR5A
FOXA1
SNX6
EAPP
EGLN3
RNASE1
DAD1
EPB41L4A
CDO1
REEP5
ITFG1
FOXJ1
SEC14L1
RHBDF2
AANAT
ARHGEF6
FGF13
ASH2L
TTI2
ART1
CHRNA10
SGO1
CDH15
PGAP6
DOHH
MAU2
SHC2
PLPPR3
ABHD17A
LBP
SYT5
TNNI3
GAMT
PUDP
PHF10
PRRG3

KCNA5
CRACR2A
NXNL2
STARD8
FAM155B
GDPD2
SAT1
GNL3L
SH3BP4
MOSPD2
DOCK6
ECSIT
LDLR
ELOF1
ANGPTL8
PRKCSH
CNN1
CDC16
ZSCAN10
THEM6
NECTIN2
APOE
TOMM40
GADD45G
LRCH2
XPO7
ACE2
FAM98C
RPL36
GTPBP3
PLVAP
NSUN5
USHBP1
COLGALT1
DDA1
PGLS
LSM7
TULP4
SNX9
RTN4IP1
QRSL1
MTRES1
MAS1
ACSBG2
MLLT1
AFDN
NDUFA10
EPO
ARPC1B
CACNG6
FCHO1
PXDN
PGPEP1
LSM4
HRC
TRPM4

SULT4A1
CRB3
CAMSAP1
UBAC1
ZBTB46
SAMD10
ATXN10
TUBGCP2
CALY
MNX1
CEP85
GATA5
OSBPL2
ADRM1
ASS1
PRDM12
EXOSC2
POMT1
UCK1
FIBCD1
UBE2M
TRIM28
METTL26
YIPF2
ATG4D
EIF2S3
TMEM160
ZC3H4
NPAS1
GMFG
MAP3K10
ARHGEF16
LRRC47
SMPDL3B
THEMIS2
CLIP1
HIP1R
ZNF317
EIF3G
ANGPTL6
SHFL
PNCK
DKC1
DUSP9
MPP1
ZNF236
SLC7A10
LRP3
C12orf65
NOL11
UBE4B
CASZ1
HSD17B3
HABP4
SLC35D2
PRRG1

UBA1
RGN
PPIL4
LATS1
EPS8L1
AAR2
TTLL9
RBM39
COX4I2
BPIFA3
ZNF341
GGT7
ACSS2
GFAP
HIGD1B
ATP6V1E1
ZNF428
TEX101
COX4I1
EMC8
GSE1
GINS2
CHMP1A
SH3BGRL
COX7B
SLC34A1
F12
PRR7
IDO1
CAP1
PPT1
RAB11FIP4
RLIM
ABCB7
TRAF3
MRPS25
SH3BP5
HACL1
TBC1D5
CAPN7
RBSN
GALNT15
SLC6A6
NAPSA
NR1H2
LRRC4B
PDLIM4
KIF3A
MGAT1
GFPT2
PSME3
PSMC3IP
ACLY
VPS25
RAMP2
G6PC1

NDUFA2
DIAPH1
NDFIP1
ACAP3
ANO1
TMEM204
KREMEN2
THOC6
TRAF7
BARX1
NINJ1
CA6
NPHP4
MAP1B
IL13RA1
WDR44
ZCCHC9
TNS4
TOP2A
STARD3
PPP1R1B
KHDRBS3
CHD1L
PEX11B
FMO5
PIAS3
PRKAB2
FSHB
RAI2
MCCC2
SELENOS
CHSY1
SNRPA1
NR0B2
LIN28A
THAP1
RHPN2
C19orf12
FAAP24
ACTR10
ABHD12B
GCH1
LGALS3
DNAJB1
FBXW9
MATN3
TRIM21
SPATA6
LRRC41
DHX30
RAF1
PPARG
NUP210
FCRLA
HSD17B7
ENOSF1

EMILIN2
ARFIP2
CNGA4
RRP8
EFR3A
PTCD3
IMMT
MRPL35
IQCA1
ILKAP
RAMP1
SCLY
PTPRE
PRKAA1
RAP1GAP2
CLUH
INPP5K
RPA1
SERPINF1
UBE2G1
TBC1D14
TMEM128
COQ3
PNISR
POPDC3
SEC61G
LANCL2
FIGNL1
DDC
GRSF1
ENAM
JCHAIN
ANKRD17
UTP3
ITGB4
WBP2
H3-3B
UNK
TRIM47
ZRANB2
KDM6B
SLC52A1
GUCY2D
DLG4
MATN2
REEP2
PCBD2
SDF2
FLOT2
ERAL1
PRMT7
NIP7
TERF2
VPS4A
MTSS2
HSPA12B

ANKEF1
SCP2D1
PCED1A
SNAP25
BTBD3
PCNA
NXT1
POLR3F
RIN2
PTPRA
DAP3
RHBG
KHDC4
BCAN
ARHGEF11
HAPLN2
DCAF8
SYT11
IGHMBP2
TESMIN
MMACHC
DPH2
TOE1
NASP
CTNNBL1
LPIN3
ZSWIM3
RBM38
VSTM2L
OSER1
SERINC3
PPP1R3D
DMGDH
BHMT2
KANK4
ANGPTL3
SYT4
SLC14A2
FBXO44
CPLANE2
CASP9
NMUR2
DCTN4
XPO4
POMP
CDK8
ALOX5AP
WASF3
GPR12
CHRM3
PEMT
SCO1
MPRIP
CHI3L1
MYBPH
PIK3C2B

DSTYK
SLC41A1
LGR6
TMCC2
COG6
SPART
RXFP2
TRPC4
POSTN
RFXAP
TPT1
GPALPP1
KL
RFC3
STARD13
IRS4
MORC4
RNF128
BEX1
FAM104A
SLC39A11
SRRM1
KMT5C
ZNF414
HSPBP1
SLF1
CNDP2
MACROD1
WDR74
LGALS12
RTN3
MED10
MORC2
C1QTNF6
FAM83F
ADCK2
MKRN1
NTS
BTG1
C12orf29
ATP13A3
SFTPD
DYDC2
TMTC1
IPO8
LARS1
IMPA1
LRRCC1
E2F5
SWAP70
ARNTL
LYVE1
AMPD3
SBF2
RRAS2
HSD17B4

ZFC3H1
RNF122
DUSP26
DPF2
MEN1
ERG28
GSC
DGLUCY
NUMB
VRTN
TTC9
MED6
EIF2S1
ELP3
ADAMDEC1
MRO
MBD2
MRPS36
CCNB1
CD180
CAMK1
THUMPD3
BHLHE40
EDEM1
CNTN6
CHL1
DPH6
KATNBL1
EMC7
GNAT2
PRPF38B
TSPAN2
TSHB
GSTM3
SYT6
VAV3
PSRC1
PTPN22
SORT1
WNT2B
PTGFRN
LAMTOR5
NOTCH2
TRIM45
CEPT1
CD101
VTCN1
NGF
AP4B1
NAPG
SPIRE1
PPHLN1
FKBP11
ARF3
TMEM106C
SLC38A2

YWHAQ
KIDINS220
GRHL1
ROCK2
RSAD2
MYCN
LPIN1
CMPK2
IAH1
IL6ST
FST
NAV1
CDC73
TIMM17A
ERN2
RPS15A
RAX
NARS1
GRP
RELCH
FBH1
RBM17
ANKRD16
ECHDC3
CCNH
HRH4
TMEM241
KCTD1
CABLES1
DOCK2
EMP1
SOX5
RERG
SPX
LRP4
MYBPC3
DDB2
RAB33A
RBMX2
STK26
PUM1
YARS1
PHC2
CDCA8
GNL2
HOOK1
CYP2J2
BTF3L4
TUT4
PRPF38A
DSC2
DSG3
RNF138
DSG1
DSC3
DSC1

DTNA
FHOD3
DAGLA
TIMM10
CBLIF
DHX34
APLNR
FADS2
TMEM258
C5AR2
TMEM165
CLOCK
PDGFRA
GGACT
CLDN10
UBAC2
ARGLU1
BIVM
TPP2
POGLUT2
CARS2
ARHGAP32
STT3A
ADAMTS8
ACRV1
ETS1
SLC37A2
KLB
TMED7
APC
NREP
WDR36
OSTF1
RFK
NAA35
C9orf40
ANXA1
CEMIP2
AGTPBP1
GOLM1
FAM189A2
PSAT1
ISCA1
HAVCR2
CCNJL
USP30
SDS
MSI1
HNF1A
FBXO21
TBX3
OASL
RNFT2
P2RX4
BICDL1
TRAFD1

DMTF1
TMEM243
CCDC146
TMEM60
CD36
PNPLA8
FAM71F1
RINT1
KCP
TES
MTO1
ADGRB3
ANKRD6
HTR1B
CEP162
SNX14
NT5E
MRAP2
EPHA7
AKIRIN2
ORC3
MAP3K7
CGA
PRR5L
LMO2
PHF21A
NAT10
EHF
ELF5
PRRG4
CAPRIN1
DNAJC14
CD63
PRPH
AVIL
AMHR2
GDF11
GLS2
ITGA7
TESPA1
FAM186B
RDH5
AGAP2
BLOC1S1
KRT85
CDK4
PPP1R1A
TSPAN31
B4GALNT1
COQ10A
FAIM2
PAN2
ESPL1
KRT7
ZC3H10
HNRNPA1

SLC26A10
ACVR1B
OS9
MIP
KCNH3
LTV1
MAP7
CD164
AFG1L
NHSL1
HEY2
PKIB
NMBR
SMPD2
REPS1
STX11
TEC
PRADC1
SEMA4F
CCT7
EGR4
RAB11FIP5
SMYD5
DYSF
EMX1
KCNMB4
USP15
GNS
CPM
MDM2
KLHL36
BCO1
MPHOSPH6
KIAA0513
DYNC1LI2
FBXL8
FHOD1
CCDC102A
SLC9A5
AGT
KCNK1
URB2
COG2
ABCB10
NTPCR
TAF5L
STX6
RGS8
RNASEL
DHX9
CEP350
NPL
NIBAN1
PIGC
LAMC1
RC3H1

GPR55
MRPL44
CHRND
PAX3
DOCK10
TTLL4
USP37
HTR2B
ITM2C
SLC19A3
SERPINE2
DNAJB2
WNT10A
TMBIM1
EIF4E2
ARMC9
CAB39
COX5B
REV1
TSGA10
TMEM127
EDAR
TGFBRAP1
GCC2
MRPS9
GPR45
C2orf49
EPC2
ISCU
STAB2
USP44
SCYL2
CKAP4
PLXNC1
APPL2
PWP1
DRAM1
WASHC4
SLC41A2
PCDH8
VPS36
CKAP2
CNMD
TBC1D4
THSD1
BORA
LRCH1
SUCLA2
MED4
PHF11
COG3
LMO7
ITM2B
SPRY2
NUDT15
EDNRB

RCBTB2
SETDB2
SCRN1
TNS3
CHST12
IGF2BP3
GPNMB
RAPGEF5
RAC1
KDELR2
NUP42
ZDHHC4
BZW2
TBRG4
DDX56
HUS1
DBNL
CCM2
MYO1G
TTYH3
TTC5
NKX2-8
NKX2-1
ZFHX2
IREB2
TM6SF1
CIB2
RSAD1
NMT1
MYCBPAP
SRSF1
VEZF1
CHAD
TACO1
TEX2
DCAF7
LIMD2
KAT7
ACTL6A
MRPL47
TRA2B
TBR1
MARCHF7
ERMN
GALNT5
SCN7A
TANK
SKIL
EPRS1
HLX
VPS45
IL10
KCTD3
RPS6KC1
IL1RN
IL36RN

IL1F10
SMPD4
WDR33
CCDC115
SAP130
BIN1
IMP4
HS6ST1
UGGT1
GYPC
STAM
ABI1
YME1L1
DNAJC1
NIPSNAP3A
LRRC8A
CDK9
TXN
ODF2
TOR1B
C9orf78
SMC2
KLF4
TOR1A
RALGPS1
NIBAN2
ST6GALNAC4
TMOD1
DAB2IP
STXBP1
SLC2A8
ANGPTL2
ZFP37
SLC31A2
SLC31A1
TLR4
ZNF189
ALDOB
STX17
PRPF4
FPGS
USP20
KIF12
ATP6V1G1
TEX10
GARNL3
MRPL50
DPM2
TSTD2
GABBR2
HEMGN
PSMB7
NR5A1
TRMO
RABEPK
GOLGA1

XPA
NCBP1
ANP32B
PDCL
RPL35
LMX1B
ARPC5L
ENPP2
DSCC1
DERL1
CCN3
RANBP6
POLR1E
PLAA
IL11RA
UBAP2
APTX
RNF38
TLN1
CCL21
SIT1
DMRT1
DNAJB5
DCTN3
CD72
TMEM8B
GRHPR
ALDH1B1
HINT2
ARHGEF39
IGFBPL1
DENND4C
RPS6
CNPY3
FOXP4
PPIL1
KLC4
KIF13A
PIM1
CMTR1
TFAP2A
SLC22A7
YIPF3
TMEM63B
FRS3
TJAP1
CAPN11
TINAG
IRF4
SLC22A23
LRRC1
GCM1
FOXF2
BPHL
RIPK1
HMGA1

TPMT
CLPS
RNF144B
TAF8
FAM8A1
FGFBP1
CPEB2
TLR2
TTC29
MYO7A
FCHSD2
ARRB1
SLCO2B1
THAP12
ANKRD42
IL18BP
NUMA1
CCDC90B
SYTL2
RAB30
CREBZF
LRRC32
PRCP
NARS2
RNF121
MRPL15
PI15
TTPA
GGH
SLCO5A1
SULF1
SDCBP
NEK1
SORL1
TMPRSS4
BUD13
MMP7
MMP20
MMP27
CFAP300
TRIM29
SLC37A4
BTG4
POU2F3
C11orf1
ARHGAP20
MMP13
TMPRSS13
ALKBH8
MAP2K5
UNC13C
CTDSPL2
SLTM
THBS1
MAPKBP1
NDUFAF1

KIF23
ITGA11
HAUS2
RTF1
PARP6
RPLP1
PAQR5
LRRC49
TUBGCP4
RMDN3
ITPKA
UACA
SMAD6
PLCB2
TMEM62
PAK6
ADAM10
CYP19A1
ZNF280D
SEMA6D
RSL24D1
GCHFR
BCAR3
TTLL7
FNBP1L
KYAT3
GTF2B
RABGGTB
GIPC2
ARHGAP29
SLC44A5
CLCA2
DNASE2B
DBT
RTCA
IFT172
SELENOI
CGREF1
HADHB
KHK
ADCY3
PNPT1
DYNC2LI1
LHCGR
THUMPD2
CYP1B1
RAB1A
ACTR2
PREB
SLC5A6
ABCG5
PREPL
SLC3A1
EMILIN1
FBXO11
SIX3

ATRAID
CENPO
LRPPRC
TRIM54
ACTR1A
MFSD13A
MYOF
LOXL4
STAMBPL1
CH25H
LBX1
KIF11
DUSP5
CALHM2
ARL3
CEP55
KIF20B
ENTPD1
EXOC6
PLCE1
RBP4
DBR1
DNAJC13
GPR87
ANXA7
FAM149B1
ASCC1
PLA2G12B
ZNF365
OIT3
ADAMTS14
RPS24
MYPN
ATIC
SMARCAL1
BARD1
STAT4
MSTN
CARF
ASNSD1
METTL5
SSB
NAB1
CDK15
PPIG
FASTKD1
MDH1B
HECW2
IDH1
OLA1
ITPRID2
CHRNA1
FAM117B
WDR12
ABI2
ITGAV

SLC40A1
SLC35A5
SLC49A4
CCDC54
COX17
MNS1
USP8
SECISBP2L
SPPL2A
GLCE
SHF
APH1B
INTS14
CILP
PARP16
PPCDC
HCN4
ARHGAP24
FAM13A
PCDH10
AP1AR
COPS4
HNRNPD
PRKG2
RASGEF1B
SEC31A
FGF5
BBS7
KIAA1109
BMPR1B
RAP1GDS1
MMRN1
PDE5A
PRDM5
NAAA
NUP54
CXCL9
BMP2K
G3BP2
FRAS1
CCNG2
USO1
CDKL2
ANXA3
PPA2
GSTCD
INTS12
ENPEP
CASP6
LEF1
HADH
C4orf17
SLC39A8
MTTP
FBN2
MAPK8IP3

RGS3
GUCD1
TTLL8
RNF185
SHISAL1
PARVG
B4GALNT3
ERP27
ETV6
KIF21A
YARS2
PLCZ1
AEBP2
SLCO1C1
ETFBKMT
ETNK1
ZCRB1
TMEM117
PRICKLE1
VAMP1
TAPBPL
CD27
PIANP
SLC38A4
AMIGO2
SCAF11
COL2A1
LLPH
LRIG3
MARCHF9
INHBE
GLIPR1
PHLDA1
TMEM19
LGR5
PTPRQ
DUSP6
POC1B
TMTC3
LUM
KERA
SNRPF
AMDHD1
NEDD1
SYCP3
ASCL1
GAS2L3
TMEM132B
SLC15A4
TDG
RITA1
SDSL
MMAB
GLTP
GIT2
TCHP

FAM222A
FOXN4
NUP58
MTMR6
SLC46A3
SLC7A1
PDX1
LNX2
SUOX
CCDC65
SLC39A5
TARBP2
RDH16
DHH
ACVRL1
GPR84
NABP2
N4BP2L1
CELA1
SMARCC2
CERS5
MAP3K12
ITGB7
GALNT6
CSAD
LMBR1L
MYG1
ESYT1
TMBIM6
ANKRD52
KRT71
ZNF740
WDFY2
LPAR6
ESD
RB1
SBNO1
SETD1B
VPS33A
VPS37B
RHOF
DENR
SLAIN1
RBM26
SRRM4
METTL21C
MBNL2
ZIC5
ABHD13
RAB20
GRTP1
SSTR1
CDH24
REM2
CBLN3
NOVA1

MDGA2
TMX1
FRMD6
RTN1
ARMH4
SYT16
SLC38A6
NAA30
RDH12
DCAF5
RAB15
WDR89
ESR2
STON2
EFCAB11
GPR65
PTGR2
JDP2
AK7
SLC24A4
FBLN5
WARS1
SLC25A47
WDR20
NIPA2
DUOXA1
MFAP1
TCF12
SORD
SERF2
ZSCAN29
DUOXA2
DUOX2
LYSMD2
SLC27A2
FGF7
HDC
GCNT3
BNIP2
GTF2A2
SRP14
BAHD1
DISP2
CDAN1
TLE3
ANP32A
COMMD4
UBE2Q2
PSTPIP1
ETFA
BCL2A1
HMG20A
SCAPER
TSPAN3
WDR61
NCOA2

MAN2C1
DNAJA4
TLNRD1
IGF1R
ARRDC4
PIF1
USP3
BBS4
PML
CYP1A1
LINS1
ULK3
PCSK6
SCAMP2
LMAN1L
RHCG
POLG
RLBP1
FANCI
ABHD2
NTRK3
DET1
MFGE8
ZNF710
UNC45A
ST8SIA2
MCTP2
FURIN
IQGAP1
EFL1
SEC11A
SEPTIN12
GLYR1
PMM2
SLC5A2
TGFB1I1
RUSF1
ARMC5
FTO
UQCRC2
CDR2
ABCC12
NKD1
DHX38
TXNL4B
MARVELD3
CHST4
CPNE2
KATNB1
KIFC3
ADAMTS18
NUDT7
CMTM3
CDH11
NOL3
MAP1LC3B

MBTPS1
ZCCHC14
MEAK7
ADAD2
OSGIN1
IRF8
RHOT2
RPS2
NDUFB10
PDPK1
TIGD7
DEF8
TCF25
GALNS
GAS8
MED9
NCOR1
COPS3
GID4
ZNF287
MYOCD
KSR1
RANBP10
CTRL
GFOD2
NOB1
UNC45B
PCTP
TOM1L1
KIF2B
TOB1
SPATA22
SGSM2
NPEPPS
SKAP1
LRRC46
SCRN2
SSH2
RHBDL3
SPACA3
ARSG
G6PC3
CLTC
C17orf64
BCAS3
PTRH2
SS18
TAF4B
AFG3L2
IMPA2
SLC39A6
RPRD1A
C18orf21
GALNT1
ASXL3
ADCYAP1

MEP1B
SLC25A52
GAREM1
ESCO1
OSBPL1A
GATA6
GREB1L
RMC1
PELP1
NPC1
SLC14A1
ARRB2
SLC13A5
ZMYND15
WRAP53
MINK1
SAT2
ASGR1
PIK3R5
TP53
ARHGDIA
TMC6
SLC16A3
CARD14
TTYH2
EIF4A3
CSNK1D
ANAPC11
TBCD
FN3KRP
NARF
RPTOR
FOXK2
TRIM65
CBX8
RNF157
CEP131
ZNF750
WDR45B
CBX4
RNF165
DYM
MAPK4
ELAC1
MBD1
SMAD4
FBXO15
P3H4
NT5C3B
RETREG3
ERBB2
GRB7
MIEN1
PNMT
STAC2
IGFBP4

FKBP10
TXNL4A
BRD4
SLC39A3
NFIC
TPGS1
PLPP2
PRDM15
FEM1A
VAV1
MVB12A
CIB3
SH3GL1
DUS3L
DPP9
DMRTC2
CCDC97
TMEM91
ZFP14
SIRT3
PGGHG
HUNK
COL6A1
SOD1
COL6A2
DNMT3L
TRPM2
SCYL1
APP
DOP1B
URB1
IL19
EMP3
SAE1
NTN5
GEMIN7
WTIP
ADAMTS10
RNPEPL1
CAPN10
TIMM29
CARM1
EVI5L
TM4SF5
PSMB6
SIGLEC10
ACP4
FAM71E1
RPS11
PTH2
NOSIP
IGLON5
RCN3
SLC2A5
RERE
PRDM16

EPHA2
ARHGEF19
EFHD2
PEX14
PGD
MYOM3
SH3BGRL3
CNKSR1
RPL11
IL22RA1
ZNF593
C1orf216
KIAA0319L
EVA1B
C1orf94
DMRTA2
PLK4
MAP3K6
GPN2
SYTL1
WDTC1
ITGB3BP
SERBP1
BCL10
CCN1
PIGK
TINAGL1
AZIN2
RPS8
KIF2C
PTPRF
BEST4
MOB3C
CYP4B1
LMO4
SYPL2
BARHL2
MTF2
SLC44A3
IGSF3
ZNF697
CTTNBP2NL
STRIP1
KCNA10
PSMA5
CD53
PROK1
CELSR2
ITGA10
GPR161
ALDH9A1
ATP1B1
TIPRL
NME7
POGK
MPC2

CREG1
DCAF6
GPA33
TBX19
UCK2
POU2F1
MAEL
ILDR2
DPT
MGST3
ADCY10
COP1
NECTIN4
UFC1
PPOX
NUF2
RGS5
SDHC
PFDN2
NR1I3
USP21
F13B
PRCC
RRNAD1
MRPL24
PIGM
CASQ1
ISG20L2
CRABP2
HDGF
ABL2
XPR1
RGS16
FAM163A
RGL1
LYPLAL1
LHX9
PRUNE1
RORC
TUFT1
SF3B4
ZNF687
TARS2
CGN
SNX27
SETDB1
ADAMTSL4
MCL1
CTSK
RFX5
PI4KB
PIP5K1A
ANP32E
MINDY1
ANXA9
SELENBP1

CERS2
ENSA
SEMA6C
MRPL9
ARNT
POGZ
C1orf56
HORMAD1
GABPB2
SYT14
DTL
DYRK3
EIF2D
INTS7
VASH2
TAF1A
SMYD2
SUSD4
DUSP10
TP53BP2
ATP8B2
ADAM15
JTB
S100A8
NUP210L
SNAPIN
UBAP2L
SLC39A1
HAX1
CREB3L4
AQP10
C1orf43
ILF2
RIT1
C1orf131
GALNT2
TTC13
SCCPDH
LYST
ACP1
SNAP47
SRP9
NVL
DEGS1
FBXO28
ARF1
CNIH4
GUK1
CDC42BPA
C1orf35
MBOAT2
PARP1
PSEN2
LBR
WNT9A
EPHX1

REN
SOX13
ETNK2
PPFIA4
PLEKHA6
PTPN7
OSR1
GDF7
PDIA6
RHOB
ATP6V1C2
HNRNPLL
GALM
CAMKMT
ABCG8
EML4
CALM2
CHAC2
RPS27A
VPS54
ASXL2
ETAA1
SNRPG
ABHD1
CIAO1
SNRNP200
MRPS5
ANKRD53
TPRKB
NAT8
SFXN5
TEX261
DQX1
DUSP11
MALL
THNSL2
C1QL2
TMEM177
SLC20A1
ZC3H8
LIPT1
AFF3
UBXN4
NXPH2
SPOPL
THSD7B
GPR17
POLR2D
AMMECR1L
SCRN3
LNPK
ZNF385B
TMEFF2
CDCA7
DLX1
UBR3

PHOSPHO2
GULP1
FAM171B
HSPD1
METTL21A
UNC80
PTH2R
NBEAL1
KANSL1L
SPAG16
RHBDD1
ACKR3
TRPM8
HES6
COPS7B
DIS3L2
CPNE9
FANCD2
VGLL4
RAB5A
RETREG2
CNOT9
MARCHF4
GMPPA
GRIP2
EAF1
CNTN4
DYNC1LI1
GADL1
OSBPL10
POMGNT2
ACKR2
GASK1A
CSRNP1
SLC25A38
ITGA9
GOLGA4
CTDSPL
STAC
IQSEC1
CAND2
RPL32
PTPRG
IL17RD
UBA3
ARL6IP5
TMF1
LRIG1
LRTM1
LIMD1
NFKBIZ
COL8A1
NXPE3
ADGRG7
PHLDB2
ABHD10

PLA1A
RABL3
ADPRH
IGSF11
ATG3
NR1I2
BOC
SRPRB
TMEM108
AGTR1
EIF2A
OSBPL11
TRPC1
NCEH1
SPATA16
LPP
RUBCN
AMT
TCTA
NICN1
UCN2
DCAF1
MANF
EAF2
ILDR1
TM4SF19
SLIT2
EIF2B5
AHSG
VWA5B2
DGKQ
FIP1L1
SLC26A1
LYAR
CORIN
ATP10D
OCIAD2
SLC10A4
SLC10A6
PLAC8
ENOPH1
GC
TRMT10A
KLHL8
PYURF
TBCK
CISD2
DDIT4L
TIFA
SPATA5
FABP2
CCNA2
METTL14
SETD7
NAF1
MARCHF1

SFRP2
RPS3A
PDGFC
CBR4
CYP4V2
ROPN1L
NDUFS6
MARCHF6
SRD5A1
MYO10
OTULINL
RPL37
SKP2
PLK2
SHISAL2B
GZMA
PIK3R1
HAPLN1
LHFPL2
SSBP2
BHMT
CRHBP
RASA1
LIX1
GIN1
PPIP5K2
PAM
SLC30A5
BTF3
FBXL17
SPATA9
TNFAIP8
FEM1C
COMMD10
ATG12
MEGF10
ADAMTS19
YIPF5
ARHGAP26
CXCL14
LECT2
SLC25A48
DDX46
TIMD4
RNF145
C1QTNF2
GABRA6
FBXO38
SPINK7
PCYOX1L
G3BP1
N4BP3
NHP2
RMND5B
BOD1
CPLX2

KCNMB1
MYLK4
TBC1D7
FARS2
GFOD1
PSD2
LRRTM2
ZMAT2
GFRA3
DCDC2
KAAG1
TRIM7
TRIM41
HIGD2A
FAM193B
PLA2G7
TNFRSF21
RNF44
MMUT
DOK3
ABT1
DAAM2
PRIM2
MLIP
HMGCLL1
LGSN
FGD2
SCUBE3
CRIP3
TTBK1
RPL7L1
IRAK1BP1
PHIP
PRSS35
MMS22L
FAXC
PNRC1
PM20D2
RARS2
SCML4
TBC1D32
GPR6
RNF217
RSPO3
ARHGAP18
ABRACL
TAAR1
SLC18B1
MTFR2
SLC2A12
SHPRH
AIG1
DYNLT1
TIAM2
TMEM181
PNLDC1

WTAP
ZMYM4
VIP
ARMT1
C7orf50
C7orf26
RBAK
CREB5
FERD3L
CDCA5
IGFBP3
IGFBP1
SSC4D
MDH2
NIPSNAP2
CCT6A
PSPH
TRIM50
ATXN7L1
TMEM168
ASB15
TRAPPC14
SLC12A9
GIGYF1
MEPCE
TMEM209
AGBL3
STRA8
ZC3HAV1L
TMEM140
NOM1
CNPY1
NCAPG2
ASB10
RAB19
DENND2A
CLTRN
SH3KBP1
TMEM47
LANCL3
SYTL5
CASK
KDM6A
AKAP4
SLC16A2
DIPK2B
ZNF182
NDUFB11
ZMYM3
TAF1
GPR174
NONO
CCDC120
EBP
AWAT2
OGT

ITGB1BP2
ZNF711
RIPPLY1
PRPS1
FRMPD3
HTR2C
IGSF1
ARHGAP36
GPC3
GPR119
RBMX
FBXO25
CETN2
ATP6V1B2
CCDC25
HMBOX1
CHRNB3
CHRNA6
GNRH1
BIN3
DOK2
SLC25A37
CHMP7
DOCK5
STAR
PLPBP
ERLIN2
SNTG1
ST18
RGS20
TACC1
GOLGA7
PLPP5
GINS4
NSD3
DNAJC5B
CRH
TRIM55
ADHFE1
LACTB2
RPL7
SLC26A7
ATP6V0D2
SYBU
DPYS
MTDH
LRP12
EBAG9
RSPO2
POLR2K
MAL2
EIF3H
UTP23
NDUFB9
TATDN1
FAM83A

ZNF7
ARHGAP39
VLDLR
AK3
UHRF2
NFIB
CER1
PLIN2
CDKN2B
C9orf72
ZCCHC7
FBXO10
SIGMAR1
CEP78
NTRK2
IDNK
SHC3
AUH
ZNF462
INIP
UGCG
ACTL7B
SNX30
STOM
GSN
MRRF
NR6A1
CRB2
OR5C1
ALAD
WDR31
POLE3
GBGT1
GTF3C5
ASB6
PTGES2
NTMT1
CIZ1
SLC25A25
SH3GLB2
MIGA2
PTGES
LCN2
LRSAM1
GPR107
PAXX
INPP5E
NOTCH1
NACC2
PROSER2
USP6NL
COMMD3
MSRB2
PDSS1
FAM171A1
MINDY3

SLC39A12
TMEM236
RSU1
ST8SIA6
PARD3
ZEB1
FAM13C
NRBF2
A1CF
CDHR1
LRIT1
RGR
POLR3A
HERC4
LRMDA
GLUD1
ANKRD1
HTR7
RPP30
FRA10AC1
ADD3
HABP2
VAX1
DNAJB12
EIF4EBP2
NPFFR1
TCF7L2
CYP17A1
INA
MTG1
PAOX
GSTO1
TAF5
PPRC1
ITPRIP
CNNM2
PDCD11
ADAM12
RGS10
BTBD10
ADM
GAS2
SLC5A12
LRRC4C
IMMP1L
PGAP2
TUT1
HSD17B12
APIP
PAMR1
DGKZ
EIF3M
GLYAT
SERPING1
SSRP1
SLC43A1

C11orf49
ARFGAP2
CELF1
HIKESHI
SESN3
ENDOD1
CCDC82
KLHL35
SERPINH1
CAPN5
INTS4
RPS3
ZC3H12C
TTC12
NCAM1
DRD2
C11orf52
HTR3B
NPAT
ATM
AASDHPPT
GLB1L2
SLX4IP
LAMTOR1
P4HA3
ST14
ADAM33
SLC22A8
KAT14
TKFC
MTA2
TMEM138
FADS1
TMC2
ROM1
EML3
CPSF7
B3GAT3
EI24
CHEK1
FEZ1
ESAM
KIRREL3
MPZL2
SCN2B
SIDT2
TMEM25
TAGLN
JPH2
DUSP15
COMMD7
KIAA1755
SPATA25
OCSTAMP
DSN1
SOGA1

CNBD2
CDH22
LSM14B
YTHDF1
CABLES2
LTO1
GPHA2
TRPT1
NUDT22
FERMT3
PLCB3
MRPL49
CDC42EP2
FAU
TM7SF2
VPS51
TBX6
PPP4C
ALDOA
TLCD3B
DOC2A
TAOK2
TMEM219
HMGA2
MMP3
CNKSR2
CLEC1A
MKX
MPP7
ITGB1
CTF1
CWC15
ARID5B
TMCO3
TMEM218
TIRAP
EEF1AKMT1
SAP18
HNMT
LYPD1
LYPD6B
PDCD4
ADRA2A
GPM6A
WDR17
SPATA4
VEGFC
CNDP1
FSIP1
CCDC83
RGS18
PRSS23
MTMR12
PPP1R1C
C11orf53
CCT5

ATPSCKMT
DIXDC1
DLAT
PIH1D2
NKAPD1
TIMM8B
IL18
TEX12
PTS
FREM2
FOXO1
CRIM1
SEC24D
ABCB9
RILPL2
DHX37
PRSS53
SLC7A11
NOCT
ENKUR
GPR158
KCNA6
NGLY1
OXSM
UEVLD
TMEM86A
C12orf45
TMEM263
BTBD11
UBE3B
IPMK
RAD9B
PLBD2
SLC2A13
GXYLT1
TWF1
EIF4E
TEX30
SRFBP1
AKAP6
NPAS3
FAM177A1
MBIP
MIPOL1
EXT2
TMEM18
ALLC
KCTD14
ME3
MSGN1
ADAMTS12
NUBPL
FER
VIPAS39
ANKRD50
UPF2

CDC123
SCLT1
CCDC3
C4orf33
FRMD4A
PTPRO
EPS8
ACAD8
THYN1
VPS26B
VTI1A
QDPR
FAM160B1
QTRT2
DRD3
MMAA
ZNF827
POU4F2
EDNRA
NR3C2
ITIH2
KIN
PIGF
ANKAR
INPP1
MFSD6
RNF144A
ASAP2
ADAM17
FLI1
KCNJ1
TMEM45B
WWC2
ACSL1
SLC25A4
BICD1
SAV1
CCDC122
NBAS
ZNF385D
GUF1
SLC35F4
SACS
FBXO4
TMEM267
CCL28
PARP8
GFRA1
CACUL1
BEND6
TIAL1
BAG3
GLT1D1
RBM46
KCNE4
AP1S3

RABGAP1L
TLCD4
MZT2B
ASTN1
FAM168B
PTPN14
MGAT5
TMEM163
GPATCH11
HSPB8
GEMIN6
TMEM178A
OBI1
CYSLTR2
GRID2
SETBP1
ARL14EP
EPG5
PSTPIP2
ATP5F1A
HAUS1
C18orf25
PDK1
PTH
PDE3B
TCF7L1
SH2D6
UHMK1
ATG10
POC5
SPOCK1
FAM151B
TADA1
CWF19L2
JMY
HOMER1
XRCC4
BOLL
SUV39H2
DCLRE1C
RPP38
NMT2
CCDC50
CAMK4
TRIM36
ZFP36L2
PAN3
PLEKHH2
PFKM
SPARCL1
DMP1
MBNL1
CAPSL
NADK2
GJA1
CCNO

DDX4
SLC30A6
PELO
RASGRP3
SAR1B
CATSPER3
GPR180
DYNLT5
ANKRD22
FARP1
SLC16A12
PRDM8
BMP3
HNRNPDL
HHEX
MED21
PLOD2
NRSN1
ZIC1
ADGRA3
CPB1
SREK1IP1
LGI2
CWC27
SRP19
CDYL
CARHSP1
TEKT5
TXNDC11
DAB2
ACMSD
BCL2L11
ANAPC1
CAST
SCOC
CLGN
CETN3
BMP6
RASSF3
HNRNPU
RANBP2
MERTK
TMEM87B
PTPRR
NR4A2
CCDC148
PLA2R1
RBMS1
FEZF2
SLC25A27
CYRIB
ASAP1
TRAPPC8
FAM81B
INO80C
LPCAT1

UBALD1
ING1
CMTM7
FBXL2
UBP1
RMND5A
RPIA
LURAP1L
GTF2E1
CFDP1
ZDHHC7
CIBAR2
C7orf31
TMPRSS11D
JAZF1
CMIP
SPHKAP
KCNJ16
PID1
TRIP12
FBXO36
CEBPG
KCTD15
MCOLN2
LGI4
DDAH1
SREK1
CHD1
ANKFN1
DGKE
HS2ST1
CACNA2D1
ZUP1
GDPD1
NUS1
SEMA3D
ASB17
GRAP
AK5
CABYR
IMPACT
ANKRD29
SDHAF4
CHST9
THY1
DNAAF1
C16orf74
TBCEL
JPH3
ANKH
OTULIN
UBASH3B
ROBO4
ROBO3
PANX3
TBRG1

NRGN
GPR15
TOMM70
ANGPT1
PITPNC1
CC2D1B
CERS3
LRRK1
ABCA5
ENPP3
C4orf19
UCHL1
DISP1
FAM167A
NEIL2
LONRF1
TRIM11
ENAH
PPP1R3A
CCSAP
ASZ1
SH3RF1
BUB3
GPR26
CCDC173
DIPK1A
ATP5MC3
SRSF12
PDLIM3
SORBS2
ELOC
LY96
CXADR
BTG3
C21orf91
CHODL
TMPRSS15
NCAM2
PDE1C
RABGEF1
MRPL39
JAM2
ATP5PF
GABPA
ADAMTS1
ADAMTS5
TSEN2
CCDC174
FLCN
DPH3
OXNAD1
CXXC1
APCDD1
PIEZO2
MPPE1
USP43

EME1
ACSS1
ANKRD40
ZNF18
CA10
VOPP1
APOOL
DKK2
CYP2U1
FBXL18
PROM2
AK9
ODF1
KLF10
AZIN1
ATP6V1C1
PIP4P2
OTUD6B
CDK19
GTF3C6
MMS19
ZFYVE27
GOLGA7B
TRMT44
SLC25A28
HSPA13
SAMSN1
USP25
ZCCHC10
MOV10
RHOC
PPM1J
HEATR3
NIFK
OXA1L
LARP1
SETD9
MIER3
NUP205
ZKSCAN2
C9orf85
PIK3AP1
RBM45
PDIA4
KDM8
OTOA
FAM126B
FLACC1
TMEM237
FZD7
DEPTOR
RNF20
CYLC2
PPARGC1B
SLC26A2
LSM11

MED7
SAXO1
RRAGA
SLC24A2
TRIM42
PXYLP1
ADCY8
RASA2
RMND1
SLA
TMBIM4
VBP1
RAB39B
AFF2
MICU3
GRIP1
VPS37A
KIF5A
PSD3
CARNMT1
MCU
MIDEAS
CFAP70
FAM161B
WIF1
GPR61
ADK
KCNMA1
BATF
DCK
ADAMTS3
ALX3
DPY19L4
NDUFAF6
DRAM2
C8orf37
PPEF2
CFAP161
ADAMTSL3
ART3
N6AMT1
RWDD2B
USP16
CCT8
MAP3K7CL
BACH1
CLDN17
CLDN8
TSPAN7
TIAM1
SCAF4
CDK20
PCGF6
SFR1
SORCS3
SFXN2

ATP5MJ
TDRD9
FGF18
PCDH1
SH3RF2
GDF6
UQCRB
MTERF3
PTDSS1
RPL30
KCNS2
SUPV3L1
EEF1A1
TYSND1
PHF6
CD109
LRFN2
NODAL
ZDHHC5
MED19
ZFAND3
NPTN
SAMD8
UNC5D
UTP14A
AIFM1
MAPK13
BAG4
MS4A1
TBC1D31
NTAQ1
ATAD2
FBXO32
NSMCE2
ZNF689
PRR14
FBRS
PHKG2
SASS6
COX6A2
ZIC3
MALSU1
VPS8
GALK2
B3GNT7
BUB1B
PDE6D
EIF4A2
SST
TATDN2
GHRL
EXOG
SHCBP1L
NMNAT2
ZFYVE9
LYZL4

SLC6A1
SMG1
FCHO2
RBPMS
TMEM171
KLHL40
C8A
TIMP4
SYN2
ODR4
CPT2
NECAP2
LRP8
CDCP2
PAXIP1
STEAP2
SSBP3
HTR5A
CLDN12
MMP14
FZD1
GATAD1
TMED6
C1orf158
ARMC12
ST3GAL2
FCSK
IL34
KIT
AASDH
CACNA2D3
RNF111
CCNB2
FAM81A
APPL1
PWWP3B
AFAP1L1
TSC22D3
VPS26C
DYRK1A
KCNJ6
KCNJ15
ERG
ETS2
SLC35B2
TMEM164
MX1
CREB3L1
TAB3
SLC38A10
PALM2AKAP2
ZNF618
TMEM268
SVOPL
SNX22
UBN2

BRAF
ACAN
PSMG3
CABP1
WDR19
SLC37A3
GAREM2
SPPL3
DPYSL5
DRC1
RAB28
CIB4
PEX10
RER1
RADIL
SKI
WIPI2
LDLRAP1
KRTCAP3
ANKRD61
PAFAH2
EXTL1
SLC30A2
TRIM63
MRPL17
DUSP2
GRHL3
UBXN11
NLRP14
PTPDC1
GALNT14
NCK1
HPD
RHPN1
TPRG1L
LRRC43
PRXL2C
XDH
XKR8
CNNM4
EYA3
FANCC
MRAS
WASF2
ABHD3
ESYT3
FAIM
TENT5B
CLSTN2
COLEC12
CUL4B
GPR153
SLC13A3
GPRASP2
RHBDL2
AUTS2

SHROOM4
CDC25C
MITD1
EIF5B
RIBC1
CATIP
CNOT11
KCNB1
TSPAN33
NRG2
B4GALT5
FGF19
PDZK1IP1
TAL1
CMPK1
BEND5
ELAVL4
COA7
ZYG11B
SLC1A7
CZIB
MAGOH
ACOT11
FAM151A
PARS2
LEXM
BSND
USP24
PLPP3
KLHL21
GMEB1
SLC45A1
SELENON
AK4
JAK1
CTRC
LZIC
KNCN
TMEM82
SLC25A34
DRAXIN
LRRC38
DHRS3
MATN1
LAPTM5
SDC3
PEF1
SYNC
RBBP4
KIAA1522
TSSK3
TMCO4
UBXN10
ALPL
WNT4
MEGF6

NFIA
OMA1
MYSM1
TM2D1
USP1
FUBP1
NEXN
DNAJB4
ADGRL4
TYW3
LHX8
B3GALT2
NTNG1
FAM102B
HENMT1
C1orf52
DNAI3
ATXN7L2
ZNF326
HFM1
GFI1
AGL
VCAM1
EXTL2
SLC30A7
ZNF281
ARPC5
CADM3
NLRP3
TRIM58
SLAMF9
KCNJ9
IGSF8
DDR2
PEA15
PEX19
NCSTN
VANGL2
OLFML2B
FCRLB
KLHDC9
C1orf74
LMX1A
LRRC52
FLVCR1
ATF3
RBM15
DENND2D
TDRD5
SNED1
BPNT1
SPATA17
C1orf115
BROX
ACP6
WDR64

KIF26B
TFB2M
PPP1R21
KLHDC8A
PM20D1
PKDCC
HAAO
MAPKAPK2
IL20
IL24
FCMR
PIGR
CAPN2
MRPL55
REL
PUS10
PEX13
KIAA1841
RFTN2
LRRTM1
MEMO1
DPY30
TYW5
MAIP1
KCNF1
SLC66A3
ARL5A
LRATD1
KCNJ3
NEUROD1
CLHC1
FRZB
DUSP19
CFAP36
NUP35
CCDC138
ZSWIM2
FBXO41
SMC6
H3-3A
COQ8A
SLC16A14
TEKT4
EN1
SGCB
NOSTRIN
CFAP221
SGPP2
INHBB
SMARCAD1
HPGDS
PDLIM5
NEURL3
RPRD2
ANKRD23
CTSS

MSX1
PACRGL
BNIPL
C1QTNF7
TNFAIP8L2
LYSMD1
SCNM1
TMOD4
VPS72
ERCC3
IWS1
BOLA3
CDC42EP3
S100A11
DHX57
BMP10
ARHGAP25
S100A9
TGFA
CCNYL1
FZD5
DCAF16
C1orf189
NPPC
GNPDA2
GABRG1
PAQR3
NIPAL1
ALPI
ANTXR2
HELQ
MRPS18C
CGGBP1
ABRAXAS1
GPR155
PMVK
PBXIP1
CLDN1
PYGO2
HIPK1
LENEP
DCST1
INAVA
KBTBD8
EOGT
LMOD3
NAXE
POGLUT1
SLC22A15
CCKAR
ATP1A1
SLC15A2
EIF4E3
PROK2
LRRC58
FSTL1

ELF3
PDCL2
TMEM183A
TMEM169
IGFBP7
TRIM46
KRTCAP2
ARPC2
CCT3
TMEM79
SSR2
RNF25
STK36
ADORA1
NEK10
CCDC141
FEV
CRYBA2
IHH
CIP2A
EOMES
CWC22
AZI2
TGFBR2
ANKZF1
HDAC11
TRAT1
FBLN2
GLB1L
STT3B
CHCHD4
NFASC
SERPINI1
CLASP2
SUCLG1
NUAK2
SPTA1
PRKCI
SLC2A2
FABP1
PPM1L
ICA1L
CTLA4
ICOS
PPP4R2
CD200R1
GTPBP8
NEPRO
CCDC191
NKX6-1
WDFY3
COX18
SYNPR
ALB
C3orf49
THOC7

ATXN7
PSMD6
PRICKLE2
ADAMTS9
PPM1K
CLRN1
GMPS
TIPARP
CCNL1
PTX3
HESX1
DCLK3
SLMAP
RPL9
SMIM14
RPP14
ABHD6
DNASE1L3
RBM47
APBB2
IL17RE
IL17RC
CRELD1
PRRT3
FANCD2OS
PCOLCE2
U2SURP
MTMR14
TTC14
RCHY1
CCDC158
CPA3
GYG1
HPS3
TOPBP1
SNRK
TCF23
DNAJC5G
UCN
ZNF513
SPDYA
WDR43
ZDHHC3
CLEC3B
SLC6A20
LZTFL1
FYCO1
CCR1
RTP3
LRRC2
ELP6
FBXO40
ZNF148
NMNAT3
SMIM12
ZMYM6

TPRA1
YEATS2
ZC3H12A
MEAF6
SNIP1
DNALI1
POLR2H
KLF15
LIPH
TMEM41A
RPN1
SENP2
HEYL
IFT122
RHO
RFC4
BAP1
TKT
PRKCD
RFT1
SFMBT1
GNL3
PBRM1
UVSSA
TASOR
ARHGEF3
SLBP
LRPAP1
SLC51A
UBXN7
RNF168
MELTF
OTOP1
EXO5
CLDN19
C1orf50
ERMAP
ZNF691
AIMP1
SGMS2
METAP1
DNAJB14
H2AZ1
SLC9B2
BDH2
PGRMC2
CDC25A
PLXNB1
CCDC51
ATRIP
SHISA5
SPRY1
BSN
APEH
INTU
RNF123

HSPA4L
MFSD8
ABHD18
CAMKV
MON1A
MST1R
RAD54L2
TEX264
GRM2
DUSP7
POC1A
PPM1M
ETNPPL
WDR82
PITX2
HMGB2
SAP30
SCRG1
HAND2
MAD2L1
ANXA5
ADAD1
GUCY1A1
FBXO8
CEP44
HPGD
ASB5
C4orf45
TMEM144
NPY1R
NPY5R
NAA15
IL15
FAM160A1
ARFIP1
HHIP
ANAPC10
ABCE1
OTUD4
LSM6
TMEM184C
PRMT9
ITGA2
MOCS2
SLC45A2
EDIL3
TMEM161B
ELOVL7
NDUFAF2
ZNF474
LMBRD2
RANBP3L
NIPBL
RNF180
SLC25A46
STARD4

PGGT1B
CCDC112
ANKRD33B
CMBL
C5orf63
PRRC1
F2RL1
AGGF1
WDR41
NDUFS4
SCGB3A2
SPINK1
HTR4
ESM1
GRPEL2
ARSK
RHOBTB3
GPX8
SERINC5
ENPP6
CASP3
PRIMPOL
ERAP1
EGFLAM
CFAP97
TMEM174
CARTPT
RICTOR
TENT2
ANKRA2
UBLCP1
FAM170A
TLR3
NSA2
GFM2
SLC6A18
CCDC127
ACSL6
SEPTIN8
GDF9
UQCRQ
LEAP2
SLC35A1
FABP7
CITED2
TBXT
CREBRF
SFXN1
SAMD3
TMEM200A
IL22RA2
PDSS2
SPATA48
STXBP5
ANKRD55
PI16

TBX20
DAGLB
KIAA0895
TRA2A
GALNT10
SAP30L
RPS14
MYOZ3
NEUROD6
BMT2
GPR85
SLU7
RP9
PTTG1
CAMLG
BMPER
RELL2
KCNK5
ZNF12
SLC29A4
TEX47
STEAP1
CDCA7L
SP8
MIOS
ELAPOR2
USP49
SYTL3
HEY1
ZNF704
FABP5
COL1A2
CHMP4C
SLC13A4
PGAM2
BRI3
SOX17
DLC1
C8orf48
SUN3
C7orf57
HNF4G
PEX2
RAD21
SLC30A8
MED30
TNFRSF11B
SBSPON
PHKG1
EN2
KCNV1
ORC5
DNAAF5
OSGIN2
OXR1
TMEM74

GPR146
GPER1
TMEM184A
NOS3
CA3
INTS1
CDK5
SLC4A2
FASTK
TMUB1
FMC1
GBX1
PHAX
ALDH7A1
FOXK1
COX6C
OSR2
YWHAZ
BAALC
FZD6
CTHRC1
SLC25A32
DCSTAMP
TP53INP1
INTS8
VIRMA
FREM1
GEM
PDP1
TMEM67
WASHC5
RPP25L
FAM219A
C9orf24
SNAPC3
MYORG
NUDT2
TMEM65
PSIP1
CCDC171
UBAP1
DIRAS2
SYK
ABCA1
NFIL3
LETM2
FXN
ZMAT4
NKX6-3
MAMDC2
PRSS37
CPA6
TMC1
ALDH1A1
KDM1B
HGSNAT

GKAP1
KIF27
C9orf64
HNRNPK
SSMEM1
SVEP1
ANKS6
PGAP4
ZHX1
CYBB
DYNLT3
METTL27
MID1IP1
SHOC1
CXorf58
KIAA1958
PTCHD1
RNF183
ASB11
PCDH19
PIGA
VEGFD
STRBP
CLDN3
GAPVD1
CARD19
ZNF367
HDX
NDUFB6
AQP7
NOL6
AQP3
TRMT10B
VCP
PIGO
STOML2
SLITRK5
MELK
ARMC3
OTUD1
ARHGAP12
FAT3
DEUP1
HECTD2
SLC7A3
FBXO33
GPR101
CLDN2
LRFN5
LRRC18
SPTSSA
WRN
MARCHF8
TSHR
CFL2
SUGT1

GTF2A1
ZCCHC24
PGM2L1
SLC16A9
INPPL1
PHOX2A
MBL2
GJB2
CRYL1
REEP3
HEPACAM
SKA3
MICU2
DDIAS
PCF11
PKNOX2
LRR1
DEPP1
ZNF22
KLHDC2
NEMF
RPUSD4
ARF6
TTC8
TMEM63C
NGB
NOXRED1
CDX2
OTX2
DRGX
NUDT5
DACT1
OXGR1
UCMA
BEND7
ATP5F1C
PRPF18
TAF3
VSTM4
COMTD1
SLC18A2
PDZD8
ZNF503
ABRAXAS2
QSOX2
FAM204A
NSD1
PRDX3
GHITM
CLEC1B
TMEM52B
PMPCA
ENTR1
FRMD7
AK8
SPACA9

TSC1
GFI1B
HPRT1
DIPK1B
ZMYND19
STOX1
RET
DDX21
BMS1
STK32C
FUNDC2
PIP4P1
METTL17
SLC39A2
NDRG2
ARHGEF40
NSMF
ZNF219
C12orf50
CASP7
PPP1R36
BTNL9
CCDC186
VWA2
METTL3
SALL2
TRUB1
ZFYVE1
C10orf82
HSPA12A
UBTD1
ANKRD2
E2F7
ARHGAP42
ISCA2
OTOGL
LARGE2
PACSIN3
TTC7B
SLC39A13
TTC39C
GLYCTK
UBTD2
NKIRAS2
DNAJC7
KCNV2
IRF2BP2
PTF1A
NT5DC2
FOXI1
SMIM4
COA6
MGAT2
THAP11
MMADHC
PDHB

PXK
PCMTD1
KCTD6
MPLKIP
ACOX2
FAM107A
IRF2
MOBP
CX3CR1
XIRP1
INSM2
DEGS2
ARF4
SEPTIN2
FILIP1L
MFSD2A
DTYMK
ING5
ATG4B
BDKRB2
RHOH
KLHL30
COG7
CDC40
STIP1
SCNN1B
HR
TXNDC2
REEP4
LGI3
SFTPC
ATXN2L
PHYHIP
CCDC110
POLR3D
FEN1
CAVIN2
MTCL1
GBX2
HJV
FNTA
SERINC2
MYL1
TRAPPC11
CHRM1
COL3A1
GFRA2
ING2
CDKN2AIP
SNRNP48
TMEM223
SLC20A2
DYNLRB2
TMUB2
STAT3
ZSWIM1

ADAM9
GDNF
AXIN2
NDUFS5
VWA3B
LRATD2
KCTD19
SLC16A4
IL7R
TMEM208
AHCYL1
DNAJC21
PKIG
NPNT
CA7
SEMA4C
CNNM3
CXXC4
TCTN2
SHOX2
PPIP5K1
TSPAN5
ABHD15
ZBTB5
CHTF8
ADAL
LCMT2
SNTB2
ZNF507
STX18
NSG1
ZBTB49
GFM1
OR13J1
ATOH8
SOX14
ANKRD49
SFTPB
USP39
TNIP2
C2orf68
TMEM150A
VAMP5
LRRC28
MAT2A
PLA2G4F
ENHO
ZNF608
SLC35G2
LETM1
TMEM129
PPIC
SPRY3
CEP120
STXBP6
TM4SF20

MFF
JMJD7-PLA2G4B
PXDC1
E2F6
FEM1B
COMMD8
ATP5ME
UQCRFS1
SLC49A3
COL4A3
KLK7
HNRNPH1
IRS1
MECP2
ROR2
AR
VXN
HSPBAP1
CHST14
FAM110B
ODAD2
AFAP1L2
ATF5
GOT1L1
ZBTB43
CPT1C
XPO6
GSG1L
MN1
APEX2
NSMCE1
CCDC126
CD2BP2
RSPO1
RGS14
TBC1D10B
LMAN2
RAB24
PRELID1
THBS3
SLC50A1
EFNA1
CXCL10
SH3TC2
CXCL11
ZRSR2
NMD3
ADRB2
B3GALNT1
HSPB3
MRPL1
SHE
NR0B1
PGM2
STK32A
IL1RAPL1

P2RY12
MINAR1
GP2
SLC33A1
SNUPN
CRADD
SIN3A
ARL13B
PTK2
PTAFR
PTPN9
NPR1
RASSF6
SDC2
MMGT1
TM2D2
HTRA4
PLEKHA2
SLC38A11
GPR183
CRCT1
METTL15
MUC15
ZEB2
GJB1
PCBP1
HINT1
DTWD2
VPREB1
CLIC3
INO80E
BNC1
DFFB
NFU1
ANTXR1
GKN1
CKAP2L
C15orf40
PROKR1
APLF
HIC2
LUZP1
HEXD
DRD5
BUB1
SPNS1
LRRC45
CHRNA5
CENPX
AGPAT2
ASPSCR1
GP9
FASN
CNBP
ACTRT2
DUS1L

GPS1
RFNG
DCXR
ZNF32
TMEM266
NLGN1
TAPT1
UGP2
LINGO1
HNRNPF
BTD
CSGALNACT2
GSX1
PCDH7
ROBO1
ONECUT1
AVEN
P2RY1
TRIM56
AHSP
WNT10B
REPS2
SYAP1
ITGAM
TPST1
TM4SF4
TOR1AIP2
S100G
TM4SF1
OTUD3
OTUD7A
GUSB
FRMPD4
ZFPM2
ZNF764
ZNF768
TAS1R3
MAP3K2
PUSL1
SF3B5
TIGD4
IFFO2
MYO7B
CHD3
TMEM154
MYRIP
ALCAM
YWHAG
UBE2E3
CNTROB
TRAPPC1
ZPLD1
KCNAB3
GPR37L1
SIMC1
TMEM192

NSG2
NIPA1
HNRNPA3
SIK2
RNF150
VGLL2
HOXD4
HOXD12
USP38
SLC16A5
NANP
ADRA1B
ADPRM
FABP6
PWWP2A
USP50
USP47
PDCD6IP
ZNF212
FAM161A
ZNF282
GLB1
FAXDC2
CRTAP
SLN
CMTM8
GABARAP
STX8
CDK1
NFRKB
FABP4
PRDM10
B3GNT2
FOS
TMED10
EMX2
SP7
SEMA3E
LRRN2
SLC30A1
DCLK2
ZNF804A
GPRC5C
TMEM182
VSTM2A
KRT8
KRT78
ADORA2B
SDR9C7
MGMT
HARS1
NFXL1
KRT75
CD14
RIOX1
SPATA24

RALGAPB
PYM1
MZB1
KRT4
SLC23A1
NPAS2
NUDT9
PA2G4
ELOVL6
PFKFB3
ARL6IP1
SMAGP
IRX1
CDH2
IRX2
EMB
SIX2
DLGAP1
STAT2
NUDCD2
IRF2BP1
OR9K2
HSPA4
FOXA3
SLC26A5
COMMD5
SGCD
GTSF1
ARMC10
RNF34
TRABD
ATF7
SOCS6
CAVIN4
ZNF296
HOXB9
POLH
SYT9
KCNS3
KIF5B
GPR37
CDCA4
SDR16C5
DYDC1
CHCHD7
HTRA3
FOXN2
LMOD2
BFSP2
FSHR
USP32
CEL
PPM1D
KBTBD2
RIOX2
TRIAP1

LSM3
KIAA0232
MTSS1
TMEM43
RNF139
PLA2G1B
CYTL1
TRH
MSANTD4
PAQR8
NUDT6
TANC2
DNAJC24
MBD3L1
CAVIN3
HAS2
PDGFD
S1PR1
HS6ST2
PYGO1
LRRC8E
PKIA
XKR6
FEZ2
ALK
INSR
MFN1
NRTN
KCNMB3
KCNG3
ATP6V0E2
OR2K2
JAGN1
TADA3
SOCS5
C9orf16
MORN4
NAIF1
RBKS
TMEM126A
TMEM126B
TRIM8
NETO2
CLDN20
CDC42BPG
SCAND1
JUNB
FAM241B
TMEM37
LRG1
SHCBP1
SOSTDC1
NPTX1
FAM98B
GAA
CANT1

KCNK3
ZDHHC16
CHST11
EXOSC1
PGAM1
CHD7
ESCO2
KRT15
LURAP1
APLN
KRT13
PDE7B
GLOD5
KSR2
ZNF524
MCC
ZBTB26
CDK5R2
POLR1C
DLK2
ZNF318
HOPX
LRRC8C
SPACA5
RSL1D1
LRRC8D
MROH2B
PPID
COL24A1
ETFDH
RXFP1
LPAR3
PTGER4
TBCA
NEUROD2
MAP6
OTP
ECEL1
BCL2L1
FGG
OR2AT4
FGB
PLRG1
DNAI2
NMUR1
CLSTN1
CXXC5
PTCRA
SLC25A33
ENC1
SPSB1
P2RY6
S100Z
GPR82
GPR34
PLEKHG5

ATF7IP
LKAAEAR1
RGS19
TCEA2
ANO5
HDAC3
SPATA46
GPHN
VAT1L
TMEM51
CAMTA1
LRRC34
PAH
SPATA5L1
GATM
SYCE1
NXNL1
RASGRP4
NHLH1
SLFNL1
BCL2
RHNO1
CTPS1
KNDC1
METTL18
COL8A2
PWWP2B
PCDHB1
ANGPTL7
FBXL14
EXOSC10
ZNF570
MLLT3
RRM2
TRAPPC12
IFNB1
RPS21
MRM3
PTEN
RPS7
PRND
PRNP
KLF17
ADRA1D
FRMD5
TVP23B
FBXW10
ZNF217
SCG2
ATPAF2
CYP4F22
FOXB1
PPIH
DRC3
SHLD1
JMJD1C

SYNPO
MAL
RAB33B
THOP1
EPHX4
LAMB2
USP19
QARS1
ORMDL3
KLF11
LRRC15
EIF2AK3
KRCC1
NME6
CYCS
FRMD3
SNTB1
MTBP
TEFM
MRPL13
MALT1
PRL
ISG20
MBOAT1
CXCR6
ATOH1
PAIP1
CLEC7A
C5orf34
NEGR1
ZNF131
DPAGT1
BSG
HINFP
COPRS
TP53RK
B3GALT1
OR5A1
BPGM
POP7
ALG14
STARD5
CSDC2
RCAN2
ABCG4
GNB2
CFAP53
MCRIP2
PDZD3
C2CD2L
OR9I1
ARNT2
GNG12
PRSS27
MYOZ2
SYNPO2

CLP1
TTC36
RSPH9
GTPBP2
FGGY
IL17D
FUT9
ZNF24
MANEA
AGXT
ACOT12
FIBP
CARNS1
BANP
PPP1CA
HCFC1
CTSW
NIPAL4
PDE3A
RASGRP1
KLHL6
CHCHD1
MRPL52
SMPDL3A
RND1
RAD9A
EFEMP2
OR10AD1
TMEM134
ZMAT3
ZFAND4
MOS
FAM71D
CCL19
CORO1B
FUT10
LRRC20
MUS81
PURG
CFL1
TMCC1
EFCAB12
PSME3IP1
FADS6
HOXC5
RAB37
DCP2
RPL38
OVOL1
RARG
SSH3
PDP2
SP3
DMXL1
METAP1D
EGFL7

NADSYN1
DHCR7
LVRN
NBEA
RNASEH2C
ANKRD13D
MRGPRF
MYD88
OXSR1
PARP14
CKS1B
ABLIM3
MAB21L3
VANGL1
GLRX
IQCB1
SYT12
C11orf86
LIPM
GPR151
DMRT2
GPR137
SNCG
ZNF449
ZBTB21
STOX2
PSMD1
ADGRG2
SUSD5
WFIKKN2
TOMM20
C1orf100
AGFG1
CNP
JUP
TDRD12
EIF1
ENDOV
TIGD3
KCNH6
PLK3
NET1
DPY19L1
PHOSPHO1
PHC3
GPR160
CBX2
SPTBN2
GOLIM4
RBM4B
ATP5MK
HOXB2
C1QTNF1
MARCHF3
SWSAP1
SLCO4C1

RBM4
PIFO
XXYLT1
UBXN2A
TCAP
CCS
NRROS
CEP19
KLHL15
SLC16A13
GLIS1
CHRNA9
PODN
SLC6A19
EXO1
RALGAPA1
LIG4
TRHR
GTF2IRD2B
ABRA
ZWILCH
RPL4
SNAPC5
STARD6
VWC2L
LINGO2
DENND4A
IGDCC3
SLC26A9
MFSD4A
PELI3
P2RY14
GPR171
GPR149
CD164L2
FUT1
DHX36
ZIC4
FBXW8
ZG16
KLC2
TEX36
CTBP2
CHST2
ZDHHC14
ATR
UBE2C
GK5
VCPIP1
RTP1
DES
PDIK1L
RAG2
TRAF6
ZNF654
PACS1

MARCKSL1
SH3BP5L
CADM2
PSMD2
PPM1E
FAM131A
CSRP2
INHBC
DDIT3
OR52W1
DPP10
CLCF1
TSGA10IP
GPR152
UBQLN3
PNLIP
LIPT2
KCNE3
DRAP1
LONRF3
UCP3
UCP2
C11orf68
MRPL48
P2RY2
ERCC4
CCDC85B
TMEM70
RPS6KB2
RMI2
TOM1L2
TEX26
GPR156
MTLN
KDF1
HSF5
FOXG1
SPHK1
BNIP3
MYPOP
CIDEA
LRRTM4
ATAD5
SMIM19
RTTN
ACBD7
ANAPC2
HMGB4
ZBTB8OS
SLC35G1
ZSCAN2
B3GNT4
CDC26
HSD11B2
CRLF3
RNPEP

DMRTA1
GJC3
KCMF1
DNAJC30
SPRYD4
VPS37D
CLEC14A
SYNE3
LPCAT4
SLCO3A1
ZNF575
BASP1
VSIG10
IRX5
FAM91A1
WSB2
GRIN1
SOX11
TYMS
TCEANC
PNMA1
TCIM
MAMSTR
C8G
GCNT4
NFATC2IP
FIBIN
FAM89B
DPP7
SEC24C
MTHFR
DEAF1
MTX3
SIX5
FBXO46
ZDHHC13
SLC38A9
ACER2
WDR73
POLE
HIC1
PPFIA3
PAWR
TGIF1
NAP1L5
NIM1K
NR2C2
GPR4
OLIG3
CAVIN1
ARIH2
ZBTB33
IRX3
ST8SIA3
RPRM
SLC25A22

RABEP2
NHLH2
ATOX1
TBL1XR1
SAMD12
C18orf32
PIDD1
RPLP2
JUN
CSTF2T
PGBD5
ACAD9
IL17RA
PNPLA2
MBOAT4
AGTRAP
SRRM3
THAP5
CRACR2B
CD151
POLR2L
ODF3B
C2orf73
GPR150
DMAP1
ADAMTSL1
CALHM5
IMPDH2
ALS2CL
MLF1
NDUFAF3
C2orf69
GRAMD1C
TSSK6
BOLA1
DDX10
PPP1R42
NDUFV2
DALRD3
ZNF518B
SPINK6
ZNF366
LCORL
PARD6G
SH2B1
SUZ12
NSUN3
RPP25
GRINA
THBD
GP5
COX5A
STX19
FAM219B
ZHX2
CPN2

C5orf46
MPI
H1-8
OPLAH
TMEM52
TMEM139
RNF186
MSC
APOLD1
RFLNA
EXOSC4
DPY19L3
TAF7
FOXE1
PFAS
HYI
CYBC1
GAK
ZBTB7A
TUFM
RMI1
FBXO34
SELENOW
SNX18
EXD1
AURKB
TAS1R2
C14orf39
MRFAP1
C3orf38
KLHDC7A
TMEM107
RRS1
EXOC3L1
CCDC89
DPM3
CYC1
PER1
HTR1F
TMTC2
FARSA
SPTY2D1
SAMD4B
EDC3
TCAIM
FUCA1
GGN
TMEM125
ZNF664
CALR
MAGED1
GVQW3
LDLRAD3
SMCO3
RAD23A
GADD45GIP1

TMEM151A
PTPN11
NSUN7
WSCD1
GATA2
ARID3B
TMEM31
ELMOD2
EGR3
GPC5
VWA1
DNAJB8
GEMIN4
FJX1
KLHL28
MKRN3
ZBTB18
C14orf28
SHARPIN
LBX2
SLITRK4
HTR1D
GCC1
LSMEM2
ZFPM1
PLD6
GPHB5
CDC42EP4
LACC1
MAF1
TPPP2
FCER1A
ARL14
PCED1B
PIPOX
FOXS1
ATOH7
CDH5
LRRC3B
FAM216B
MYADM
MROH1
SERTAD2
AKAP5
NKPD1
RNF227
C1orf194
R3HDM2
B3GNT3
SEPHS2
GPBAR1
C14orf119
CCR8
BBS10
FIZ1
DCTPP1

ZNF771
TSHZ1
PSTK
SOCS4
ZADH2
ZNF48
TMEM150B
TMEM86B
SEPTIN1
TRNAU1AP
EXOC3
TDRD6
LYNX1
TH
MED14
FAHD1
TDRP
RCC1
PRKRA
ZNRF2
RRH
SLC9A4
FGD6
GPR139
PLD5
OAZ2
PNPLA1
ALX1
CCDC43
KCTD4
MEIOC
TIGD2
HCLS1
MTURN
ZNF609
PAK2
DEFB124
MCFD2
HARBI1
DEFB123
CYP8B1
SERTM1
ARHGAP45
GLIPR1L2
MIGA1
NRIP1
BHLHA15
RNF182
TSPYL5
FUT7
SKIDA1
MB21D2
GSX2
SSTR2
PCGF5
PRF1

MAB21L1
YOD1
TMEM64
CHRM4
SHISA2
S1PR5
CLRN3
GPR157
AGTR2
SLC36A4
ZDHHC20
OR51E1
HOXC9
PPA1
HOXC10
PSMG4
MAP6D1
CXCR2
C11orf42
SSR4
CUEDC1
KCTD2
D2HGDH
OXTR
CMTR2
FAM83H
GPR62
TCEAL8
LRRC57
MRPL14
GPR137C
C1orf105
BBS12
ZFP82
LSMEM1
NQO1
AEN
FKRP
TRAPPC5
SLC25A42
METTL23
SLC26A11
HIGD1A
CHRM2
EHMT1
ADIPOQ
F2R
NPM1
PJA1
DHTKD1
PENK
ZNF746
POLR2A
SLC25A41
TLCD5
TMEM132E

ZNF322
NME9
HEPHL1
LRRC75A
SYNE4
OGFOD3
WASHC1
UTS2R
AATK
DDN
ZNF467
SOX2
RAP2B
ZBTB2
RNF135
SGSH
MAB21L2
FANCB
SETD2
TMIE
MRPS23
GPR135
SLX1B
P2RY13
TNFSF15
ATG9B
GPR88
PLAG1
YIPF6
ZBTB20
DIPK2A
MACIR
AMIGO1
GPR3
TMEM252
ODF3L2
ADGRB1
LSM10
RELL1
RFX7
SLC35C1
TIGIT
RNF41
FTMT
IBA57
ZNF329
C5orf24
ADO
COA4
PRKAG1
GINS3
NEUROG1
CCDC149
MRPS11
SNRPE
RTKN2

PNMA8A
CHST15
USH1G
IDH2
TMEM259
GET1
TNRC18
FAM181B
TMEM30B
DEXI
NOP10
FAM89A
KCNIP1
TDRKH
IST1
ERCC6L2
MRPL41
ENPP7
CREB3L2
TSEN54
RGMA
ASB18
UBA7
MRPS16
SHISAL2A
RAD51B
LDOC1
ARL6IP4
EXT1
SHMT2
MOB2
HHIPL1
ATP6AP2
CYB5D1
BACE2
KCNA4
FIGN
IZUMO1
TMIGD1
C8orf33
KCNJ14
C1S
GLTPD2
ZNF804B
KBTBD3
YBEY
NXPH4
CACNB4
TRAPPC6B
NPLOC4
OTOL1
KCNK4
TSHZ2
CAPN12
EXOC7
KPNA2

XKRX
BGN
CEP97
LHFPL1
FES
GLRX5
TBPL2
CAV3
LIMK2
MFSD5
RNASE10
ADI1
RWDD4
SPNS3
SATB1
NXPH3
CSF1R
EPHB3
EPGN
KRTAP11-1
TSPAN10
PLCB1
SKA2
NDN
CCDC172
KCNB2
BRICD5
IGIP
TSKU
CMC4
ANXA2
RGS6
HOXB4
SLC35D3
PAQR7
PAPPA
NGRN
RPS17
HCAR2
CCDC87
C1orf116
DDX28
ACBD3
C16orf72
PLCXD3
ALG12
LCK
RBM10
ADGRG3
RPL35A
RGS7
C11orf54
CEP63
SRPRA
OTOP3
EWSR1

ODF3L1
GJC1
CNOT10
MTA1
CADM1
PYCR1
NAA38
SPNS2
SLC25A21
OTOP2
PCP4
ABAT
SLC25A10
LYSMD4
WBP2NL
IGSF5
NKX2-5
AFMID
GAS6
ARHGEF37
CALHM3
PTGDR2
CEP57L1
RIPPLY3
GPR19
GJD3
RABIF
TMEM119
FANCF
C2CD4C
CHST6
RUVBL2
GDPGP1
PTTG1IP
DDX41
ABHD16B
CCDC60
DAZAP2
CCBE1
SELENOF
EPHA10
BOLA2
BCOR
JRKL
CABCOCO1
KIAA2026
OVCH2
SYNDIG1L
FHL3
PMCH
TMEM89
C19orf71
CCDC159
RIPK4
LRIT3
SF3A3

GRIN2A
URAD
ASB7
SH2D7
TREX2
MEX3B
TENT5C
COA5
UTP11
PSMG1
SETD3
TNFAIP8L3
FBXL7
TANGO2
GKN2
FAM167B
MRPL54
HMCES
CCR3
KRTAP8-1
HOATZ
NDUFB1
MARCHF11
KLHL25
TRMT12
ALYREF
NOG
UPP1
LHFPL6
CMTM4
TMEM50A
FIGLA
TBK1
CBX6
MACC1
TBL3
ACP7
KREMEN1
TRAIP
CHEK2
FOXL2
AIFM3
KCTD16
B3GALT5
ZNF703
SLC35F3
KCTD8
EMILIN3
OLFML1
RBM12B
CCR4
BTBD9
NUDT14
GPR39
FAM3B
KIRREL1

CNGA2
TOB2
ARSI
TTC32
LRRC55
DNAH2
SH2D1A
SDR42E2
KMT5A
SMTN
COA3
ST6GALNAC3
PTP4A2
DENND5A
VPS33B
ADAP2
UQCR10
TRIML1
CLDN5
NIPSNAP1
CNTN2
SPRR4
LRTOMT
KCNQ3
NR2C2AP
C1QTNF12
CRELD2
SCFD2
UBE2F
GPR173
PGP
SNRNP35
DGAT2L6
IRAK1
CMSS1
PCDH9
OAF
KCNK12
POU6F1
TM2D3
TSSC4
TACSTD2
SIX6
CCSER1
ZDHHC23
SRPK3
GDF3
IQCF2
EFNA5
PKP3
CSF1
COLEC10
ACTRT3
PLA2G6
MAML2
A3GALT2

SS18L1
COPB2
LRRC19
THAP7
KNTC1
CCR10
NCMAP
BPIFC
TXNRD2
FOXO4
POU3F2
PROS1
GAST
NUTM1
HDDC3
CEND1
C6orf58
DHRS7C
SOCS3
SLITRK6
LPAR5
XPOT
PDE4B
TAFA3
SNN
NELL2
MED12
SEPTIN9
AMER1
ZBTB40
CLDN6
EIF4ENIF1
SERINC4
RNLS
NDUFA12
UBE2G2
OSBP2
TRARG1
ZBTB7C
APOO
PRR16
DRD1
TMEM186
SDR42E1
RBM33
ARMCX2
BTBD6
RBM43
IMMP2L
JAG2
FMNL1
PTRHD1
OR6A2
ZFP90
USP18
CHRM5

TMEM106A
SIVA1
RFX6
ROBO2
AP3M1
CA13
UBOX5
BRF1
LRRC14B
SEMA4B
MROH2A
CIB1
NELFA
SLC24A3
SGCZ
EFCAB10
FLRT2
INTS5
RPS27L
MANEAL
FAF1
MYADML2
FAM43A
FAM131C
PDE6G
PRKG1
DLK1
OLFML2A
SP1
MRPL40
DBX2
INKA1
PCGF3
LMLN
P4HB
PSMD13
NDUFA4L2
SHC4
ZFP36L1
NTF3
PMEL
SYN3
SNAI3
ZBTB3
MORN5
C6orf201
MYBL1
MOSMO
DRG1
ANKFY1
YTHDF3
ADARB2
NRG3
SRL
C11orf87
CXorf38

KCNQ5
ADAMTSL5
MORF4L1
WDR53
DMWD
PIGP
IKZF1
PCYT2
NAT8L
BCAP31
GNB1L
EVI2B
TMEM210
THNSL1
ATP6V0C
PRSS38
LAMP1
POMK
C16orf54
KLHDC8B
KLHL34
SETD4
PTCH1
RTN4RL1
CALHM1
RNPC3
IRS2
BICD2
CCIN
SLITRK2
LRCH3
NDUFA13
ZNF566
DLEU7
TAL2
AIDA
CDIN1
ZPBP2
KRT5
CYP2R1
LRRC70
ANKRD46
PIP5K1C
TEX38
ZBTB6
C2orf76
POLR3C
PRR30
UBL4B
WWOX
BCL9L
POLR1D
ZNRF1
FFAR4
BPIFB3
BPIFB4

SAPCD2
EDARADD
GLDN
KPNA4
TMPRSS12
RPS23
KLK12
RGS7BP
MYT1L
TMEM222
ARHGAP30
SMYD4
FOXD2
GPATCH8
NF2
SMIM29
UBE2H
HPDL
KATNA1
FSD2
ARAP1
KIF24
PDE2A
ZFP91
C17orf58
BCDIN3D
LYRM7
CFAP73
BCR
FSCN2
SPIN2B
HYAL3
KCNK18
CXCR3
ZNF397
TPCN1
TNFRSF4
HEXIM1
KRT14
POFUT2
QRFPR
ERCC6L
OR13F1
TMEM17
FGF3
C1QL4
RTN4RL2
ZDHHC17
SERPINA11
P2RY4
ZNF395
FAM183A
KANK3
EMID1
ACTL7A
ESPN

PTRH1
SAMD7
GPR141
TMPRSS6
CYP4A11
TMEM216
RPS19BP1
TMPRSS11A
TMEM262
C3orf70
PLCD1
CCK
ENTPD5
MITF
NAP1L1
CMC1
SLIT1
LYPD6
VSTM2B
RNF220
ANGPTL5
SHTN1
TSPYL4
GCNT1
SESTD1
FNBP1
DYNC2H1
BCAM
NPSR1
WDR86
EPOR
CIDEC
LUZP2
CHP1
KCNJ11
CDHR4
COL4A1
GJA4
HSPA14
ATP13A5
SIRT7
IFT140
AGMO
SBK2
CYP26C1
TLR5
USP7
NHLRC1
PLEKHN1
ZNF385C
TET3
ISG15
MYMK
SAMD11
VMAC
C5orf52

HAPLN4
B3GLCT
SPRY4
ERAS
TRPV2
AMTN
TMEM203
SLC18A3
KBTBD12
THSD4
DNAJB13
GABRD
TCEA1
NHEJ1
SEMA4D
LIN28B
DIPK1C
MCRS1
TMEM72
CARD9
PEAR1
ZFP69
HELT
TMEM220
EIF4EBP1
P2RX2
CCDC157
PABIR1
SHISA7
LRRC74B
LDLRAD2
C2orf66
CYHR1
COL14A1
DNER
KLHL17
PLA2G2C
TPRG1
S100A3
UBQLN2
RILPL1
CLCN1
NRN1L
ARL4C
RNF133
TMEM221
RAB42
C11orf95
PRSS45P
PLA2G4E
GPR89B
MESP2
C6orf132
TMEM215
AGRN
OTOG

FAM166A
TMPPE
HEPACAM2
SMTNL2
ZC3H6
LAMTOR4
DCUN1D3
COMMD6
HYKK
C15orf62
ZNF383
LRRIQ4
PLSCR1
C3orf62
ENO4
SLC38A3
GTF2F2
CIBAR1
FOCAD
PRR19
ZP3
H3-5
PPP3R2
GPR21
DYNLT4
SELL
NANOS2
BLOC1S5
CERKL
SLC24A5
INSC
SERPINA5
C19orf54
LCTL
NCCRP1
COL25A1
FAM83G
CFAP77
SRSF10
CCDC9B
NBR1
RALGAPA2
NDOR1
FBLL1
PAQR9
CLEC20A
ASAH2
SUMO2
HMX3
RTL6
S100A16
PTAR1
CC2D2B
SAXO2
C2orf80
IDO2

SLC4A5
UROS
ZDHHC9
QRFP
SMIM15
OSTN
VWC2
FAM221A
RBM34
TMEM198
BCL2L15
FZD9
SPRED3
ADRB3
SKOR1
PRELP
PLA2G2E
MTF1
TMCO2
SHISA6
TMEM201
NHLRC3
HMX2
SNTN
CALHM6
CNR2
ENTPD8
RPL14
BEND4
MSL1
LRRK2
BSX
GJB3
INSYN2A
HACD4
CFAP126
NYX
C9orf152
NOC2L
AADACL3
SLC15A5
LIPI
ZNF292
KCTD21
SBSN
ADAT2
ZNF567
NDUFA4
ANKDD1B
ALKBH2
RNFT1
RNF222
RELN
APOD
H1-0
LITAF

TMEM120A
ARID2
SF3B3
PRSS48
IL1RAPL2
SP6
ANKRD34B
PLAC9
CLDN4
GRAPL
JPT1
S100A13
PCDH18
MAOA
C15orf61
NUGGC
PNRC2
GJB5
FHIT
ALKAL2
RRP7A
LIN54
FAM53B
FAM180A
S100A14
HMGB1
MMP23B
SH2D5
RASSF10
GJB4
BLOC1S2
IL1RAP
MYBPC1
ZNF699
TDRD7
KIAA0895L
SERPINA3
SPATS2L
WDSUB1
S100A4
PLEKHG4
FAT4
KIF19
ACADSB
STK40
OR10J1
TMEM63A
SEMA4A
MPHOSPH8
FAM217B
LCOR
SUPT5H
XPNPEP3
PPIA
SUPT3H
NIF3L1

IARS1
ZBTB44
STK31
NLGN3
NTNG2
LONP1
TRRAP
ASB13
SLC35F1
INCA1
PTPN1
EVL
THEM5
EPHB4
XRCC6
S100A5
C20orf204
TSC22D2
ZNF569
YRDC
ZNF777
PIK3R4
TRAPPC2
RFX8
MYL6B
SIAH1
NCOR2
ARL9
PRPF40A
GDAP2
ANAPC7
TPK1
SLC6A9
AFAP1
MYO18A
BORCS6
MAN2A2
MME
FAM72A
CCDC196
CACNA1H
SULF2
LAMA2
PFN3
PLXNB2
AJAP1
XRCC2
MYO6
HDAC2
MMP1
TCF4
TRPV1
ZNF512B
AMZ2
NF1
VKORC1L1

DAPK1
GM2A
POU3F4
CD47
MAML3
STRN3
CHRNG
MVB12B
ILRUN
ADA
ARID5A
PATE2
PPTC7
TOMM20L
NHLRC2
LAMB3
TEX43
ARHGEF12
HCAR1
PDLIM7
TMEM26
FAM3C
SLC39A10
AP2A1
FUT11
ANXA4
LAGE3
WDR5B
FAM163B
WDR45
METTL9
ZNF470
SERTAD1
ZNF398
ZSCAN25
ANXA6
GMFB
SIGLEC15
ZNF420
DTHD1
ZSCAN26
MAFG
ARRDC1
GAL3ST4
DYNC1H1
SLC6A17
PCBP2
SLC25A29
PGAP1
PCNX3
ACSL5
LRRC8B
ABCB8
SND1
PSMD12
ADGRA1

PIWIL2
NOL4L
CYSRT1
ENTPD4
C1D
TBC1D9B
FAM110D
GTF2E2
IL27
GUCA2A
FITM2
BLM
TRIM33
LRP10
PELI1
ZNF655
MRPL21
UAP1L1
FBXL22
SLC22A5
DACT3
ADARB1
HTT
C5AR1
DIO3
VEPH1
OR51D1
IPP
OPALIN
MAP3K5
CYP2F1
GSTK1
HNRNPAB
PDGFA
COL13A1
SPN
SLC2A10
RPF2
SLC28A3
MIB2
MYO5A
ATG7
SIPA1L1
RAB40C
PIGN
HOXA4
TOPORS
BCO2
ENTPD6
DMBX1
CCDC154
FAR1
EOLA1
CDC42SE1
SERPINB2
DPP4

PDCD1LG2
CCER1
DNAH10
OR51C1P
SPTAN1
NMB
PARVA
KLHL14
FAM114A1
RPE
PHF2
RPS26
S100A10
CFAP43
LHFPL5
RPL37A
HOXC6
MCMBP
EME2
KLHDC1
TAF13
ATAD3A
OR52M1
FAM118B
SLC9A8
CYP2A13
INKA2
GPAA1
ADAMTSL2
SGTB
CYRIA
MYO1C
MEIG1
SLC22A12
KIF13B
NRAP
ADH5
SLC22A6
ERO1A
PLCG2
FCHSD1
RPL12
MPZL1
VPS13A
MBP
ELOVL2
LEKR1
C1orf122
CLEC9A
IRAK4
ODAD3
MRPL42
ENTPD7
ZNF335
RPS4X
MAK16

ZNF667
AVPR1B
SIRPA
GRK6
PRIM1
MARCHF5
ZBTB14
CD2AP
TMPRSS11F
ZNF248
CHSY3
LPAR1
MB
HIBCH
TMEM229B
SOWAHC
ZNF770
MIER1
MAN1A2
SVIP
DDRGK1
TFDP1
BPIFA1
RPS6KL1
CACNA1E
QRICH1
FKBP1C
DDX42
RPL23A
SLC29A3
STYX
UBL5
HELZ
UCKL1
CARD11
SDAD1
ZKSCAN8
TMEM239
HYLS1
HOXC4
GET3
ASPH
SPRED2
WWP2
UVRAG
ITSN2
NTRK1
OGA
TCAF1
ZNF583
CCT8L2
ZXDB
TPM2
SH3BGRL2
ANKRD35
YTHDF2

ATL1
CNGA1
MAFK
ARMH1
GPN1
PLN
ITGBL1
ZNF511
KCNRG
WDHD1
CTNND1
SLC34A3
RD3
SH2D1B
ARC
TLK1
ZNF536
MMP17
BAZ1A
COPS8
MDM4
KLHL9
FAM3D
NCOA6
TAT
C6orf89
CALM1
TAFA2
TTC37
SLC9A6
ABCA4
IPO9
CEP290
MT-CO2
GLMP
TOGARAM1
ANKRD13B
ECI2
TEX45
MT-CYB
LDB1
PPP1R14C
CTR9
SMOC1
MSRB1
LRRTM3
ZNF652
SLC5A3
GPATCH3
CDC42BPB
PLXNB3
RPL10A
EPS8L3
SYCP1
RUNDC1
CCDC152

SFMBT2
ASB12
MT-ND4
SMC5
PRMT6
CIPC
MT-ATP6
TOP1
PRC1
MAP3K3
L1CAM
SREBF2
C1orf174
RASGEF1A
SPOUT1
RPL39
ATG9A
NOS1AP
APRT
TBKBP1
CCDC167
MT-CO3
ZFP2
SOWAHA
L3MBTL3
MFAP3L
NAGA
SMG5
KIFBP
TGM2
ARMCX6
PJA2
RORB
SGMS1
OR10Z1
INF2
EFCAB2
COX20
CHML
STUM
TATDN3
C1orf53
SAMD5
TEDDM1
GJE1
ECT2L
GPR52
METTL11B
TMEM244
CENPW
FAM229B
FANK1
PRR9
LELP1
EEF1AKMT2
DDO

PLPP4
RBM20
C6orf163
RIPPLY2
C1orf146
C10orf62
SAMD13
CCDC160
EFCAB7
GLYATL3
LDLRAD1
ZYG11A
C1orf185
GLT6D1
LIPN
LIPK
LRIT2
LRRC73
SYS1
INPP5B
MAFB
GIGYF2
RUFY2
NHSL2
PHACTR4
ZDHHC18
CXorf65
GPRIN2
MACO1
PTPN20
GDF5-AS1
BMPR2
OXLD1
OR13C3
COL5A2
NBDY
PJVK
MRPL38
SMIM5
SP5
BTBD17
SDHD
LAYN
MBD5
CSNK2B
FAM155A
FOXB2
DISP3
RACK1
TBC1D8
NMS
ASPDH
AKT1S1
C9orf135
INSYN2B
ODAD4

MRPL53
DCTN1
TCTN1
FAM216A
ZBTB48
MZT1
LRRC3C
UQCC3
FBXO48
GRXCR2
LRRC10B
FBXO47
C12orf73
TRIM13
ERICH4
MS4A13
SPIRE2
SLC35B4
TMEM231
FAM71F2
TMEM240
CDKL4
TMEM88B
C4orf47
TRIQK
SDHAF1
ARID3C
PSENEN
C11orf91
ZBTB10
C4orf46
LGR4
PSMB10
VIT
E2F4
PDE7A
TMEM170B
CTXN3
SNX2
SARNP
ADGRG1
IPO7
PRR13
TECPR1
INSYN1
CNEP1R1
IZUMO3
ATP6AP1L
RGL3
TMSB4X
C9orf92
CPT1B
HMGN1
DENND6B
EIF3CL
LCMT1

MFSD2B
CDPF1
LIN52
TECRL
MANSC4
ETFRF1
C17orf107
ITSN1
ITPRIPL2
DENND1C
CRYZL1
C5orf51
ARRDC5
PLPP6
GMNC
LRRC72
BHLHA9
OLIG2
RNPS1
NYNRIN
DNAJC19
IFITM5
C18orf63
JPT2
TMEM211
SERPINB5
CFAP99
RNASE13
ANKUB1
TSSK2
COL6A6
LRRC30
TMEM200C
HACD2
CFAP44
LNP1
VGLL3
TRIM71
ANKRD28
COLQ
METTL6
XKR4
GPX3
DIO2
SELENOH
DIO1
STK38L
TSN
SLC48A1
TSSK1B
RNF208
KRTAP6-3
SYT3
NUP62
DENND1B
SFT2D2

CEP43
SCAF8
CFAP45
ACKR1
CRYGS
KLHL23
LINGO4
TRIM59
MLLT11
ASIC3
CCDC183
DNLZ
SUPT4H1
TSGA13
NRAS
ANKRD39
QTRT1
CHUK
MXD3
COG8
LCAT
PTPRCAP
GPC2
SIPA1
SYNJ2BP
ARL2
SRA1
STIMATE
DNAJC9
CPLX3
VDAC1
TMX2
HEXA
LEPROT
LBH
ADAT3
PPP1CB
LAT
NCKIPSD
TREX1
S1PR3
SLC35F6
RPS29
DDX47
EMP2
PPM1N
CEACAM16
SLC23A3
DNASE1
GALT
CLDN9
ITGA1
NUDT19
TAX1BP3
AP1G2
TM6SF2

GANC
REPIN1
SMIM7
FBXO16
TSPAN4
CPNE1
ARL16
SMCO1
LYRM4
MYCBP
TMEM213
ALG3
SMIM30
C19orf38
C17orf67
MINDY4B
FIS1
NPS
COLCA2
MBLAC1
FOXI3
NEURL1B
VSTM5
FAM187A
PLIN5
HIGD1C
NOTO
PPME1
STARD10
B3GALT9
ZSWIM8
IQCF5
IQCF6
C10orf105
ARHGEF33
IFRD2
TOMM6
FER1L6
MTCP1
ZSWIM7
GPR33
LRRC69
GSG1L2
ACSM4
COL28A1
PHB2
GRID2IP
UBXN2B
MSMP
FAM166B
PEX26
GRXCR1
C5orf49
UBE2QL1
FASTKD5
KCNU1

HOMEZ
DDX3X
VPS16
NPEPL1
KRTAP10-4
RPL17-C18orf32
SKOR2
SIAH3
C20orf202
RSC1A1
TMEM242
TMEM167B
TNFRSF25
TSTD1
CYB5RL
LACTBL1
IFI30
FNIP1
SYCE3
CKLF
CEBPZOS
CELA3B
RNASEK
TAFA5
UMAD1
ZGLP1
VAMP2
C6orf226
PPP3R1
FANCG
AP4M1
PPP2R2A
TRIM16
FXYD7
XKR9
SLC12A8
FADS3
OR4E2
CCNL2
MYBPHL
OR52B4
BTBD19
FAM185A
PSMB11
EXOSC6
C1QTNF5
CPTP
PRR29
ATXN1L
SMIM13
PRRT4
TMEM233
MKRN2OS
C2CD4D
SLC26A6
NOL7

NTF4
PIGBOS1
OR52I2
TMEM185B
CUTA
HSBP1L1
C13orf42
C14orf132
PARG
SCAMP4
RD3L
MRLN
FAM174C
OST4
CLDN25
DHFR
PATL2
NKX1-2
SLC39A7
ZNF688
PET100
ANKRD66
ACBD6
CLIC1
ANKRD63
STMND1
FKBPL
HSBP1
PLSCR5
CFAP97D1
SBK3
AKAIN1
TMEM253
TMA7
TEX50
DYTN
TMEM114
FLOT1
HSD17B8
KANTR
TNF
PET117
DDAH2
RNF224
BTBD18
DHX16
PIRT
MRPS18B
RPS28
CTXN2
UQCRHL
TMEM229A
ZNF879
CCDC188
KBTBD13
ATF6B

FAM133B
MCIDAS
PSORS1C2
JRK
BAG6
C11orf94
NEU1
HNRNPUL2-BSCL2
ATP6V1G2
SHISA8
C19orf81
TUBB
FAM237A
C12orf75
SMIM1
HGH1
PPP1R3E
RAMACL
SPAAR
SMIM27
RNF148
RPS18
ATAT1
APOM
PATE3
LY6G6C
CLEC2L
ZBED5
SLFN14
ABCF1
PSMB8
ZBTB9
ZNF853
PPT2
ARHGEF38
ZNF593OS
B3GALT4
LSM2
NRM
AGPAT1
POLR1H
C3orf84
TRIM27
C4orf51
B3GNT9
CDKN2AIPNL
RNF223
PFDN6
PBX2
AGER
PRSS56
SHISA9
KIFC1
C2orf74
RGL2
MPC1L

TMEM250
TXNDC5
CASTOR1
RNF103
RBM14
ALKBH6
ASB14
KLHL41
STPG4
MEIKIN
NME1
TNFSF12
TLR9
WBP1
GET4
C1orf226
ADSL
STRIT1
PCDHGC3
PDXP
RPP21
ATP5MF
ARPC4
UGT1A1
ARPC1A
ATP5PO
PLEKHO2
C8orf58
HOGA1
PI4KA
HYPK
AMACR
MTFP1
IL10RB
WDR92
NME2
CFAP57
TTC4
TMEM35B
JMJD7
MRPS6
ZNF512
RTL9
ACY1
NFS1
DDOST
TMEM199
DNAJC25-GNG10
TMEM141
IFITM10
PCP4L1
ABHD14A
STIMATE-MUSTN1
RBM14-RBM4
LY75-CD302
CCDC153

ACTN3
SMIM31
TNFSF12-TNFSF13
FMN1
HAUS5
TMEM150C
CLRN2
FER1L5
HS3ST5
PDCD6
ARPIN-AP3S2
YJEFN3
ARPC4-TTLL3
GIMD1
SMIM20
TUNAR
CDK3
GPR162
ATP6V1E2
SELENOP
PRODH2
EXOC1L
PTX4
RGS21
C8orf88
SHLD3
HOXA10
TMEM200B
C1orf210
SMIM18
SLC10A5
ALG11
ATXN7L3B
ZNF260
LYN
PINX1
LRRC24
HSPB2-C11orf52
AP5B1
OMP
INS
RTL1
FPGT
MEX3A
EEF1G
CKLF-CMTM1
SYS1-DBNDD2
BORCS8
FXYD6-FXYD2
SMIM35
EID1
POLR2M
FDXACB1
TRIL
TIFAB
DNAAF4

SMIM3
HMBS
DND1
POLG2
CAPNS2
GPR142
HP
C11orf97
TAS2R38
GATC
ZBED6
FNTB
RPL36A-HNRNPH2
GALNT4
CNPY2
CUX1
TEX49
CHURC1
C17orf49
RTEL1
PDF
RNASE12
SPESP1
SPECC1L-ADORA2A
BCL2L2-PABPN1
SYNJ2BP-COX16
C20orf141
RNASE4
MC1R
HOXB7
FRRS1L
PMF1-BGLAP
ARMH2
MRC1
HSPB9
TLE7
SERTM2
XKR7
CCPG1
TMEM178B
CLEC19A
FIGNL2
PECAM1
TEN1-CDK3
GAN
C15orf65
TCF24
SMIM36
CLK2
GFY
GATAD2B
KRTAP3-1
DSEL
ATP23
C17orf114
MYMX

DENND11
IQSEC3
SKA1
TDO2
SPON1
OVCA2
FAM189B
PKLR
MRPL12
INTS3
LSM14A
C19orf84
DNAJC28
CCNQ
GTF2I
ATP6V1G3
PAXBP1
MYZAP
TMEM50B
DPEP2NB
CTSO
KRT12
SCAMP3
KRT23
OTUD7B
GJA5
RBP3
RBM8A
TIMM23
RNF115
CFAP298-TCP10L
RPL17
SEC22B
TXNIP
SRGAP2
BAHCC1
RASSF5
STRADA
KLF14
NCOA4
MRPS21
GDF10
AARSD1
C17orf113
ATF7-NPFF
S1PR2
CALR3
TRABD2B
MAGIX
IKBKG
SPIB
CCDC194
SLC25A53
EGLN2
ARHGAP19-SLIT1
COMMD3-BMI1

FMC1-LUC7L2
MEI4
NCBP2AS2
BORCS7-ASMT
URGCP-MRPS24
GAS2L2
RPS10-NUDT3
C17orf50
RASL10B
SRXN1
CCL5
LIX1L
SMIM32
ANKRD34A
GTF2H5
KMT2B
CFAP206
MUSTN1
DCP1A
ATP6V1FNB
ARL2-SNX15
GRIN2B
LYPD4
SNURF
ZBTB8B
C1QTNF3-AMACR
TM4SF19-DYNLT2B
PANK4
FAM181A
AGBL1
NAP1L4
EEF1D
CHCHD10
B2M
DDX24
USP27X
NOL12
PTPRK
INO80B-WBP1
TMPRSS11E
CASTOR2
PUF60
PRIMA1
SMDT1
NATD1
SOCS7
ADRA2B
SCRIB
DECR2
VASN
CDIP1
PROP1
TMEM269
DLG5
DERL3
PRPF8

C8orf89
YWHAE
LY6H
RCC1L
SEBOX
LHX1
PVALB
NMRAL1
KRTAP7-1
NPHP3-ACAD11
CLPTM1L
ITPK1
CCDC92
PIWIL1
PPP4R3B
SYNRG
NUDT18
ZNF2
GPR20
YTHDC1
FAM120B
PRAG1
MMP11
DYNLT2
MPV17L
BDH1
DLL1
ALOX5
ARHGAP11A
TTC34
GRIFIN
BFAR
GON7
NAPEPLD
KLF13
NTAN1
SMARCB1
MLLT6
U2AF1L5
NDE1
TMEM251
NCF4
DUSP14
UHRF1
ORAI1
AATF
SDCCAG8
SLC22A18
ERMARD
HNF1B
RNH1
PIK3R6
SCARF1
ZNF623
TMEM179
SINHCAF

UNC79
PLCH2
TAF9
APBA2
PLPPR1
BAAT
RAB7B
DACH1
PYCR3
MIF
CEP170
DNAJA3
CWC25
TC2N
WNT9B
TAF15
PLA2G10
MYT1
IFI27L2
C17orf98
CEP20
TRPV6
PDCD1
HEATR9
SALL3
RXFP3
MTMR10
ARHGEF26
MARF1
PIGW
BTBD7
CDK7
OTUB2
FAM243B
HERC2
PIP4K2B
SRCIN1
TJP1
ZNF280B
HMOX2
ELANE
NEFL
SCG5
GSTT1
MROH6
PSMB3
CCDC125
SRD5A2
SPC25
NEU4
CISD3
TIGD5
TFPT
DUSP8
SSTR3
GFUS

RDH13
GGNBP2
PPP4R4
NDUFA3
MYO19
G6PC2
CDC42EP5
NAPRT
RRN3
TIMM22
C17orf78
LENG1
DHRS11
SLC43A2
NR2E3
ZNHIT3
C11orf98
MRM1
PCGF2
IQCA1L
ASB2
GSDMD
CCDC166
UBR7
BMERB1
SERF1B
MMP28
OR2S2
SMIM34B
TSTD3
AK6
GARRE1
TAMM41
GATD3B
OR8B4
PCDH20
RASA3
CACFD1
TRAPPC4
TMEM42
FBL
PADI3
VPS11
SURF1
ZNF660
HHAT
HYOU1
CEP72
BORCS5
REXO4
PAGR1
TUBGCP5
RPS25
RPL7A
OR5P2
PADI6

SURF4
DUSP16
CENATAC
MATR3
NDUFA6
MED22
SURF2
ATG16L1
SLC2A6
MLXIP
PSMC4
SERTAD4
FOXR1
ZNF35
SURF6
DYRK1B
BLACAT1
BCL2L14
PADI1
FOXO6
RCC2
INPP5D
DUSP29
KIAA1143
KCNIP4
RYBP
KAT6B
SAG
RPS6KA1
SLC16A1
TMEM265
RPH3AL
GREM1
NSMCE3
DLGAP2
ADORA3
CORO7
SLC25A26
CTIF
CALB2
ECH1
ASAP3
C1orf232
BCL7A
B3GALNT2
RINL
E2F2
NFKBIB
MAP4K1
PRSS22
TCEA3
HNRNPL
FBXO17
MAGI1
HNRNPR
EIF3K

LGALS4
ZNF436
MRPS12
ATAD1
ZNF445
ID3
IBTK
LBHD2
TPBG
CCER2
SIRT2
SARS2
TEX52
CTXND2
EEF1B2
CCDC195
NDUFS1
GPR1
INO80D
PRRT1B
C19orf85
FAM169B
FRG1
EXOC3L2
TCF20
VSIG10L2
PRSS50
SPEM2
SMCR8
TMEM256
CANX
MAML1
TNK1
GPR22
HBP1
ZBTB4
VPS53
LTC4S
TPTEP2-CSNK1E
FGF11
IGSF6
CHRNB1
GNG14
TMEM159
PLSCR3
PLCL2
KPNA7
PRKAR2B
SQSTM1
DUS4L
SMURF1
LLGL1
EEF2K
SCO2
DCHS2
TMEM102

TOP3A
RUFY1
POLR3E
ANKS4B
PDZD9
SHMT1
PERCC1
TMEM256-PLSCR3
THSD8
MIEF2
MGAT4B
LYNX1-SLURP2
FLII
SPEM1
TMEM247
OR8S1
PDE11A
GMPR2
EEF1AKMT4
CARMIL3
HSF1
SMIM41
DCAF11
DHRS4
ADCY4
EPHA1
CCDC39
OR2F1
DHRS1
PSME2
KBTBD4
RUVBL1
TINF2
PSME1
EEF1AKMT4-ECE2
TMEM14C
OR6B1
MED18
FDFT1
AGPAT5
RHOU
PLEKHM3
REC8
CHORDC1
MTMR9
CPSF1
SESN2
ADIPOR2
CLDN23
GATA4
MTCH2
MGAT4C
TSSK4
RNF31
ZFP1
FNBP4

RABGGTA
CIDEB
MDP1
LTB4R2
PTPMT1
CPNE6
PAK1IP1
PCK2
SLC39A4
NEDD8
IPO4
MSRA
TMEM249
SGPP1
ABCF2-H2BE1
BOP1
FITM1
NOP9
PPP1R3B
TRIM17
TGM1
EMC9
LRTM2
NDUFS3
ATP5IF1
C3orf52
NEDD8-MDP1
FBXL6
ZYX
ADCK5
LTB4R
DGAT1
NFATC4
NRL
AGBL2
GET1-SH3BGR
ASDURF
DERPC
ARMCX5-GPRASP2
SMIM40
GUCA1ANB
GTF2H1
C1RL
TNRC6A
EIF5A
NOC4L
ACAP1
SLC2A4
IL27RA
PPP1R27
PODNL1
ASF1B
MCRIP1
SLC5A11
PEX5
C19orf67

STING1
TNNT3
STRA6
GRK1
GCGR
SMIM33
DNAJC18
RFX1
CLDN7
CC2D1A
MCTS1
ECSCR
LDHA
NEURL4
CTDNEP1
ADGRL1
GPS2
TWIST2
ARHGAP17
ATP4B
CYP11A1
C1GALT1C1
CTTN
CCDC33
UBL7
CLSTN3
GJA10
HPS5
DCAF15
SEMA7A
RLN3
CASP8AP2
ELP5
SAMD1
FAM20C
DDX51
YBX2
NANOS3
ISLR
TMEM95
C1R
DUS4L-BCAP29

Supplemental File 3: genes with significant evidence of positive selection

TNMD
CALCR
KDM7A
ARHGAP44
ADAM22
MMP25
SCMH1
PLAUR
TYROBP
NUB1
GPRC5A
TACC3
ACP3
SLC11A1
PHLDB1
MARCO
ABCC2
NCAPH2
INSRR
ANK1
ARHGAP31
SARS1
FAM136A
SLC18A1
PI4K2B
CLEC16A
CDH1
BARX2
ZIC2
CP
DTNBP1
XK
ELN
HEXB
COL23A1
NNAT
KCNQ1
TRAPPC3
EIF2AK2
MCOLN3
ITIH1
TRAF1
F7
TMCC3
RIMBP2
BCAT1
CDH3
CA11
ST3GAL6
ANKS1A
SPEN
FAM107B

ISOC1
ZFAT
DNTTIP2
CDHR2
ZZEF1
MCAM
STXBP2
MCCC1
P2RY10
TP73
CDH17
KIF22
CARMIL1
SMARCD3
WDR70
BCKDHB
ITGAE
DIS3
CYLD
SLCO1A2
MYBPC2
TRIP6
SH3BP2
NID2
RFX2
SLC4A11
KIF16B
FUS
ARHGAP4
NDUFB2
IRAK3
LYZ
SI
PLEKHG2
TF
SCFD1
SNAP23
XYLB
GABRP
NUP188
SH2D3C
DSP
SETD1A
TRMT2A
TBC1D10A
HSCB
LMF2
HIF1A
ZFYVE21
PCNX1
RPS6KA5
YY1
ACIN1
MMP9
ABHD12
CTSZ

GID8
PLCB4
SAMHD1
MAP1LC3A
CEP76
MIB1
SRPX
SMS
MAGED2
UGGT2
MMP15
SETD6
ZNF174
AQP8
BCKDK
SYT17
RPAP1
SPG11
SGK3
RP1
FCGRT
TRMT1
DMPK
SGTA
PRX
NUMBL
HNRNPUL1
COMP
LSR
SCN1B
PON3
NPVF
WASL
SPAM1
IMPDH1
CORO2A
C5
DDX58
RAPGEF1
GATA3
NSMCE4A
PALD1
MICU1
TLX1
EDRF1
AKAP10
COL1A1
VAT1
INPP4B
GLRB
PPARGC1A
SIAE
DTX4
ATG2A
APOA5
PDHX

MADD
ASIC1
SLC11A2
FOXM1
ENDOU
ATN1
PTPN6
SRSF9
RNGTT
SLC26A8
RHAG
COL9A1
MED23
VNN1
PERP
TMEM30A
TTK
HARS2
DROSHA
SLC27A6
GNPDA1
HMGXB3
PDGFRB
CLDN16
XRN1
NEK11
HEMK1
MAPKAPK3
CLCN2
NEK4
NCL
CENPA
GCA
PECR
EHBP1
SLC9A2
BIRC6
LCT
C2orf42
QSOX1
FBXO2
CRYZ
EXOC8
ACADM
PRDX6
ESYT2
A4GNT
IFT46
TNNT2
FASTKD2
ATP10B
SPACA1
ZC2HC1B
VAMP8
RPN2
SENP5

HEATR1
CNTRL
SLIRP
IFIT2
PYROXD2
MXI1
PANK3
PLEKHG1
MTERF2
TBX2
DPPA4
CRY2
PLG
RBBP6
NEUROG3
EBPL
ITIH5
MXD4
MOGAT1
GCNT7
ZNFX1
VAMP7
LYPD3
CRISP2
TRERF1
HROB
GTF2F1
ALKBH7
ATG4C
GPCPD1
BFSP1
NCLN
FRMD8
PRR12
AHDC1
KTN1
RGS13
TAS2R4
PLA2G5
EPS15L1
KDR
MGAT3
LIF
CCDC32
DLL4
SUMF2
CSRP3
DCTD
PLD2
PHF20L1
LDLR
ACE2
FAM98C
ACSBG2
MLLT1
METTL26

GMFG
SMPDL3B
HIP1R
ANGPTL6
DKC1
ZNF341
DIAPH1
NPHP4
TNS4
SNRPA1
ACTR10
TRIM21
IMMT
RAMP1
PTPRE
LANCL2
KDM6B
GUCY2D
MTSS2
RIN2
KANK4
LGR6
TMCC2
GPALPP1
SLC39A11
ZNF414
RTN3
ATP13A3
DYDC2
MEN1
CHL1
PRPF38B
RAX
DSC2
DSG1
DSC1
UBAC2
CARS2
HNF1A
KRT85
NHSL1
KIAA0513
KCNK1
TAF5L
RNASEL
DNAJB2
COX5B
EDNRB
GPNMB
TBRG4
ZFHX2
MARCHF7
KCTD3
TXN
DAB2IP
ZNF189

HEMGN
FAM8A1
GGH
NEK1
SORL1
GIPC2
CGREF1
ATRAID
TRIM54
ENTPD1
ANXA7
FAM149B1
HECW2
COX17
MNS1
FGF5
PPA2
PARVG
SCAF11
SLC15A4
TARBP2
NIPA2
BAHD1
PIF1
LINS1
LMAN1L
SLC5A2
DEF8
PCTP
SLC39A6
ZMYND15
NARF
ZNF750
NFIC
PLPP2
FEM1A
VAV1
DOP1B
ILDR2
NUF2
ISG20L2
RORC
SNX27
RFX5
POGZ
DTL
SOX13
PLEKHA6
ATP6V1C2
ASXL2
ABHD1
NXPH2
SPAG16
NR1I2
SRPRB
UCN2

AHSG
PLAC8
NAF1
ROPN1L
SSBP2
SLC25A48
TNFRSF21
DAAM2
PRSS35
AIG1
TMEM181
NOM1
ASB10
SH3KBP1
ZMYM3
AWAT2
ST18
TACC1
ST8SIA6
HTR7
CYP17A1
TUT1
HSD17B12
DGKZ
GLYAT
SERPING1
ARFGAP2
CCDC82
CHEK1
COMMD7
KIAA1755
CDH22
TRPT1
DLAT
PTS
SCLT1
FAM160B1
EDNRA
BEND6
SETBP1
BMP3
CPB1
TMEM87B
ZDHHC7
TBCEL
CC2D1B
DIPK1A
AK9
OXA1L
LARP1
CYLC2
ART3
SCAF4
SUPV3L1
PDE6D
FCHO2

C8A
SYN2
PWWP3B
ETS2
MX1
UBN2
SKI
WIPI2
KRTCAP3
PAFAH2
ESYT3
TENT5B
PEX2
LARGE2
MYL1
CHRM1
TCTN2
ATOH8
COL4A3
TBC1D10B
ZRSR2
PTAFR
NPR1
MUC15
HIC2
ITGAM
KCNAB3
ZNF282
GLB1
EMB
OR9K2
RNF139
MBD3L1
ALK
CLDN20
SCAND1
ZNF318
MAP6
FGG
CXXC5
SYCE1
IFNB1
DRC3
LAMB2
HINFP
CORO1B
MUS81
METAP1D
EXO1
GTF2IRD2B
STARD6
DES
OR52W1
HSF5
GJC3
CLEC14A

SIX5
CAVIN1
ACAD9
PNPLA2
BOLA1
MSC
PFAS
SNX18
C3orf38
CYC1
GATA2
TMEM31
CDH5
MYADM
RNF227
FIZ1
EXOC3
RCC1
GSX2
CLRN3
PPA1
FAM83H
DIPK2A
KCNIP1
C1S
BGN
C1orf116
GDPGP1
COA5
CCR3
UPP1
ACP7
B3GALT5
SLC35F3
OLFML1
ADAP2
LRTOMT
IRAK1
CMSS1
SRPK3
POU3F2
NUTM1
RNLS
FAM43A
LAMP1
SETD4
SAPCD2
KLK12
SMYD4
UBE2H
KATNA1
RPS19BP1
C5orf52
AMTN
SLC18A3
PEAR1

ZFP69
CYHR1
RILPL1
RAB42
PLA2G4E
LAMTOR4
PLSCR1
SELL
NDOR1
FBLL1
CLEC20A
CC2D2B
QRFP
RBM34
SLC15A5
ALKBH2
NUGGC
WDSUB1
SEMA4A
XPNPEP3
PIK3R4
SLC6A9
ZSCAN25
ZNF420
ARRDC1
PCBP2
PDCD1LG2
CFAP43
SLC22A12
ZNF667
HOXC4
UVRAG
NTRK1
ZXDB
ZNF652
EPS8L3
MT-ND4
ZFP2
KIFBP
ARMCX6
EFCAB2
GLYATL3
GIGYF2
RNASE13
LNP1
ACKR1
CHUK
SIPA1
SYNJ2BP
ADAT3
DDX47
MBLAC1
FOXI3
NOTO
B3GALT9
GPR33

LRRC69
ACSM4
GRXCR1
FASTKD5
SKOR2
CEBPZOS
VAMP2
PRR29
SLC26A6
TEX50
MCIDAS
PSMB8
TMEM250
STPG4
RPP21
ARPC4
C8orf58
NFS1
LY75-CD302
TMEM200B
TRIL
ATP23
DPEP2NB
RNF115
MEI4
DCP1A
SCRIB
PROP1
DERL3
SDCCAG8
APBA2
DACH1
HEATR9
SALL3
ARHGEF26
SCG5
GSTT1
CISD3
GFUS
PPP4R4
MYO19
OR2S2
MATR3
CORO7
ZNF445
EXOC3L2
PERCC1
LYNX1-SLURP2
SPEM1
CARMIL3
SMIM41
AGPAT5
REC8
RNF31
TMEM249
NFATC4

AGBL2
SEMA7A
NANOS3
ISLR
TMEM95
C1R

Supplemental File 4: Results of the enrichment analyses (enriched Panther pathway terms)

geneSet	description	link	size	overlap	expect	enrichmentRatio	pValue	FDR	overlapId	userId
P04375	5HT3 type receptor mediated signaling pathway	http://www.pantherdb.org/pathway/pathwayDiagram.jsp?catAccession=P04375	12	4	0.6238030095759234	6.412.280.701.754.380	0.0024298477859624468	0.1416563342810584	8773;6570;6844;8673	SLC18A1;SNAP23;VAMP8;VAMP2
P00050	Plasminogen activating cascade	http://www.pantherdb.org/pathway/pathwayDiagram.jsp?catAccession=P00050	13	4	0.6757865937072504	5.919.028.340.080.970	0.0033724716922153286	0.1416563342810584	5340;2266;4318;5329	PLAUR;MMP9;PLG;FGG
P00042	Muscarinic acetylcholine receptor 1 and 3 signaling pathway	http://www.pantherdb.org/pathway/pathwayDiagram.jsp?catAccession=P00042	31	6	16.114.911.080.711.300	37.232.597.623.089.900	0.004207613889536388	0.1416563342810584	8773;1128;6844;5332;6572;8673	SNAP23;PLCB4;VAMP8;CHRM1;SLC18A3;VAMP2
P04376	5HT4 type receptor mediated signaling pathway	http://www.pantherdb.org/pathway/pathwayDiagram.jsp?catAccession=P04376	17	4	0.8837209302325582	4.526.315.789.473.680	0.009574804032375539	0.24176380181748236	8773;6570;6844;8673	SLC18A1;SNAP23;VAMP8;VAMP2
P04379	Beta3 adrenergic receptor signaling pathway	http://www.pantherdb.org/pathway/pathwayDiagram.jsp?catAcc	13	3	0.6757865937072504	4.439.271.255.060.720	0.026487417645411293	0.32914231755500256	8773;6844;8673	SNAP23;VAMP8;VAMP2

		ession=P 04379								
P04374	5HT2 type receptor mediated signaling pathway	http://www.pantherdb.org/pathway/pathwayDiagram.jsp?catAccession=P04374	35	5	18.194.254.445.964.400	27.481.203.007.518.700	0.031719285219601656	0.32914231755500256	8773;6570;6844;5332;8673	SLC18A1;SNAP23;PLCB4;VAMP8;VAMP2
P05916	Opioid prodynorphin pathway	http://www.pantherdb.org/pathway/pathwayDiagram.jsp?catAccession=P05916	14	3	0.7277701778385772	412.218.045.112.782	0.032473897154464026	0.32914231755500256	8773;6844;8673	SNAP23;VAMP8;VAMP2
P04380	Cortocotropin releasing factor receptor signaling pathway	http://www.pantherdb.org/pathway/pathwayDiagram.jsp?catAccession=P04380	14	3	0.7277701778385772	412.218.045.112.782	0.032473897154464026	0.32914231755500256	8773;6844;8673	SNAP23;VAMP8;VAMP2
P00011	Blood coagulation	http://www.pantherdb.org/pathway/pathwayDiagram.jsp?catAccession=P00011	25	4	12.995.896.032.831.700	30.778.947.368.421.000	0.03712621268692873	0.32914231755500256	5340;2266;2155;5329	PLAUR;F7;PLG;FGG
P04377	Beta1 adrenergic receptor signaling pathway	http://www.pantherdb.org/pathway/pathwayDiagram.jsp?catAccession=P04377	15	3	0.7797537619699042	38.473.684.210.526.300	0.03910601792732704	0.32914231755500256	8773;6844;8673	SNAP23;VAMP8;VAMP2
P05917	Opioid proopiomelanocortin pathway	http://www.pantherdb.org/pathway/pathwayDiagram.jsp?catAccession=P05917	15	3	0.7797537619699042	38.473.684.210.526.300	0.03910601792732704	0.32914231755500256	8773;6844;8673	SNAP23;VAMP8;VAMP2

P00030	Hypoxia response via HIF activation	http://www.pantherdb.org/pathway/pathwayDiagram.jsp?catAccession=P00030	15	3	0.7797537619699042	38.473.684.210.526.300	0.03910601792732704	0.32914231755500256	7295;6097;3091	HIF1A;TXN;RORC
P05915	Opioid proenkephalin pathway	http://www.pantherdb.org/pathway/pathwayDiagram.jsp?catAccession=P05915	16	3	0.8317373461012312	3.606.907.894.736.840	0.04637240510125218	0.3345437796590336	8773;6844;8673	SNAP23;VAMP8;VAMP2
P04378	Beta2 adrenergic receptor signaling pathway	http://www.pantherdb.org/pathway/pathwayDiagram.jsp?catAccession=P04378	16	3	0.8317373461012312	3.606.907.894.736.840	0.04637240510125218	0.3345437796590336	8773;6844;8673	SNAP23;VAMP8;VAMP2
P04391	Oxytocin receptor mediated signaling pathway	http://www.pantherdb.org/pathway/pathwayDiagram.jsp?catAccession=P04391	28	4	14.555.403.556.771.500	274.812.030.075.188	0.05344445085032723	0.34250113364750123	8773;6844;5332;8673	SNAP23;PLCB4;VAMP8;VAMP2
P00002	Alpha adrenergic receptor signaling pathway	http://www.pantherdb.org/pathway/pathwayDiagram.jsp?catAccession=P00002	17	3	0.8837209302325582	33.947.368.421.052.600	0.05425760533029722	0.34250113364750123	8773;6844;5332	SNAP23;PLCB4;VAMP2
P00040	Metabotropic glutamate receptor group II pathway	http://www.pantherdb.org/pathway/pathwayDiagram.jsp?catAccession=P00040	18	3	0.9357045143638851	32.061.403.508.771.900	0.06274269922382636	0.3727654483297919	8773;6844;8673	SNAP23;VAMP8;VAMP2

P04394	Thyrotropin-releasing hormone receptor signaling pathway	http://www.pantherdb.org/pathway/pathwayDiagram.jsp?catAccession=P04394	32	4	16.634.746.922.024.600	24.046.052.631.578.900	0.08035734910526449	0.4036969779622145	8773;6844;5332;8673	SNAP23;PLCB4;VAMP8;VAMP2
P00043	Muscarinic acetylcholine receptor 2 and 4 signaling pathway	http://www.pantherdb.org/pathway/pathwayDiagram.jsp?catAccession=P00043	32	4	16.634.746.922.024.600	24.046.052.631.578.900	0.08035734910526449	0.4036969779622145	8773;6844;6572;8673	SNAP23;VAMP8;SLC18A3;VAMP2
P04373	5HT1 type receptor mediated signaling pathway	http://www.pantherdb.org/pathway/pathwayDiagram.jsp?catAccession=P04373	20	3	1.039.671.682.626.530	28.855.263.157.894.700	0.08142282684026414	0.4036969779622145	8773;6570;6844	SLC18A1;SNAP23;VAMP2
P00001	Adrenaline and noradrenaline biosynthesis	http://www.pantherdb.org/pathway/pathwayDiagram.jsp?catAccession=P00001	21	3	10.916.552.667.578.600	274.812.030.075.188	0.09156740588423673	0.4036969779622145	8773;6570;6844	SLC18A1;SNAP23;VAMP2
P00044	Nicotinic acetylcholine receptor signaling pathway	http://www.pantherdb.org/pathway/pathwayDiagram.jsp?catAccession=P00044	47	5	2.443.228.454.172.360	20.464.725.643.896.900	0.09259811281453945	0.4036969779622145	8773;6844;6572;80179;8673	SNAP23;VAMP8;SLC18A3;VAMP2;MYO19
P00054	Toll receptor signaling pathway	http://www.pantherdb.org/pathway/pathwayDiagram.jsp?catAccession=P00054	34	4	17.674.418.604.651.100	2.263.157.894.736.840	0.0959279947632985	0.4036969779622145	1147;3456;3654;11213	IRAK3;IFNB1;IRAK1;CHUK

P00010	B cell activation	http://www.pantherdb.org/pathway/pathwayDiagram.jsp?catAccession=P00010	34	4	17.674.418.604.651.100	2.263.157.894.736.840	0.0959279947632985	0.4036969779622145	1147;7409;5777;4776	PTPN6;VAV1;CHUK;NFATC4
P00037	Ionotropic glutamate receptor pathway	http://www.pantherdb.org/pathway/pathwayDiagram.jsp?catAccession=P00037	22	3	11.436.388.508.891.900	2.623.205.741.626.790	0.10221182192482403	0.40394781329513557	8773;6844;8673	SNAP23;VAMP8;VAMP2
P00012	Cadherin signaling pathway	http://www.pantherdb.org/pathway/pathwayDiagram.jsp?catAccession=P00012	63	6	3.274.965.800.273.590	1.832.080.200.501.250	0.10398656579874777	0.40394781329513557	999;1001;1003;1015;54825;64405	CDH1;CDH3;CDHR2;CDH17;CDH22;CDH5
P00039	Metabotropic glutamate receptor group III pathway	http://www.pantherdb.org/pathway/pathwayDiagram.jsp?catAccession=P00039	27	3	14.035.567.715.458.200	21.374.269.005.847.900	0.16189264740208342	0.6055984217633491	8773;6844;8673	SNAP23;VAMP8;VAMP2
P00005	Angiogenesis	http://www.pantherdb.org/pathway/pathwayDiagram.jsp?catAccession=P00005	105	8	5.458.276.333.789.320	14.656.641.604.010.000	0.1728503541628399	0.6091568803385425	5338;2155;5159;5777;3791;7867;3091;54567	F7;HIF1A;PTPN6;PDGFRB;MAPKAPK3;KDR;DLL4;PLD2
P05912	Dopamine receptor mediated signaling pathway	http://www.pantherdb.org/pathway/pathwayDiagram.jsp?catAccession=P05912	28	3	14.555.403.556.771.500	206.109.022.556.391	0.1749064309882944	0.6091568803385425	8773;6844;8673	SNAP23;VAMP8;VAMP2

P00029	Huntington disease	http://www.pantherdb.org/pathway/pathwayDiagram.jsp?catAccession=P00029	77	6	4.002.735.978.112.170	14.989.747.095.010.200	0.2064538841562351	0.6950614099926582	8976;7161;9026;1537;1429;10493	TP73;WASL;VAT1;CRYZ;HIP1R;CYC1
P00031	Inflammation mediated by chemokine and cytokine signaling pathway	http://www.pantherdb.org/pathway/pathwayDiagram.jsp?catAccession=P00031	95	7	493.844.049.247.606	14.174.515.235.457.000	0.21908138076587602	0.7137812728178542	6003;1147;7409;1232;10093;4776;5332	PLCB4;RGS13;VAV1;CCR3;CHUK;ARPC4;NFATC4
P00016	Cytoskeletal regulation by Rho GTPase	http://www.pantherdb.org/pathway/pathwayDiagram.jsp?catAccession=P00016	32	3	16.634.746.922.024.600	1.803.453.947.368.420	0.2295393429005027	0.7244835510297116	8976;1729;10093	WASL;DIAPH1;ARPC4
P00032	Insulin/IGF pathway-mitogen activated protein kinase kinase/MAP kinase cascade	http://www.pantherdb.org/pathway/pathwayDiagram.jsp?catAccession=P00032	20	2	1.039.671.682.626.530	19.236.842.105.263.100	0.27910502929172964	0.8513988124074395	9252;3645	INSRR;RPS6KA5
P00004	Alzheimer disease-presenilin pathway	http://www.pantherdb.org/pathway/pathwayDiagram.jsp?catAccession=P00004	69	5	35.868.673.050.615.500	13.939.740.655.987.700	0.2866095012064648	0.8513988124074395	999;4318;1001;4324;64386	MMP25;CDH1;CDH3;MMP9;MMP15
P00015	Circadian clock system	http://www.pantherdb.org/pathway/pathwayDiagram.jsp?catAccession=P00015	7	1	0.3638850889192886	274.812.030.075.188	0.31234929122694677	0.8565768531778164	1408	CRY2

P04392	P53 pathway feedback loops 1	http://www.pantherdb.org/pathway/pathwayDiagram.jsp?catAccession=P04392	7	1	0.3638850889192886	274.812.030.075.188	0.31234929122694677	0.8565768531778164	7161	TP73
P00057	Wnt signaling pathway	http://www.pantherdb.org/pathway/pathwayDiagram.jsp?catAccession=P00057	143	9	7.433.652.530.779.750	12.107.103.422.892.800	0.32213056350334046	0.8565768531778164	999;1001;1003;1015;4776;5332;6604;54825;64405	CDH1;CDH3;CDHR2;CDH17;SMARCD3;PLCB4;CDH22;CDH5;NFATC4
P00056	VEGF signaling pathway	http://www.pantherdb.org/pathway/pathwayDiagram.jsp?catAccession=P00056	39	3	2.027.359.781.121.750	1.479.757.085.020.240	0.3307573987518301	0.8565768531778164	3791;7867;3091	HIF1A;MAPKAPK3;KDR
P00053	T cell activation	http://www.pantherdb.org/pathway/pathwayDiagram.jsp?catAccession=P00053	39	3	2.027.359.781.121.750	1.479.757.085.020.240	0.3307573987518301	0.8565768531778164	1147;7409;4776	VAV1;CHUK;NFATC4
P00041	Metabotropic glutamate receptor group I pathway	http://www.pantherdb.org/pathway/pathwayDiagram.jsp?catAccession=P00041	9	1	0.46785225718194257	21.374.269.005.847.900	0.38233359749169293	0.930638674594358	5332	PLCB4
P00034	Integrin signalling pathway	http://www.pantherdb.org/pathway/pathwayDiagram.jsp?catAcc	115	7	5.978.112.175.102.590	11.709.382.151.029.700	0.38945588373869144	0.930638674594358	1277;3684;1297;3682;3913;128	ITGAE;RAPGEF1;COL1A1;COL9A1;COL4A3;ITGAM;LAMB2

		ession=P00034							5;2889	
P00023	General transcription regulation	http://www.pantherdb.org/pathway/pathwayDiagram.jsp?catAccession=P00023	26	2	13.515.731.874.145.000	14.797.570.850.202.400	0.39556105889122783	0.930638674594358	2962;80298	MTERF2;GTF2F1
P00019	Endothelin signaling pathway	http://www.pantherdb.org/pathway/pathwayDiagram.jsp?catAccession=P00019	44	3	22.872.777.017.783.800	1.311.602.870.813.390	0.4035649277558101	0.930638674594358	1910;1909;5332	PLCB4;EDNRB;EDNRA
P05918	p38 MAPK pathway	http://www.pantherdb.org/pathway/pathwayDiagram.jsp?catAccession=P05918	27	2	14.035.567.715.458.200	142.495.126.705.653	0.41425453181035976	0.930638674594358	9252;7867	RPS6KA5;MAPKAPK3
P02775	Salvage pyrimidine ribonucleotides	http://www.pantherdb.org/pathway/pathwayDiagram.jsp?catAccession=P02775	10	1	0.5198358413132695	19.236.842.105.263.100	0.4146409936311496	0.930638674594358	7378	UPP1

Supplemental File 5: Redundancy in terms reduced with affinity propagation

P04 375	P04 376	P04 374	P04 373	P00 001								
P00 050	P00 011											
P04 379	P05 916	P04 380	P04 377	P05 917	P05 915	P04 378	P00 040	P00 043	P00 044	P00 037	P00 039	P05 912
P00 030												
P04 391	P00 042	P00 002	P04 394	P00 041	P00 019							
P00 054												
P00 010	P00 031	P00 053										
P00 012	P00 004	P00 057										
P00 005	P00 056											
P00 029	P00 016	P04 392										
P00 032	P05 918											
P00 015												
P00 034												
P00 023												
P02 775												

Supplemental File 6: Redundancy in terms reduced with weighted set crossover

Coverage: 0.790322580645161
P00042
P00050
P00005
P00012
P00029
P00054
P00031
P00034
P00030
P04375

Supplemental File 7: GO Slim summary of Biological Process

GO:0065007	biological regulation	426
GO:0008152	metabolic process	406
GO:0050896	response to stimulus	316
GO:0032501	multicellular organismal process	279
GO:0051179	localization	250
GO:0016043	cellular component organization	241
GO:0032502	developmental process	238
GO:0007154	cell communication	213
GO:0051704	multi-organism process	86
GO:0008283	cell proliferation	79
GO:0000003	reproduction	50
GO:0040007	growth	27

Supplemental File 8: GO Slim summary of Cellular Component

GO:0016020	membrane	325
GO:0005634	nucleus	249
GO:0032991	protein-containing complex	190
GO:0031974	membrane-enclosed lumen	185
GO:0012505	endomembrane system	182
GO:0005829	cytosol	174
GO:0031982	vesicle	163
GO:0005615	extracellular space	112
GO:0042995	cell projection	91
GO:0005856	cytoskeleton	77
GO:0005783	endoplasmic reticulum	70
GO:0005739	mitochondrion	59
GO:0005794	Golgi apparatus	52
GO:0005773	vacuole	42
GO:0005768	endosome	37
GO:0031975	envelope	35
GO:0005694	chromosome	31
GO:0031012	extracellular matrix	26
GO:0042579	microbody	3
GO:0005811	lipid droplet	3
GO:0005840	ribosome	2

Supplemental File 9: GO Slim summary of Molecular Function

GO:0005515	protein binding	433
GO:0043167	ion binding	229
GO:0003676	nucleic acid binding	147
GO:0016740	transferase activity	94
GO:0016787	hydrolase activity	92
GO:0000166	nucleotide binding	67
GO:0005215	transporter activity	47
GO:0060089	molecular transducer activity	37
GO:0030234	enzyme regulator activity	36
GO:0008289	lipid binding	30
GO:0005198	structural molecule activity	28
GO:0003682	chromatin binding	24
GO:0060090	molecular adaptor activity	12
GO:0030246	carbohydrate binding	8
GO:0009055	electron transfer activity	3
GO:0045182	translation regulator activity	2
GO:0016209	antioxidant activity	2
GO:0019825	oxygen binding	1

Supplemental File 10: Category terms for the user uploaded gene IDs and positively selected gene annotations

userId	geneSymbol	geneName	entrezgene	gLink
TNMD	TNMD	tenomodulin	64102	https://www.ncbi.nlm.nih.gov/gene/?term=64102
CALCR	CALCR	calcitonin receptor	799	https://www.ncbi.nlm.nih.gov/gene/?term=799
KDM7A	KDM7A	lysine demethylase 7A	80853	https://www.ncbi.nlm.nih.gov/gene/?term=80853
ARHGAP44	ARHGAP44	Rho GTPase activating protein 44	9912	https://www.ncbi.nlm.nih.gov/gene/?term=9912
ADAM22	ADAM22	ADAM metallopeptidase domain 22	53616	https://www.ncbi.nlm.nih.gov/gene/?term=53616
MMP25	MMP25	matrix metallopeptidase 25	64386	https://www.ncbi.nlm.nih.gov/gene/?term=64386
SCMH1	SCMH1	Scm polycomb group protein homolog 1	22955	https://www.ncbi.nlm.nih.gov/gene/?term=22955
PLAUR	PLAUR	plasminogen activator, urokinase receptor	5329	https://www.ncbi.nlm.nih.gov/gene/?term=5329
TYROBP	TYROBP	TYRO protein tyrosine kinase binding protein	7305	https://www.ncbi.nlm.nih.gov/gene/?term=7305
NUB1	NUB1	negative regulator of ubiquitin like proteins 1	51667	https://www.ncbi.nlm.nih.gov/gene/?term=51667
GPRC5A	GPRC5A	G protein-coupled receptor class C group 5 member A	9052	https://www.ncbi.nlm.nih.gov/gene/?term=9052
TACC3	TACC3	transforming acidic coiled-coil containing protein 3	10460	https://www.ncbi.nlm.nih.gov/gene/?term=10460
SLC11A1	SLC11A1	solute carrier family 11 member 1	6556	https://www.ncbi.nlm.nih.gov/gene/?term=6556
PHLDB1	PHLDB1	pleckstrin homology like domain family B member 1	23187	https://www.ncbi.nlm.nih.gov/gene/?term=23187
MARCO	MARCO	macrophage receptor with collagenous structure	8685	https://www.ncbi.nlm.nih.gov/gene/?term=8685
ABCC2	ABCC2	ATP binding cassette subfamily C member 2	1244	https://www.ncbi.nlm.nih.gov/gene/?term=1244
NCAPH2	NCAPH2	non-SMC condensin II complex subunit H2	29781	https://www.ncbi.nlm.nih.gov/gene/?term=29781
INSRR	INSRR	insulin receptor related receptor	3645	https://www.ncbi.nlm.nih.gov/gene/?term=3645
ANK1	ANK1	ankyrin 1	286	https://www.ncbi.nlm.nih.gov/gene/?term=286
ARHGAP31	ARHGAP31	Rho GTPase activating protein 31	57514	https://www.ncbi.nlm.nih.gov/gene/?term=57514
FAM136A	FAM136A	family with sequence similarity 136 member A	84908	https://www.ncbi.nlm.nih.gov/gene/?term=84908
SLC18A1	SLC18A1	solute carrier family 18 member A1	6570	https://www.ncbi.nlm.nih.gov/gene/?term=6570

PI4K2B	PI4K2B	phosphatidylinositol 4-kinase type 2 beta	55300	https://www.ncbi.nlm.nih.gov/gene/?term=55300
CLEC16A	CLEC16A	C-type lectin domain containing 16A	23274	https://www.ncbi.nlm.nih.gov/gene/?term=23274
CDH1	CDH1	cadherin 1	999	https://www.ncbi.nlm.nih.gov/gene/?term=999
BARX2	BARX2	BARX homeobox 2	8538	https://www.ncbi.nlm.nih.gov/gene/?term=8538
ZIC2	ZIC2	Zic family member 2	7546	https://www.ncbi.nlm.nih.gov/gene/?term=7546
CP	CP	ceruloplasmin	1356	https://www.ncbi.nlm.nih.gov/gene/?term=1356
DTNBP1	DTNBP1	dystrobrevin binding protein 1	84062	https://www.ncbi.nlm.nih.gov/gene/?term=84062
XK	XK	X-linked Kx blood group	7504	https://www.ncbi.nlm.nih.gov/gene/?term=7504
ELN	ELN	elastin	2006	https://www.ncbi.nlm.nih.gov/gene/?term=2006
HEXB	HEXB	hexosaminidase subunit beta	3074	https://www.ncbi.nlm.nih.gov/gene/?term=3074
COL23A1	COL23A1	collagen type XXIII alpha 1 chain	91522	https://www.ncbi.nlm.nih.gov/gene/?term=91522
NNAT	NNAT	neuronatin	4826	https://www.ncbi.nlm.nih.gov/gene/?term=4826
KCNQ1	KCNQ1	potassium voltage-gated channel subfamily Q member 1	3784	https://www.ncbi.nlm.nih.gov/gene/?term=3784
TRAPPC3	TRAPPC3	trafficking protein particle complex 3	27095	https://www.ncbi.nlm.nih.gov/gene/?term=27095
EIF2AK2	EIF2AK2	eukaryotic translation initiation factor 2 alpha kinase 2	5610	https://www.ncbi.nlm.nih.gov/gene/?term=5610
MCOLN3	MCOLN3	mucolipin 3	55283	https://www.ncbi.nlm.nih.gov/gene/?term=55283
ITIH1	ITIH1	inter-alpha-trypsin inhibitor heavy chain 1	3697	https://www.ncbi.nlm.nih.gov/gene/?term=3697
TRAF1	TRAF1	TNF receptor associated factor 1	7185	https://www.ncbi.nlm.nih.gov/gene/?term=7185
F7	F7	coagulation factor VII	2155	https://www.ncbi.nlm.nih.gov/gene/?term=2155
TMCC3	TMCC3	transmembrane and coiled-coil domain family 3	57458	https://www.ncbi.nlm.nih.gov/gene/?term=57458
RIMBP2	RIMBP2	RIMS binding protein 2	23504	https://www.ncbi.nlm.nih.gov/gene/?term=23504
BCAT1	BCAT1	branched chain amino acid transaminase 1	586	https://www.ncbi.nlm.nih.gov/gene/?term=586
CDH3	CDH3	cadherin 3	1001	https://www.ncbi.nlm.nih.gov/gene/?term=1001
CA11	CA11	carbonic anhydrase 11	770	https://www.ncbi.nlm.nih.gov/gene/?term=770
ST3GAL6	ST3GAL6	ST3 beta-galactoside alpha-2,3-sialyltransferase 6	10402	https://www.ncbi.nlm.nih.gov/gene/?term=10402

ANKS1A	ANKS1A	ankyrin repeat and sterile alpha motif domain containing 1A	23294	https://www.ncbi.nlm.nih.gov/gene/?term=23294
SPEN	SPEN	spen family transcriptional repressor	23013	https://www.ncbi.nlm.nih.gov/gene/?term=23013
FAM107B	FAM107B	family with sequence similarity 107 member B	83641	https://www.ncbi.nlm.nih.gov/gene/?term=83641
ISOC1	ISOC1	isochorismatase domain containing 1	51015	https://www.ncbi.nlm.nih.gov/gene/?term=51015
ZFAT	ZFAT	zinc finger and AT-hook domain containing	57623	https://www.ncbi.nlm.nih.gov/gene/?term=57623
DNTTIP2	DNTTIP2	deoxynucleotidyltransferase terminal interacting protein 2	30836	https://www.ncbi.nlm.nih.gov/gene/?term=30836
CDHR2	CDHR2	cadherin related family member 2	54825	https://www.ncbi.nlm.nih.gov/gene/?term=54825
ZZEF1	ZZEF1	zinc finger ZZ-type and EF-hand domain containing 1	23140	https://www.ncbi.nlm.nih.gov/gene/?term=23140
MCAM	MCAM	melanoma cell adhesion molecule	4162	https://www.ncbi.nlm.nih.gov/gene/?term=4162
STXBP2	STXBP2	syntaxin binding protein 2	6813	https://www.ncbi.nlm.nih.gov/gene/?term=6813
MCCC1	MCCC1	methylcrotonoyl-CoA carboxylase 1	56922	https://www.ncbi.nlm.nih.gov/gene/?term=56922
P2RY10	P2RY10	P2Y receptor family member 10	27334	https://www.ncbi.nlm.nih.gov/gene/?term=27334
TP73	TP73	tumor protein p73	7161	https://www.ncbi.nlm.nih.gov/gene/?term=7161
CDH17	CDH17	cadherin 17	1015	https://www.ncbi.nlm.nih.gov/gene/?term=1015
KIF22	KIF22	kinesin family member 22	3835	https://www.ncbi.nlm.nih.gov/gene/?term=3835
CARMIL1	CARMIL1	capping protein regulator and myosin 1 linker 1	55604	https://www.ncbi.nlm.nih.gov/gene/?term=55604
SMARCD3	SMARCD3	SWI/SNF related, matrix associated, actin dependent regulator of chromatin, subfamily d, member 3	6604	https://www.ncbi.nlm.nih.gov/gene/?term=6604
WDR70	WDR70	WD repeat domain 70	55100	https://www.ncbi.nlm.nih.gov/gene/?term=55100
BCKDHB	BCKDHB	branched chain keto acid dehydrogenase E1 subunit beta	594	https://www.ncbi.nlm.nih.gov/gene/?term=594
ITGAE	ITGAE	integrin subunit alpha E	3682	https://www.ncbi.nlm.nih.gov/gene/?term=3682
DIS3	DIS3	DIS3 homolog, exosome endoribonuclease and 3'-5' exoribonuclease	22894	https://www.ncbi.nlm.nih.gov/gene/?term=22894

CYLD	CYLD	CYLD lysine 63 deubiquitinase	1540	https://www.ncbi.nlm.nih.gov/gene/?term=1540
SLCO1A2	SLCO1A2	solute carrier organic anion transporter family member 1A2	6579	https://www.ncbi.nlm.nih.gov/gene/?term=6579
MYBPC2	MYBPC2	myosin binding protein C, fast type	4606	https://www.ncbi.nlm.nih.gov/gene/?term=4606
TRIP6	TRIP6	thyroid hormone receptor interactor 6	7205	https://www.ncbi.nlm.nih.gov/gene/?term=7205
SH3BP2	SH3BP2	SH3 domain binding protein 2	6452	https://www.ncbi.nlm.nih.gov/gene/?term=6452
NID2	NID2	nidogen 2	22795	https://www.ncbi.nlm.nih.gov/gene/?term=22795
RFX2	RFX2	regulatory factor X2	5990	https://www.ncbi.nlm.nih.gov/gene/?term=5990
SLC4A11	SLC4A11	solute carrier family 4 member 11	83959	https://www.ncbi.nlm.nih.gov/gene/?term=83959
KIF16B	KIF16B	kinesin family member 16B	55614	https://www.ncbi.nlm.nih.gov/gene/?term=55614
FUS	FUS	FUS RNA binding protein	2521	https://www.ncbi.nlm.nih.gov/gene/?term=2521
ARHGAP4	ARHGAP4	Rho GTPase activating protein 4	393	https://www.ncbi.nlm.nih.gov/gene/?term=393
NDUFB2	NDUFB2	NADH:ubiquinone oxidoreductase subunit B2	4708	https://www.ncbi.nlm.nih.gov/gene/?term=4708
IRAK3	IRAK3	interleukin 1 receptor associated kinase 3	11213	https://www.ncbi.nlm.nih.gov/gene/?term=11213
LYZ	LYZ	lysozyme	4069	https://www.ncbi.nlm.nih.gov/gene/?term=4069
SI	SI	sucrase-isomaltase	6476	https://www.ncbi.nlm.nih.gov/gene/?term=6476
PLEKHG2	PLEKHG2	pleckstrin homology and RhoGEF domain containing G2	64857	https://www.ncbi.nlm.nih.gov/gene/?term=64857
TF	TF	transferrin	7018	https://www.ncbi.nlm.nih.gov/gene/?term=7018
SCFD1	SCFD1	sec1 family domain containing 1	23256	https://www.ncbi.nlm.nih.gov/gene/?term=23256
SNAP23	SNAP23	synaptosome associated protein 23	8773	https://www.ncbi.nlm.nih.gov/gene/?term=8773
XYLB	XYLB	xylulokinase	9942	https://www.ncbi.nlm.nih.gov/gene/?term=9942
GABRP	GABRP	gamma-aminobutyric acid type A receptor pi subunit	2568	https://www.ncbi.nlm.nih.gov/gene/?term=2568
NUP188	NUP188	nucleoporin 188	23511	https://www.ncbi.nlm.nih.gov/gene/?term=23511
SH2D3C	SH2D3C	SH2 domain containing 3C	10044	https://www.ncbi.nlm.nih.gov/gene/?term=10044
DSP	DSP	desmoplakin	1832	https://www.ncbi.nlm.nih.gov/gene/?term=1832
SETD1A	SETD1A	SET domain containing 1A, histone	9739	https://www.ncbi.nlm.nih.gov/gene/?term=9739

		lysine methyltransferase		
TRMT2A	TRMT2A	tRNA methyltransferase 2 homolog A	27037	https://www.ncbi.nlm.nih.gov/gene/?term=27037
TBC1D10A	TBC1D10A	TBC1 domain family member 10A	83874	https://www.ncbi.nlm.nih.gov/gene/?term=83874
HSCB	HSCB	HscB mitochondrial iron-sulfur cluster cochaperone	150274	https://www.ncbi.nlm.nih.gov/gene/?term=150274
LMF2	LMF2	lipase maturation factor 2	91289	https://www.ncbi.nlm.nih.gov/gene/?term=91289
HIF1A	HIF1A	hypoxia inducible factor 1 subunit alpha	3091	https://www.ncbi.nlm.nih.gov/gene/?term=3091
ZFYVE21	ZFYVE21	zinc finger FYVE-type containing 21	79038	https://www.ncbi.nlm.nih.gov/gene/?term=79038
PCNX1	PCNX1	pecanex 1	22990	https://www.ncbi.nlm.nih.gov/gene/?term=22990
RPS6KA5	RPS6KA5	ribosomal protein S6 kinase A5	9252	https://www.ncbi.nlm.nih.gov/gene/?term=9252
YY1	YY1	YY1 transcription factor	7528	https://www.ncbi.nlm.nih.gov/gene/?term=7528
ACIN1	ACIN1	apoptotic chromatin condensation inducer 1	22985	https://www.ncbi.nlm.nih.gov/gene/?term=22985
MMP9	MMP9	matrix metallopeptidase 9	4318	https://www.ncbi.nlm.nih.gov/gene/?term=4318
ABHD12	ABHD12	abhydrolase domain containing 12	26090	https://www.ncbi.nlm.nih.gov/gene/?term=26090
CTSZ	CTSZ	cathepsin Z	1522	https://www.ncbi.nlm.nih.gov/gene/?term=1522
GID8	GID8	GID complex subunit 8 homolog	54994	https://www.ncbi.nlm.nih.gov/gene/?term=54994
PLCB4	PLCB4	phospholipase C beta 4	5332	https://www.ncbi.nlm.nih.gov/gene/?term=5332
SAMHD1	SAMHD1	SAM and HD domain containing deoxynucleoside triphosphate triphosphohydrolase 1	25939	https://www.ncbi.nlm.nih.gov/gene/?term=25939
MAP1LC3A	MAP1LC3A	microtubule associated protein 1 light chain 3 alpha	84557	https://www.ncbi.nlm.nih.gov/gene/?term=84557
CEP76	CEP76	centrosomal protein 76	79959	https://www.ncbi.nlm.nih.gov/gene/?term=79959
MIB1	MIB1	mindbomb E3 ubiquitin protein ligase 1	57534	https://www.ncbi.nlm.nih.gov/gene/?term=57534
SRPX	SRPX	sushi repeat containing protein X-linked	8406	https://www.ncbi.nlm.nih.gov/gene/?term=8406
SMS	SMS	spermine synthase	6611	https://www.ncbi.nlm.nih.gov/gene/?term=6611
MAGED2	MAGED2	MAGE family member D2	10916	https://www.ncbi.nlm.nih.gov/gene/?term=10916

UGGT2	UGGT2	UDP-glucose glycoprotein glucosyltransferase 2	55757	https://www.ncbi.nlm.nih.gov/gene/?term=55757
MMP15	MMP15	matrix metallopeptidase 15	4324	https://www.ncbi.nlm.nih.gov/gene/?term=4324
SETD6	SETD6	SET domain containing 6, protein lysine methyltransferase	79918	https://www.ncbi.nlm.nih.gov/gene/?term=79918
ZNF174	ZNF174	zinc finger protein 174	7727	https://www.ncbi.nlm.nih.gov/gene/?term=7727
AQP8	AQP8	aquaporin 8	343	https://www.ncbi.nlm.nih.gov/gene/?term=343
BCKDK	BCKDK	branched chain ketoacid dehydrogenase kinase	10295	https://www.ncbi.nlm.nih.gov/gene/?term=10295
SYT17	SYT17	synaptotagmin 17	51760	https://www.ncbi.nlm.nih.gov/gene/?term=51760
RPAP1	RPAP1	RNA polymerase II associated protein 1	26015	https://www.ncbi.nlm.nih.gov/gene/?term=26015
SPG11	SPG11	SPG11, spatacsin vesicle trafficking associated	80208	https://www.ncbi.nlm.nih.gov/gene/?term=80208
SGK3	SGK3	serum/glucocorticoid regulated kinase family member 3	23678	https://www.ncbi.nlm.nih.gov/gene/?term=23678
RP1	RP1	RP1, axonemal microtubule associated	6101	https://www.ncbi.nlm.nih.gov/gene/?term=6101
FCGRT	FCGRT	Fc fragment of IgG receptor and transporter	2217	https://www.ncbi.nlm.nih.gov/gene/?term=2217
TRMT1	TRMT1	tRNA methyltransferase 1	55621	https://www.ncbi.nlm.nih.gov/gene/?term=55621
DMPK	DMPK	DM1 protein kinase	1760	https://www.ncbi.nlm.nih.gov/gene/?term=1760
SGTA	SGTA	small glutamine rich tetratricopeptide repeat containing alpha	6449	https://www.ncbi.nlm.nih.gov/gene/?term=6449
PRX	PRX	periaxin	57716	https://www.ncbi.nlm.nih.gov/gene/?term=57716
NUMBL	NUMBL	NUMB like, endocytic adaptor protein	9253	https://www.ncbi.nlm.nih.gov/gene/?term=9253
HNRNPUL1	HNRNPUL1	heterogeneous nuclear ribonucleoprotein U like 1	11100	https://www.ncbi.nlm.nih.gov/gene/?term=11100
COMP	COMP	cartilage oligomeric matrix protein	1311	https://www.ncbi.nlm.nih.gov/gene/?term=1311
LSR	LSR	lipolysis stimulated lipoprotein receptor	51599	https://www.ncbi.nlm.nih.gov/gene/?term=51599
SCN1B	SCN1B	sodium voltage-gated channel beta subunit 1	6324	https://www.ncbi.nlm.nih.gov/gene/?term=6324
PON3	PON3	paraoxonase 3	5446	https://www.ncbi.nlm.nih.gov/gene/?term=5446

NPVF	NPVF	neuropeptide VF precursor	64111	https://www.ncbi.nlm.nih.gov/gene/?term=64111
WASL	WASL	Wiskott-Aldrich syndrome like	8976	https://www.ncbi.nlm.nih.gov/gene/?term=8976
SPAM1	SPAM1	sperm adhesion molecule 1	6677	https://www.ncbi.nlm.nih.gov/gene/?term=6677
IMPDH1	IMPDH1	inosine monophosphate dehydrogenase 1	3614	https://www.ncbi.nlm.nih.gov/gene/?term=3614
CORO2A	CORO2A	coronin 2A	7464	https://www.ncbi.nlm.nih.gov/gene/?term=7464
C5	C5	complement C5	727	https://www.ncbi.nlm.nih.gov/gene/?term=727
DDX58	DDX58	DExD/H-box helicase 58	23586	https://www.ncbi.nlm.nih.gov/gene/?term=23586
RAPGEF 1	RAPGEF1	Rap guanine nucleotide exchange factor 1	2889	https://www.ncbi.nlm.nih.gov/gene/?term=2889
GATA3	GATA3	GATA binding protein 3	2625	https://www.ncbi.nlm.nih.gov/gene/?term=2625
NSMCE4 A	NSMCE4A	NSE4 homolog A, SMC5-SMC6 complex component	54780	https://www.ncbi.nlm.nih.gov/gene/?term=54780
PALD1	PALD1	phosphatase domain containing paladin 1	27143	https://www.ncbi.nlm.nih.gov/gene/?term=27143
MICU1	MICU1	mitochondrial calcium uptake 1	10367	https://www.ncbi.nlm.nih.gov/gene/?term=10367
TLX1	TLX1	T cell leukemia homeobox 1	3195	https://www.ncbi.nlm.nih.gov/gene/?term=3195
EDRF1	EDRF1	erythroid differentiation regulatory factor 1	26098	https://www.ncbi.nlm.nih.gov/gene/?term=26098
AKAP10	AKAP10	A-kinase anchoring protein 10	11216	https://www.ncbi.nlm.nih.gov/gene/?term=11216
COL1A1	COL1A1	collagen type I alpha 1 chain	1277	https://www.ncbi.nlm.nih.gov/gene/?term=1277
VAT1	VAT1	vesicle amine transport 1	10493	https://www.ncbi.nlm.nih.gov/gene/?term=10493
INPP4B	INPP4B	inositol polyphosphate-4-phosphatase type II B	8821	https://www.ncbi.nlm.nih.gov/gene/?term=8821
GLRB	GLRB	glycine receptor beta	2743	https://www.ncbi.nlm.nih.gov/gene/?term=2743
PPARGC 1A	PPARGC1A	PPARG coactivator 1 alpha	10891	https://www.ncbi.nlm.nih.gov/gene/?term=10891
SIAE	SIAE	sialic acid acetylesterase	54414	https://www.ncbi.nlm.nih.gov/gene/?term=54414
DTX4	DTX4	deltex E3 ubiquitin ligase 4	23220	https://www.ncbi.nlm.nih.gov/gene/?term=23220
ATG2A	ATG2A	autophagy related 2A	23130	https://www.ncbi.nlm.nih.gov/gene/?term=23130
APOA5	APOA5	apolipoprotein A5	116519	https://www.ncbi.nlm.nih.gov/gene/?term=116519
PDHX	PDHX	pyruvate dehydrogenase complex component X	8050	https://www.ncbi.nlm.nih.gov/gene/?term=8050

MADD	MADD	MAP kinase activating death domain	8567	https://www.ncbi.nlm.nih.gov/gene/?term=8567
ASIC1	ASIC1	acid sensing ion channel subunit 1	41	https://www.ncbi.nlm.nih.gov/gene/?term=41
SLC11A2	SLC11A2	solute carrier family 11 member 2	4891	https://www.ncbi.nlm.nih.gov/gene/?term=4891
FOXM1	FOXM1	forkhead box M1	2305	https://www.ncbi.nlm.nih.gov/gene/?term=2305
ENDOU	ENDOU	endonuclease, poly(U) specific	8909	https://www.ncbi.nlm.nih.gov/gene/?term=8909
ATN1	ATN1	atrophin 1	1822	https://www.ncbi.nlm.nih.gov/gene/?term=1822
PTPN6	PTPN6	protein tyrosine phosphatase, non-receptor type 6	5777	https://www.ncbi.nlm.nih.gov/gene/?term=5777
SRSF9	SRSF9	serine and arginine rich splicing factor 9	8683	https://www.ncbi.nlm.nih.gov/gene/?term=8683
RNGTT	RNGTT	RNA guanylyltransferase and 5'-phosphatase	8732	https://www.ncbi.nlm.nih.gov/gene/?term=8732
SLC26A8	SLC26A8	solute carrier family 26 member 8	116369	https://www.ncbi.nlm.nih.gov/gene/?term=116369
RHAG	RHAG	Rh associated glycoprotein	6005	https://www.ncbi.nlm.nih.gov/gene/?term=6005
COL9A1	COL9A1	collagen type IX alpha 1 chain	1297	https://www.ncbi.nlm.nih.gov/gene/?term=1297
MED23	MED23	mediator complex subunit 23	9439	https://www.ncbi.nlm.nih.gov/gene/?term=9439
VNN1	VNN1	vanin 1	8876	https://www.ncbi.nlm.nih.gov/gene/?term=8876
PERP	PERP	PERP, TP53 apoptosis effector	64065	https://www.ncbi.nlm.nih.gov/gene/?term=64065
TMEM30A	TMEM30A	transmembrane protein 30A	55754	https://www.ncbi.nlm.nih.gov/gene/?term=55754
TTK	TTK	TTK protein kinase	7272	https://www.ncbi.nlm.nih.gov/gene/?term=7272
HARS2	HARS2	histidyl-tRNA synthetase 2, mitochondrial	23438	https://www.ncbi.nlm.nih.gov/gene/?term=23438
DROSHA	DROSHA	drosha ribonuclease III	29102	https://www.ncbi.nlm.nih.gov/gene/?term=29102
SLC27A6	SLC27A6	solute carrier family 27 member 6	28965	https://www.ncbi.nlm.nih.gov/gene/?term=28965
GNPDA1	GNPDA1	glucosamine-6-phosphate deaminase 1	10007	https://www.ncbi.nlm.nih.gov/gene/?term=10007
HMGXB3	HMGXB3	HMG-box containing 3	22993	https://www.ncbi.nlm.nih.gov/gene/?term=22993
PDGFRB	PDGFRB	platelet derived growth factor receptor beta	5159	https://www.ncbi.nlm.nih.gov/gene/?term=5159
CLDN16	CLDN16	claudin 16	10686	https://www.ncbi.nlm.nih.gov/gene/?term=10686
XRN1	XRN1	5'-3' exoribonuclease 1	54464	https://www.ncbi.nlm.nih.gov/gene/?term=54464

NEK11	NEK11	NIMA related kinase 11	79858	https://www.ncbi.nlm.nih.gov/gene/?term=79858
HEMK1	HEMK1	HemK methyltransferase family member 1	51409	https://www.ncbi.nlm.nih.gov/gene/?term=51409
MAPKAPK3	MAPKAPK3	mitogen-activated protein kinase-activated protein kinase 3	7867	https://www.ncbi.nlm.nih.gov/gene/?term=7867
CLCN2	CLCN2	chloride voltage-gated channel 2	1181	https://www.ncbi.nlm.nih.gov/gene/?term=1181
NEK4	NEK4	NIMA related kinase 4	6787	https://www.ncbi.nlm.nih.gov/gene/?term=6787
NCL	NCL	nucleolin	4691	https://www.ncbi.nlm.nih.gov/gene/?term=4691
CENPA	CENPA	centromere protein A	1058	https://www.ncbi.nlm.nih.gov/gene/?term=1058
GCA	GCA	grancalcin	25801	https://www.ncbi.nlm.nih.gov/gene/?term=25801
PECR	PECR	peroxisomal trans-2-enoyl-CoA reductase	55825	https://www.ncbi.nlm.nih.gov/gene/?term=55825
EHBP1	EHBP1	EH domain binding protein 1	23301	https://www.ncbi.nlm.nih.gov/gene/?term=23301
SLC9A2	SLC9A2	solute carrier family 9 member A2	6549	https://www.ncbi.nlm.nih.gov/gene/?term=6549
BIRC6	BIRC6	baculoviral IAP repeat containing 6	57448	https://www.ncbi.nlm.nih.gov/gene/?term=57448
LCT	LCT	lactase	3938	https://www.ncbi.nlm.nih.gov/gene/?term=3938
C2orf42	C2orf42	chromosome 2 open reading frame 42	54980	https://www.ncbi.nlm.nih.gov/gene/?term=54980
QSOX1	QSOX1	quiescin sulfhydryl oxidase 1	5768	https://www.ncbi.nlm.nih.gov/gene/?term=5768
FBXO2	FBXO2	F-box protein 2	26232	https://www.ncbi.nlm.nih.gov/gene/?term=26232
CRYZ	CRYZ	crystallin zeta	1429	https://www.ncbi.nlm.nih.gov/gene/?term=1429
EXOC8	EXOC8	exocyst complex component 8	149371	https://www.ncbi.nlm.nih.gov/gene/?term=149371
ACADM	ACADM	acyl-CoA dehydrogenase medium chain	34	https://www.ncbi.nlm.nih.gov/gene/?term=34
PRDX6	PRDX6	peroxiredoxin 6	9588	https://www.ncbi.nlm.nih.gov/gene/?term=9588
ESYT2	ESYT2	extended synaptotagmin 2	57488	https://www.ncbi.nlm.nih.gov/gene/?term=57488
A4GNT	A4GNT	alpha-1,4-N-acetylglucosaminyltransferase	51146	https://www.ncbi.nlm.nih.gov/gene/?term=51146
IFT46	IFT46	intraflagellar transport 46	56912	https://www.ncbi.nlm.nih.gov/gene/?term=56912
TNNT2	TNNT2	troponin T2, cardiac type	7139	https://www.ncbi.nlm.nih.gov/gene/?term=7139
FASTKD2	FASTKD2	FAST kinase domains 2	22868	https://www.ncbi.nlm.nih.gov/gene/?term=22868

ATP10B	ATP10B	ATPase phospholipid transporting 10B (putative)	23120	https://www.ncbi.nlm.nih.gov/gene/?term=23120
SPACA1	SPACA1	sperm acrosome associated 1	81833	https://www.ncbi.nlm.nih.gov/gene/?term=81833
ZC2HC1B	ZC2HC1B	zinc finger C2HC-type containing 1B	153918	https://www.ncbi.nlm.nih.gov/gene/?term=153918
VAMP8	VAMP8	vesicle associated membrane protein 8	8673	https://www.ncbi.nlm.nih.gov/gene/?term=8673
RPN2	RPN2	ribophorin II	6185	https://www.ncbi.nlm.nih.gov/gene/?term=6185
SENP5	SENP5	SUMO specific peptidase 5	205564	https://www.ncbi.nlm.nih.gov/gene/?term=205564
HEATR1	HEATR1	HEAT repeat containing 1	55127	https://www.ncbi.nlm.nih.gov/gene/?term=55127
CNTRL	CNTRL	centriolin	11064	https://www.ncbi.nlm.nih.gov/gene/?term=11064
SLIRP	SLIRP	SRA stem-loop interacting RNA binding protein	81892	https://www.ncbi.nlm.nih.gov/gene/?term=81892
IFIT2	IFIT2	interferon induced protein with tetratricopeptide repeats 2	3433	https://www.ncbi.nlm.nih.gov/gene/?term=3433
PYROXD2	PYROXD2	pyridine nucleotide-disulphide oxidoreductase domain 2	84795	https://www.ncbi.nlm.nih.gov/gene/?term=84795
MXI1	MXI1	MAX interactor 1, dimerization protein	4601	https://www.ncbi.nlm.nih.gov/gene/?term=4601
PANK3	PANK3	pantothenate kinase 3	79646	https://www.ncbi.nlm.nih.gov/gene/?term=79646
PLEKHG1	PLEKHG1	pleckstrin homology and RhoGEF domain containing G1	57480	https://www.ncbi.nlm.nih.gov/gene/?term=57480
MTERF2	MTERF2	mitochondrial transcription termination factor 2	80298	https://www.ncbi.nlm.nih.gov/gene/?term=80298
TBX2	TBX2	T-box 2	6909	https://www.ncbi.nlm.nih.gov/gene/?term=6909
DPPA4	DPPA4	developmental pluripotency associated 4	55211	https://www.ncbi.nlm.nih.gov/gene/?term=55211
CRY2	CRY2	cryptochrome circadian regulator 2	1408	https://www.ncbi.nlm.nih.gov/gene/?term=1408
PLG	PLG	plasminogen	5340	https://www.ncbi.nlm.nih.gov/gene/?term=5340
RBBP6	RBBP6	RB binding protein 6, ubiquitin ligase	5930	https://www.ncbi.nlm.nih.gov/gene/?term=5930
NEUROG3	NEUROG3	neurogenin 3	50674	https://www.ncbi.nlm.nih.gov/gene/?term=50674
EBPL	EBPL	EBP like	84650	https://www.ncbi.nlm.nih.gov/gene/?term=84650

ITIH5	ITIH5	inter-alpha-trypsin inhibitor heavy chain family member 5	80760	https://www.ncbi.nlm.nih.gov/gene/?term=80760
MXD4	MXD4	MAX dimerization protein 4	10608	https://www.ncbi.nlm.nih.gov/gene/?term=10608
MOGAT1	MOGAT1	monoacylglycerol O-acyltransferase 1	116255	https://www.ncbi.nlm.nih.gov/gene/?term=116255
GCNT7	GCNT7	glucosaminyl (N-acetyl) transferase family member 7	140687	https://www.ncbi.nlm.nih.gov/gene/?term=140687
ZNFX1	ZNFX1	zinc finger NFX1-type containing 1	57169	https://www.ncbi.nlm.nih.gov/gene/?term=57169
VAMP7	VAMP7	vesicle associated membrane protein 7	6845	https://www.ncbi.nlm.nih.gov/gene/?term=6845
LYPD3	LYPD3	LY6/PLAUR domain containing 3	27076	https://www.ncbi.nlm.nih.gov/gene/?term=27076
CRISP2	CRISP2	cysteine rich secretory protein 2	7180	https://www.ncbi.nlm.nih.gov/gene/?term=7180
TRERF1	TRERF1	transcriptional regulating factor 1	55809	https://www.ncbi.nlm.nih.gov/gene/?term=55809
GTF2F1	GTF2F1	general transcription factor IIF subunit 1	2962	https://www.ncbi.nlm.nih.gov/gene/?term=2962
ALKBH7	ALKBH7	alkB homolog 7	84266	https://www.ncbi.nlm.nih.gov/gene/?term=84266
ATG4C	ATG4C	autophagy related 4C cysteine peptidase	84938	https://www.ncbi.nlm.nih.gov/gene/?term=84938
GPCPD1	GPCPD1	glycerophosphocholine phosphodiesterase 1	56261	https://www.ncbi.nlm.nih.gov/gene/?term=56261
BFSP1	BFSP1	beaded filament structural protein 1	631	https://www.ncbi.nlm.nih.gov/gene/?term=631
NCLN	NCLN	nicalin	56926	https://www.ncbi.nlm.nih.gov/gene/?term=56926
FRMD8	FRMD8	FERM domain containing 8	83786	https://www.ncbi.nlm.nih.gov/gene/?term=83786
PRR12	PRR12	proline rich 12	57479	https://www.ncbi.nlm.nih.gov/gene/?term=57479
AHDC1	AHDC1	AT-hook DNA binding motif containing 1	27245	https://www.ncbi.nlm.nih.gov/gene/?term=27245
KTN1	KTN1	kinectin 1	3895	https://www.ncbi.nlm.nih.gov/gene/?term=3895
RGS13	RGS13	regulator of G protein signaling 13	6003	https://www.ncbi.nlm.nih.gov/gene/?term=6003
TAS2R4	TAS2R4	taste 2 receptor member 4	50832	https://www.ncbi.nlm.nih.gov/gene/?term=50832
PLA2G5	PLA2G5	phospholipase A2 group V	5322	https://www.ncbi.nlm.nih.gov/gene/?term=5322
EPS15L1	EPS15L1	epidermal growth factor receptor pathway substrate 15 like 1	58513	https://www.ncbi.nlm.nih.gov/gene/?term=58513
KDR	KDR	kinase insert domain receptor	3791	https://www.ncbi.nlm.nih.gov/gene/?term=3791
MGAT3	MGAT3	mannosyl (beta-1,4-)-glycoprotein beta-1,4-N-	4248	https://www.ncbi.nlm.nih.gov/gene/?term=4248

		acetylglucosaminyltransferase		
LIF	LIF	LIF, interleukin 6 family cytokine	3976	https://www.ncbi.nlm.nih.gov/gene/?term=3976
CCDC32	CCDC32	coiled-coil domain containing 32	90416	https://www.ncbi.nlm.nih.gov/gene/?term=90416
DLL4	DLL4	delta like canonical Notch ligand 4	54567	https://www.ncbi.nlm.nih.gov/gene/?term=54567
SUMF2	SUMF2	sulfatase modifying factor 2	25870	https://www.ncbi.nlm.nih.gov/gene/?term=25870
CSRP3	CSRP3	cysteine and glycine rich protein 3	8048	https://www.ncbi.nlm.nih.gov/gene/?term=8048
DCTD	DCTD	dCMP deaminase	1635	https://www.ncbi.nlm.nih.gov/gene/?term=1635
PLD2	PLD2	phospholipase D2	5338	https://www.ncbi.nlm.nih.gov/gene/?term=5338
PHF20L1	PHF20L1	PHD finger protein 20 like 1	51105	https://www.ncbi.nlm.nih.gov/gene/?term=51105
LDLR	LDLR	low density lipoprotein receptor	3949	https://www.ncbi.nlm.nih.gov/gene/?term=3949
ACE2	ACE2	angiotensin I converting enzyme 2	59272	https://www.ncbi.nlm.nih.gov/gene/?term=59272
FAM98C	FAM98C	family with sequence similarity 98 member C	147965	https://www.ncbi.nlm.nih.gov/gene/?term=147965
ACSBG2	ACSBG2	acyl-CoA synthetase bubblegum family member 2	81616	https://www.ncbi.nlm.nih.gov/gene/?term=81616
MLLT1	MLLT1	MLLT1, super elongation complex subunit	4298	https://www.ncbi.nlm.nih.gov/gene/?term=4298
METTL26	METTL26	methyltransferase like 26	84326	https://www.ncbi.nlm.nih.gov/gene/?term=84326
GMFG	GMFG	glia maturation factor gamma	9535	https://www.ncbi.nlm.nih.gov/gene/?term=9535
SMPDL3B	SMPDL3B	sphingomyelin phosphodiesterase acid like 3B	27293	https://www.ncbi.nlm.nih.gov/gene/?term=27293
HIP1R	HIP1R	huntingtin interacting protein 1 related	9026	https://www.ncbi.nlm.nih.gov/gene/?term=9026
ANGPTL6	ANGPTL6	angiopoietin like 6	83854	https://www.ncbi.nlm.nih.gov/gene/?term=83854
DKC1	DKC1	dyskerin pseudouridine synthase 1	1736	https://www.ncbi.nlm.nih.gov/gene/?term=1736
ZNF341	ZNF341	zinc finger protein 341	84905	https://www.ncbi.nlm.nih.gov/gene/?term=84905
DIAPH1	DIAPH1	diaphanous related formin 1	1729	https://www.ncbi.nlm.nih.gov/gene/?term=1729
NPHP4	NPHP4	nephrocystin 4	261734	https://www.ncbi.nlm.nih.gov/gene/?term=261734
TNS4	TNS4	tensin 4	84951	https://www.ncbi.nlm.nih.gov/gene/?term=84951
SNRPA1	SNRPA1	small nuclear ribonucleoprotein polypeptide A'	6627	https://www.ncbi.nlm.nih.gov/gene/?term=6627

ACTR10	ACTR10	actin related protein 10	55860	https://www.ncbi.nlm.nih.gov/gene/?term=55860
TRIM21	TRIM21	tripartite motif containing 21	6737	https://www.ncbi.nlm.nih.gov/gene/?term=6737
IMMT	IMMT	inner membrane mitochondrial protein	10989	https://www.ncbi.nlm.nih.gov/gene/?term=10989
RAMP1	RAMP1	receptor activity modifying protein 1	10267	https://www.ncbi.nlm.nih.gov/gene/?term=10267
PTPRE	PTPRE	protein tyrosine phosphatase, receptor type E	5791	https://www.ncbi.nlm.nih.gov/gene/?term=5791
LANCL2	LANCL2	LanC like 2	55915	https://www.ncbi.nlm.nih.gov/gene/?term=55915
KDM6B	KDM6B	lysine demethylase 6B	23135	https://www.ncbi.nlm.nih.gov/gene/?term=23135
GUCY2D	GUCY2D	guanylate cyclase 2D, retinal	3000	https://www.ncbi.nlm.nih.gov/gene/?term=3000
RIN2	RIN2	Ras and Rab interactor 2	54453	https://www.ncbi.nlm.nih.gov/gene/?term=54453
KANK4	KANK4	KN motif and ankyrin repeat domains 4	163782	https://www.ncbi.nlm.nih.gov/gene/?term=163782
LGR6	LGR6	leucine rich repeat containing G protein-coupled receptor 6	59352	https://www.ncbi.nlm.nih.gov/gene/?term=59352
TMCC2	TMCC2	transmembrane and coiled-coil domain family 2	9911	https://www.ncbi.nlm.nih.gov/gene/?term=9911
GPALPP1	GPALPP1	GPALPP motifs containing 1	55425	https://www.ncbi.nlm.nih.gov/gene/?term=55425
SLC39A11	SLC39A11	solute carrier family 39 member 11	201266	https://www.ncbi.nlm.nih.gov/gene/?term=201266
ZNF414	ZNF414	zinc finger protein 414	84330	https://www.ncbi.nlm.nih.gov/gene/?term=84330
RTN3	RTN3	reticulon 3	10313	https://www.ncbi.nlm.nih.gov/gene/?term=10313
ATP13A3	ATP13A3	ATPase 13A3	79572	https://www.ncbi.nlm.nih.gov/gene/?term=79572
DYDC2	DYDC2	DPY30 domain containing 2	84332	https://www.ncbi.nlm.nih.gov/gene/?term=84332
MEN1	MEN1	menin 1	4221	https://www.ncbi.nlm.nih.gov/gene/?term=4221
CHL1	CHL1	cell adhesion molecule L1 like	10752	https://www.ncbi.nlm.nih.gov/gene/?term=10752
PRPF38B	PRPF38B	pre-mRNA processing factor 38B	55119	https://www.ncbi.nlm.nih.gov/gene/?term=55119
RAX	RAX	retina and anterior neural fold homeobox	30062	https://www.ncbi.nlm.nih.gov/gene/?term=30062
DSC2	DSC2	desmocollin 2	1824	https://www.ncbi.nlm.nih.gov/gene/?term=1824
DSG1	DSG1	desmoglein 1	1828	https://www.ncbi.nlm.nih.gov/gene/?term=1828
DSC1	DSC1	desmocollin 1	1823	https://www.ncbi.nlm.nih.gov/gene/?term=1823
UBAC2	UBAC2	UBA domain containing 2	337867	https://www.ncbi.nlm.nih.gov/gene/?term=337867

CARS2	CARS2	cysteinyl-tRNA synthetase 2, mitochondrial	79587	https://www.ncbi.nlm.nih.gov/gene/?term=79587
HNF1A	HNF1A	HNF1 homeobox A	6927	https://www.ncbi.nlm.nih.gov/gene/?term=6927
KRT85	KRT85	keratin 85	3891	https://www.ncbi.nlm.nih.gov/gene/?term=3891
NHSL1	NHSL1	NHS like 1	57224	https://www.ncbi.nlm.nih.gov/gene/?term=57224
KIAA0513	KIAA0513	KIAA0513	9764	https://www.ncbi.nlm.nih.gov/gene/?term=9764
KCNK1	KCNK1	potassium two pore domain channel subfamily K member 1	3775	https://www.ncbi.nlm.nih.gov/gene/?term=3775
TAF5L	TAF5L	TATA-box binding protein associated factor 5 like	27097	https://www.ncbi.nlm.nih.gov/gene/?term=27097
RNASEL	RNASEL	ribonuclease L	6041	https://www.ncbi.nlm.nih.gov/gene/?term=6041
DNAJB2	DNAJB2	DnaJ heat shock protein family (Hsp40) member B2	3300	https://www.ncbi.nlm.nih.gov/gene/?term=3300
COX5B	COX5B	cytochrome c oxidase subunit 5B	1329	https://www.ncbi.nlm.nih.gov/gene/?term=1329
EDNRB	EDNRB	endothelin receptor type B	1910	https://www.ncbi.nlm.nih.gov/gene/?term=1910
GPNMB	GPNMB	glycoprotein nmb	10457	https://www.ncbi.nlm.nih.gov/gene/?term=10457
TBRG4	TBRG4	transforming growth factor beta regulator 4	9238	https://www.ncbi.nlm.nih.gov/gene/?term=9238
ZFHX2	ZFHX2	zinc finger homeobox 2	85446	https://www.ncbi.nlm.nih.gov/gene/?term=85446
KCTD3	KCTD3	potassium channel tetramerization domain containing 3	51133	https://www.ncbi.nlm.nih.gov/gene/?term=51133
TXN	TXN	thioredoxin	7295	https://www.ncbi.nlm.nih.gov/gene/?term=7295
DAB2IP	DAB2IP	DAB2 interacting protein	153090	https://www.ncbi.nlm.nih.gov/gene/?term=153090
ZNF189	ZNF189	zinc finger protein 189	7743	https://www.ncbi.nlm.nih.gov/gene/?term=7743
HEMGN	HEMGN	hemogen	55363	https://www.ncbi.nlm.nih.gov/gene/?term=55363
FAM8A1	FAM8A1	family with sequence similarity 8 member A1	51439	https://www.ncbi.nlm.nih.gov/gene/?term=51439
GGH	GGH	gamma-glutamyl hydrolase	8836	https://www.ncbi.nlm.nih.gov/gene/?term=8836
NEK1	NEK1	NIMA related kinase 1	4750	https://www.ncbi.nlm.nih.gov/gene/?term=4750
SORL1	SORL1	sortilin related receptor 1	6653	https://www.ncbi.nlm.nih.gov/gene/?term=6653
GIPC2	GIPC2	GIPC PDZ domain containing family member 2	54810	https://www.ncbi.nlm.nih.gov/gene/?term=54810

CGREF1	CGREF1	cell growth regulator with EF-hand domain 1	10669	https://www.ncbi.nlm.nih.gov/gene/?term=10669
ATRAID	ATRAID	all-trans retinoic acid induced differentiation factor	51374	https://www.ncbi.nlm.nih.gov/gene/?term=51374
TRIM54	TRIM54	tripartite motif containing 54	57159	https://www.ncbi.nlm.nih.gov/gene/?term=57159
ENTPD1	ENTPD1	ectonucleoside triphosphate diphosphohydrolase 1	953	https://www.ncbi.nlm.nih.gov/gene/?term=953
ANXA7	ANXA7	annexin A7	310	https://www.ncbi.nlm.nih.gov/gene/?term=310
FAM149B1	FAM149B1	family with sequence similarity 149 member B1	317662	https://www.ncbi.nlm.nih.gov/gene/?term=317662
HECW2	HECW2	HECT, C2 and WW domain containing E3 ubiquitin protein ligase 2	57520	https://www.ncbi.nlm.nih.gov/gene/?term=57520
COX17	COX17	cytochrome c oxidase copper chaperone COX17	10063	https://www.ncbi.nlm.nih.gov/gene/?term=10063
MNS1	MNS1	meiosis specific nuclear structural 1	55329	https://www.ncbi.nlm.nih.gov/gene/?term=55329
FGF5	FGF5	fibroblast growth factor 5	2250	https://www.ncbi.nlm.nih.gov/gene/?term=2250
PPA2	PPA2	pyrophosphatase (inorganic) 2	27068	https://www.ncbi.nlm.nih.gov/gene/?term=27068
PARVG	PARVG	parvin gamma	64098	https://www.ncbi.nlm.nih.gov/gene/?term=64098
SCAF11	SCAF11	SR-related CTD associated factor 11	9169	https://www.ncbi.nlm.nih.gov/gene/?term=9169
SLC15A4	SLC15A4	solute carrier family 15 member 4	121260	https://www.ncbi.nlm.nih.gov/gene/?term=121260
TARBP2	TARBP2	TARBP2, RISC loading complex RNA binding subunit	6895	https://www.ncbi.nlm.nih.gov/gene/?term=6895
NIPA2	NIPA2	NIPA magnesium transporter 2	81614	https://www.ncbi.nlm.nih.gov/gene/?term=81614
BAHD1	BAHD1	bromo adjacent homology domain containing 1	22893	https://www.ncbi.nlm.nih.gov/gene/?term=22893
PIF1	PIF1	PIF1 5'-to-3' DNA helicase	80119	https://www.ncbi.nlm.nih.gov/gene/?term=80119
LINS1	LINS1	lines homolog 1	55180	https://www.ncbi.nlm.nih.gov/gene/?term=55180
LMAN1L	LMAN1L	lectin, mannose binding 1 like	79748	https://www.ncbi.nlm.nih.gov/gene/?term=79748
SLC5A2	SLC5A2	solute carrier family 5 member 2	6524	https://www.ncbi.nlm.nih.gov/gene/?term=6524
DEF8	DEF8	differentially expressed in FDCP 8 homolog	54849	https://www.ncbi.nlm.nih.gov/gene/?term=54849
PCTP	PCTP	phosphatidylcholine transfer protein	58488	https://www.ncbi.nlm.nih.gov/gene/?term=58488

SLC39A6	SLC39A6	solute carrier family 39 member 6	25800	https://www.ncbi.nlm.nih.gov/gene/?term=25800
ZMYND15	ZMYND15	zinc finger MYND-type containing 15	84225	https://www.ncbi.nlm.nih.gov/gene/?term=84225
NARF	NARF	nuclear prelamin A recognition factor	26502	https://www.ncbi.nlm.nih.gov/gene/?term=26502
ZNF750	ZNF750	zinc finger protein 750	79755	https://www.ncbi.nlm.nih.gov/gene/?term=79755
NFIC	NFIC	nuclear factor I C	4782	https://www.ncbi.nlm.nih.gov/gene/?term=4782
PLPP2	PLPP2	phospholipid phosphatase 2	8612	https://www.ncbi.nlm.nih.gov/gene/?term=8612
FEM1A	FEM1A	fem-1 homolog A	55527	https://www.ncbi.nlm.nih.gov/gene/?term=55527
VAV1	VAV1	vav guanine nucleotide exchange factor 1	7409	https://www.ncbi.nlm.nih.gov/gene/?term=7409
DOP1B	DOP1B	DOP1 leucine zipper like protein B	9980	https://www.ncbi.nlm.nih.gov/gene/?term=9980
ILDR2	ILDR2	immunoglobulin like domain containing receptor 2	387597	https://www.ncbi.nlm.nih.gov/gene/?term=387597
NUF2	NUF2	NDC80 kinetochore complex component NUF2	83540	https://www.ncbi.nlm.nih.gov/gene/?term=83540
ISG20L2	ISG20L2	interferon stimulated exonuclease gene 20 like 2	81875	https://www.ncbi.nlm.nih.gov/gene/?term=81875
RORC	RORC	RAR related orphan receptor C	6097	https://www.ncbi.nlm.nih.gov/gene/?term=6097
SNX27	SNX27	sorting nexin family member 27	81609	https://www.ncbi.nlm.nih.gov/gene/?term=81609
RFX5	RFX5	regulatory factor X5	5993	https://www.ncbi.nlm.nih.gov/gene/?term=5993
POGZ	POGZ	pogo transposable element derived with ZNF domain	23126	https://www.ncbi.nlm.nih.gov/gene/?term=23126
DTL	DTL	denticleless E3 ubiquitin protein ligase homolog	51514	https://www.ncbi.nlm.nih.gov/gene/?term=51514
SOX13	SOX13	SRY-box 13	9580	https://www.ncbi.nlm.nih.gov/gene/?term=9580
PLEKHA6	PLEKHA6	pleckstrin homology domain containing A6	22874	https://www.ncbi.nlm.nih.gov/gene/?term=22874
ATP6V1C2	ATP6V1C2	ATPase H+ transporting V1 subunit C2	245973	https://www.ncbi.nlm.nih.gov/gene/?term=245973
ASXL2	ASXL2	ASXL transcriptional regulator 2	55252	https://www.ncbi.nlm.nih.gov/gene/?term=55252
ABHD1	ABHD1	abhydrolase domain containing 1	84696	https://www.ncbi.nlm.nih.gov/gene/?term=84696
NXPH2	NXPH2	neurexophilin 2	11249	https://www.ncbi.nlm.nih.gov/gene/?term=11249
SPAG16	SPAG16	sperm associated antigen 16	79582	https://www.ncbi.nlm.nih.gov/gene/?term=79582

NR1I2	NR1I2	nuclear receptor subfamily 1 group I member 2	8856	https://www.ncbi.nlm.nih.gov/gene/?term=8856
SRPRB	SRPRB	SRP receptor subunit beta	58477	https://www.ncbi.nlm.nih.gov/gene/?term=58477
UCN2	UCN2	urocortin 2	90226	https://www.ncbi.nlm.nih.gov/gene/?term=90226
AHSG	AHSG	alpha 2-HS glycoprotein	197	https://www.ncbi.nlm.nih.gov/gene/?term=197
PLAC8	PLAC8	placenta specific 8	51316	https://www.ncbi.nlm.nih.gov/gene/?term=51316
NAF1	NAF1	nuclear assembly factor 1 ribonucleoprotein	92345	https://www.ncbi.nlm.nih.gov/gene/?term=92345
ROPN1L	ROPN1L	rhophilin associated tail protein 1 like	83853	https://www.ncbi.nlm.nih.gov/gene/?term=83853
SSBP2	SSBP2	single stranded DNA binding protein 2	23635	https://www.ncbi.nlm.nih.gov/gene/?term=23635
SLC25A48	SLC25A48	solute carrier family 25 member 48	153328	https://www.ncbi.nlm.nih.gov/gene/?term=153328
TNFRSF21	TNFRSF21	TNF receptor superfamily member 21	27242	https://www.ncbi.nlm.nih.gov/gene/?term=27242
DAAM2	DAAM2	dishevelled associated activator of morphogenesis 2	23500	https://www.ncbi.nlm.nih.gov/gene/?term=23500
PRSS35	PRSS35	serine protease 35	167681	https://www.ncbi.nlm.nih.gov/gene/?term=167681
AIG1	AIG1	androgen induced 1	51390	https://www.ncbi.nlm.nih.gov/gene/?term=51390
TMEM181	TMEM181	transmembrane protein 181	57583	https://www.ncbi.nlm.nih.gov/gene/?term=57583
NOM1	NOM1	nucleolar protein with MIF4G domain 1	64434	https://www.ncbi.nlm.nih.gov/gene/?term=64434
ASB10	ASB10	ankyrin repeat and SOCS box containing 10	136371	https://www.ncbi.nlm.nih.gov/gene/?term=136371
SH3KBP1	SH3KBP1	SH3 domain containing kinase binding protein 1	30011	https://www.ncbi.nlm.nih.gov/gene/?term=30011
ZMYM3	ZMYM3	zinc finger MYM-type containing 3	9203	https://www.ncbi.nlm.nih.gov/gene/?term=9203
AWAT2	AWAT2	acyl-CoA wax alcohol acyltransferase 2	158835	https://www.ncbi.nlm.nih.gov/gene/?term=158835
ST18	ST18	ST18, C2H2C-type zinc finger	9705	https://www.ncbi.nlm.nih.gov/gene/?term=9705
TACC1	TACC1	transforming acidic coiled-coil containing protein 1	6867	https://www.ncbi.nlm.nih.gov/gene/?term=6867
ST8SIA6	ST8SIA6	ST8 alpha-N-acetyl-neuraminide alpha-2,8-sialyltransferase 6	338596	https://www.ncbi.nlm.nih.gov/gene/?term=338596
HTR7	HTR7	5-hydroxytryptamine receptor 7	3363	https://www.ncbi.nlm.nih.gov/gene/?term=3363

CYP17A1	CYP17A1	cytochrome P450 family 17 subfamily A member 1	1586	https://www.ncbi.nlm.nih.gov/gene/?term=1586
TUT1	TUT1	terminal uridylyl transferase 1, U6 snRNA-specific	64852	https://www.ncbi.nlm.nih.gov/gene/?term=64852
HSD17B12	HSD17B12	hydroxysteroid 17-beta dehydrogenase 12	51144	https://www.ncbi.nlm.nih.gov/gene/?term=51144
DGKZ	DGKZ	diacylglycerol kinase zeta	8525	https://www.ncbi.nlm.nih.gov/gene/?term=8525
GLYAT	GLYAT	glycine-N-acyltransferase	10249	https://www.ncbi.nlm.nih.gov/gene/?term=10249
SERPING1	SERPING1	serpin family G member 1	710	https://www.ncbi.nlm.nih.gov/gene/?term=710
ARFGAP2	ARFGAP2	ADP ribosylation factor GTPase activating protein 2	84364	https://www.ncbi.nlm.nih.gov/gene/?term=84364
CCDC82	CCDC82	coiled-coil domain containing 82	79780	https://www.ncbi.nlm.nih.gov/gene/?term=79780
CHEK1	CHEK1	checkpoint kinase 1	1111	https://www.ncbi.nlm.nih.gov/gene/?term=1111
COMMD7	COMMD7	COMM domain containing 7	149951	https://www.ncbi.nlm.nih.gov/gene/?term=149951
KIAA1755	KIAA1755	KIAA1755	85449	https://www.ncbi.nlm.nih.gov/gene/?term=85449
CDH22	CDH22	cadherin 22	64405	https://www.ncbi.nlm.nih.gov/gene/?term=64405
TRPT1	TRPT1	tRNA phosphotransferase 1	83707	https://www.ncbi.nlm.nih.gov/gene/?term=83707
DLAT	DLAT	dihydrolipoamide S-acetyltransferase	1737	https://www.ncbi.nlm.nih.gov/gene/?term=1737
PTS	PTS	6-pyruvoyltetrahydropterin synthase	5805	https://www.ncbi.nlm.nih.gov/gene/?term=5805
SCLT1	SCLT1	sodium channel and clathrin linker 1	132320	https://www.ncbi.nlm.nih.gov/gene/?term=132320
FAM160B1	FAM160B1	family with sequence similarity 160 member B1	57700	https://www.ncbi.nlm.nih.gov/gene/?term=57700
EDNRA	EDNRA	endothelin receptor type A	1909	https://www.ncbi.nlm.nih.gov/gene/?term=1909
BEND6	BEND6	BEN domain containing 6	221336	https://www.ncbi.nlm.nih.gov/gene/?term=221336
SETBP1	SETBP1	SET binding protein 1	26040	https://www.ncbi.nlm.nih.gov/gene/?term=26040
BMP3	BMP3	bone morphogenetic protein 3	651	https://www.ncbi.nlm.nih.gov/gene/?term=651
CPB1	CPB1	carboxypeptidase B1	1360	https://www.ncbi.nlm.nih.gov/gene/?term=1360
TMEM87B	TMEM87B	transmembrane protein 87B	84910	https://www.ncbi.nlm.nih.gov/gene/?term=84910
ZDHHC7	ZDHHC7	zinc finger DHHC-type containing 7	55625	https://www.ncbi.nlm.nih.gov/gene/?term=55625
TBCEL	TBCEL	tubulin folding cofactor E like	219899	https://www.ncbi.nlm.nih.gov/gene/?term=219899

CC2D1B	CC2D1B	coiled-coil and C2 domain containing 1B	200014	https://www.ncbi.nlm.nih.gov/gene/?term=200014
DIPK1A	DIPK1A	divergent protein kinase domain 1A	388650	https://www.ncbi.nlm.nih.gov/gene/?term=388650
AK9	AK9	adenylate kinase 9	221264	https://www.ncbi.nlm.nih.gov/gene/?term=221264
OXA1L	OXA1L	OXA1L, mitochondrial inner membrane protein	5018	https://www.ncbi.nlm.nih.gov/gene/?term=5018
LARP1	LARP1	La ribonucleoprotein domain family member 1	23367	https://www.ncbi.nlm.nih.gov/gene/?term=23367
CYLC2	CYLC2	cylicin 2	1539	https://www.ncbi.nlm.nih.gov/gene/?term=1539
ART3	ART3	ADP-ribosyltransferase 3	419	https://www.ncbi.nlm.nih.gov/gene/?term=419
SCAF4	SCAF4	SR-related CTD associated factor 4	57466	https://www.ncbi.nlm.nih.gov/gene/?term=57466
SUPV3L1	SUPV3L1	Suv3 like RNA helicase	6832	https://www.ncbi.nlm.nih.gov/gene/?term=6832
PDE6D	PDE6D	phosphodiesterase 6D	5147	https://www.ncbi.nlm.nih.gov/gene/?term=5147
FCHO2	FCHO2	FCH domain only 2	115548	https://www.ncbi.nlm.nih.gov/gene/?term=115548
C8A	C8A	complement C8 alpha chain	731	https://www.ncbi.nlm.nih.gov/gene/?term=731
SYN2	SYN2	synapsin II	6854	https://www.ncbi.nlm.nih.gov/gene/?term=6854
PWWP3B	PWWP3B	PWWP domain containing 3B	139221	https://www.ncbi.nlm.nih.gov/gene/?term=139221
ETS2	ETS2	NA	2114	https://www.ncbi.nlm.nih.gov/gene/?term=2114
MX1	MX1	MX dynamin like GTPase 1	4599	https://www.ncbi.nlm.nih.gov/gene/?term=4599
UBN2	UBN2	ubinuclein 2	254048	https://www.ncbi.nlm.nih.gov/gene/?term=254048
SKI	SKI	SKI proto-oncogene	6497	https://www.ncbi.nlm.nih.gov/gene/?term=6497
WIPI2	WIPI2	WD repeat domain, phosphoinositide interacting 2	26100	https://www.ncbi.nlm.nih.gov/gene/?term=26100
KRTCAP3	KRTCAP3	keratinocyte associated protein 3	200634	https://www.ncbi.nlm.nih.gov/gene/?term=200634
PAFAH2	PAFAH2	platelet activating factor acetylhydrolase 2	5051	https://www.ncbi.nlm.nih.gov/gene/?term=5051
ESYT3	ESYT3	extended synaptotagmin 3	83850	https://www.ncbi.nlm.nih.gov/gene/?term=83850
TENT5B	TENT5B	terminal nucleotidyltransferase 5B	115572	https://www.ncbi.nlm.nih.gov/gene/?term=115572
PEX2	PEX2	peroxisomal biogenesis factor 2	5828	https://www.ncbi.nlm.nih.gov/gene/?term=5828

LARGE2	LARGE2	LARGE xylosyl- and glucuronyltransferase 2	120071	https://www.ncbi.nlm.nih.gov/gene/?term=120071
MYL1	MYL1	myosin light chain 1	4632	https://www.ncbi.nlm.nih.gov/gene/?term=4632
CHRM1	CHRM1	cholinergic receptor muscarinic 1	1128	https://www.ncbi.nlm.nih.gov/gene/?term=1128
TCTN2	TCTN2	tectonic family member 2	79867	https://www.ncbi.nlm.nih.gov/gene/?term=79867
ATOH8	ATOH8	atonal bHLH transcription factor 8	84913	https://www.ncbi.nlm.nih.gov/gene/?term=84913
COL4A3	COL4A3	collagen type IV alpha 3 chain	1285	https://www.ncbi.nlm.nih.gov/gene/?term=1285
TBC1D10B	TBC1D10B	TBC1 domain family member 10B	26000	https://www.ncbi.nlm.nih.gov/gene/?term=26000
ZRSR2	ZRSR2	zinc finger CCCH-type, RNA binding motif and serine/arginine rich 2	8233	https://www.ncbi.nlm.nih.gov/gene/?term=8233
PTAFR	PTAFR	platelet activating factor receptor	5724	https://www.ncbi.nlm.nih.gov/gene/?term=5724
NPR1	NPR1	natriuretic peptide receptor 1	4881	https://www.ncbi.nlm.nih.gov/gene/?term=4881
MUC15	MUC15	mucin 15, cell surface associated	143662	https://www.ncbi.nlm.nih.gov/gene/?term=143662
HIC2	HIC2	HIC ZBTB transcriptional repressor 2	23119	https://www.ncbi.nlm.nih.gov/gene/?term=23119
ITGAM	ITGAM	integrin subunit alpha M	3684	https://www.ncbi.nlm.nih.gov/gene/?term=3684
KCNAB3	KCNAB3	potassium voltage-gated channel subfamily A regulatory beta subunit 3	9196	https://www.ncbi.nlm.nih.gov/gene/?term=9196
ZNF282	ZNF282	zinc finger protein 282	8427	https://www.ncbi.nlm.nih.gov/gene/?term=8427
GLB1	GLB1	galactosidase beta 1	2720	https://www.ncbi.nlm.nih.gov/gene/?term=2720
EMB	EMB	embigin	133418	https://www.ncbi.nlm.nih.gov/gene/?term=133418
OR9K2	OR9K2	olfactory receptor family 9 subfamily K member 2	441639	https://www.ncbi.nlm.nih.gov/gene/?term=441639
RNF139	RNF139	ring finger protein 139	11236	https://www.ncbi.nlm.nih.gov/gene/?term=11236
MBD3L1	MBD3L1	methyl-CpG binding domain protein 3 like 1	85509	https://www.ncbi.nlm.nih.gov/gene/?term=85509
ALK	ALK	ALK receptor tyrosine kinase	238	https://www.ncbi.nlm.nih.gov/gene/?term=238
CLDN20	CLDN20	claudin 20	49861	https://www.ncbi.nlm.nih.gov/gene/?term=49861
SCAND1	SCAND1	SCAN domain containing 1	51282	https://www.ncbi.nlm.nih.gov/gene/?term=51282
ZNF318	ZNF318	zinc finger protein 318	24149	https://www.ncbi.nlm.nih.gov/gene/?term=24149

MAP6	MAP6	microtubule associated protein 6	4135	https://www.ncbi.nlm.nih.gov/gene/?term=4135
FGG	FGG	fibrinogen gamma chain	2266	https://www.ncbi.nlm.nih.gov/gene/?term=2266
CXXC5	CXXC5	CXXC finger protein 5	51523	https://www.ncbi.nlm.nih.gov/gene/?term=51523
SYCE1	SYCE1	synaptonemal complex central element protein 1	93426	https://www.ncbi.nlm.nih.gov/gene/?term=93426
IFNB1	IFNB1	interferon beta 1	3456	https://www.ncbi.nlm.nih.gov/gene/?term=3456
DRC3	DRC3	dynein regulatory complex subunit 3	83450	https://www.ncbi.nlm.nih.gov/gene/?term=83450
LAMB2	LAMB2	laminin subunit beta 2	3913	https://www.ncbi.nlm.nih.gov/gene/?term=3913
HINFP	HINFP	histone H4 transcription factor	25988	https://www.ncbi.nlm.nih.gov/gene/?term=25988
CORO1B	CORO1B	coronin 1B	57175	https://www.ncbi.nlm.nih.gov/gene/?term=57175
MUS81	MUS81	MUS81 structure-specific endonuclease subunit	80198	https://www.ncbi.nlm.nih.gov/gene/?term=80198
METAP1D	METAP1D	methionyl aminopeptidase type 1D, mitochondrial	254042	https://www.ncbi.nlm.nih.gov/gene/?term=254042
EXO1	EXO1	exonuclease 1	9156	https://www.ncbi.nlm.nih.gov/gene/?term=9156
GTF2IRD2B	GTF2IRD2B	GTF2I repeat domain containing 2B	389524	https://www.ncbi.nlm.nih.gov/gene/?term=389524
STARD6	STARD6	StAR related lipid transfer domain containing 6	147323	https://www.ncbi.nlm.nih.gov/gene/?term=147323
DES	DES	desmin	1674	https://www.ncbi.nlm.nih.gov/gene/?term=1674
OR52W1	OR52W1	olfactory receptor family 52 subfamily W member 1	120787	https://www.ncbi.nlm.nih.gov/gene/?term=120787
HSF5	HSF5	heat shock transcription factor 5	124535	https://www.ncbi.nlm.nih.gov/gene/?term=124535
GJC3	GJC3	gap junction protein gamma 3	349149	https://www.ncbi.nlm.nih.gov/gene/?term=349149
CLEC14A	CLEC14A	C-type lectin domain containing 14A	161198	https://www.ncbi.nlm.nih.gov/gene/?term=161198
SIX5	SIX5	SIX homeobox 5	147912	https://www.ncbi.nlm.nih.gov/gene/?term=147912
CAVIN1	CAVIN1	caveolae associated protein 1	284119	https://www.ncbi.nlm.nih.gov/gene/?term=284119
ACAD9	ACAD9	acyl-CoA dehydrogenase family member 9	28976	https://www.ncbi.nlm.nih.gov/gene/?term=28976
PNPLA2	PNPLA2	patatin like phospholipase domain containing 2	57104	https://www.ncbi.nlm.nih.gov/gene/?term=57104
BOLA1	BOLA1	bolA family member 1	51027	https://www.ncbi.nlm.nih.gov/gene/?term=51027

MSC	MSC	musculin	9242	https://www.ncbi.nlm.nih.gov/gene/?term=9242
PFAS	PFAS	phosphoribosylformylglycinamidine synthase	5198	https://www.ncbi.nlm.nih.gov/gene/?term=5198
SNX18	SNX18	sorting nexin 18	112574	https://www.ncbi.nlm.nih.gov/gene/?term=112574
C3orf38	C3orf38	chromosome 3 open reading frame 38	285237	https://www.ncbi.nlm.nih.gov/gene/?term=285237
CYC1	CYC1	cytochrome c1	1537	https://www.ncbi.nlm.nih.gov/gene/?term=1537
GATA2	GATA2	GATA binding protein 2	2624	https://www.ncbi.nlm.nih.gov/gene/?term=2624
TMEM31	TMEM31	transmembrane protein 31	203562	https://www.ncbi.nlm.nih.gov/gene/?term=203562
CDH5	CDH5	cadherin 5	1003	https://www.ncbi.nlm.nih.gov/gene/?term=1003
MYADM	MYADM	myeloid associated differentiation marker	91663	https://www.ncbi.nlm.nih.gov/gene/?term=91663
RNF227	RNF227	ring finger protein 227	284023	https://www.ncbi.nlm.nih.gov/gene/?term=284023
FIZ1	FIZ1	FLT3 interacting zinc finger 1	84922	https://www.ncbi.nlm.nih.gov/gene/?term=84922
EXOC3	EXOC3	exocyst complex component 3	11336	https://www.ncbi.nlm.nih.gov/gene/?term=11336
RCC1	RCC1	regulator of chromosome condensation 1	1104	https://www.ncbi.nlm.nih.gov/gene/?term=1104
GSX2	GSX2	GS homeobox 2	170825	https://www.ncbi.nlm.nih.gov/gene/?term=170825
CLRN3	CLRN3	clarin 3	119467	https://www.ncbi.nlm.nih.gov/gene/?term=119467
PPA1	PPA1	pyrophosphatase (inorganic) 1	5464	https://www.ncbi.nlm.nih.gov/gene/?term=5464
FAM83H	FAM83H	family with sequence similarity 83 member H	286077	https://www.ncbi.nlm.nih.gov/gene/?term=286077
DIPK2A	DIPK2A	divergent protein kinase domain 2A	205428	https://www.ncbi.nlm.nih.gov/gene/?term=205428
KCNIP1	KCNIP1	potassium voltage-gated channel interacting protein 1	30820	https://www.ncbi.nlm.nih.gov/gene/?term=30820
C1S	C1S	complement C1s	716	https://www.ncbi.nlm.nih.gov/gene/?term=716
BGN	BGN	biglycan	633	https://www.ncbi.nlm.nih.gov/gene/?term=633
C1orf116	C1orf116	chromosome 1 open reading frame 116	79098	https://www.ncbi.nlm.nih.gov/gene/?term=79098
GDPGP1	GDPGP1	GDP-D-glucose phosphorylase 1	390637	https://www.ncbi.nlm.nih.gov/gene/?term=390637
COA5	COA5	cytochrome c oxidase assembly factor 5	493753	https://www.ncbi.nlm.nih.gov/gene/?term=493753
CCR3	CCR3	C-C motif chemokine receptor 3	1232	https://www.ncbi.nlm.nih.gov/gene/?term=1232
UPP1	UPP1	uridine phosphorylase 1	7378	https://www.ncbi.nlm.nih.gov/gene/?term=7378

ACP7	ACP7	acid phosphatase 7, tartrate resistant (putative)	390928	https://www.ncbi.nlm.nih.gov/gene/?term=390928
B3GALT5	B3GALT5	beta-1,3-galactosyltransferase 5	10317	https://www.ncbi.nlm.nih.gov/gene/?term=10317
SLC35F3	SLC35F3	solute carrier family 35 member F3	148641	https://www.ncbi.nlm.nih.gov/gene/?term=148641
OLFML1	OLFML1	olfactomedin like 1	283298	https://www.ncbi.nlm.nih.gov/gene/?term=283298
ADAP2	ADAP2	ArfGAP with dual PH domains 2	55803	https://www.ncbi.nlm.nih.gov/gene/?term=55803
LRTOMT	LRTOMT	leucine rich transmembrane and O-methyltransferase domain containing	220074	https://www.ncbi.nlm.nih.gov/gene/?term=220074
IRAK1	IRAK1	interleukin 1 receptor associated kinase 1	3654	https://www.ncbi.nlm.nih.gov/gene/?term=3654
CMSS1	CMSS1	cms1 ribosomal small subunit homolog	84319	https://www.ncbi.nlm.nih.gov/gene/?term=84319
SRPK3	SRPK3	SRSF protein kinase 3	26576	https://www.ncbi.nlm.nih.gov/gene/?term=26576
POU3F2	POU3F2	POU class 3 homeobox 2	5454	https://www.ncbi.nlm.nih.gov/gene/?term=5454
NUTM1	NUTM1	NUT midline carcinoma family member 1	256646	https://www.ncbi.nlm.nih.gov/gene/?term=256646
RNLS	RNLS	renalase, FAD dependent amine oxidase	55328	https://www.ncbi.nlm.nih.gov/gene/?term=55328
FAM43A	FAM43A	family with sequence similarity 43 member A	131583	https://www.ncbi.nlm.nih.gov/gene/?term=131583
LAMP1	LAMP1	lysosomal associated membrane protein 1	3916	https://www.ncbi.nlm.nih.gov/gene/?term=3916
SETD4	SETD4	SET domain containing 4	54093	https://www.ncbi.nlm.nih.gov/gene/?term=54093
SAPCD2	SAPCD2	suppressor APC domain containing 2	89958	https://www.ncbi.nlm.nih.gov/gene/?term=89958
KLK12	KLK12	kallikrein related peptidase 12	43849	https://www.ncbi.nlm.nih.gov/gene/?term=43849
SMYD4	SMYD4	SET and MYND domain containing 4	114826	https://www.ncbi.nlm.nih.gov/gene/?term=114826
UBE2H	UBE2H	ubiquitin conjugating enzyme E2 H	7328	https://www.ncbi.nlm.nih.gov/gene/?term=7328
KATNA1	KATNA1	katanin catalytic subunit A1	11104	https://www.ncbi.nlm.nih.gov/gene/?term=11104
RPS19BP1	RPS19BP1	ribosomal protein S19 binding protein 1	91582	https://www.ncbi.nlm.nih.gov/gene/?term=91582
C5orf52	C5orf52	chromosome 5 open reading frame 52	100190949	https://www.ncbi.nlm.nih.gov/gene/?term=100190949
AMTN	AMTN	amelotin	401138	https://www.ncbi.nlm.nih.gov/gene/?term=401138
SLC18A3	SLC18A3	solute carrier family 18 member A3	6572	https://www.ncbi.nlm.nih.gov/gene/?term=6572

PEAR1	PEAR1	platelet endothelial aggregation receptor 1	375033	https://www.ncbi.nlm.nih.gov/gene/?term=375033
ZFP69	ZFP69	ZFP69 zinc finger protein	339559	https://www.ncbi.nlm.nih.gov/gene/?term=339559
CYHR1	CYHR1	cysteine and histidine rich 1	50626	https://www.ncbi.nlm.nih.gov/gene/?term=50626
RILPL1	RILPL1	Rab interacting lysosomal protein like 1	353116	https://www.ncbi.nlm.nih.gov/gene/?term=353116
RAB42	RAB42	RAB42, member RAS oncogene family	115273	https://www.ncbi.nlm.nih.gov/gene/?term=115273
PLA2G4E	PLA2G4E	phospholipase A2 group IVE	123745	https://www.ncbi.nlm.nih.gov/gene/?term=123745
LAMTOR 4	LAMTOR4	late endosomal/lysosomal adaptor, MAPK and MTOR activator 4	389541	https://www.ncbi.nlm.nih.gov/gene/?term=389541
PLSCR1	PLSCR1	phospholipid scramblase 1	5359	https://www.ncbi.nlm.nih.gov/gene/?term=5359
SELL	SELL	selectin L	6402	https://www.ncbi.nlm.nih.gov/gene/?term=6402
NDOR1	NDOR1	NADPH dependent diflavin oxidoreductase 1	27158	https://www.ncbi.nlm.nih.gov/gene/?term=27158
FBLL1	FBLL1	fibrillarin like 1	345630	https://www.ncbi.nlm.nih.gov/gene/?term=345630
CLEC20A	CLEC20A	C-type lectin domain containing 20A	400797	https://www.ncbi.nlm.nih.gov/gene/?term=400797
CC2D2B	CC2D2B	coiled-coil and C2 domain containing 2B	387707	https://www.ncbi.nlm.nih.gov/gene/?term=387707
QRFP	QRFP	pyroglutamylated RFamide peptide	347148	https://www.ncbi.nlm.nih.gov/gene/?term=347148
RBM34	RBM34	RNA binding motif protein 34	23029	https://www.ncbi.nlm.nih.gov/gene/?term=23029
SLC15A5	SLC15A5	solute carrier family 15 member 5	729025	https://www.ncbi.nlm.nih.gov/gene/?term=729025
ALKBH2	ALKBH2	alkB homolog 2, alpha-ketoglutarate dependent dioxygenase	121642	https://www.ncbi.nlm.nih.gov/gene/?term=121642
NUGGC	NUGGC	nuclear GTPase, germinal center associated	389643	https://www.ncbi.nlm.nih.gov/gene/?term=389643
WDSUB1	WDSUB1	WD repeat, sterile alpha motif and U-box domain containing 1	151525	https://www.ncbi.nlm.nih.gov/gene/?term=151525
SEMA4A	SEMA4A	semaphorin 4A	64218	https://www.ncbi.nlm.nih.gov/gene/?term=64218
XPNPEP 3	XPNPEP3	X-prolyl aminopeptidase 3	63929	https://www.ncbi.nlm.nih.gov/gene/?term=63929
PIK3R4	PIK3R4	phosphoinositide-3-kinase regulatory subunit 4	30849	https://www.ncbi.nlm.nih.gov/gene/?term=30849
SLC6A9	SLC6A9	solute carrier family 6 member 9	6536	https://www.ncbi.nlm.nih.gov/gene/?term=6536

ZSCAN25	ZSCAN25	zinc finger and SCAN domain containing 25	221785	https://www.ncbi.nlm.nih.gov/gene/?term=221785
ZNF420	ZNF420	zinc finger protein 420	147923	https://www.ncbi.nlm.nih.gov/gene/?term=147923
ARRDC1	ARRDC1	arrestin domain containing 1	92714	https://www.ncbi.nlm.nih.gov/gene/?term=92714
PCBP2	PCBP2	poly(rC) binding protein 2	5094	https://www.ncbi.nlm.nih.gov/gene/?term=5094
PDCD1LG2	PDCD1LG2	programmed cell death 1 ligand 2	80380	https://www.ncbi.nlm.nih.gov/gene/?term=80380
CFAP43	CFAP43	cilia and flagella associated protein 43	80217	https://www.ncbi.nlm.nih.gov/gene/?term=80217
SLC22A12	SLC22A12	solute carrier family 22 member 12	116085	https://www.ncbi.nlm.nih.gov/gene/?term=116085
ZNF667	ZNF667	zinc finger protein 667	63934	https://www.ncbi.nlm.nih.gov/gene/?term=63934
HOXC4	HOXC4	homeobox C4	3221	https://www.ncbi.nlm.nih.gov/gene/?term=3221
UVRAG	UVRAG	UV radiation resistance associated	7405	https://www.ncbi.nlm.nih.gov/gene/?term=7405
NTRK1	NTRK1	neurotrophic receptor tyrosine kinase 1	4914	https://www.ncbi.nlm.nih.gov/gene/?term=4914
ZXDB	ZXDB	zinc finger X-linked duplicated B	158586	https://www.ncbi.nlm.nih.gov/gene/?term=158586
ZNF652	ZNF652	zinc finger protein 652	22834	https://www.ncbi.nlm.nih.gov/gene/?term=22834
EPS8L3	EPS8L3	EPS8 like 3	79574	https://www.ncbi.nlm.nih.gov/gene/?term=79574
ZFP2	ZFP2	ZFP2 zinc finger protein	80108	https://www.ncbi.nlm.nih.gov/gene/?term=80108
ARMCX6	ARMCX6	armadillo repeat containing X-linked 6	54470	https://www.ncbi.nlm.nih.gov/gene/?term=54470
EFCAB2	EFCAB2	EF-hand calcium binding domain 2	84288	https://www.ncbi.nlm.nih.gov/gene/?term=84288
GLYATL3	GLYATL3	glycine-N-acyltransferase like 3	389396	https://www.ncbi.nlm.nih.gov/gene/?term=389396
GIGYF2	GIGYF2	GRB10 interacting GYF protein 2	26058	https://www.ncbi.nlm.nih.gov/gene/?term=26058
RNASE13	RNASE13	ribonuclease A family member 13 (inactive)	440163	https://www.ncbi.nlm.nih.gov/gene/?term=440163
LNP1	LNP1	leukemia NUP98 fusion partner 1	348801	https://www.ncbi.nlm.nih.gov/gene/?term=348801
ACKR1	ACKR1	atypical chemokine receptor 1 (Duffy blood group)	2532	https://www.ncbi.nlm.nih.gov/gene/?term=2532
CHUK	CHUK	conserved helix-loop-helix ubiquitous kinase	1147	https://www.ncbi.nlm.nih.gov/gene/?term=1147
SIPA1	SIPA1	signal-induced proliferation-associated 1	6494	https://www.ncbi.nlm.nih.gov/gene/?term=6494
SYNJ2BP	SYNJ2BP	synaptojanin 2 binding protein	55333	https://www.ncbi.nlm.nih.gov/gene/?term=55333
ADAT3	ADAT3	adenosine deaminase, tRNA specific 3	113179	https://www.ncbi.nlm.nih.gov/gene/?term=113179

DDX47	DDX47	DEAD-box helicase 47	51202	https://www.ncbi.nlm.nih.gov/gene/?term=51202
MBLAC1	MBLAC1	metallo-beta-lactamase domain containing 1	255374	https://www.ncbi.nlm.nih.gov/gene/?term=255374
FOXI3	FOXI3	forkhead box I3	344167	https://www.ncbi.nlm.nih.gov/gene/?term=344167
NOTO	NOTO	notochord homeobox	344022	https://www.ncbi.nlm.nih.gov/gene/?term=344022
GPR33	GPR33	G protein-coupled receptor 33 (gene/pseudogene)	2856	https://www.ncbi.nlm.nih.gov/gene/?term=2856
LRRC69	LRRC69	leucine rich repeat containing 69	100130742	https://www.ncbi.nlm.nih.gov/gene/?term=100130742
ACSM4	ACSM4	acyl-CoA synthetase medium chain family member 4	341392	https://www.ncbi.nlm.nih.gov/gene/?term=341392
GRXCR1	GRXCR1	glutaredoxin and cysteine rich domain containing 1	389207	https://www.ncbi.nlm.nih.gov/gene/?term=389207
FASTKD5	FASTKD5	FAST kinase domains 5	60493	https://www.ncbi.nlm.nih.gov/gene/?term=60493
SKOR2	SKOR2	SKI family transcriptional corepressor 2	652991	https://www.ncbi.nlm.nih.gov/gene/?term=652991
CEBPZOS	CEBPZOS	CEBPZ opposite strand	100505876	https://www.ncbi.nlm.nih.gov/gene/?term=100505876
VAMP2	VAMP2	vesicle associated membrane protein 2	6844	https://www.ncbi.nlm.nih.gov/gene/?term=6844
PRR29	PRR29	proline rich 29	92340	https://www.ncbi.nlm.nih.gov/gene/?term=92340
SLC26A6	SLC26A6	solute carrier family 26 member 6	65010	https://www.ncbi.nlm.nih.gov/gene/?term=65010
TEX50	TEX50	testis expressed 50	730159	https://www.ncbi.nlm.nih.gov/gene/?term=730159
MCIDAS	MCIDAS	multiciliate differentiation and DNA synthesis associated cell cycle protein	345643	https://www.ncbi.nlm.nih.gov/gene/?term=345643
PSMB8	PSMB8	proteasome subunit beta 8	5696	https://www.ncbi.nlm.nih.gov/gene/?term=5696
TMEM250	TMEM250	transmembrane protein 250	90120	https://www.ncbi.nlm.nih.gov/gene/?term=90120
STPG4	STPG4	sperm-tail PG-rich repeat containing 4	285051	https://www.ncbi.nlm.nih.gov/gene/?term=285051
RPP21	RPP21	ribonuclease P/MRP subunit p21	79897	https://www.ncbi.nlm.nih.gov/gene/?term=79897
ARPC4	ARPC4	actin related protein 2/3 complex subunit 4	10093	https://www.ncbi.nlm.nih.gov/gene/?term=10093
C8orf58	C8orf58	chromosome 8 open reading frame 58	541565	https://www.ncbi.nlm.nih.gov/gene/?term=541565

NFS1	NFS1	NFS1, cysteine desulfurase	9054	https://www.ncbi.nlm.nih.gov/gene/?term=9054
LY75-CD302	LY75-CD302	LY75-CD302 readthrough	100526664	https://www.ncbi.nlm.nih.gov/gene/?term=100526664
TMEM200B	TMEM200B	transmembrane protein 200B	399474	https://www.ncbi.nlm.nih.gov/gene/?term=399474
TRIL	TRIL	TLR4 interactor with leucine rich repeats	9865	https://www.ncbi.nlm.nih.gov/gene/?term=9865
ATP23	ATP23	ATP23 metallopeptidase and ATP synthase assembly factor homolog	91419	https://www.ncbi.nlm.nih.gov/gene/?term=91419
DPEP2NB	DPEP2NB	DPEP2 neighbor	100131303	https://www.ncbi.nlm.nih.gov/gene/?term=100131303
RNF115	RNF115	ring finger protein 115	27246	https://www.ncbi.nlm.nih.gov/gene/?term=27246
MEI4	MEI4	meiotic double-stranded break formation protein 4	101928601	https://www.ncbi.nlm.nih.gov/gene/?term=101928601
DCP1A	DCP1A	decapping mRNA 1A	55802	https://www.ncbi.nlm.nih.gov/gene/?term=55802
SCRIB	SCRIB	scribble planar cell polarity protein	23513	https://www.ncbi.nlm.nih.gov/gene/?term=23513
PROP1	PROP1	PROP paired-like homeobox 1	5626	https://www.ncbi.nlm.nih.gov/gene/?term=5626
DERL3	DERL3	derlin 3	91319	https://www.ncbi.nlm.nih.gov/gene/?term=91319
SDCCAG8	SDCCAG8	serologically defined colon cancer antigen 8	10806	https://www.ncbi.nlm.nih.gov/gene/?term=10806
APBA2	APBA2	amyloid beta precursor protein binding family A member 2	321	https://www.ncbi.nlm.nih.gov/gene/?term=321
DACH1	DACH1	dachshund family transcription factor 1	1602	https://www.ncbi.nlm.nih.gov/gene/?term=1602
HEATR9	HEATR9	HEAT repeat containing 9	256957	https://www.ncbi.nlm.nih.gov/gene/?term=256957
SALL3	SALL3	spalt like transcription factor 3	27164	https://www.ncbi.nlm.nih.gov/gene/?term=27164
ARHGEF26	ARHGEF26	Rho guanine nucleotide exchange factor 26	26084	https://www.ncbi.nlm.nih.gov/gene/?term=26084
SCG5	SCG5	secretogranin V	6447	https://www.ncbi.nlm.nih.gov/gene/?term=6447
GSTT1	GSTT1	glutathione S-transferase theta 1	2952	https://www.ncbi.nlm.nih.gov/gene/?term=2952
CISD3	CISD3	CDGSH iron sulfur domain 3	284106	https://www.ncbi.nlm.nih.gov/gene/?term=284106
PPP4R4	PPP4R4	protein phosphatase 4 regulatory subunit 4	57718	https://www.ncbi.nlm.nih.gov/gene/?term=57718
MYO19	MYO19	myosin XIX	80179	https://www.ncbi.nlm.nih.gov/gene/?term=80179

OR2S2	OR2S2	olfactory receptor family 2 subfamily S member 2 (gene/pseudogene)	56656	https://www.ncbi.nlm.nih.gov/gene/?term=56656
MATR3	MATR3	matrin 3	9782	https://www.ncbi.nlm.nih.gov/gene/?term=9782
CORO7	CORO7	coronin 7	79585	https://www.ncbi.nlm.nih.gov/gene/?term=79585
ZNF445	ZNF445	zinc finger protein 445	353274	https://www.ncbi.nlm.nih.gov/gene/?term=353274
EXOC3L2	EXOC3L2	exocyst complex component 3 like 2	90332	https://www.ncbi.nlm.nih.gov/gene/?term=90332
LYNX1-SLURP2	LYNX1-SLURP2	LYNX1-SLURP2 readthrough	111188157	https://www.ncbi.nlm.nih.gov/gene/?term=111188157
SPEM1	SPEM1	spermatid maturation 1	374768	https://www.ncbi.nlm.nih.gov/gene/?term=374768
CARMIL3	CARMIL3	capping protein regulator and myosin 1 linker 3	90668	https://www.ncbi.nlm.nih.gov/gene/?term=90668
SMIM41	SMIM41	small integral membrane protein 41	113523638	https://www.ncbi.nlm.nih.gov/gene/?term=113523638
AGPAT5	AGPAT5	1-acylglycerol-3-phosphate O-acyltransferase 5	55326	https://www.ncbi.nlm.nih.gov/gene/?term=55326
REC8	REC8	REC8 meiotic recombination protein	9985	https://www.ncbi.nlm.nih.gov/gene/?term=9985
RNF31	RNF31	ring finger protein 31	55072	https://www.ncbi.nlm.nih.gov/gene/?term=55072
TMEM249	TMEM249	transmembrane protein 249	340393	https://www.ncbi.nlm.nih.gov/gene/?term=340393
NFATC4	NFATC4	nuclear factor of activated T cells 4	4776	https://www.ncbi.nlm.nih.gov/gene/?term=4776
AGBL2	AGBL2	ATP/GTP binding protein like 2	79841	https://www.ncbi.nlm.nih.gov/gene/?term=79841
SEMA7A	SEMA7A	semaphorin 7A (John Milton Hagen blood group)	8482	https://www.ncbi.nlm.nih.gov/gene/?term=8482
NANOS3	NANOS3	nanos C2HC-type zinc finger 3	342977	https://www.ncbi.nlm.nih.gov/gene/?term=342977
ISLR	ISLR	immunoglobulin superfamily containing leucine rich repeat	3671	https://www.ncbi.nlm.nih.gov/gene/?term=3671
TMEM95	TMEM95	transmembrane protein 95	339168	https://www.ncbi.nlm.nih.gov/gene/?term=339168
C1R	C1R	complement C1r	715	https://www.ncbi.nlm.nih.gov/gene/?term=715

Supplemental File 11: Genes without unique ID

Gene name
ACP3
SARS1
HROB
MTSS2
MARCHF7
MT-ND4
KIFBP
B3GALT9
GFUS
PERCC1

Supplemental file 12: Link to an interactive .html file showing all the data

The following link includes the enriched Panther terms, the enrichment P-values (hypergeometric test), Q-values (FDR) for the test, the Panther term set size, and the enrichment ratio.

Download at: https://osf.io/download/nfvmx/

Supplemental file 13: Fig. S1

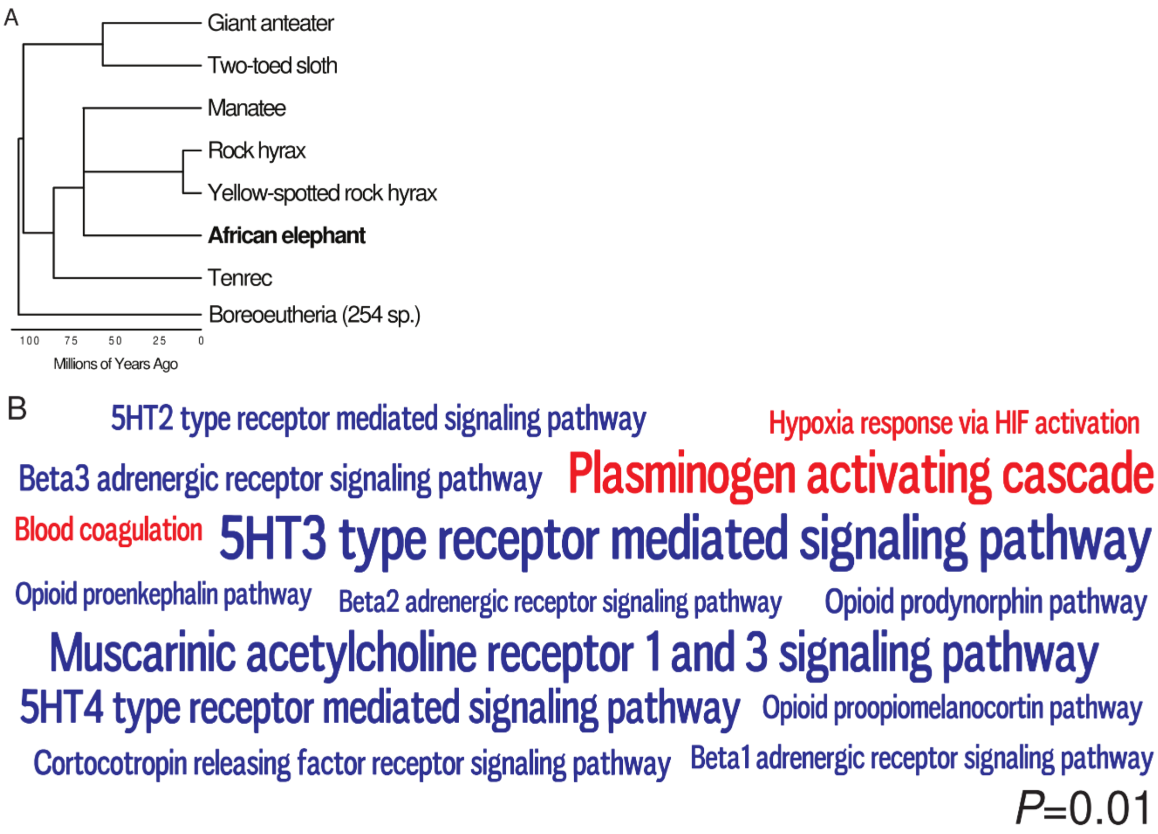

Fig. S1. Positive selection on genes associated with self-domestication in African elephants. (A) Phylogeny of Afrotherian species included in tests for positive selection acting on elephant protein-coding genes. Note that the phylogenetic relationships between elephant, hyrax, and manatee lineages are uncertain, and these species were included as an unresolved polytomy in selection tests. Phylogenetic relationships are shown with branches proportional to divergence times. The other 254 species not shown are within the Boreoeutheria, the sister lineage to Afrotheria. (B) Word Cloud showing Panther pathways in which genes with positive selection in elephants are enriched. Terms are drawn proportional to −log10 P-value (see Inset scale) and colored blue if related to domestication.
